# Supplementary material for: Refining models of archaic admixture in Eurasia with ArchaicSeeker 2.0
Source: Nat Commun. 2021 Oct 29;12:6232. doi: 10.1038/s41467-021-26503-5 (PMC8556419; doi:10.1038/s41467-021-26503-5)
Supplement: Supplementary file 1 — Supplementary Information [file 41467_2021_26503_MOESM1_ESM.pdf]

SUPPLEMENTARY INFORMATION

Refining models of archaic human admixture in Eurasia with *ArchaicSeeker2.0*

Correspondence and requests for materials should be addressed to Dr. Shuhua Xu (xushua@fudan.edu.cn)

Content

1 Supplementary Note 1: Data Collection 3
2 Supplementary Note 2: *ArchaicSeeker 2.0* Algorithm 13
2.1 Matching Model Calibration 15
2.2 Seeking Algorithm 19
2.3 Matching Algorithm 21
2.4 Segments Connection and Filtration 23
2.5 Parameters Estimation 24
2.6 Introgression History Inference Method 25
2.7 Phasing, Sequencing Error, and Modern Human Contamination 29
2.8 Comparison of Available Archaic Inference Methods 30
3 Supplementary Note 3: Simulations 32
3.1 Introgressions from Single Archaic Lineage 34
3.1.1 Scenarios with Diverse Divergence Time 34
3.1.2 Scenarios with Diverse Introgression Time 36
3.1.3 Scenarios with Diverse Introgression Proportion 38
3.1.4 Scenarios of Two-Waves Introgression from Single Archaic Lineage 40
3.2 Introgressions from both Denisovan and Neanderthal 43
3.3 Introgression from a Deep Divergent Archaic Lineage 45
3.4 Summary Tables of Simulation Scenarios 47
4 Supplementary Note 4: Simulation Evaluations 51
4.1 Introgressed Segments Detection 51
4.1.1 Length-Based Evaluation 52
4.1.2 SNP-Based Evaluation 70
4.1.3 Segment-Based Evaluation 93
4.1.4 Matching Algorithm Evaluation 99
4.2 Introgression History Inference 105
4.2.1 Segment Length Distribution 106
4.2.2 Inference with the Ground-truth Introgressed Segments 114
4.2.3 Inference with the Inferred Segments 122
4.3 Further Simulation Validations 132
4.3.1 Simulations with *msprime* 132
4.3.2 Incomplete Lineage Sorting 133

4.3.3 D Statistics 134
5 Supplementary Note 5: Empirical Data Analysis 138
5.1 Archaic Sequences Detection 138
5.2 Archaic Introgression Model Inference 163
5.3 Archaic Ancestry Sharing 168
5.3.1 Archaic Ancestry Sharing Definition...168
5.3.2 Archaic Ancestry Sharing Properties...172
5.4 Introgression Desert 181
6 Reference…………………………………………………………………………185

1 Supplementary Note 1: Data Collection

In this analysis, we collected high coverage sequencing genomic data of three ancient samples [1-3] and thousands of modern human sequencing data from worldwide populations [4-6]. For the three ancient samples, two of them are archaic hominins and the other belongs to anatomically modern humans (AMH). The two archaic hominins were Altai Denisovan [3] and Altai Neanderthal [2]. The recent published high coverage archaic hominin sequencing data Vindija33.19 Neanderthal [7] was not used in our analysis due to the following rationale: this newly published archaic genome data did not use the uracil-DNA-glycosylase when they extracted the ancient DNA; instead, they developed a novel calling method to generate the SNPs data [7]. In the Vindija33.19 high coverage sequencing analysis, they redid the calling of Altai Neanderthal with this new calling method and QCs. We compared the different versions of the high coverage sequenced archaic genome (Supplementary Table 1.1). As a result, the number of SNPs in the recent version (Kay Prüfer 2017, Science) is much less than that of the previous version. To involve more makers, we excluded Vindija33.19 in our analysis. The ancient AMH sample is an ancient Siberian Ust'-Ishim who lived in Western Siberia 45,000 years ago [1].

Supplementary Table 1.1 Number of SNVs of the Different Versions Archaic Genomes

**Version**	Archaic Hominin	0/0	0/1	1/1	1/2	non-missing	non 0/0
Kay Prüfer 2014, Nature	Altai Neanderthal	2,629,547,064	1,983,462	4,072,560	6,385	2,635,609,471	6,062,407
Kay Prüfer 2017, Science	Altai Neanderthal	1,762,259,184	384,644	2,387,206	2,462	1,765,033,496	2,774,312
Matthias Meyer, 2010, Science	Altai Denisovan	2,611,580,063	1,808,894	4,169,135	4,778	2,617,562,870	5,982,807
Kay Prüfer 2017, Science	Altai Denisovan	1,761,514,441	417,315	2,474,442	2,234	1,764,408,432	2,893,991
Kay Prüfer 2017, Science	Vindija33.19	1,761,763,557	359,470	2,446,360	1,581	1,764,570,968	2,807,411

We also collected modern human genomic data from three datasets: the 1000 Genome Project (KGP) [4], Simons Genome Diversity Project (SGDP) [5], and the Estonian Biocentre Human Genome Diversity Panel (EGDP) [6]. Samples from these three datasets cover almost all the continental populations and most of the representative isolated populations.

Among the three modern human genomic datasets, the KGP mainly concerns continental populations and it covers 26 populations from 5 continents (Supplementary Table 1.4). For each population, the sample size is around 100. We used this dataset to do the analysis requiring large sample sizes. The other two datasets cover a much more diverse set of populations compared with the KGP. The SGDP contains 279 samples from 130 populations (Only for B and S Panel, Supplementary Table 1.2); The EGDP includes 402 samples from 126 populations (Supplementary Table 1.3). In these two datasets, the sample size of each population was typically small. Except for a few populations, such as Papuan in the SGDP, the sample size of most populations is only two or three. We used these two datasets to analyze with small sample sizes.

The datasets of the two archaic hominins and Ust'-Ishim data [1] were downloaded from the website of the Max Plank Institute for Evolutionary Anthropology (two archaic hominins: http://cdna.eva.mpg.de/neandertal/altai; Ust'-Ishim: http://cdna.eva.mpg.de/ust-ishim/). The KGP [4] dataset was obtained from the FTP site of KGP (ftp://ftp.1000genomes.ebi.ac.uk/vol1/ftp/release/20130502) and EGDP data [6] was downloaded from the website of Estonian Biocentre (http://evolbio.ut.ee/CGgenomes_VCF). For the data from SGDP [5], we downloaded the sra format from NCBI/EBI with the accession number (PRJEB9586); then we converted the sra format to the fastq format and conducted a joint calling with another 1508 unpublished sequencing data generated by our group.

Since *ArchaicSeeker 2.0* requires the haplotype information of the introgressed modern human populations, we performed a phasing procedure for several datasets in which the haplotype information was unknown. For the KGP and EGDP datasets, the haplotype information of the original downloaded data was already known. We phased the Ust'-Ishim and the SGDP dataset using the software *SHAPEIT2* [8] software with the

default parameters and the 1000 Genome Project as the phasing reference. We removed multi-allelic SNPs, indels, and structural variants from the vcf files. Only the bi-allelic SNPs were kept for the downstream analysis.

Supplementary Table 1.2 Simons Genome Diversity Project (SGDP) Populations

Population Label	Continent/Region
BantuHerero	Africa
BantuKenya	Africa
BantuTswana	Africa
Biaka	Africa
Dinka	Africa
Esan	Africa
Gambia	Africa
Ju_hoan_North	Africa
Khomani_San	Africa
Luhya	Africa
Luo	Africa
Mandenka	Africa
Masai	Africa
Mbuti	Africa
Mende	Africa
Yoruba	Africa
Mozabite	North Africa
Saharawi	North Africa
Somali	North Africa
Chane	America
Karitiaa	America
Mayan	America
Mixe	America
Mixtec	America
Piapoco	America
Pima	America
Quechua	America
Surui	America
Zapotec	America
Aleut	Central Asia Siberia
Altaia	Central Asia Siberia
Chukchi	Central Asia Siberia
Eskimo_Chaplin	Central Asia Siberia

Eskimo_Naukan	Central Asia Siberia
Eskimo_Sireniki	Central Asia Siberia
Even	Central Asia Siberia
Itelman	Central Asia Siberia
Kyrgyz	Central Asia Siberia
Mansi	Central Asia Siberia
Mongola	Central Asia Siberia
Tlingit	Central Asia Siberia
Tubalar	Central Asia Siberia
Ulchi	Central Asia Siberia
Yakut	Central Asia Siberia
Ami	East Asia
Atayal	East Asia
Burmese	East Asia
Cambodia	East Asia
Dai	East Asia
Daur	East Asia
Han	East Asia
Hezhen	East Asia
Japanese	East Asia
Kinh	East Asia
Korean	East Asia
Lahu	East Asia
Miao	East Asia
Naxi	East Asia
Oroqen	East Asia
She	East Asia
Thai	East Asia
Tu	East Asia
Tujia	East Asia
Uygur	East Asia
Xibo	East Asia
Yi	East Asia
Australia	Oceania
Bougainville	Oceania
Papuan	Oceania
Dusun	Oceania
Hawaiia	Oceania
Igorot	Oceania
Maori	Oceania
Balochi	South Asia
Bengali	South Asia
Brahmin	South Asia
Brahui	South Asia

Burusho	South Asia
Hazara	South Asia
Irula	South Asia
Kalash	South Asia
Kapu	South Asia
Khonda_Dora	South Asia
Kusunda	South Asia
Madiga	South Asia
Makrani	South Asia
Mala	South Asia
Pathan	South Asia
Punjabi	South Asia
Relli	South Asia
Sindhi	South Asia
Yadava	South Asia
Abkhasia	West Eurasia
Adygei	West Eurasia
Albania	West Eurasia
Armenia	West Eurasia
Basque	West Eurasia
BedouinB	West Eurasia
Bergamo	West Eurasia
Bulgaria	West Eurasia
Chechen	West Eurasia
Crete	West Eurasia
Czech	West Eurasia
Druze	West Eurasia
English	West Eurasia
Estonia	West Eurasia
Finnish	West Eurasia
French	West Eurasia
Georgia	West Eurasia
Greek	West Eurasia
Hungaria	West Eurasia
Icelandic	West Eurasia
Irania	West Eurasia
Iraqi_Jew	West Eurasia
Jordania	West Eurasia
Lezgin	West Eurasia
North_Ossetia	West Eurasia
Norwegia	West Eurasia
Orcadia	West Eurasia
Palestinia	West Eurasia
Polish	West Eurasia

Russia	West Eurasia
Saami	West Eurasia
Samaritan	West Eurasia
Sardinia	West Eurasia
Spanish	West Eurasia
Tajik	West Eurasia
Turkish	West Eurasia
Tusca	West Eurasia
Yemenite_Jew	West Eurasia

Supplementary Table 1.3 Estonian Biocentre Human Genome Diversity Panel (EGDP) Populations

Population Label	Continent/Region
Congo-pygmies	Africa
Cachi	America
Colla	America
Wichi	America
Abkhazias	Caucasus
Armenias	Caucasus
Avars	Caucasus
Azerbaijanis	Caucasus
Balkars	Caucasus
Circassias	Caucasus
Georgias	Caucasus
Kabardins	Caucasus
Kumyks	Caucasus
Lezgins	Caucasus
North-Ossetias	Caucasus
Tabasarans	Caucasus
Ishkasim	Central Asia
Kazakhs	Central Asia
Kyrgyz	Central Asia
KyrgyzTdj	Central Asia
Rushan-Vanch	Central Asia
Shugnan	Central Asia
Tajiks	Central Asia
Turkmens	Central Asia
Uygurs	Central Asia
Uzbek	Central Asia
Yaghnobi	Central Asia

Population Label	Continent/Region
Albanias	Europe
Bashkirs	Europe
Belarusias	Europe
Chuvashes	Europe
Cossacks	Europe
CossacksKuban	Europe
Croats	Europe
Estonias	Europe
Finnish	Europe
Germans	Europe
Hungarias	Europe
Ingrias	Europe
Karelias	Europe
Komis	Europe
Kryashen-Tatars	Europe
Latvias	Europe
Lithuanias	Europe
Maris	Europe
Mishar-Tatars	Europe
Moldavias	Europe
Mordvins	Europe
Poles	Europe
Roma	Europe
Russias	Europe
Russias-Central	Europe
Russias-North	Europe
Russias-West	Europe
Saami	Europe
Swedes	Europe
Tatars	Europe
Udmurds	Europe
Ukrainiaseast	Europe
Ukrainiasnorth	Europe
Ukrainiaswest	Europe
Vepsas	Europe
Koinanbe	Sahul
Kosipe	Sahul
Altaias	Siberia
Buryats	Siberia
Chukchis	Siberia
Eskimo	Siberia
Evenks	Siberia
EvensMagadan	Siberia

Population Label	Continent/Region
EvensSakha	Siberia
Forest-Nenets	Siberia
Kets	Siberia
Khantys	Siberia
Koryaks	Siberia
Mansis	Siberia
Mongolias	Siberia
Nganasans	Siberia
Sakha	Siberia
Selkups	Siberia
Shor	Siberia
Tundra-Nenets	Siberia
Tuvinias	Siberia
Yakuts	Siberia
Asur	South Asia
Balija	South Asia
Bengali	South Asia
Brahmin	South Asia
Dhaka-mixed-popul	South Asia
Gond	South Asia
Gupta	South Asia
Ho	South Asia
Kapu	South Asia
Kol	South Asia
Kshatriya	South Asia
Kurmi	South Asia
Madhya-Pradesh	South Asia
Malayan	South Asia
Marwadi	South Asia
Orissa	South Asia
Punjab	South Asia
Santhal	South Asia
Tamang	South Asia
Thakur	South Asia
Aeta	Southeast Asia Island
Agta	Southeast Asia Island
Bajo	Southeast Asia Island
Batak	Southeast Asia Island
Dusun	Southeast Asia Island
Igorot	Southeast Asia Island
Lebbo	Southeast Asia Island
Luzon	Southeast Asia Island
Murut	Southeast Asia Island

Population Label	Continent/Region
Vizayan	Southeast Asia Island
Burmese	Southeast Asia Mainland
Vietnamesecentral	Southeast Asia Mainland
Vietnamesenorth	Southeast Asia Mainland
Vietnamesesouth	Southeast Asia Mainland
Arabs-Israel-1	West Asia
Arabs-Israel-2	West Asia
Assyrias	West Asia
Druze	West Asia
Iranias	West Asia
Jordanias	West Asia
Lebanese	West Asia
Saudi-Arabias	West Asia

Supplementary Table 1.4 Non-African 1000 Genome Project (KGP) Populations

Population Label	Continent/Region
ACB	America
ASW	America
CLM	America
MXL	America
PEL	America
PUR	America
CDX	East Asia
CHB	East Asia
CHS	East Asia
JPT	East Asia
KHV	East Asia
CEU	Europe
FIN	Europe
GBR	Europe
IBS	Europe
TSI	Europe
BEB	South Asia
GIH	South Asia
ITU	South Asia
PJL	South Asia
STU	South Asia

Since there could be a potential effect of the SNV calling pipelines as three data sets from different sources are used for our analysis, we evaluated potential batch effects between them by a PCA (see Supplementary Figure 1.1). In the PCA plots, different colors stand for different continents and different shapes stand for different datasets. As data were clustered by colors, not by shapes, it indicates that the likelihood that our analysis was cofounded by such a batch effect is low.

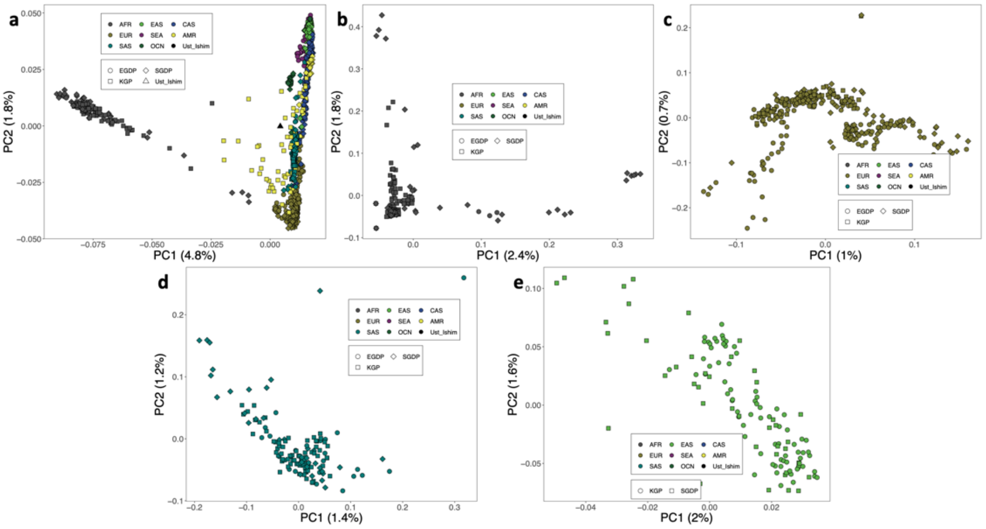

Supplementary Figure 1.1 **PCA of the Four Datasets.** We applied PCA to the four datasets with different continents populations: a. global populations; b. African populations; c. American populations; d. South Asian populations; e East Asian populations. Here different shapes stand for different datasets: circle stands for EGDP; rectangle for KGP, diamond for SGDP, and triangle for Ust. Abbreviations: AFR (Africa), EAS (East Asia), CAS (Central Aisa Siberia), EUR (Europe), SEA (Southeast Aisa), AMR (America), SAS (South Asia), OCN (Oceania).

2 Supplementary Note 2: *ArchaicSeeker 2.0* Algorithm

In this note, we described a novel method and its corresponding software called *ArchaicSeeker 2.0*. This software is designed to detect the alien genomic regions in modern human genomes that were derived from the archaic lineages and to infer the introgression history. An evaluation study of the software using massive simulation data indicates an excellent performance: the median value of the precision was 93.0% (95% CI, 89.4%–95.9%), that of the TPR was 90.4% (95% CI, 84.1%–94.1%), and that of the FPR was 0.14% (95% CI, 0.07%–0.22%) . For the introgression history analysis, the history was correctly inferred by our software for most cases (122/144, 84.7%) of our simulation scenarios.

Compared with the original version of *ArchaicSeeker* [9], the new updated version has three major improvements.

Firstly, it can automatically determine the boundary of each introgressed segment. In the original version, we analyzed data by artificially spitting genomes into different chunks of the same size. Therefore, the boundary of an introgressed segment is determined by the boundaries of these chunks. The new version seeks introgressed segments with a Hidden Markov Model, which determines the border between introgressed and non-introgressed segments automatically.

Secondly, the new version can find the proper introgression ancestry for each archaic segment simultaneously. The original version analyzed introgression from one specific archaic lineage for each run and it cannot be used to find sequences derived from some unknown archaic hominins. Analyses of introgression from different archaic hominins were independent and one specific segment could be inferred as introgression from different archaic hominins in runs with different archaic references. To solve this problem, we proposed a matching algorithm. The framework of this matching algorithm is much flexible, and it allows inferring the archaic segments derived from different archaic lineages simultaneously and one segment will uniquely match to a specific archaic lineage. In other words, we could infer introgressed segments of Neanderthal, Denisovan, and some other unknown archaic hominins at the same time.

Thirdly, the new version incorporates an additional functional module on inferring introgression history. Here, we improved the General Discrete Admixture Model described in our previous studies [10-12]. Instead of allowing only one wave admixture from the modern human, the model implemented in *ArchaicSeeker 2.0* allows us to infer introgression histories with multiple archaic introgression events.

In our method, we used African whole-genome sequencing data (YRI) and archaic high coverage whole-genome sequencing data (Altai Denisovan, Altai Neanderthal) as the references. We inferred introgressed sequences at the haplotype level, so the to-be-inferred modern human population data should be phased. For the reference panel, haplotype information is not required, so users can use unphased archaic data as input.

Users should provide prior phylogenetic relationship information of the input populations as the matching model. We will use this information to match each candidate segment to a specific archaic lineage. For example, when we analyzed the data with three high coverage archaic hominins and YRI, the phylogenetic model (Supplementary Figure 2.1) could be provided in the *Newick* format (Jin, Wang et al. 2012) exemplified by the following one:

((YRI:100,Test:100):557.5,(Denisovan:340,Altai:300):237.5)

The unit in the matching model could be in generations, one thousand years, or some other units specified by the users. Our method will calibrate the model with genomic sequencing data automatically. Then it will match all the candidate segments, which were discovered by the seeking algorithm to a specific ancestry, including leaf node (Denisovan, Altai etc.) or internal node (such as Denisovan _Altai etc.).

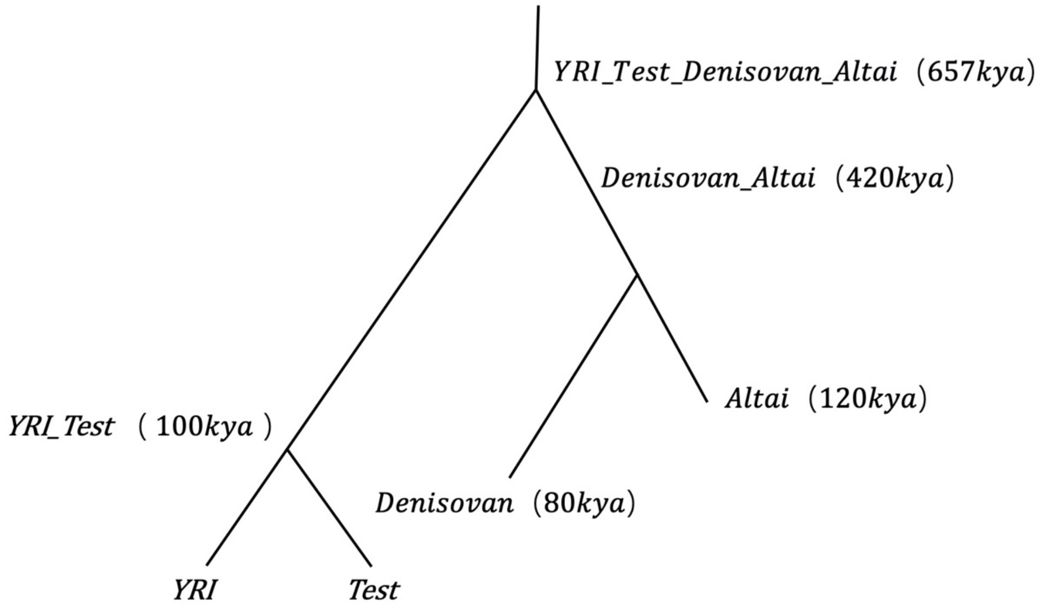

Supplementary Figure 2.1 **An example of the matching model.** This model is used to match each candidate archaic segment to a specific ancestry. In this model, each leaf node corresponds to a population in the analysis and the internal node stands for the ancestor of its child nodes. For example, Denisovan_Altai stands for the ancestor of Denisovan and Altai. The phylogenetic relationship and the divergence time information are obtained from previous studies [2,3,7].

Many model-based methods were very sensitive to the values of model parameters and the selection of appropriate model parameters was often a “skillful” step in data analysis. In contrast, our method provides an EM algorithm [13] to estimate the model parameters, which makes our method more user-friendly and more robust.

2.1 Matching Model Calibration

To match each segment to a specific archaic ancestry, prior phylogenetic relationship information of the input populations is required. This information will be used to match candidate introgressed segments discovered by the seeking algorithm to a proper ancestry. We call this information the matching model (Supplementary Figure 2.1). Often, the topology of the matching model may not be a serious problem, but the branch length (divergence time) may be difficult to provide. Furthermore, the uncertainty of the branch length affects the results of our method greatly. To make our

method more robust, we provide a simple model calibration procedure. We calibrate the matching model with population genomic pairwise differences. To automatically set the root of the tree, an outgroup genome is required in our method. Here, we use the *PanTro5* chimpanzee reference genome [14] as the outgroup.

The matching model is a binary tree with n leaf nodes labeled, N_i $(i = 1,2, \ldots, n)$ and $n-1$ internal nodes labeled, N_i $(i = n+1, n+2, \ldots, 2n-1)$. The edge which connects nodeN_i with its parent is designated asd_i $(i = 1,2, \ldots, 2n-1)$. The distance between any two leaf nodesN_i and N_j is defined asD_{ij} $(i, j = 1,2, \ldots, n; i \neq j)$. The distance between any leaf nodeN_i and the outgroup is defined as R_i $(i = 1,2, \ldots, n)$. D_{ij} andR_i could represent by the summation of certain edges (Supplementary Figure 2.2).

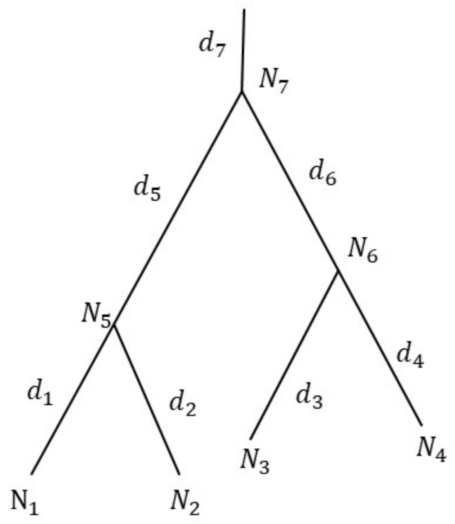

Supplementary Figure 2.2 Sketch map of the matching model. There are 4 leaf nodes (N_1, N_2, N_3, N_4) and 3 internal nodes (N_5, N_6, N_7) in the matching model. Each leaf node stands for a population and an internal node stands for the ancestry of its child nodes. d_i stands for the length of the edge that connecs N_i and its parent node. D_{ij} stands for the distance between leaf noe N_i and leaf node N_j. R_i stands for the distance between leaf node N_i and the outgroup. In this sketch map, the distance between N_1 and N_3 is $D_{13} = d_1 + d_5 + d_6 + d_3$; the distance between N_4 and the outgroup is $R_4 = d_4 + d_6 + d_7$.

In the matching model, each leaf node corresponds to a population. We can

calculate the pairwise population differences using genomic data. The differences between populations i and j are defined as π_{ij} $(i, j = 1,2, \ldots, n; i \neq j)$, which is the conditional probability, given allele frequencies in populations i and j, that two random gene copies, one drawn from population i and the other from population j, are copies of different alleles.

$$\pi_{ij} = \sum_{k=1}^{S} \left((1 - p_{ik}) p_{jk} + p_{ik} \left(1 - p_{jk} \right) \right), \tag{1}$$

where S is the number of SNPs and p_{ik} is the frequency of the derived allele in population i at position k.

To set the location of the root, an outgroup was introduced in this analysis. We defined the difference between population i and the outgroup as Ω_i $(i = 1,2, \ldots, n)$,

$$\Omega_i = \sum_{k=1}^{S} p_{ik}, \tag{2}$$

where S is the number of SNPs; p_{ik} is the frequency of the derived allele in population i at position k.

To make sure the calibrated model is in the same unit as the original model, we estimated a relative mutation rate μ using the genomic data,

$$\mu = \frac{\sum_{i=1}^{n-1} \sum_{j=i+1}^{n} \pi_{ij}}{L * \sum_{i=1}^{n-1} \sum_{j=i+1}^{n} D_{ij}}, \tag{3}$$

where L is the total length of the genome.

Then, we could calculate the observed branch length D'_{ij} between N_i and N_j as

$$D'_{ij} = \frac{\pi_{ij}}{L * \mu}. \tag{4}$$

The observed branch length of any leaf node N_i to the outgroup, R'_i is defined as

follow,

$$R'_i = \frac{\Omega_i}{L\mu}. \quad (5)$$

The genomic differences of any two individuals follow a Poisson distribution. That is, we approximate the pairwise population distance (π) as a Poisson distribution of parameter $\lambda = L\mu D$, where D is the distance of these two populations in the matching model, L is the length of the genome, and μ is the relative mutation rate. L and μ are constant. The probability of observing k different markers is

$$P(\pi = k) = \frac{\lambda^k e^{-\lambda}}{k!}. \quad (6)$$

By comparing the genomic data of any two populations, i and j, we could calculate the population differences, π_{ij}. By comparing the genomic data between population i and the outgroup genome, we could calculate the differences from the outgroup, Ω_i. Since the branch length parameter D_{ij} and R_i are the function of $d_1, d_2, d_3, \dots, d_{2n-1}$, we could get the observation likelihood

$$L(d_1, d_2, d_3, \dots, d_{2n-1}) = \prod_{i=1}^{n-1} \prod_{j=i+1}^{n} \frac{(L\mu D_{ij})^{\pi_{ij}} \times e^{-L\mu D_{ij}}}{\pi_{ij}!} * \prod_{i=1}^{n} \frac{(L\mu R_i)^{\Omega_i} \times e^{-L\mu R_i}}{\Omega_i!}. \quad (7)$$

In the empirical data analysis, we found that π_{ij} and Ω_i were often extreme large, and that affected the resolution of our method, we adjusted the equation (7), by dividing the difference parameters π_{ij} and Ω_i to the product of genome length L and relative mutation rate μ. Then the distribution switch from the difference to the branch length. Similarly, we used the observation of D'_{ij} and R'_i to calculate the likelihood.

By maximize the log-likelihood function, we could estimate the branch length $\widehat{d_i}$. The maximization algorithm is the globally-convergent method-of-moving-asymptotes (MMA) algorithm [15] implemented in *nlopt* package [16].

2.2 Seeking Algorithm

After calibrating the matching model, we start to detect the candidate introgressed segments from the modern human genomes with a Hidden Markov Model (HMM) based method. The basic idea of this process is selecting segments with a large number of alleles not present in African reference genomes, while most of which shared with archaic reference populations. In this seeking algorithm, each haplotype from the modern human population is analyzed parallel with the reference panel.

For a general Hidden Markov Model, it consists of observation states, hidden states, transition probability matrix, emission probability matrix, and initial distribution.

Observation States

Firstly, we selected one haplotype from the modern human population. We classified the status of each SNPs into 4 different observation states according to the test modern human haplotype and the derived allele frequency in the reference panel (Supplementary Table 2.1).

Supplementary Table 2.1 Observation states of the seeking algorithm

State	Archaic	African	Test
**State 1 (Test Pop Specific Markers)**	DAF* = 0	DAF = 0	Derived Allele
**State 2 (Archaic Markers)**	DAF $\neq$ 0	DAF = 0	Derived Allele
**State 3 (AMH Markers)**	DAF = 0	DAF $\neq$ 0	Derived Allele
**State 4 (Common Markers)**		Others	

*DAF means derived allele frequency

State 1 and State 2 are of a high likely carried by the introgressed segments, while State 3 is less likely.

Hidden States

We have two hidden states in this method. State 0 stands for the AMH ancestry and State 1 stands for archaic ancestry.

Transition Probability Matrix

The transition probability matrix defines the probability of transition between the two hidden states. These probabilities are controlled by introgression time T (in generation), introgression proportion α and the genetic distance between adjacent sites i and $i-1$, denoted by r_i (in Morgan):

$$P(a \to b) = \begin{cases} \alpha(1 - e^{-Tr_i}), & a = 0, b = 1 \\ 1 - \alpha(1 - e^{-Tr_i}), & a = 0, b = 0 \\ (1 - \alpha)(1 - e^{-Tr_i}), & a = 1, b = 0 \\ \alpha + (1 - \alpha)e^{-Tr_i}, & a = 1, b = 1 \end{cases}, \quad (8)$$

where a and b are the hidden states of the two adjacent markers. In our model, we set T = 2000 and α = 0.02 as the initial value of this matrix. The genetic distance was calculated by the genetic map from the HapMap Project [17].

Emission Probability Matrix

Emission probability is the probability of observation states conditional on the underlying hidden states. The following table (Supplementary Table 2.2) defines the initial values of the matrix.

Supplementary Table 2.2 Initial values of the emission probability matrix

States	Hidden State 0 (AMH)	Hidden State 1 (Archaic)
Observed State 1	$(1-\varepsilon)\varepsilon$*	$(1-\varepsilon)\varepsilon$
Observed State 2	$(1-\varepsilon)^2$	ε^2
Observed State 3	ε^2	$(1-\varepsilon)^2$
Observed State 4	$(1-\varepsilon)\varepsilon$	$(1-\varepsilon)\varepsilon$

*$\varepsilon \in (0,1)$ is designed as a parameter to control the likelihood of an archaic shared marker derived from the archaic population. Often, we give a large number for this parameter. In our analysis we set $\varepsilon = 0.99$.

Initial Distribution

For the Hidden State 1 (Archaic), we set the initial probability of that state as the introgression proportion α, the initial probability of the Hidden State 0 (AMH) is set to $1-\alpha$. The default value of α is 0.02.

In our method, we use Viterbi Algorithm [18] to infer the hidden states and then find the candidate archaic introgressed segments.

2.3 Matching Algorithm

After applying the seeking algorithm, we select several archaic candidate segments. To infer the accurate ancestry of those candidates, we perform a matching procedure. With the matching algorithm, we match every candidate segment to a specific edge in the matching model.

Previous methods classified the introgressed archaic segments into several specific archaic hominin catalogs, such as Denisovan ancestry, Neanderthal ancestry, or Unclassified. These kinds of methods classified segments by comparing the segmented genome with the sequenced Denisovan or Neanderthal genome directly [19-21].

The description of ancestry maybe not precise. In most cases, introgressed sequences may not be inherited from the sequenced archaic hominin directly. A better way to describe the ancestral information of one introgressed segment is by providing both the closest related ancestry and the corresponding divergence time.

Here, we propose a likelihood-based matching algorithm to match every candidate introgressed segments to a proper ancestry and infer the divergence time to the ancestry.

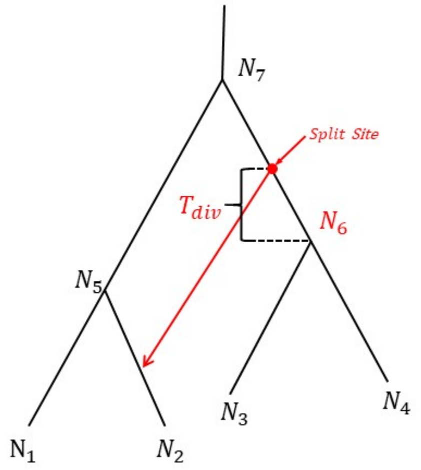

Supplementary Figure 2.3 **Sketch map of the introgression scenarios.** In this figure, the red line indicates the introgression direction. The red dot stands for the so-called *split site*. The introgression node is N_6, which is the ancestor of N_3 and N_4. The relative divergence time T_{div} is the time between the split-site and the introgression node N_6.

For the ancestry of a specific candidate segment, we could find a *split site* on the tree (Supplementary Figure 2.3), which stands for the divergence place of this segment to the closest ancestry. The closest child node to this split site is defined as the introgression node (N_6 in Supplementary Figure 2.3). The distance between the *split site* and introgression node is the relative divergence time T_{div}. We assume any nodes ($N_1 \sim N_7$ in Supplementary Figure 2.3) could be the introgression node. For different introgression nodes or introgressed ancestries, the topologies of the introgression model are different. By calculating the likelihood of every possible introgression topologies, we can find the most likely one by the likelihood value and calculate its corresponding T_{div}.

When the introgression node is N_i, the distance between the candidate segment and leaf node N_j defined as A_{ij} $(i = 1,2, \ldots ,2n - 1; j = 1,2,..n)$. A_{ij} is a parameter related to T_{div} and the branch length d.

Then, we calculate the differences between this candidate segment and every population. Suppose this segment starts at position s and end at position z,

$$\Pi_i = \sum_{k=s}^{z} \sum_{l=0}^{1} \eta_k^l p_{ik}^l , \tag{9}$$

where ϖ_i is the difference between the candidate segment and the population N_i. $\eta_k^l = \begin{cases} 0, & l = c_k \\ 1, & l \neq c_k \end{cases}$ is an indicator function. c_k stands for the allele in the candidate segment at position k. p_{ik}^l is the frequency of allele l in population i at position k.

Next, we calculate the likelihood of the genomic differences under all possible introgression topologies. For an introgression topology with introgression node N_i, we could calculate the likelihood of this specific topology.

$$L_i(T_{div\,i}) = \prod_{j=1}^{n} \frac{\left(LuA_{ij}\right)^{\omega_j} \times e^{-LuA_{ij}}}{\omega_j!}. \tag{10}$$

Similar reason with equation (7), we adjusted the genomic difference parameters to branch length parameters. Then, by maximizing the log-likelihood function, we could get the estimation of the divergence time $\hat{T}_{div\,i}$ of introgression topology with introgression node N_i. The maximization algorithm is the globally-convergent method-of-moving-asymptotes (MMA) algorithm [15] implemented in *nlopt* package [16].

Then we compare the likelihood of different introgression topologies and find the largest one.

2.4 Segments Connection and Filtration

After the matching procedure, we filter out the smaller segment and connect segments with a smaller gap.

As the length of ancestral segments in an admixed population follows an exponential distribution[10] and Archaic introgression could be treated as a special case of population admixture, the length of introgressed segments, $s_{archaic}$ and that of the AMH segments, s_{AMH} should follow an exponential distribution related to introgression time T and introgression proportion α.

$$f(s_{archaic}; T, \alpha) = (1-\alpha)Te^{-(1-\alpha)Ts_{archaic}}, \tag{11}$$

$$f(s_{AMH}; T, \alpha) = \alpha Te^{-\alpha Ts_{AMH}}. \tag{12}$$

Here we performed a two tailed-test for the introgressed segment length with a p-value 0.05. The 95% confidence interval of the introgressed segments and the AMH segments are

$$\left[\frac{-\ln(0.975)}{(1-\alpha)T}, \frac{-\ln(0.025)}{(1-\alpha)T}\right], \tag{13}$$

and

$$\left[\frac{-\ln(0.975)}{\alpha T}, \frac{-\ln(0.025)}{\alpha T}\right], \tag{14}$$

respectively.

Let $s_{archaic}{}' = \frac{-ln\ (0.975)}{(1-\alpha)T}$ and $s_{AMH}{}' = \frac{-\ln(0.975)}{\alpha T}$. For two adjacent introgressed segments, if the gap between them is smaller than $s_{AMH}{}'$ and they matched to the same ancestry, we merged them and performed the matching algorithm to the newly merged segment. If the ancestry of the newly merged segment identical to that of the previous two segments, we will keep it, or we will restore the separation.

After the connection, if segments smaller than $s_{archaic}{}'$ this segment will be filtered out.

2.5 Parameters Estimation

This parameter estimation step estimated the introgression time T, introgression proportion α and updated several parameters in the HMM emission probability matrix. Unlike the classic Baum-Welch Algorithm estimating the elements in the transition probability matrix and emission probability matrix directly, we performed a modified EM Algorithm to estimate the demographic parameter underlining these two matrixes.

Here we focused on the segments matched to the leaf node in the matching model and only those segments will be considered in the following analysis.

The transition probability matrix is determined by introgression time T and the

introgression proportion α. These two parameters are easily to estimate.

$$A = \frac{\sum_{i=1}^{n_{seg}} s_i}{L * n_{hap}}, \quad (15)$$

$$T = \frac{n_{seg}}{(1-\alpha)\sum_{i=1}^{n_{seg}} s_i}, \quad (16)$$

where n_{seg} is the number of introgressed segments; s_i stands for the segment length of the *i*th archaic segments; n_{hap} is the number of haplotypes and L is the total length of the genome.

The emission matrix probability will be updated by calculating the proportion of those observed states in the two hidden states. There are eight probabilities in the emission matrix (Supplementary Table 2.2) and we will not update all of them. The emission probability of Observed State 4 condition on the two hidden states and probability of Observed State 3 condition on the archaic state will not be updated in the parameter estimation procedure. The Observed State 4 takes most of our genome, and provides little information about the introgression information. We will not give a higher weight for this observation state. In addition, we do not update the probability of Observed State 3 condition on the archaic state. That is because the fluctuation of this probability will increase the false positive rate of our method. We fixed this probability to a smaller value to filter the false positive segments.

After updating of the parameters, we repeated the seeking algorithm and the connection procedure. Until the absolute value of the difference between the updated T and previous T less than 0.01, we finished the parameter estimation iteration.

2.6 Introgression History Inference Method

In this section, we described a method to infer the history of archaic introgression based on the length distribution of introgressed segments. In our previous studies [10-12], we proposed the General Discrete Admixture Model to describe the complex admixture scenarios and developed a method, *MultiWaver*, to explore multiple-wave admixture histories. Here, we modified the General Discrete Admixture Model in *MultiWaver* to

infer multiple-wave introgression events.

In the General Discrete Admixture Model, we considered an admixed population with K ancestral populations and n-wave discrete admixture events (Supplementary Figure 2.4). Each wave (the i_{th} wave) of admixture can be determined by three parameters: k_i, α_i, and t_i. Here k_i is the ancestral population of the i_{th} event, α_i is the admixture proportion of the i_{th} the event, and t_i is the admixture time of the i_{th} event. The general discrete admixture model is determined by the admixture order, $O = (k_0, k_1, \ldots, k_n)$, the admixture proportion $\{\alpha_i\}_{0 \le i \le n}$, and the admixture time $\{t_i\}_{0 \le i \le n+1}$. We used a likelihood ratio test (LRT) [22] to select a proper admixture model, and implemented an expectation-maximization (EM) algorithm to estimate the corresponding parameters. For details, please refer to our previous method [10-12].

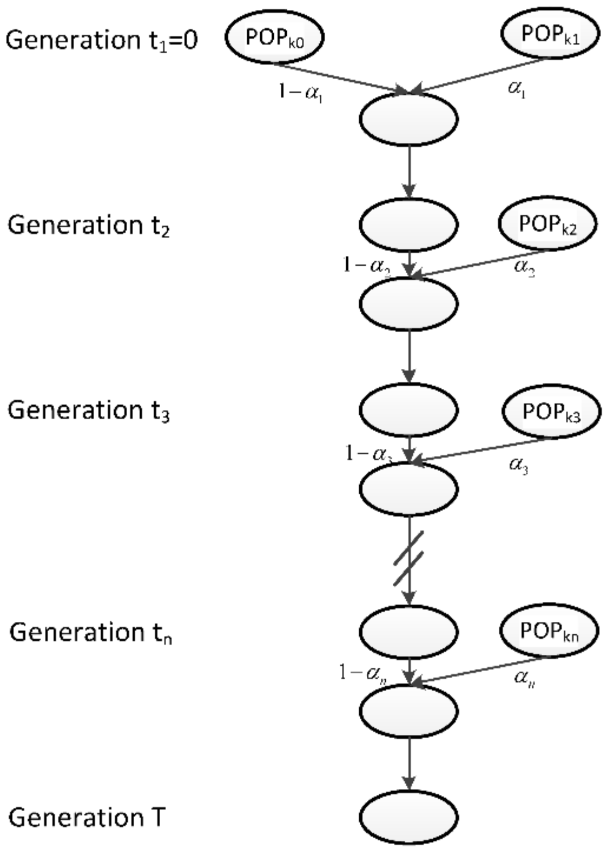

Supplementary Figure 2.4 The General Discrete Admixture Model. This figure described the basic model of *MultiWaver*. We considered n-wave admixture events and the admixture proportion is α_i for the i_{th} wave. The admixture time is t_i for the i_{th} wave. [10-12]

In this study, we modified the original General Discrete Admixture Model (Supplementary Figure 2.4) to infer the introgression history, by assuming the number of admixture waves from the modern human population is one, and the number of admixture waves from archaic populations is n. We supposed that the modern human populations have one or several pulses of introgressions from archaic hominins. Then archaic introgression model (Supplementary Figure 2.5) can be regarded as a special case of the General Discrete Admixture Model.

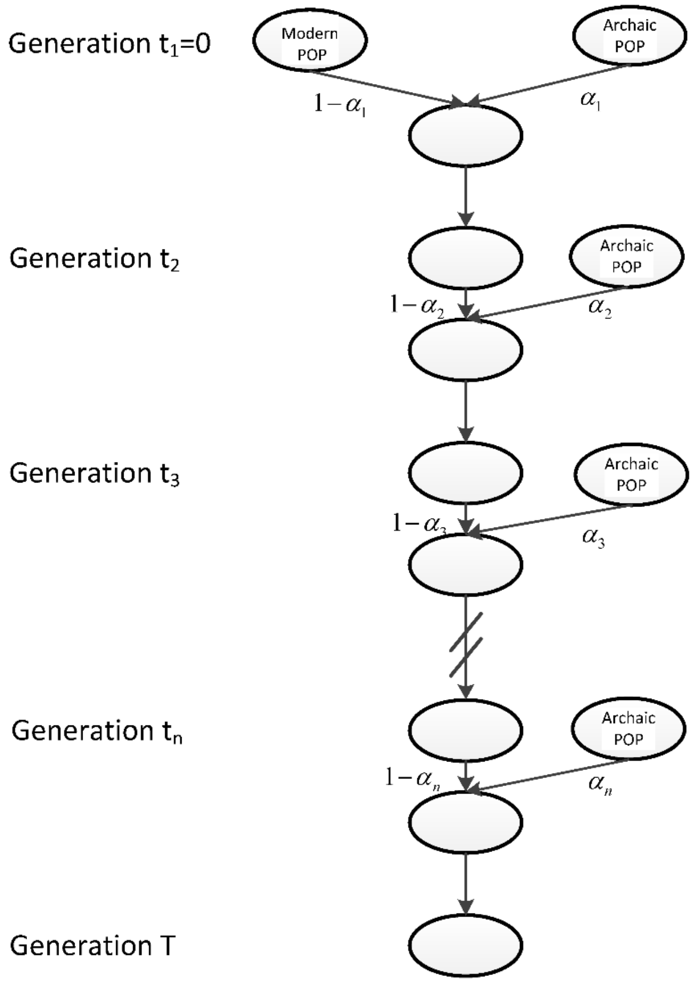

Supplementary Figure 2.5 The Archaic Introgression Model. This figure illustrated the archaic introgression model to describe the introgression history. In this model, we assumed the modern human population contributed to the admixed population only once as the founder. Then *n* waves of admixture pluses derived from archaic hominins introgressed into the modern human founder.

Thus, the idea of the *MultiWaver* method can be applied to this special problem. Here, we infer admixture history under the archaic introgression model. The detailed procedures are as follows,

Step 1: Estimate the total archaic introgression proportion. We divide the total length of inferred archaic segments to the total length of human genomes to get the estimation of introgression proportion.

Step 2: Determine the number of archaic introgression events. We assume the number of admixture waves contributed from the modern human population is one. We only need to determine the number of introgression events from the archaic populations. As we know, the length distribution of archaic segments is a mixed exponential distribution and this distribution can be treated as a summation of the different exponential distribution[11,12]. We use an expectation-maximization (EM) algorithm to separate the mixed exponential distribution to the summation of different exponential distributions and use a likelihood ratio test (LRT) to determine the number of exponential distributions to be spitted. Each exponential distribution stands for one introgression event. This procedure is the same as that of *MultiWaver* method [12].

Step 3: After the separation of the mixed distribution to several exponential distributions, we can estimate the introgression time (t_i) and proportion (α_i) of each event. This procedure is the same as that of *MultiWaver* method [12].

The difference between *MultiWaver* and this method is that one of the ancestral populations (modern human population) in this method only contributes to the final admixed population one time as one of the founders. This kind of model is simpler than the model in the *MultiWaver* method. We can treat this model as a special case of the *MultiWaver* model and use the information of archaic segment length to determine the archaic introgression model and estimate the parameters under this model.

2.7 Phasing, Sequencing Error, and Modern Human Contamination

Haplotype information is required for *ArchaicSeeker 2.0*, so the phasing error can potentially introduce uncertainties. However, the phasing error may not be serious for the non-AMH makers owing to the high linkage among those markers. It is relatively reliable to phase the introgressed region or at least for the non-AMH AIMs, which are the most informative markers in our method.

Sequencing error and modern human contamination are also two inevitable problems for the analysis of archaic or ancient genomes. A sequencing error in the archaic genome likely leave a derived allele in the genomic data. The allele at the same genomic position in African reference and test genome are still ancestral alleles. This will cause a State 4 observation but it is the least informative. In our model, we use the information of at least two high coverage archaic genomes as the references. The sequencing error and the contamination are less likely to be in the same position on all the archaic references. Therefore, it would not influence our results.

As we know, the recombination rate is not evenly distributed on the genome[23,24]. Local recombination hotspots do affect the results of history inference. It is hard to measure the recombination rate and mutation rate at an extremely fine scale. History inference methods with nucleotide difference information will also face the uneven distribution of local mutation rates. Here, we introduced the recombination map option to reduce this influence as much as possible.

2.8 Comparison of Available Archaic Inference Methods

Since most of the available archaic inference methods are hard to apply, we didn't compare them by applying them to the simulation dataset. We briefly compared these methods on the software functions and properties.

Supplementary Table 2.3 Archaic Inference Methods

	S* series methods	***Sprime***	***ArchIE***	***CRF***	***Skov's method***	***Jacobs's method***	***ArchaicSeeker1.0***	***ArchaicSeeker2.0***
History Inference	Unable*	Enable to infer the number of introgression	Unable*	Unable	Introgression time and proportion of one pulse admixture	Combined several methods to reconstruct the introgression history	Unable	Enable to infer multiple wave introgression history
Software Available	Not available	Available	Available	Not available	Available	Not available	Available	Available
Archaic Reference	Not required	Not required	Not required	Required	Not required	Required	Required	Required
Haplotype Information	Required	Required	Required	Required	Not required	Required	Required	Required
Unknown Archaic Detection	Enable	Enable	Enable	Unable	Enable	Enable	Unable	Enable

	S* series methods	***Sprime***	***ArchIE***	***CRF***	***Skov's method***	***Jacobs's method***	***ArchaicSeeker1.0***	***ArchaicSeeker2.0***
Prior Simulation Null Model	Required	Not required	Required	Not required	Not required	Required	Not required	Not required
Ancestry Matching	Unable**	Unable**	Unable**	Enable	Unable	Enable	Enable	Enable
Output Information	Introgressed region in population	Introgressed region in population	Introgressed region in population	Introgressed region in haplotype	Introgressed region in individual	Introgressed region in haplotype	Introgressed region in haplotype	Introgressed region in haplotype

* Required additional steps to fit the introgression model with massive simulations.

** Required additional step to match a proper archaic ancestry.

3 Supplementary Note 3: Simulations

To evaluate the performance of our methods, we generated massive simulation genomic data. The scenarios of these simulations were modified from the demographic history. For each series of the simulations, we only changed one or two parameters to evaluate the effect of these parameters on our method.

Here, we use *ms* [25] to simulate the genomic data. To extract the real introgressed segments, we developed a script called *SimAncestry* to analyze the tree topology of *ms* output.

The script *SimAncestry* is only a simple script to analyze the tree structure outputted by the software *ms*. It cannot apply to the real data as it was not designed to work with SNP data. The algorithm used in *SimAncestry* is as follows. For each non-African node, we make a transversal up to the root. If this node coalesces to archaic lineage before any African lineages, we say this node is introgressed. This script is used only to obtain the ground truth for introgressed sequences.

The simulation parameters we used were adapted from a study by Vernot and Akey[20]. For each simulation parameter set, we repeated 100 times. For each run, we simulated 200 African haplotypes, 200 Non-African haplotypes, 2 haplotypes of Altai Denisovan, 2 haplotypes of Altai Neanderthal, and several introgressed lineage haplotypes, which used to find introgressed segments. We simulated 10 Mb genomes for each run. The mutation rate was set to 1.25×10^{-8} per generation per bp. The recombination rate was set to 1×10^{-8}. The generation time was set to 25 years per generation[26]. In the *ms* command line, N_0 was set to 10000.

The basic demographic parameters set as follow (Supplementary Figure 3.1),

$T_{AMH-Archaic}$ = 657.5 kya #Divergence time between AMH and archaic humans

$T_{Den-Nean}$ = 420 kya #Divergence time between Denisovan and Neanderthal

$T_{Afr-NonAfr}$ = 100 kya #Divergence time between African and Non-African

$T_{AltaiDenDeath}$ = 80 kya #Time of Altai Denisovan death

$T_{AltaiNeanDeath}$ = 60 kya #Time of Altai Neanderthal death

$T_{AMHexp1}$ = 23 kya #Time of the first AMH population expansion

$T_{AMHexp2}$ = 5 kya #Time of the second AMH population expansion

$N_{Archaic}$ = 1500 #Effective population size of archaic humans

N_{AMH} = 3000 #Effective population size of AMH populations

N_{AMH1} = 5000 #Effective population size of AMH before the first time AMH population expansion

N_{Afr2} = 15000 #Effective population size of Africans after the first time AMH population expansion

$N_{NonAfr2}$ = 10000 #Effective population size of Non-African after the first time AMH population expansion

N_{Afr3} = 500000 # Effective population size of African after the second time AMH population expansion (now)

$N_{NonAfr3}$ = 1000000 # Effective population size of Non-African after the second time AMH population expansion (now)

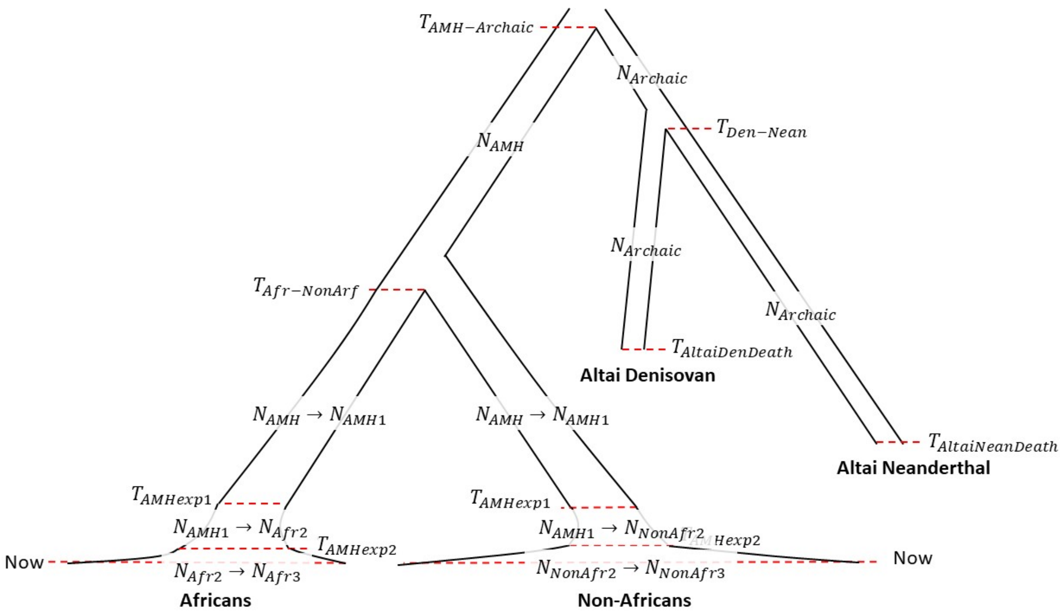

Supplementary Figure 3.1 The basic demographic scenario. This figure demonstrates the basic demographic model applied in our simulation analysis. In this scenario, we described the basic phylogenetic relationship among Africans, Non-Africans, Altai Denisovan, and Altai Neanderthal. The following diverse scenarios are modified based on this scenario. This detailed value of parameters shows in the text.

3.1 Introgressions from Single Archaic Lineage

In these series simulations, we modeled introgression only from one archaic hominin lineage. The introgressed lineage divergence from Altai Denisovan, Altai Neanderthal, or the ancestor of Denisovan and Neanderthal (unknown archaic hominin). The divergence time to the archaic lineage, the introgression time, and the introgression proportion changed in gradient to evaluate the influences of these parameters.

We also simulated the two-wave introgression from one archaic lineage, where the parameter representing the introgression time and that representing introgression proportion are both changed in a step-wise manner.

3.1.1 Scenarios with Diverse Divergence Time

In these simulation scenarios, we set the divergence time to the archaic lineage as different values. For scenarios of introgression from the Denisovan lineage (Supplementary Figure 3.2), the divergence time to Altai Denisovan, $T_{DenSplit}$ was set to nine different values, from 267 to 403 kya with an increasing step 17 kya. For scenarios of introgression from the Neanderthal lineage (Supplementary Figure 3.3), the divergence time to Altai Neanderthal, $T_{NeanSplit}$ was set to nine different values, from 75 to 115 kya with an increasing step 5 kya. The introgression timeT_{Intro} was set to 50 kya and the introgression proportion α was set to 2%.

Totally 1800 times simulations were performed under these series scenarios.

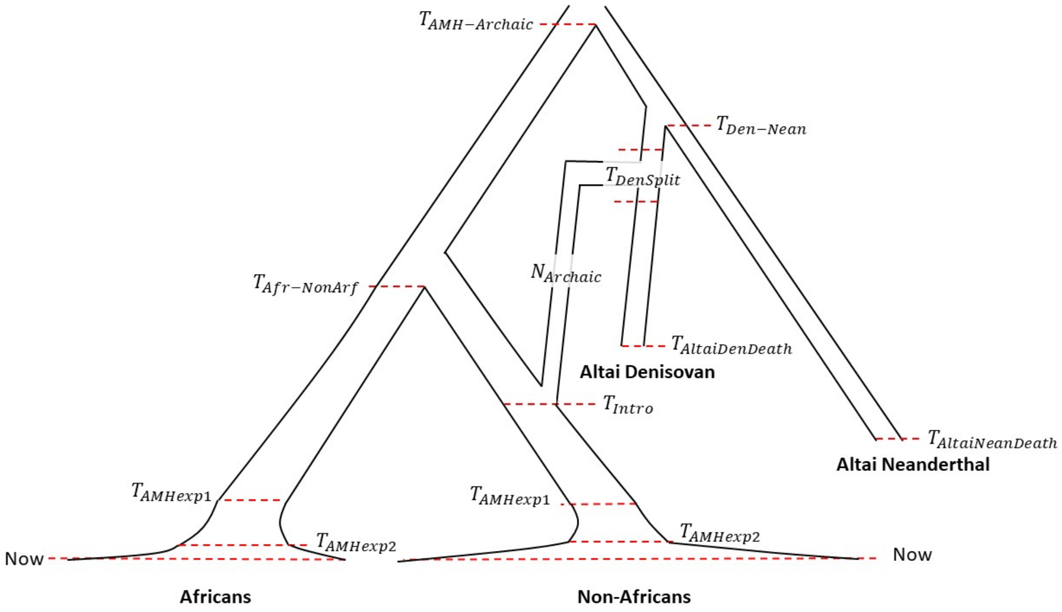

Supplementary Figure 3.2 Introgression from Denisovan lineage with different divergence times. In these scenarios, we modeled an introgression event from Denisovan lineage to the Non-Africans. The divergence time to the Denisovan lineage, $T_{DenSplit}$ changed in gradient.

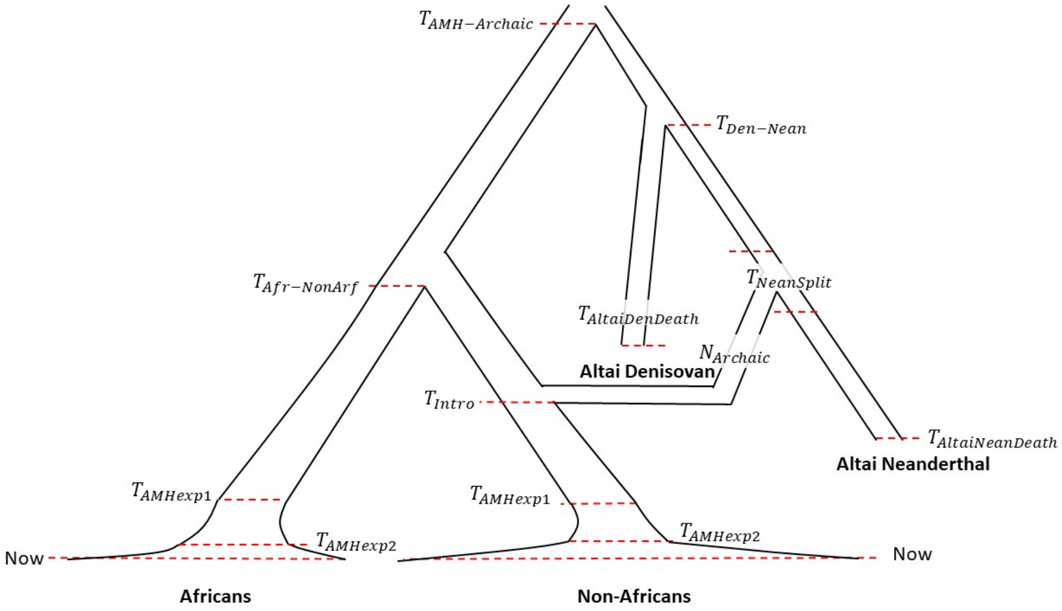

Supplementary Figure 3.3 Introgression from Neanderthal lineage with different divergence times. In these scenarios, we modeled an introgression event from Neanderthal lineage to the Non-Africans. The divergence time to the Neanderthal lineage $T_{NeanSplit}$ changed in gradient.

An example of *ms* command line of the Denisovan lineage introgression is that ms 405 1 -t 5000 -r 4000 10000000 -I 7 200 200 1 1 1 1 1 0 -n 1 50 -n 2 100 -n 3 1e-

10 -n 4 1e-10 -n 5 1e-10 -n 6 1e-10 -n 7 1e-10 -eg 1e-10 1 175.328 -eg 2e-10 2 230.259 -eg 0.005 1 15.2585 -eg 0.005001 2 9.62704 -eg 0.023 1 1.65852 -eg 0.023001 2 1.65852 -es 0.05 2 0.98 -ej 0.050001 8 7 -en 0.050002 7 0.15 -ej 0.08 4 3 -en 0.080001 3 0.15 -ej 0.1 2 1 -en 0.100001 1 0.3 -eg 0.100002 1 0.310828 -ej 0.06 6 5 -en 0.060001 5 0.15 -ej 0.42 5 3 -ej 0.267 7 3 -ej 0.6575 3 1 -T

# "-ej 0.267 7 3" This parameter used to control the divergence time to Denisovan, 0.267 correspond to 267kya divergence from the Altai Denisovan

An example of *ms* command line of the Neanderthal-like introgression is that

ms 405 1 -t 5000 -r 4000 10000000 -I 7 200 200 1 1 1 1 1 0 -n 1 50 -n 2 100 -n 3 1e-10 -n 4 1e-10 -n 5 1e-10 -n 6 1e-10 -n 7 1e-10 -eg 1e-10 1 175.328 -eg 2e-10 2 230.259 -eg 0.005 1 15.2585 -eg 0.005001 2 9.62704 -eg 0.023 1 1.65852 -eg 0.023001 2 1.65852 -es 0.05 2 0.98 -ej 0.050001 8 7 -en 0.050002 7 0.15 -ej 0.08 4 3 -en 0.080001 3 0.15 -ej 0.1 2 1 -en 0.100001 1 0.3 -eg 0.100002 1 0.310828 -ej 0.06 6 5 -en 0.060001 5 0.15 -ej 0.42 5 3 -ej 0.075 7 5 -ej 0.6575 3 1 -T

# "-ej 0.075 7 5" This parameter used to control the divergence time to Neanderthal, 0.075 correspond to 75kya divergence from the Altai Neanderthal

In these simulations, individual (haplotype) 1-200 (pop 1) stand for the Africans; individual 201-400 (pop 2) stand for the Non-Africans; individual 401-402 (pop 3-4) stand for the Altai Denisovan; individual 403-404 (pop 5-6) stand for the Altai Neanderthal; individual 405 (pop 7) stands for the introgressed lineage used to identify the introgressed segment. Noted, individual 405 will only be used to identify the introgressed segments in the script *SimAncestry* and it will not be used in the *ArchaicSeeker 2.0* analysis.

3.1.2 Scenarios with Diverse Introgression Time

In these simulation scenarios, we set the introgression time T_{Intro} as nine different values, from 18 to 82 kya with an increasing step 8 kya. $T_{DenSplit}$ was set to 335 kya and $T_{NeanSplit}$ was set to 95 kya (Supplementary Figure 3.4-3.5). The introgression proportion α was set to 2%.

Totally 1800 times simulations were performed under these series scenarios.

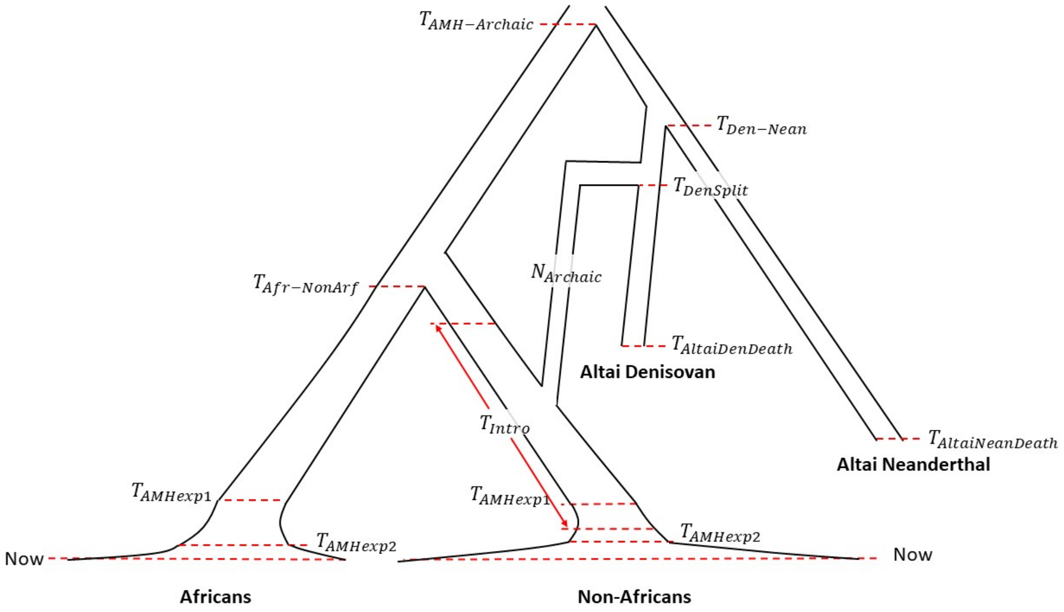

Supplementary Figure 3.4 Introgression from Denisovan lineage with different introgression time. In these scenarios, we modeled an introgression event from Denisovan lineage to the Non-Africans. The introgression time T_{Intro} changed in gradient.

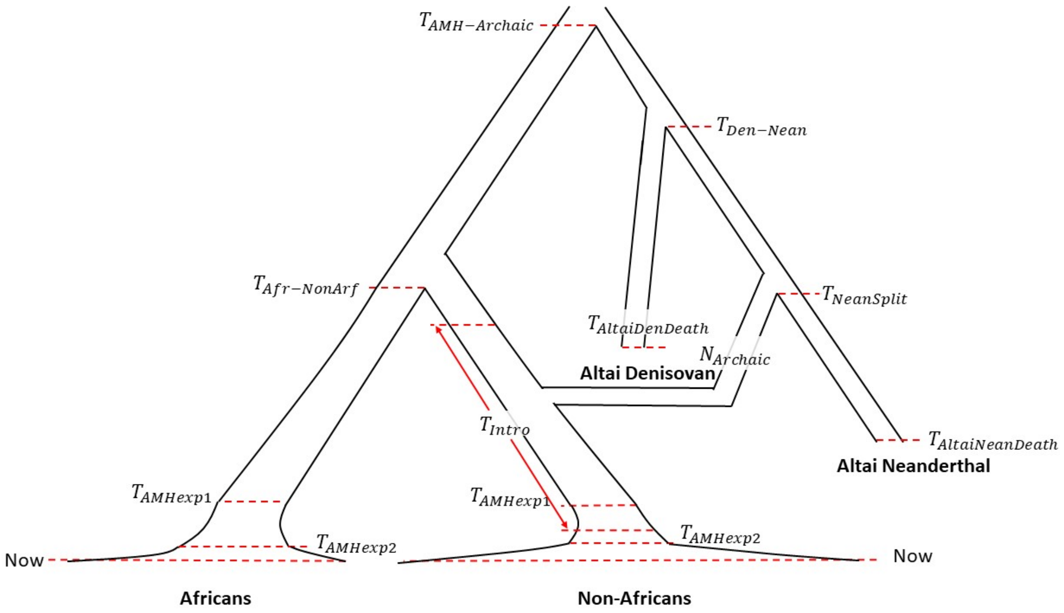

Supplementary Figure 3.5 Introgression from Neanderthal lineage with different introgression time. In these scenarios, we modeled an introgression event from Neanderthal lineage to the Non-Africans. The introgression time T_{Intro} changed in gradient.

An example of *ms* command line of the Denisovan lineage introgression is that ms 405 1 -t 5000 -r 4000 10000000 -I 7 200 200 1 1 1 1 1 0 -n 1 50 -n 2 100 -n 3 1e-

10 -n 4 1e-10 -n 5 1e-10 -n 6 1e-10 -n 7 1e-10 -eg 1e-10 1 175.328 -eg 2e-10 2 230.259 -eg 0.005 1 15.2585 -eg 0.005001 2 9.62704 -eg 0.023 1 1.65852 -eg 0.023001 2 1.65852 -es 0.018 2 0.98 -ej 0.018001 8 7 -en 0.018002 7 0.15 -ej 0.08 4 3 -en 0.080001 3 0.15 -ej 0.1 2 1 -en 0.100001 1 0.3 -eg 0.100002 1 0.310828 -ej 0.06 6 5 -en 0.060001 5 0.15 -ej 0.42 5 3 -ej 0.335 7 3 -ej 0.6575 3 1 -T

# "-es 0.018 2 0.98 -ej 0.018001 8 7 -en 0.018002 7 0.15" These parameters related to the introgression time. 0.018 corresponds to 18 kya introgression time. A slight increase for the second and third parameters is to avoid errors.

An example of *ms* command line of the Neanderthal lineage introgression is that

ms 405 1 -t 5000 -r 4000 10000000 -I 7 200 200 1 1 1 1 1 0 -n 1 50 -n 2 100 -n 3 1e-10 -n 4 1e-10 -n 5 1e-10 -n 6 1e-10 -n 7 1e-10 -eg 1e-10 1 175.328 -eg 2e-10 2 230.259 -eg 0.005 1 15.2585 -eg 0.005001 2 9.62704 -eg 0.023 1 1.65852 -eg 0.023001 2 1.65852 -es 0.018 2 0.98 -ej 0.018001 8 7 -en 0.018002 7 0.15 -ej 0.08 4 3 -en 0.080001 3 0.15 -ej 0.1 2 1 -en 0.100001 1 0.3 -eg 0.100002 1 0.310828 -ej 0.06 6 5 -en 0.060001 5 0.15 -ej 0.42 5 3 -ej 0.095 7 5 -ej 0.6575 3 1 -T

# "-es 0.018 2 0.98 -ej 0.018001 8 7 -en 0.018002 7 0.15" These parameters related to the introgression time. 0.018 corresponds to 18 kya introgression time.

The individuals' annotation is the same as Supplementary Note 3.1.1.

3.1.3 Scenarios with Diverse Introgression Proportion

In these simulation scenarios, we set the introgression proportion α as 10 different values, from 0.4% to 4% with an increasing step 0.4%. $T_{DenSplit}$ was set to 335 kya and $T_{NeanSplit}$ was set to 95 kya. The introgression time T_{Intro} was set to 50 kya (Supplementary Figure 3.6-3.7).

Totally 2000 times simulations were performed under these series scenarios.

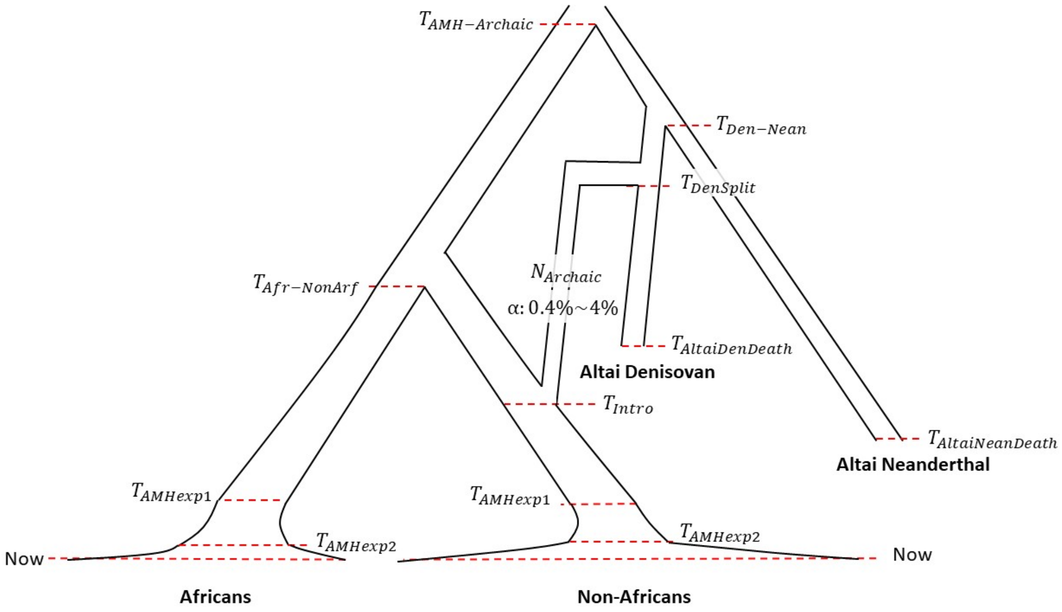

Supplementary Figure 3.6 Introgression from Denisovan lineage with diverse introgression proportion. In these scenarios, we modeled an introgression event from Denisovan lineage to the Non-Africans. The introgression proportion α changed in gradient.

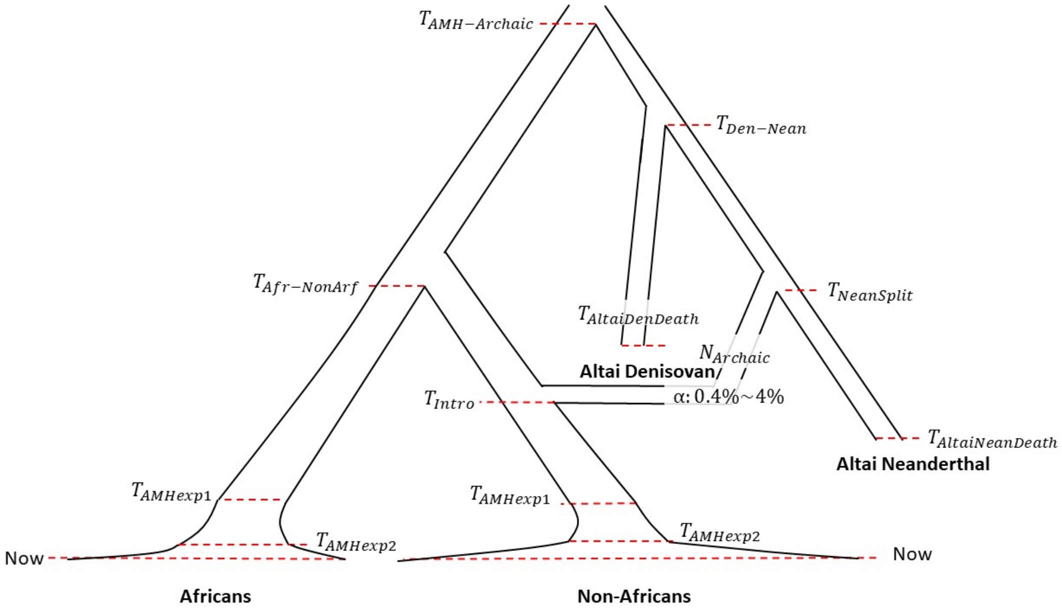

Supplementary Figure 3.7 Introgression from Neanderthal lineage with diverse introgression proportion. In these scenarios, we modeled an introgression event from Neanderthal lineage to the Non-Africans. The introgression proportion α changed in gradient.

An example of *ms* command line of the Denisovan lineage introgression is that ms 405 1 -t 5000 -r 4000 10000000 -I 7 200 200 1 1 1 1 1 0 -n 1 50 -n 2 100 -n 3 1e-10 -n 4 1e-10 -n 5 1e-10 -n 6 1e-10 -n 7 1e-10 -eg 1e-10 1 175.328 -eg 2e-10 2

230.259 -eg 0.005 1 15.2585 -eg 0.005001 2 9.62704 -eg 0.023 1 1.65852 -eg 0.023001 2 1.65852 -es 0.05 2 0.996 -ej 0.050001 8 7 -en 0.050002 7 0.15 -ej 0.08 4 3 -en 0.080001 3 0.15 -ej 0.1 2 1 -en 0.100001 1 0.3 -eg 0.100002 1 0.310828 -ej 0.06 6 5 -en 0.060001 5 0.15 -ej 0.42 5 3 -ej 0.335 7 3 -ej 0.6575 3 1 -T

# "-es 0.05 2 0.996" This parameter controls the introgression proportion. 0.996 corresponds to 0.004 introgression proportion.

An example of *ms* command line of the Neanderthal lineage introgression is that ms 405 1 -t 5000 -r 4000 10000000 -I 7 200 200 1 1 1 1 1 0 -n 1 50 -n 2 100 -n 3 1e-10 -n 4 1e-10 -n 5 1e-10 -n 6 1e-10 -n 7 1e-10 -eg 1e-10 1 175.328 -eg 2e-10 2 230.259 -eg 0.005 1 15.2585 -eg 0.005001 2 9.62704 -eg 0.023 1 1.65852 -eg 0.023001 2 1.65852 -es 0.05 2 0.996 -ej 0.050001 8 7 -en 0.050002 7 0.15 -ej 0.08 4 3 -en 0.080001 3 0.15 -ej 0.1 2 1 -en 0.100001 1 0.3 -eg 0.100002 1 0.310828 -ej 0.06 6 5 -en 0.060001 5 0.15 -ej 0.42 5 3 -ej 0.095 7 5 -ej 0.6575 3 1 -T

# "-es 0.05 2 0.996" This parameter control the introgression proportion. 0.996 corresponds to 0.004 introgression proportion.

The individuals' annotation is the same as Supplementary Note 3.1.1.

3.1.4 Scenarios of Two-Waves Introgression from Single Archaic Lineage

These simulation scenarios are a little bit different from previous ones. In these simulations, we allowed two-wave introgression from one archaic lineage. The introgression time of those two waves T_{Intro1} and T_{Intro2} were set for four possible different values, from 26 to 74 kya with an increasing step 16 kya and we set T_{Intro1} smaller than T_{Intro2}. For each pair of introgression times, we changed the introgression proportion of each waves, α_1 and α_2. We set four possible values for introgression proportion, from 0.4% to 1.6% with an increasing step 0.4%. We also required $\alpha_1 + \alpha_2 = 2\%$, so there were only four possible introgression proportion pairs. $T_{DenSplit}$ was set to 335 kya and $T_{NeanSplit}$ was set to 95 kya (Supplementary Figure 3.8-3.9).

Totally 4800 times simulations were performed under these series scenarios.

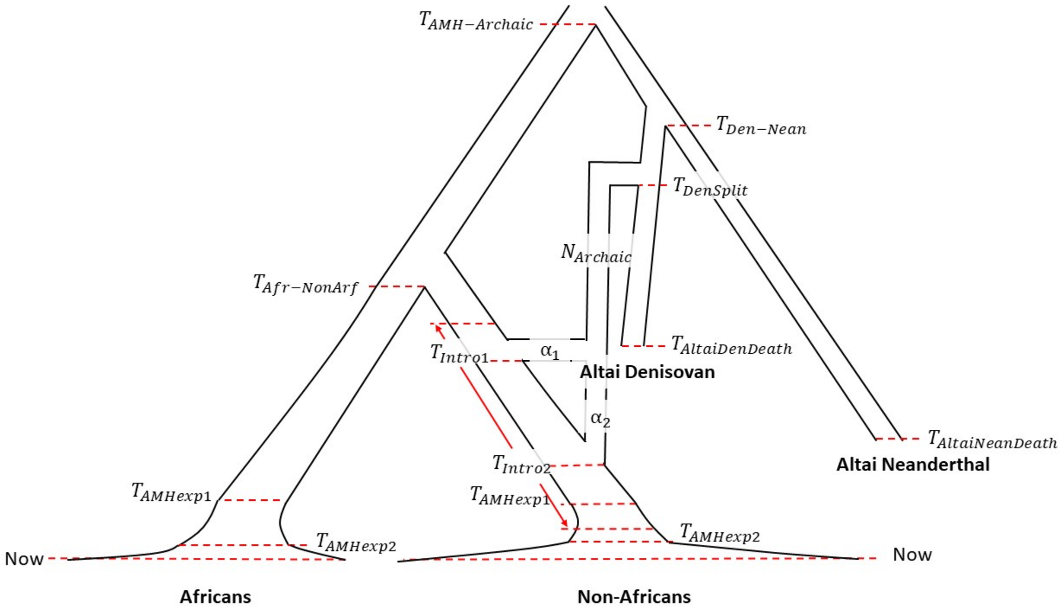

Supplementary Figure 3.8 Two waves introgression from Denisovan with diverse introgression time and proportion. In these scenarios, we modeled two introgression events from Denisovan lineage to the Non-Africans. The introgression time T_{Intro1} and T_{Intro2} and proportion α_1 and α_2 were set to different values.

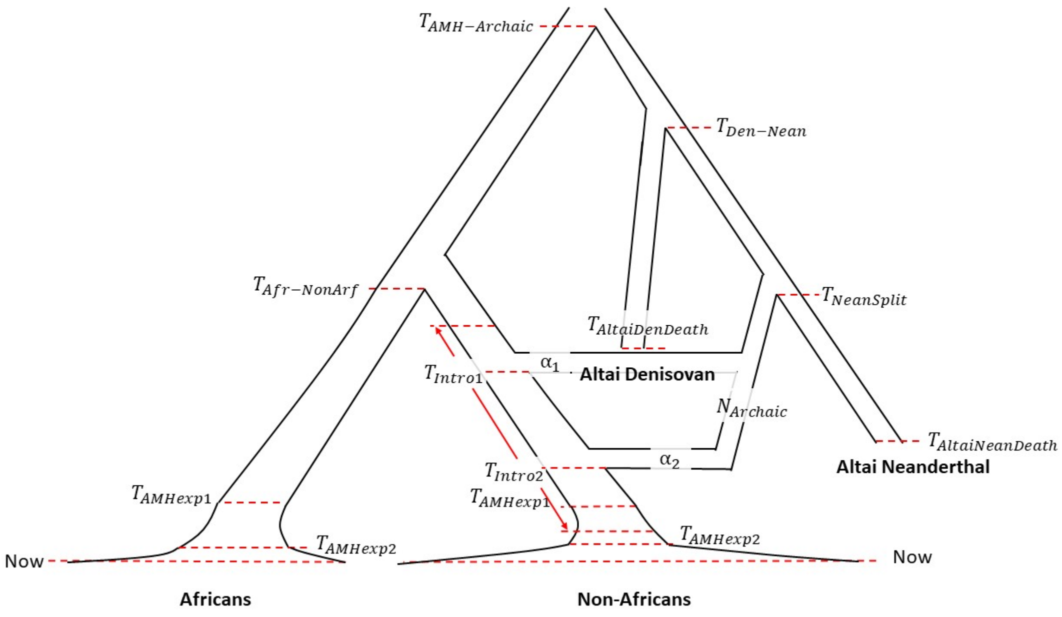

Supplementary Figure 3.9 Two waves introgression from Neanderthal lineage with diverse introgression time and proportion. In these scenarios, we modeled two introgression events from Neanderthal lineage to the Non-Africans. The introgression time T_{Intro1} and T_{Intro2} and proportion α_1 and α_2 were set to different values.

An example of *ms* command line of the Denisovan lineage introgression is that

ms 406 1 -t 5000 -r 4000 10000000 -I 8 200 200 1 1 1 1 1 1 0 -n 1 50 -n 2 100 -n 3 1e-10 -n 4 1e-10 -n 5 1e-10 -n 6 1e-10 -n 7 1e-10 -n 8 1e-10 -eg 1e-10 1 175.328 -eg 2e-10 2 230.259 -eg 0.005 1 15.2585 -eg 0.005001 2 9.62704 -eg 0.023 1 1.65852 -eg 0.023001 2 1.65852 -es 0.026 2 0.996 -ej 0.026001 9 7 -en 0.026002 7 0.15 -es 0.042 2 0.984 -ej 0.042001 10 8 -en 0.042002 8 0.15 -ej 0.08 4 3 -en 0.080001 3 0.15 -ej 0.1 2 1 -en 0.100001 1 0.3 -eg 0.100002 1 0.310828 -ej 0.06 6 5 -en 0.060001 5 0.15 -ej 0.42 5 3 -ej 0.335 7 3 -ej 0.335001 8 3 -ej 0.6575 3 1 -T

# "-es 0.042 2 0.984 -ej 0.042001 10 8 -en 0.042002 8 0.15" These parameters control the first wave of introgression. 0.042 corresponds to 42 kya introgression time and 0.984 corresponds to 1.6% introgression proportion of this wave.

# "-es 0.026 2 0.996 -ej 0.026001 9 7 -en 0.026002 7 0.15" These parameters control the second wave of introgression. 0.026 corresponds to 26 kya introgression time and 0.996 corresponds to 0.4% introgression proportion of this wave.

An example of *ms* command line of the Neanderthal lineage introgression is that

ms 406 1 -t 5000 -r 4000 10000000 -I 8 200 200 1 1 1 1 1 1 0 -n 1 50 -n 2 100 -n 3 1e-10 -n 4 1e-10 -n 5 1e-10 -n 6 1e-10 -n 7 1e-10 -n 8 1e-10 -eg 1e-10 1 175.328 -eg 2e-10 2 230.259 -eg 0.005 1 15.2585 -eg 0.005001 2 9.62704 -eg 0.023 1 1.65852 -eg 0.023001 2 1.65852 -es 0.026 2 0.996 -ej 0.026001 9 7 -en 0.026002 7 0.15 -es 0.042 2 0.984 -ej 0.042001 10 8 -en 0.042002 8 0.15 -ej 0.08 4 3 -en 0.080001 3 0.15 -ej 0.1 2 1 -en 0.100001 1 0.3 -eg 0.100002 1 0.310828 -ej 0.06 6 5 -en 0.060001 5 0.15 -ej 0.42 5 3 -ej 0.335 7 5 -ej 0.335001 8 5 -ej 0.6575 3 1 -T

# "-es 0.042 2 0.984 -ej 0.042001 10 8 -en 0.042002 8 0.15" These parameters control the first wave of introgression. 0.042 corresponds to 42 kya introgression time and 0.984 corresponds to 1.6% introgression proportion of this wave.

# "-es 0.026 2 0.996 -ej 0.026001 9 7 -en 0.026002 7 0.15" These parameters control the second wave of introgression. 0.026 corresponds to 26 kya introgression time and 0.996 corresponds to 0.4% introgression proportion of this wave.

In these simulations, individual (haplotype) 1-200 (pop 1) stand for the Africans; individual 201-400 (pop 2) stand for the test Non-Africans; individual 401-402 (pop 3-

4) stand for the Altai Denisovan; individual 403-404 (pop 5-6) stand for the Altai Neanderthal; individual 405 (pop 7) stands for the introgressed lineage of the second wave; individual 406 (pop 8) stands for the introgressed lineage of the first wave. Individual 405 and 406 are only used to identify the introgressed segments of each wave in the script *SimAncestry* and they will not be used in the *ArchaicSeeker 2.0* analysis.

3.2 Introgressions from both Denisovan and Neanderthal

In these series simulations, we modeled introgression from both Denisovan and Neanderthal. Two introgressed lineages divergence from the two sequenced archaic hominins, respectively. The introgression time and the introgression proportion were change in gradient to evaluate the influence of these parameters.

We allowed one wave introgression from each archaic lineage. The introgression time of those two waves, $T_{IntroDen}$ and $T_{IntroNean}$ were set to three possible values, 30, 50 and 70 kya. For each pair of introgression time, we changed the introgression proportion of each wave, α_{Den} and α_{Nean}. We set four possible values for introgression proportion, from 0.4% to 1.6% with an increasing step 0.4%. We also required $\alpha_{Den} + \alpha_{Nean} = 2\%$, so there are only 4 possible introgression proportion pairs. $T_{DenSplit}$ was set to 335 kya and $T_{NeanSplit}$ was set to 95 kya (Supplementary Figure 3.10).

Totally 3600 times simulations were performed under these series scenarios.

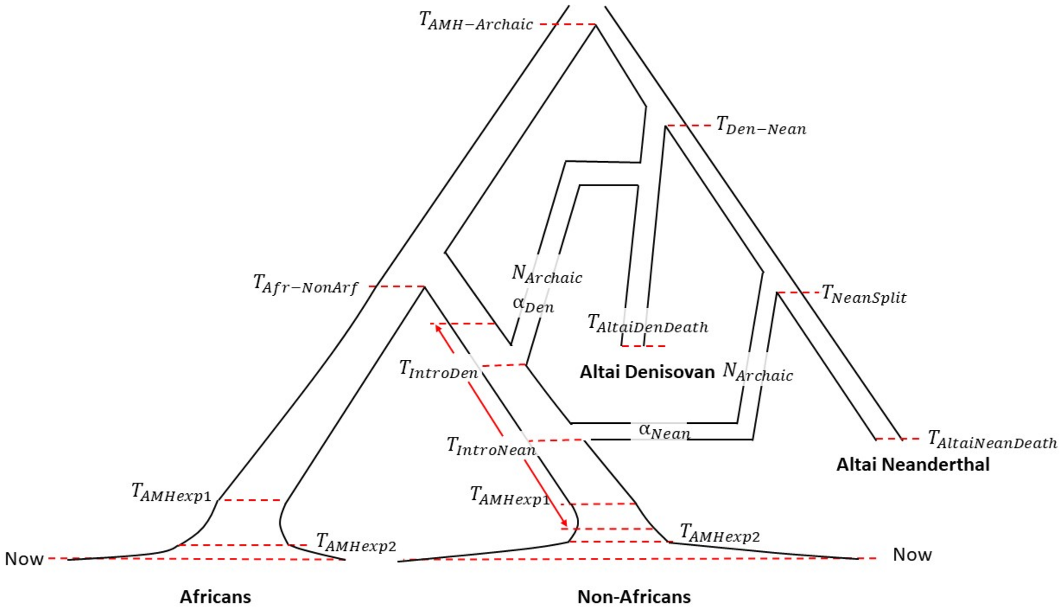

Supplementary Figure 3.10 Introgression from two archaic lineages with diverse introgression time and proportion. In these scenarios, we modeled introgression events from both Denisovan lineage and Neanderthal lineage to the Non-Africans. The introgression time from Denisovan lineage is $T_{IntroDen}$ and that from Neanderthal is $T_{IntroNean}$. The introgression proportion of Denisovan lineage is α_{Den} and that of Neanderthal is α_{Nean}. The introgression time and introgression proportion were set to different values.

An example of *ms* command line is that

ms 406 1 -t 5000 -r 4000 10000000 -I 8 200 200 1 1 1 1 1 1 0 -n 1 50 -n 2 100 -n 3 1e-10 -n 4 1e-10 -n 5 1e-10 -n 6 1e-10 -n 7 1e-10 -n 8 1e-10 -eg 1e-10 1 175.328 -eg 2e-10 2 230.259 -eg 0.005 1 15.2585 -eg 0.005001 2 9.62704 -eg 0.023 1 1.65852 -eg 0.023001 2 1.65852 -es 0.03 2 0.984 -ej 0.030001 9 7 -en 0.030002 7 0.15 -es 0.030003 2 0.996 -ej 0.030004 10 8 -en 0.030005 8 0.15 -ej 0.08 4 3 -en 0.080001 3 0.15 -ej 0.1 2 1 -en 0.100001 1 0.3 -eg 0.100002 1 0.310828 -ej 0.06 6 5 -en 0.060001 5 0.15 -ej 0.42 5 3 -ej 0.095 7 5 -ej 0.335 8 3 -ej 0.6575 3 1 -T

# "-es 0.03 2 0.984 -ej 0.030001 9 7 -en 0.030002 7 0.15" These parameters control the introgression events of Neanderthal. 0.03 corresponds the introgression time is 30 kya; 0.984 means the introgression proportion is 1.6%.

# "-es 0.030003 2 0.996 -ej 0.030004 10 8 -en 0.030005 8 0.15 -ej 0.08 4 3" These parameters control the introgression events of Denisovan. 0.030003 corresponds the

introgression time is 30 kya; 0.996 means the introgression proportion is 0.4%.

In these simulations, individual (haplotype) 1-200 (pop 1) stands for the Africans; individual 201-400 (pop 2) stand for the test Non-Africans; individual 401-402 (pop 3-4) stand for the Altai Denisovan; individual 403-404 (pop 5-6) stand for the Altai Neanderthal; individual 405 (pop 7) stands for the Neanderthal introgressed lineage; individual 406 (pop 8) stands for the Denisovan introgressed lineage. Individual 405 and 406 are only used to identify the introgressed segments in the script *SimAncestry* and they will not be used in the *ArchaicSeeker 2.0* analysis.

3.3 Introgression from a Deep Divergent Archaic Lineage

To test the ability of our method to detect the deep divergent lineage archaic hominins, which divergence from the ancestor of Denisovan and Neanderthal, we performed these series simulations. In these simulation scenarios, we changed the divergence time between the introgressed linage and the ancient archaic lineage, $T_{UnSplit}$. Four possible values were set for this parameter, 467.5, 515, 562 and 610 kya. The introgression time T_{intro} was set to 50 kya and the introgression proportion α set to 2.0% (Supplementary Figure 3.11).

Totally 400 simulations were performed under these series scenarios.

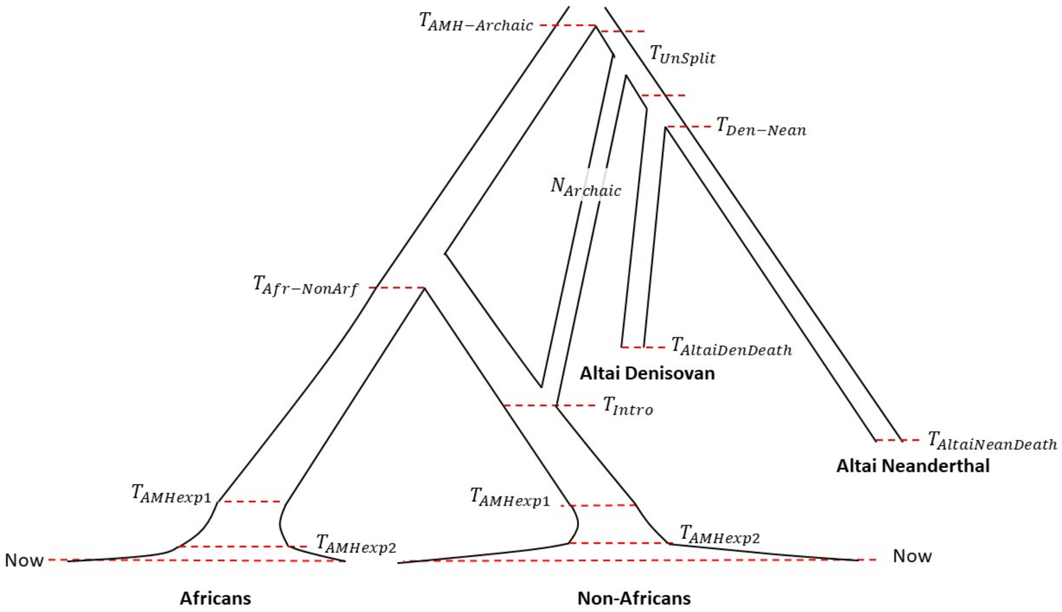

Supplementary Figure 3.11 Introgression from a deep divergent archaic lineage. In these

scenarios, we modeled an introgression event from a deep divergent archaic lineage to the Non-Africans. The divergence time to the archaic lineage $T_{UnSplit}$ changed in gradient.

An example of *ms* command line is as follows,

ms 405 1 -t 5000 -r 4000 10000000 -I 7 200 200 1 1 1 1 1 0 -n 1 50 -n 2 100 -n 3 1e-10 -n 4 1e-10 -n 5 1e-10 -n 6 1e-10 -n 7 1e-10 -eg 1e-10 1 175.328 -eg 2e-10 2 230.259 -eg 0.005 1 15.2585 -eg 0.005001 2 9.62704 -eg 0.023 1 1.65852 -eg 0.023001 2 1.65852 -es 0.05 2 0.98 -ej 0.050001 8 7 -en 0.050002 7 0.15 -ej 0.08 4 3 -en 0.080001 3 0.15 -ej 0.1 2 1 -en 0.100001 1 0.3 -eg 0.100002 1 0.310828 -ej 0.06 6 5 -en 0.060001 5 0.15 -ej 0.42 5 3 -ej 0.4675 7 3 -ej 0.6575 3 1 -T

# "-ej 0.4675 7 3" this parameter controls the divergence time of introgressed lineage to archaic lineage. 0.4675 corresponds 467.5 kya.

The individuals' annotation is the same as Supplementary Note 3.1.1.

3.4 Summary Tables of Simulation Scenarios

Supplementary Table 3.1 Simulation parameters of introgression from one archaic lineage

Section	Archaic Lineage	Divergence Time (kya)	Divergence Time (gen)	Introgression Time (kya)	Introgression Time (gen)	Introgression Proportion
S3.1.1	Denisovan	267	10680	50	2000	0.02
S3.1.1	Denisovan	284	11360	50	2000	0.02
S3.1.1	Denisovan	301	12040	50	2000	0.02
S3.1.1	Denisovan	318	12720	50	2000	0.02
S3.1.1	Denisovan	335	13400	50	2000	0.02
S3.1.1	Denisovan	352	14080	50	2000	0.02
S3.1.1	Denisovan	369	14760	50	2000	0.02
S3.1.1	Denisovan	386	15440	50	2000	0.02
S3.1.1	Denisovan	403	16120	50	2000	0.02
S3.1.1	Neanderthal	75	3000	50	2000	0.02
S3.1.1	Neanderthal	80	3200	50	2000	0.02
S3.1.1	Neanderthal	85	3400	50	2000	0.02
S3.1.1	Neanderthal	90	3600	50	2000	0.02
S3.1.1	Neanderthal	95	3800	50	2000	0.02
S3.1.1	Neanderthal	100	4000	50	2000	0.02
S3.1.1	Neanderthal	105	4200	50	2000	0.02
S3.1.1	Neanderthal	110	4400	50	2000	0.02
S3.1.1	Neanderthal	115	4600	50	2000	0.02
S3.1.2	Denisovan	335	13400	18	720	0.02
S3.1.2	Denisovan	335	13400	26	1040	0.02
S3.1.2	Denisovan	335	13400	34	1360	0.02
S3.1.2	Denisovan	335	13400	42	1680	0.02
S3.1.2	Denisovan	335	13400	50	2000	0.02
S3.1.2	Denisovan	335	13400	58	2320	0.02
S3.1.2	Denisovan	335	13400	66	2640	0.02
S3.1.2	Denisovan	335	13400	74	2960	0.02
S3.1.2	Denisovan	335	13400	82	3280	0.02
S3.1.2	Neanderthal	95	3800	18	720	0.02
S3.1.2	Neanderthal	95	3800	26	1040	0.02
S3.1.2	Neanderthal	95	3800	34	1360	0.02
S3.1.2	Neanderthal	95	3800	42	1680	0.02
S3.1.2	Neanderthal	95	3800	50	2000	0.02
S3.1.2	Neanderthal	95	3800	58	2320	0.02
S3.1.2	Neanderthal	95	3800	66	2640	0.02
S3.1.2	Neanderthal	95	3800	74	2960	0.02

Section	Archaic Lineage	Divergence Time (kya)	Divergence Time (gen)	Introgression Time (kya)	Introgression Time (gen)	Introgression Proportion
S3.1.2	Neanderthal	95	3800	82	3280	0.02
S3.1.3	Denisovan	335	13400	50	2000	0.004
S3.1.3	Denisovan	335	13400	50	2000	0.008
S3.1.3	Denisovan	335	13400	50	2000	0.012
S3.1.3	Denisovan	335	13400	50	2000	0.016
S3.1.3	Denisovan	335	13400	50	2000	0.02
S3.1.3	Denisovan	335	13400	50	2000	0.024
S3.1.3	Denisovan	335	13400	50	2000	0.028
S3.1.3	Denisovan	335	13400	50	2000	0.032
S3.1.3	Denisovan	335	13400	50	2000	0.036
S3.1.3	Denisovan	335	13400	50	2000	0.04
S3.1.3	Neanderthal	95	3800	50	2000	0.004
S3.1.3	Neanderthal	95	3800	50	2000	0.008
S3.1.3	Neanderthal	95	3800	50	2000	0.012
S3.1.3	Neanderthal	95	3800	50	2000	0.016
S3.1.3	Neanderthal	95	3800	50	2000	0.02
S3.1.3	Neanderthal	95	3800	50	2000	0.024
S3.1.3	Neanderthal	95	3800	50	2000	0.028
S3.1.3	Neanderthal	95	3800	50	2000	0.032
S3.1.3	Neanderthal	95	3800	50	2000	0.036
S3.1.3	Neanderthal	95	3800	50	2000	0.04
S3.1.4	Denisovan	335	13400	26,42	1040,1680	0.004,0.016
S3.1.4	Denisovan	335	13400	26,42	1040,1680	0.008,0.012
S3.1.4	Denisovan	335	13400	26,42	1040,1680	0.012,0.008
S3.1.4	Denisovan	335	13400	26,42	1040,1680	0.016,0.004
S3.1.4	Denisovan	335	13400	26,58	1040,2320	0.004,0.016
S3.1.4	Denisovan	335	13400	26,58	1040,2320	0.008,0.012
S3.1.4	Denisovan	335	13400	26,58	1040,2320	0.012,0.008
S3.1.4	Denisovan	335	13400	26,58	1040,2320	0.016,0.004
S3.1.4	Denisovan	335	13400	26,74	1040,2960	0.004,0.016
S3.1.4	Denisovan	335	13400	26,74	1040,2960	0.008,0.012
S3.1.4	Denisovan	335	13400	26,74	1040,2960	0.012,0.008
S3.1.4	Denisovan	335	13400	26,74	1040,2960	0.016,0.004
S3.1.4	Denisovan	335	13400	42,58	1680,2320	0.004,0.016
S3.1.4	Denisovan	335	13400	42,58	1680,2320	0.008,0.012
S3.1.4	Denisovan	335	13400	42,58	1680,2320	0.012,0.008
S3.1.4	Denisovan	335	13400	42,58	1680,2320	0.016,0.004
S3.1.4	Denisovan	335	13400	42,74	1680,2960	0.004,0.016
S3.1.4	Denisovan	335	13400	42,74	1680,2960	0.008,0.012

Section	Archaic Lineage	Divergence Time (kya)	Divergence Time (gen)	Introgression Time (kya)	Introgression Time (gen)	Introgression Proportion
S3.1.4	Denisovan	335	13400	42,74	1680,2960	0.012,0.008
S3.1.4	Denisovan	335	13400	42,74	1680,2960	0.016,0.004
S3.1.4	Denisovan	335	13400	58,74	2320,2960	0.004,0.016
S3.1.4	Denisovan	335	13400	58,74	2320,2960	0.008,0.012
S3.1.4	Denisovan	335	13400	58,74	2320,2960	0.012,0.008
S3.1.4	Denisovan	335	13400	58,74	2320,2960	0.016,0.004
S3.1.4	Neanderthal	95	3800	26,42	1040,1680	0.004,0.016
S3.1.4	Neanderthal	95	3800	26,42	1040,1680	0.008,0.012
S3.1.4	Neanderthal	95	3800	26,42	1040,1680	0.012,0.008
S3.1.4	Neanderthal	95	3800	26,42	1040,1680	0.016,0.004
S3.1.4	Neanderthal	95	3800	26,58	1040,2320	0.004,0.016
S3.1.4	Neanderthal	95	3800	26,58	1040,2320	0.008,0.012
S3.1.4	Neanderthal	95	3800	26,58	1040,2320	0.012,0.008
S3.1.4	Neanderthal	95	3800	26,58	1040,2320	0.016,0.004
S3.1.4	Neanderthal	95	3800	26,74	1040,2960	0.004,0.016
S3.1.4	Neanderthal	95	3800	26,74	1040,2960	0.008,0.012
S3.1.4	Neanderthal	95	3800	26,74	1040,2960	0.012,0.008
S3.1.4	Neanderthal	95	3800	26,74	1040,2960	0.016,0.004
S3.1.4	Neanderthal	95	3800	42,58	1680,2320	0.004,0.016
S3.1.4	Neanderthal	95	3800	42,58	1680,2320	0.008,0.012
S3.1.4	Neanderthal	95	3800	42,58	1680,2320	0.012,0.008
S3.1.4	Neanderthal	95	3800	42,58	1680,2320	0.016,0.004
S3.1.4	Neanderthal	95	3800	42,74	1680,2960	0.004,0.016
S3.1.4	Neanderthal	95	3800	42,74	1680,2960	0.008,0.012
S3.1.4	Neanderthal	95	3800	42,74	1680,2960	0.012,0.008
S3.1.4	Neanderthal	95	3800	42,74	1680,2960	0.016,0.004
S3.1.4	Neanderthal	95	3800	58,74	2320,2960	0.004,0.016
S3.1.4	Neanderthal	95	3800	58,74	2320,2960	0.008,0.012
S3.1.4	Neanderthal	95	3800	58,74	2320,2960	0.012,0.008
S3.1.4	Neanderthal	95	3800	58,74	2320,2960	0.016,0.004
S3.3	Unknown	467.5	18700	50	2000	0.02
S3.3	Unknown	515	20600	50	2000	0.02
S3.3	Unknown	562.5	22500	50	2000	0.02
S3.3	Unknown	610	24400	50	2000	0.02

Supplementary Table 3.2 **Simulation parameters of introgression from two archaic lineages**

Section	Denisovan Introgression Time (kya)	Denisovan Introgression Time (gen)	Denisovan Introgression Proportion	Neanderthal Introgression Time (kya)	Neanderthal Introgression Time (gen)	Neanderthal Introgression Proportion
S3.2	30	1200	0.004	30	1200	0.016
S3.2	30	1200	0.008	30	1200	0.012
S3.2	30	1200	0.012	30	1200	0.008
S3.2	30	1200	0.016	30	1200	0.004
S3.2	30	1200	0.004	50	2000	0.016
S3.2	30	1200	0.008	50	2000	0.012
S3.2	30	1200	0.012	50	2000	0.008
S3.2	30	1200	0.016	50	2000	0.004
S3.2	30	1200	0.004	70	2800	0.016
S3.2	30	1200	0.008	70	2800	0.012
S3.2	30	1200	0.012	70	2800	0.008
S3.2	30	1200	0.016	70	2800	0.004
S3.2	50	2000	0.004	30	1200	0.016
S3.2	50	2000	0.008	30	1200	0.012
S3.2	50	2000	0.012	30	1200	0.008
S3.2	50	2000	0.016	30	1200	0.004
S3.2	50	2000	0.004	50	2000	0.016
S3.2	50	2000	0.008	50	2000	0.012
S3.2	50	2000	0.012	50	2000	0.008
S3.2	50	2000	0.016	50	2000	0.004
S3.2	50	2000	0.004	70	2800	0.016
S3.2	50	2000	0.008	70	2800	0.012
S3.2	50	2000	0.012	70	2800	0.008
S3.2	50	2000	0.016	70	2800	0.004
S3.2	70	2800	0.004	30	1200	0.016
S3.2	70	2800	0.008	30	1200	0.012
S3.2	70	2800	0.012	30	1200	0.008
S3.2	70	2800	0.016	30	1200	0.004
S3.2	70	2800	0.004	50	2000	0.016
S3.2	70	2800	0.008	50	2000	0.012
S3.2	70	2800	0.012	50	2000	0.008
S3.2	70	2800	0.016	50	2000	0.004
S3.2	70	2800	0.004	70	2800	0.016
S3.2	70	2800	0.008	70	2800	0.012
S3.2	70	2800	0.012	70	2800	0.008
S3.2	70	2800	0.016	70	2800	0.004

4 Supplementary Note 4: Simulation Evaluations

We evaluated our method *ArchaicSeeker 2.0* with massive simulation data. The new method developed to find archaic introgression tracks in the modern human genomes and reconstruct the introgression history.

Our evaluations could be divided into two parts, the power, and precision of introgressed sequences detection and the accuracy of introgression history inference. For the history inference, we focused on the accuracy of inferred introgression model, which is the number of introgression events.

Simulation results showed that *ArchaicSeeker 2.0* have a high precision (~ 93.0%), a high true positive rate (~ 90.4%), and a low false-positive rate (~0.14%) in the archaic segments detection. In most simulations, more than 80% of introgressed segments matched to the correct ancestry. For the introgression history inference, *ArchaicSeeker 2.0* inferred the correct scenarios for most cases (122/144, 84.7%).

4.1 Introgressed Segments Detection

We run the *ArchaicSeeker 2.0* with the simulation data of diverse demographic histories (Supplementary Note 3). All of the software parameters are set as default values. The introgression time is 2000 generations ago and the introgression proportion is set to 0.02. The likelihood of archaic shared markers derived from archaic population ε set to 0.99. There are 200 African individuals (haplotypes), 200 Non-African individuals (haplotypes), 2 Altai Denisovan individuals (haplotypes) and 2 Altai Neanderthal individuals (haplotypes) involved in the following analysis. The matching model we used shows as follow (Supplementary Figure 4.1).

"((Africans:100,Non-Africans:100):557.5,(Denisova:340,Neanderthal:350):237.5);"

For those scenarios with introgression only from one of the archaic lineages, we still use the matching model with two archaic hominins.

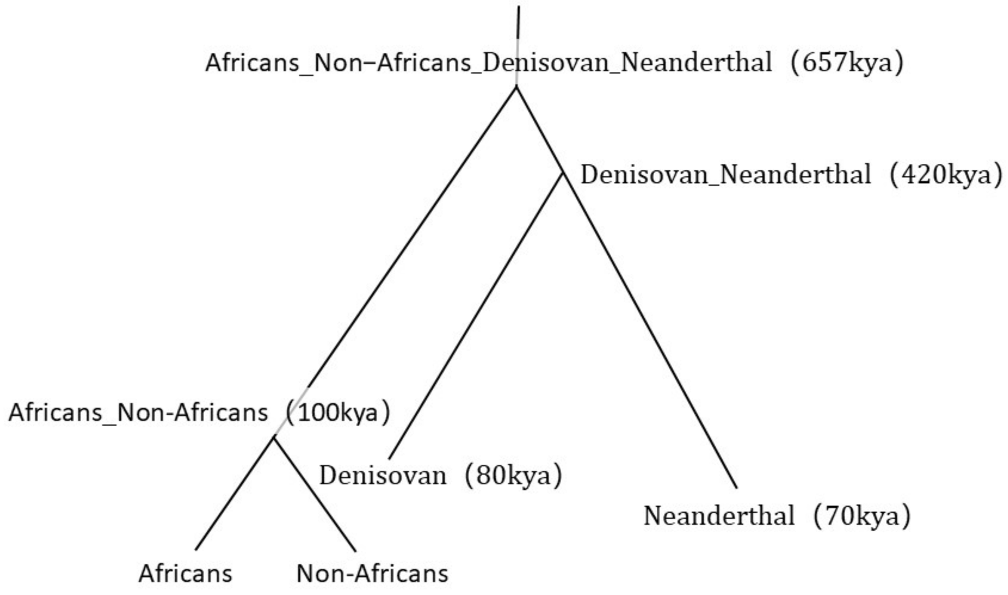

Supplementary Figure 4.1 Matching model used in the simulation data analysis. This figure illustrates the divergence time between different lineages and the time of archaic hominins dead. We used this model to match the candidate archaic segments to the most likely archaic ancestry.

4.1.1 Length-Based Evaluation

Firstly, we directly compared the inferred introgressed segments with the ground-truth introgressed segments. Three simple statistics were used in the following analysis, precision, true positive rate (TPR) and false positive rate (FPR). We defined

$$\text{Precision} = \frac{L_{overlapping}}{L_{inferred}}, \tag{17}$$

$$\text{TPR} = \frac{L_{overlapping}}{L_{intro}}, \tag{18}$$

$$\text{FPR} = \frac{L_{mis-inferred}}{L_{non-intro}}, \tag{19}$$

where $L_{overlapping}$ is the total length of the inferred introgressed sequences overlapping with the ground-truth introgressed sequences; $L_{inferred}$ is the total length

of the inferred introgressed sequences; L_{intro} is the total length of the ground-truth introgressed sequences; $L_{non-intro}$ is the total length of the ground-truth AMH (non-introgressed) sequences.

For all of these 14400 simulations, *ArchaicSeeker 2.0* performed well. The median value of precision is 93.0% (89.4%~95.9%, 95% CI); the median value of TPR is 90.4% (84.1%~94.1%, 95% CI); the median value of FPR is 0.14% (0.07%~0.22%, 95% CI).

Our method exhibits different performance under different simulation scenarios. However, those does not affect our results very much. Our method is very stable and powerful to detect introgressed segments under various introgression scenarios.

Under the scenarios with different divergence time to archaic lineage, T_{split}, the precision, the FPR, and the TPR not affected by the changing of T_{split} very much. For different divergence time, the precision stable above 92% and the FPR is no more than 0.15%. The TPR of Denisovan derived introgression slightly decreased as the increasing of T_{split}. When T_{split} equals to 403 kya, the TPR of Denisovan scenarios drops to 88.9%. The TPR under the Neanderthal introgression scenarios is not significant affected (Supplementary Figure 4.2, Supplementary Table 4.1).

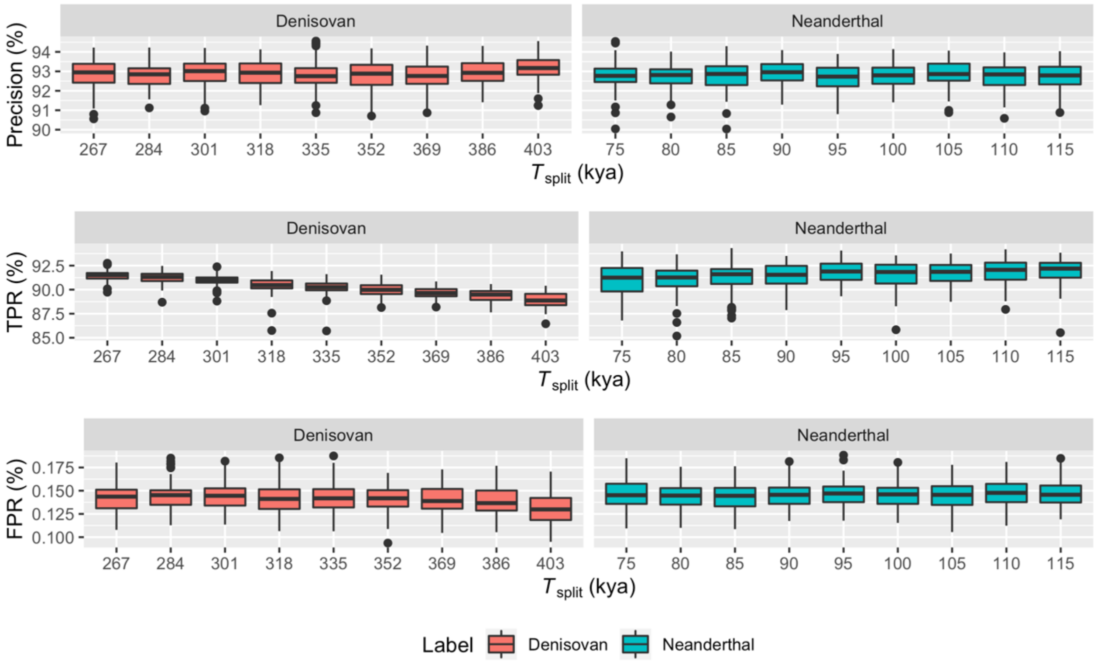

Supplementary Figure 4.2 Length based comparison of "Diverse Divergence Time Scenarios". Comparison between the inferred introgressed segments and the ground truth segments under the

scenarios of different divergence times from the Denisovan and Neanderthal. The x-axis represents the divergence time from the introgressed archaic lineage (Denisovan or Neanderthal) and values are in kya (thousand years ago). The y-axis represents the summary statistics of precision, TPR and FPR. 100 replicates were performed independently and 100 test individuals were analyzed within each replication. Bounds of box represent the interquartile range (IQR; 25th to 75th percentile), with a center line indicating the median. Whiskers are represented in the form of Tukey style.

Supplementary Table 4.1 Length based comparison of "Diverse Divergence Time Scenarios"

Archaic Lineage	T_{split} (kya)	Precision	TPR	FPR
Denisovan	267	93.0%(91.1%~94.0%)*	91.5%(90.3%~92.6%)	0.144%(0.114%~0.173%)
Denisovan	284	92.9%(91.7%~93.8%)	91.3%(90.0%~92.2%)	0.145%(0.116%~0.179%)
Denisovan	301	93.0%(91.2%~94.0%)	91.0%(89.8%~91.9%)	0.145%(0.119%~0.170%)
Denisovan	318	92.9%(91.4%~94.1%)	90.5%(89.2%~91.6%)	0.141%(0.111%~0.173%)
Denisovan	335	92.8%(91.4%~94.4%)	90.3%(88.9%~91.1%)	0.142%(0.117%~0.171%)
Denisovan	352	92.9%(91.1%~93.9%)	90.0%(88.6%~91.2%)	0.142%(0.110%~0.167%)
Denisovan	369	92.8%(91.5%~94.0%)	89.7%(88.6%~90.6%)	0.139%(0.115%~0.172%)
Denisovan	386	92.9%(91.5%~94.0%)	89.5%(87.9%~90.6%)	0.137%(0.116%~0.164%)
Denisovan	403	93.2%(91.6%~94.3%)	88.9%(87.5%~90.2%)	0.130%(0.106%~0.162%)
Neanderthal	75	92.8%(91.2%~94.1%)	91.3%(87.3%~93.6%)	0.145%(0.115%~0.175%)
Neanderthal	80	92.8%(91.4%~93.8%)	91.3%(87.5%~93.6%)	0.145%(0.115%~0.173%)
Neanderthal	85	92.9%(91.4%~94.0%)	91.6%(87.8%~93.3%)	0.145%(0.117%~0.167%)
Neanderthal	90	93.0%(91.6%~94.0%)	91.6%(88.2%~93.4%)	0.146%(0.124%~0.177%)
Neanderthal	95	92.7%(91.3%~93.8%)	91.9%(89.3%~93.9%)	0.147%(0.122%~0.172%)
Neanderthal	100	92.8%(91.6%~93.8%)	91.9%(88.4%~93.5%)	0.146%(0.120%~0.169%)
Neanderthal	105	92.9%(91.5%~94.0%)	91.9%(89.0%~93.6%)	0.146%(0.111%~0.173%)
Neanderthal	110	92.8%(91.2%~93.8%)	92.1%(89.1%~93.5%)	0.148%(0.119%~0.173%)
Neanderthal	115	92.8%(91.2%~93.9%)	92.2%(89.1%~93.7%)	0.146%(0.121%~0.177%)

* This interval is the 95% CI for 100 repeat simulations.

Under the scenarios with different introgression time, T_{intro}, our method performed better in the cases of recent introgression. It is easy to understand that more recent introgressed sequences trend to be longer and are easily to detect. However, the TPR in the scenarios of Neanderthal-derived introgression declines when introgression time is within 30 kya. When T_{intro} equals to 18 kya, the TPR in the Neanderthal

introgressed scenarios drop to ~80%. However, the confidential interval also becomes wide, from 69.1%~97.6% (95% CI). The reason of this unstable might be the introgressed sequences are too long to have sufficient markers to identify the complete introgressed segments. This kind of power declining could only be observed in cases of Neanderthal introgression might because the T_{split} to Denisovan (335 kya) is longer than T_{split} to Neanderthal (95 kya) (Supplementary Figure 4.3, Supplementary Table 4.2).

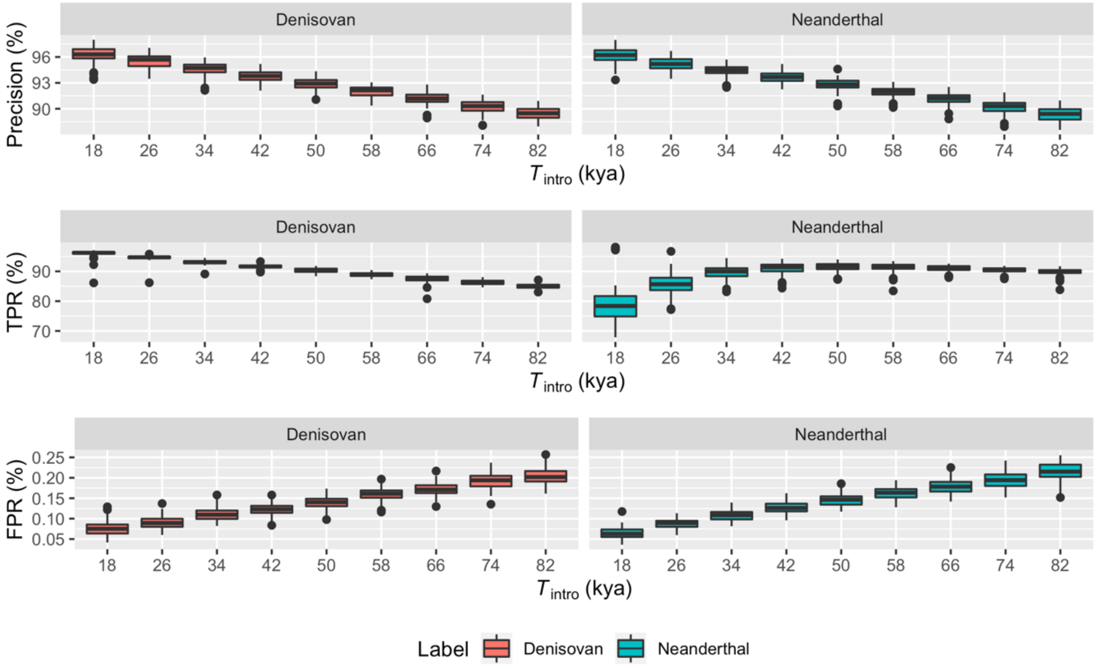

Supplementary Figure 4.3 Length based comparison of "Diverse Introgression Time Scenarios". Comparison between the inferred introgressed segments and the ground truth segments under the scenarios of different introgression time. The x-axis represents the introgression time from the archaic lineage (Denisovan or Neanderthal) and values is in kya (thousand years ago). The y-axis represents the summary statistics of precision, TPR and FPR. 100 replicates were performed independently and 100 test individuals were analyzed within each replication. Bounds of box represent the interquartile range (IQR; 25th to 75th percentile), with a center line indicating the median. Whiskers are represented in the form of Tukey style.

Supplementary Table 4.2 Length based comparison of "Diverse Introgression

Time Scenarios"

Archaic Lineage	T_{intro} (kya)	Precision	TPR	FPR
Denisovan	18	96.3%(94.0%~97.8%)*	96.2%(94.4%~97.0%)	0.075%(0.043%~0.112%)
Denisovan	26	95.7%(94.3%~96.7%)	94.7%(93.8%~95.4%)	0.089%(0.064%~0.122%)
Denisovan	34	94.8%(92.9%~95.6%)	93.1%(92.0%~94.1%)	0.110%(0.084%~0.144%)
Denisovan	42	93.8%(92.5%~95.0%)	91.6%(90.1%~92.8%)	0.123%(0.095%~0.148%)
Denisovan	50	92.9%(91.2%~94.3%)	90.4%(89.0%~91.5%)	0.140%(0.113%~0.171%)
Denisovan	58	92.1%(90.7%~93.0%)	89.0%(87.7%~90.2%)	0.161%(0.129%~0.188%)
Denisovan	66	91.2%(90.0%~92.5%)	87.6%(85.6%~89.1%)	0.171%(0.147%~0.202%)
Denisovan	74	90.3%(88.7%~91.5%)	86.2%(85.0%~87.8%)	0.194%(0.161%~0.228%)
Denisovan	82	89.5%(88.2%~90.7%)	85.1%(83.6%~86.4%)	0.202%(0.166%~0.250%)
Neanderthal	18	96.2%(94.6%~97.8%)	78.5%(69.1%~97.6%)	0.063%(0.036%~0.090%)
Neanderthal	26	95.2%(94.1%~96.3%)	85.7%(77.9%~92.4%)	0.090%(0.068%~0.111%)
Neanderthal	34	94.5%(92.7%~95.6%)	90.0%(84.6%~93.6%)	0.109%(0.086%~0.135%)
Neanderthal	42	93.7%(92.3%~94.7%)	91.4%(85.8%~93.7%)	0.126%(0.098%~0.156%)
Neanderthal	50	92.8%(91.5%~93.7%)	91.8%(87.5%~93.3%)	0.147%(0.120%~0.174%)
Neanderthal	58	92.0%(90.6%~93.0%)	91.7%(87.7%~93.0%)	0.164%(0.134%~0.191%)
Neanderthal	66	91.2%(89.9%~92.1%)	91.2%(88.5%~92.4%)	0.179%(0.144%~0.221%)
Neanderthal	74	90.3%(88.5%~91.6%)	90.6%(88.1%~91.7%)	0.194%(0.160%~0.233%)
Neanderthal	82	89.4%(87.9%~90.7%)	89.9%(87.3%~90.9%)	0.215%(0.178%~0.253%)

* This interval is the 95% CI for 100 repeat simulations.

Under the scenarios of different introgression proportion, α, the TPR is relative stable above 90%. The precision increase as the introgression proportion increase. When the introgression proportion is larger than 2.0%, the precision increased not that greatly. Sufficient introgression genetic materials will help the model to summary the properties of introgression tracks. The FPR also increased while the introgression proportion rise. However, the FPR will not be greater than 0.3% (Supplementary Figure 4.4, Supplementary Table 4.3).

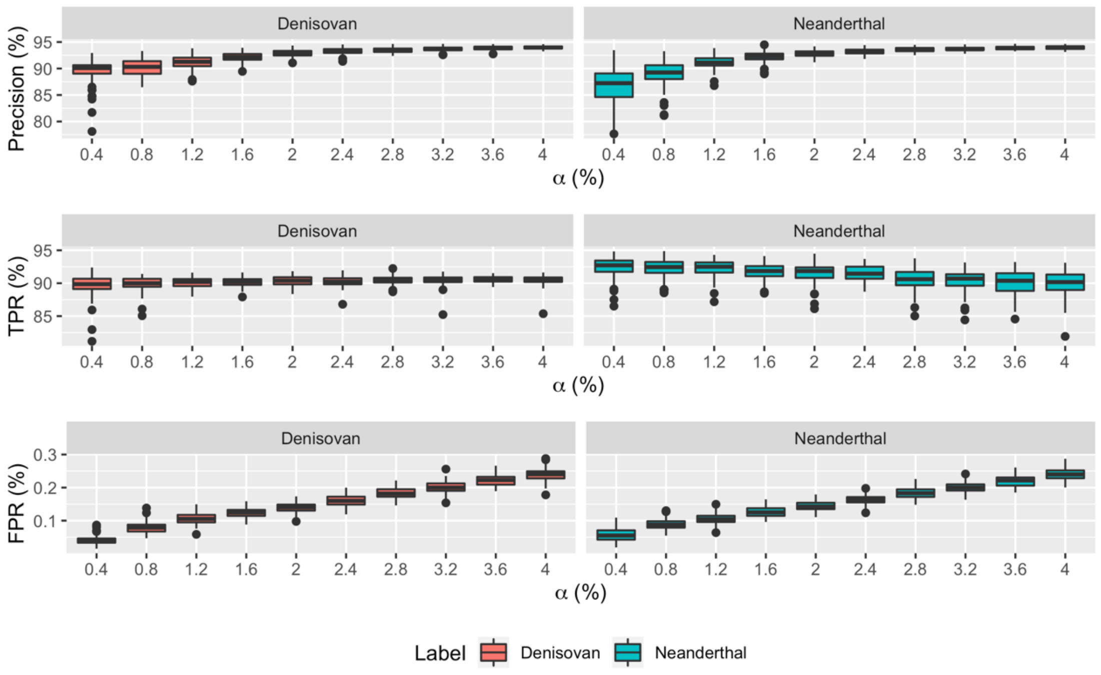

Supplementary Figure 4.4 Length based comparison of "Diverse Introgression Proportion Scenarios". Comparison between the inferred introgressed segments and the ground truth segments under the scenarios of different introgression proportion. The x-axis represents the introgression proportion from the archaic lineage (Denisovan or Neanderthal). The y-axis represents the summary statistics of precision, TPR and FPR. 100 replicates were performed independently and 100 test individuals were analyzed within each replication. Bounds of box represent the interquartile range (IQR; 25th to 75th percentile), with a center line indicating the median. Whiskers are represented in the form of Tukey style.

Supplementary Table 4.3 Length based comparison of "Diverse Introgression Proportion Scenarios"

Archaic Lineage	α	Precision	TPR	FPR
Denisovan	0.40%	90.1%(84.2%~92.7%)*	89.9%(85.9%~92.1%)	0.040%(0.021%~0.070%)
Denisovan	0.80%	90.4%(87.3%~92.8%)	90.0%(87.7%~91.4%)	0.079%(0.050%~0.115%)
Denisovan	1.20%	91.3%(88.1%~93.4%)	90.2%(88.6%~91.5%)	0.105%(0.077%~0.145%)
Denisovan	1.60%	92.2%(90.2%~93.7%)	90.3%(88.8%~91.5%)	0.125%(0.094%~0.152%)
Denisovan	2.00%	92.9%(91.2%~94.3%)	90.4%(89.0%~91.5%)	0.140%(0.113%~0.171%)
Denisovan	2.40%	93.3%(91.9%~94.0%)	90.2%(89.1%~91.7%)	0.160%(0.127%~0.187%)
Denisovan	2.80%	93.5%(92.6%~94.4%)	90.5%(89.1%~91.6%)	0.182%(0.150%~0.217%)
Denisovan	3.20%	93.7%(92.7%~94.5%)	90.5%(89.2%~91.4%)	0.200%(0.160%~0.233%)

Denisovan	3.60%	93.9%(93.2%~94.5%)	90.6%(89.7%~91.4%)	0.223%(0.190%~0.260%)
Denisovan	4.00%	94.0%(93.4%~94.5%)	90.6%(89.4%~91.5%)	0.241%(0.203%~0.273%)
Neanderthal	0.40%	87.3%(78.5%~92.0%)	92.7%(88.8%~94.4%)	0.055%(0.028%~0.099%)
Neanderthal	0.80%	89.3%(83.0%~92.7%)	92.5%(89.0%~94.2%)	0.088%(0.062%~0.127%)
Neanderthal	1.20%	91.1%(88.8%~93.2%)	92.5%(89.3%~93.9%)	0.105%(0.074%~0.135%)
Neanderthal	1.60%	92.3%(89.9%~93.5%)	91.9%(88.9%~93.9%)	0.125%(0.098%~0.158%)
Neanderthal	2.00%	92.9%(91.4%~94.1%)	91.9%(88.3%~94.0%)	0.143%(0.118%~0.169%)
Neanderthal	2.40%	93.3%(92.2%~94.1%)	91.5%(88.9%~93.3%)	0.163%(0.136%~0.190%)
Neanderthal	2.80%	93.5%(92.7%~94.3%)	90.6%(87.0%~93.0%)	0.183%(0.156%~0.220%)
Neanderthal	3.20%	93.6%(93.0%~94.5%)	90.8%(86.3%~92.9%)	0.200%(0.172%~0.229%)
Neanderthal	3.60%	93.9%(93.3%~94.5%)	90.4%(85.8%~92.9%)	0.222%(0.192%~0.257%)
Neanderthal	4.00%	94.0%(93.4%~94.5%)	90.2%(85.9%~92.5%)	0.240%(0.207%~0.279%)

* This interval is the 95% CI for 100 repeat simulations.

Then we allow two-wave introgression from the same ancestry. We set the parameter subscript of the recent wave introgression as 1 and that of the ancient wave event as 2. When the recent introgression proportion α_1 is larger, the precision and the TPR are larger, and the FPR is smaller. For different introgression time combinations, our software performed better when the introgression events are more recent happened. The effect of recent wave is greater than the ancient wave (Supplementary Figure 4.5, Supplementary Table 4.4).

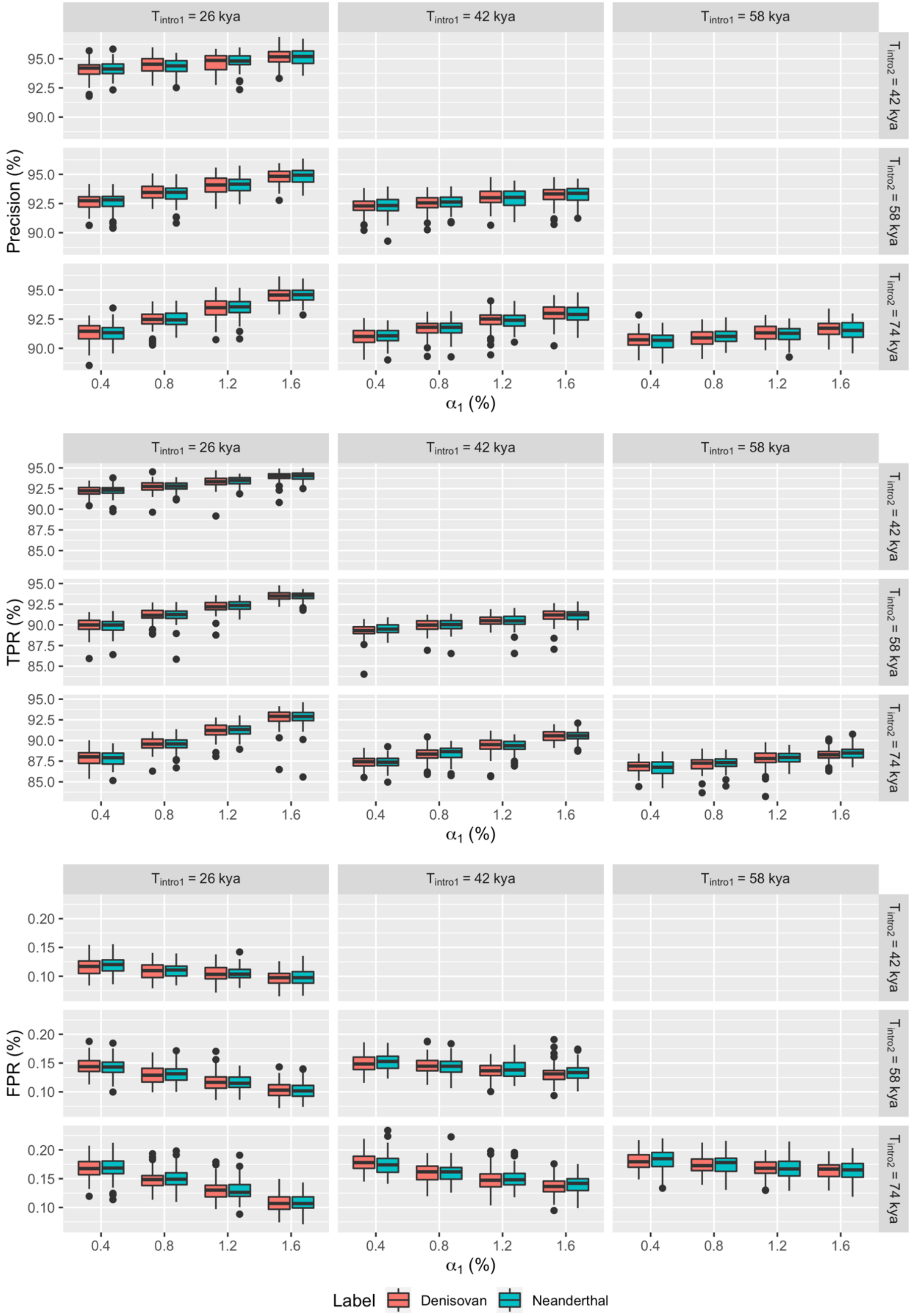

Supplementary Figure 4.5 Length based comparison of "Two Waves Introgression from Single Archaic Population Scenarios". Comparison between the inferred introgressed segments and the ground truth segments under the scenarios of two waves of introgression from one archaic lineage. The introgression time and introgression proportion of the two waves are not constant. Introgression

time of the two waves are different. The summation of the introgression proportion of the two waves is 2%. The x-axis represents the introgression proportion from the first wave. The y-axis represents the summary statistics of precision, TPR and FPR. 100 replicates were performed independently and 100 test individuals were analyzed within each replication. Bounds of box represent the interquartile range (IQR; 25th to 75th percentile), with a center line indicating the median. Whiskers are represented in the form of Tukey style.

Supplementary Table 4.4 **Length based comparison of "Two Waves Introgression from Single Archaic Population Scenarios"**

Archaic Lineage	T_{intro1} (kya)	T_{intro2} (kya)	α_1	α_2	Precision	TPR	FPR
Denisovan	26	42	0.4	1.6	94.2%(92.5%~95.3%)	92.2%(90.9%~93.3%)	0.117%(0.089%~0.141%)
Denisovan	26	42	0.8	1.2	94.6%(93.2%~95.8%)	92.8%(91.7%~93.7%)	0.110%(0.081%~0.139%)
Denisovan	26	42	1.2	0.8	94.9%(92.8%~95.7%)	93.3%(92.3%~94.5%)	0.104%(0.078%~0.134%)
Denisovan	26	42	1.6	0.4	95.2%(93.6%~96.1%)	94.0%(92.8%~94.8%)	0.098%(0.070%~0.123%)
Denisovan	26	58	0.4	1.6	92.7%(91.2%~94.0%)	90.0%(88.3%~91.2%)	0.144%(0.121%~0.176%)
Denisovan	26	58	0.8	1.2	93.5%(92.2%~94.8%)	91.2%(89.4%~92.4%)	0.129%(0.102%~0.159%)
Denisovan	26	58	1.2	0.8	94.1%(92.4%~95.5%)	92.2%(91.0%~93.5%)	0.117%(0.089%~0.151%)
Denisovan	26	58	1.6	0.4	94.8%(93.7%~95.8%)	93.5%(92.5%~94.6%)	0.103%(0.081%~0.131%)
Denisovan	26	74	0.4	1.6	91.5%(89.5%~92.6%)	88.0%(85.9%~89.5%)	0.168%(0.142%~0.203%)
Denisovan	26	74	0.8	1.2	92.5%(90.5%~93.9%)	89.6%(88.3%~90.9%)	0.148%(0.118%~0.183%)
Denisovan	26	74	1.2	0.8	93.5%(91.5%~95.0%)	91.2%(89.5%~92.5%)	0.131%(0.100%~0.168%)
Denisovan	26	74	1.6	0.4	94.6%(93.1%~95.6%)	93.0%(91.0%~94.0%)	0.107%(0.082%~0.148%)
Denisovan	42	58	0.4	1.6	92.3%(90.7%~93.6%)	89.3%(87.7%~90.6%)	0.149%(0.125%~0.178%)
Denisovan	42	58	0.8	1.2	92.6%(91.1%~93.9%)	90.0%(88.6%~91.2%)	0.145%(0.115%~0.173%)
Denisovan	42	58	1.2	0.8	93.0%(91.8%~94.3%)	90.5%(89.6%~91.8%)	0.137%(0.106%~0.159%)
Denisovan	42	58	1.6	0.4	93.4%(91.2%~94.6%)	91.2%(89.5%~92.2%)	0.131%(0.105%~0.167%)
Denisovan	42	74	0.4	1.6	91.0%(89.5%~92.4%)	87.4%(85.9%~89.0%)	0.178%(0.148%~0.217%)
Denisovan	42	74	0.8	1.2	91.8%(90.1%~92.9%)	88.4%(86.2%~89.8%)	0.162%(0.134%~0.191%)
Denisovan	42	74	1.2	0.8	92.5%(90.7%~93.7%)	89.5%(87.5%~90.9%)	0.148%(0.108%~0.187%)
Denisovan	42	74	1.6	0.4	93.0%(91.4%~94.0%)	90.6%(89.2%~91.7%)	0.137%(0.106%~0.164%)
Denisovan	58	74	0.4	1.6	90.7%(89.2%~92.1%)	87.0%(85.3%~88.3%)	0.180%(0.150%~0.210%)
Denisovan	58	74	0.8	1.2	90.9%(89.5%~92.3%)	87.3%(85.7%~88.3%)	0.173%(0.147%~0.206%)

Archaic Lineage	T_{intro1} (kya)	T_{intro2} (kya)	α_1	α_2	Precision	TPR	FPR
Denisovan	58	74	1.2	0.8	91.3%(89.9%~92.6%)	87.8%(85.6%~89.4%)	0.168%(0.131%~0.197%)
Denisovan	58	74	1.6	0.4	91.7%(90.0%~92.8%)	88.3%(86.6%~89.7%)	0.167%(0.136%~0.195%)
Neanderthal	26	42	0.4	1.6	94.1%(92.9%~95.3%)	92.3%(91.1%~93.4%)	0.120%(0.090%~0.152%)
Neanderthal	26	42	0.8	1.2	94.4%(92.9%~95.5%)	92.8%(91.7%~93.7%)	0.111%(0.087%~0.136%)
Neanderthal	26	42	1.2	0.8	94.8%(93.1%~95.9%)	93.5%(92.5%~94.3%)	0.104%(0.084%~0.129%)
Neanderthal	26	42	1.6	0.4	95.2%(93.7%~96.3%)	94.1%(93.1%~94.9%)	0.098%(0.075%~0.126%)
Neanderthal	26	58	0.4	1.6	92.8%(90.8%~94.0%)	90.0%(88.2%~91.1%)	0.144%(0.113%~0.172%)
Neanderthal	26	58	0.8	1.2	93.5%(91.4%~94.7%)	91.3%(90.0%~92.3%)	0.132%(0.105%~0.157%)
Neanderthal	26	58	1.2	0.8	94.2%(92.7%~95.6%)	92.4%(91.2%~93.4%)	0.115%(0.088%~0.142%)
Neanderthal	26	58	1.6	0.4	94.9%(93.5%~96.0%)	93.6%(92.1%~94.3%)	0.102%(0.075%~0.138%)
Neanderthal	26	74	0.4	1.6	91.4%(89.7%~92.9%)	87.9%(86.2%~89.3%)	0.169%(0.125%~0.200%)
Neanderthal	26	74	0.8	1.2	92.4%(91.0%~93.9%)	89.6%(87.7%~90.9%)	0.149%(0.120%~0.189%)
Neanderthal	26	74	1.2	0.8	93.6%(91.9%~94.9%)	91.3%(89.5%~92.7%)	0.126%(0.103%~0.166%)
Neanderthal	26	74	1.6	0.4	94.6%(93.4%~95.8%)	92.9%(91.1%~93.9%)	0.107%(0.083%~0.138%)
Neanderthal	42	58	0.4	1.6	92.3%(90.9%~93.7%)	89.5%(88.1%~90.7%)	0.153%(0.131%~0.180%)
Neanderthal	42	58	0.8	1.2	92.6%(91.4%~93.6%)	90.0%(88.6%~91.2%)	0.145%(0.116%~0.175%)
Neanderthal	42	58	1.2	0.8	93.1%(91.2%~94.3%)	90.5%(88.5%~91.7%)	0.138%(0.113%~0.175%)
Neanderthal	42	58	1.6	0.4	93.4%(91.5%~94.6%)	91.2%(89.7%~92.3%)	0.134%(0.105%~0.165%)
Neanderthal	42	74	0.4	1.6	91.1%(89.6%~92.2%)	87.4%(85.9%~88.7%)	0.174%(0.145%~0.213%)
Neanderthal	42	74	0.8	1.2	91.8%(90.2%~92.8%)	88.7%(86.5%~89.9%)	0.163%(0.132%~0.187%)
Neanderthal	42	74	1.2	0.8	92.4%(91.0%~93.7%)	89.4%(87.5%~90.6%)	0.148%(0.122%~0.190%)
Neanderthal	42	74	1.6	0.4	92.9%(91.5%~94.3%)	90.6%(89.1%~91.7%)	0.142%(0.102%~0.166%)
Neanderthal	58	74	0.4	1.6	90.7%(89.0%~91.9%)	86.8%(85.0%~88.2%)	0.185%(0.146%~0.214%)
Neanderthal	58	74	0.8	1.2	91.0%(89.7%~92.2%)	87.3%(85.8%~88.4%)	0.178%(0.140%~0.210%)

Archaic Lineage	T_{intro1} (kya)	T_{intro2} (kya)	α_1	α_2	Precision	TPR	FPR
Neanderthal	58	74	1.2	0.8	91.3%(89.7%~92.4%)	88.0%(86.2%~89.3%)	0.167%(0.136%~0.205%)
Neanderthal	58	74	1.6	0.4	91.5%(89.8%~92.8%)	88.5%(87.1%~89.8%)	0.165%(0.135%~0.194%)

When we consider the two archaic lineages (Denisovan and Neanderthal) introgressed into modern human population, our results are still good. Firstly, we talk about the precision. If the introgression time of the two lineages, $T_{DenIntro}$ and $T_{NeanIntro}$, are equal, the precision will not be affected by the influence of the bias of introgression proportion. If the introgression time of the two lineages are different, analysis of scenarios with higher proportion of recent wave introgression exhibit higher accuracy. Then we look at the TPR. The pattern is similar with that of the precision, while the Denisovan introgressed segments have a higher weight. At last, we check the FPR. The pattern of the FPR is opposite with the pattern of the precision (Supplementary Figure 4.6, Supplementary Table 4.5).

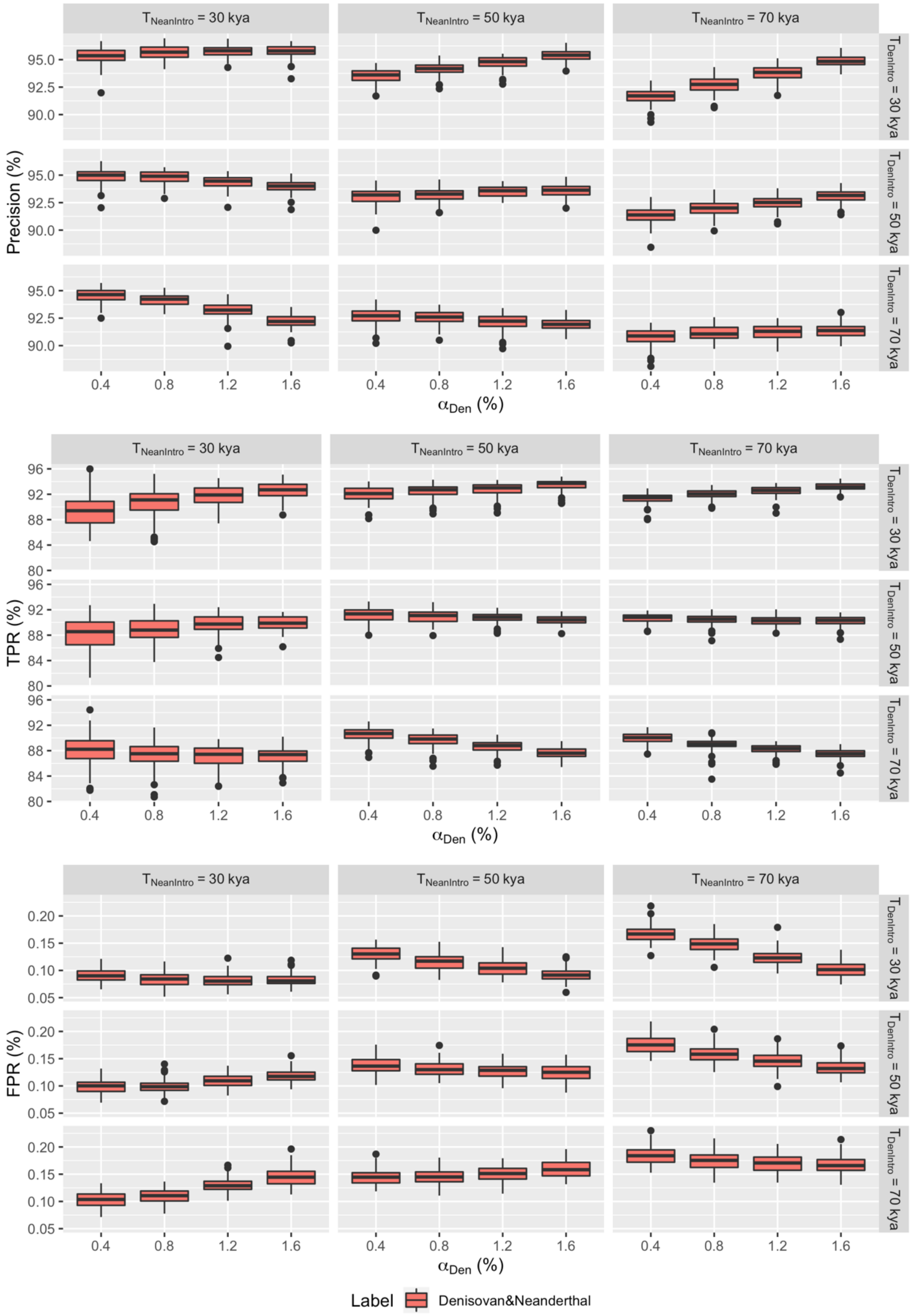

Supplementary Figure 4.6 Length based comparison of "Double Archaic Population Scenarios". Comparison between the inferred introgressed segments and the ground truth segments under the scenarios of two waves of introgression from different archaic lineage. The introgression time and introgression proportion of the two waves are not constant. The summation of the

introgression proportion of the two waves is 2%. The x-axis represents the introgression proportion from the Denisovan lineage. The y-axis represents the summary statistics of precision, TPR and FPR. 100 replicates were performed independently and 100 test individuals were analyzed within each replication. Bounds of box represent the interquartile range (IQR; 25th to 75th percentile), with a center line indicating the median. Whiskers are represented in the form of Tukey style.

Supplementary Table 4.5 **Length based comparison of "Double Archaic Population Scenarios"**

Archaic Lineage	$T_{DenIntro}$ (kya)	$T_{NeanIntro}$ (kya)	α_{Den}	α_{Nean}	Precision	TPR	FPR
Denisovan&Neanderthal	30	30	0.4	1.6	95.4%(94.1%~96.4%)*	89.4%(84.8%~94.1%)	0.090%(0.067%~0.118%)
Denisovan&Neanderthal	30	30	0.8	1.2	95.7%(94.3%~96.8%)	91.1%(85.0%~94.2%)	0.085%(0.065%~0.111%)
Denisovan&Neanderthal	30	30	1.2	0.8	95.8%(94.8%~96.5%)	91.9%(88.0%~94.1%)	0.080%(0.061%~0.102%)
Denisovan&Neanderthal	30	30	1.6	0.4	95.8%(94.4%~96.6%)	92.7%(90.1%~94.5%)	0.081%(0.064%~0.109%)
Denisovan&Neanderthal	30	50	0.4	1.6	93.6%(92.2%~94.6%)	92.2%(88.8%~93.8%)	0.130%(0.103%~0.155%)
Denisovan&Neanderthal	30	50	0.8	1.2	94.2%(92.7%~95.1%)	92.7%(89.7%~94.1%)	0.117%(0.093%~0.145%)
Denisovan&Neanderthal	30	50	1.2	0.8	94.8%(93.1%~95.5%)	93.0%(89.7%~94.1%)	0.104%(0.080%~0.129%)
Denisovan&Neanderthal	30	50	1.6	0.4	95.4%(94.2%~96.2%)	93.7%(91.0%~94.5%)	0.091%(0.072%~0.118%)
Denisovan&Neanderthal	30	70	0.4	1.6	91.7%(90.0%~93.1%)	91.5%(89.5%~92.5%)	0.167%(0.142%~0.202%)
Denisovan&Neanderthal	30	70	0.8	1.2	92.8%(91.3%~94.0%)	92.1%(90.0%~93.2%)	0.149%(0.120%~0.182%)
Denisovan&Neanderthal	30	70	1.2	0.8	93.9%(92.1%~94.8%)	92.6%(91.1%~93.7%)	0.123%(0.099%~0.152%)
Denisovan&Neanderthal	30	70	1.6	0.4	94.8%(93.8%~95.9%)	93.2%(92.0%~94.4%)	0.102%(0.080%~0.127%)
Denisovan&Neanderthal	50	30	0.4	1.6	95.0%(93.4%~96.0%)	88.6%(83.3%~92.3%)	0.100%(0.074%~0.129%)
Denisovan&Neanderthal	50	30	0.8	1.2	94.9%(93.5%~95.6%)	88.8%(84.4%~92.7%)	0.099%(0.078%~0.126%)
Denisovan&Neanderthal	50	30	1.2	0.8	94.5%(93.3%~95.2%)	89.8%(86.5%~92.0%)	0.110%(0.084%~0.129%)
Denisovan&Neanderthal	50	30	1.6	0.4	94.0%(92.9%~95.0%)	89.9%(87.8%~91.6%)	0.118%(0.095%~0.143%)
Denisovan&Neanderthal	50	50	0.4	1.6	93.2%(91.6%~94.2%)	91.4%(88.5%~93.0%)	0.136%(0.113%~0.167%)
Denisovan&Neanderthal	50	50	0.8	1.2	93.3%(91.8%~94.3%)	91.1%(89.0%~92.6%)	0.130%(0.106%~0.159%)
Denisovan&Neanderthal	50	50	1.2	0.8	93.6%(92.6%~94.2%)	90.8%(88.6%~92.1%)	0.129%(0.101%~0.155%)
Denisovan&Neanderthal	50	50	1.6	0.4	93.6%(92.5%~94.7%)	90.4%(89.2%~91.7%)	0.125%(0.098%~0.153%)
Denisovan&Neanderthal	50	70	0.4	1.6	91.4%(89.7%~92.5%)	90.8%(89.0%~91.8%)	0.175%(0.150%~0.207%)
Denisovan&Neanderthal	50	70	0.8	1.2	92.0%(90.4%~93.2%)	90.5%(88.7%~91.7%)	0.159%(0.132%~0.193%)
Denisovan&Neanderthal	50	70	1.2	0.8	92.5%(91.2%~93.5%)	90.3%(88.8%~91.4%)	0.146%(0.118%~0.179%)

Archaic Lineage	$T_{DenIntro}$ (kya)	$T_{NeanIntro}$ (kya)	α_{Den}	α_{Nean}	Precision	TPR	FPR
Denisovan&Neanderthal	50	70	1.6	0.4	93.2%(91.7%~94.0%)	90.3%(88.4%~91.3%)	0.132%(0.112%~0.166%)
Denisovan&Neanderthal	70	30	0.4	1.6	94.7%(93.1%~95.6%)	88.2%(82.1%~92.1%)	0.104%(0.077%~0.128%)
Denisovan&Neanderthal	70	30	0.8	1.2	94.2%(93.1%~94.9%)	87.5%(82.6%~90.6%)	0.111%(0.086%~0.133%)
Denisovan&Neanderthal	70	30	1.2	0.8	93.3%(91.8%~94.1%)	87.5%(82.5%~89.7%)	0.129%(0.106%~0.157%)
Denisovan&Neanderthal	70	30	1.6	0.4	92.2%(91.2%~93.3%)	87.4%(83.7%~88.9%)	0.145%(0.120%~0.177%)
Denisovan&Neanderthal	70	50	0.4	1.6	92.7%(90.9%~93.8%)	90.7%(87.6%~92.3%)	0.145%(0.121%~0.179%)
Denisovan&Neanderthal	70	50	0.8	1.2	92.6%(91.1%~93.6%)	89.8%(86.8%~91.2%)	0.145%(0.116%~0.168%)
Denisovan&Neanderthal	70	50	1.2	0.8	92.2%(90.3%~93.3%)	88.8%(86.3%~90.0%)	0.152%(0.125%~0.176%)
Denisovan&Neanderthal	70	50	1.6	0.4	91.9%(91.0%~92.9%)	87.6%(85.8%~89.4%)	0.158%(0.132%~0.188%)
Denisovan&Neanderthal	70	70	0.4	1.6	90.9%(88.9%~92.0%)	90.1%(88.0%~91.2%)	0.184%(0.154%~0.220%)
Denisovan&Neanderthal	70	70	0.8	1.2	91.1%(89.8%~92.5%)	89.0%(86.2%~90.5%)	0.176%(0.137%~0.201%)
Denisovan&Neanderthal	70	70	1.2	0.8	91.3%(89.8%~92.4%)	88.4%(86.4%~89.4%)	0.171%(0.141%~0.205%)
Denisovan&Neanderthal	70	70	1.6	0.4	91.4%(90.2%~92.5%)	87.5%(85.9%~88.7%)	0.166%(0.136%~0.194%)

* This interval is the 95% CI for 100 repeat simulations.

Under the deep divergent archaic linage introgression scenarios, the precision and FPR is good and stable, while, the TPR decrease greatly when T_{split} increase. When T_{split} equals to 610 kya, the TPR dropped to 81.9% (80.0%~83.5%, 95% CI). Even though the performance is not as good as that of previous scenarios, the TPR is still greater than 80% and precision is about 93% (Supplementary Figure 4.7, Supplementary Table 4.6).

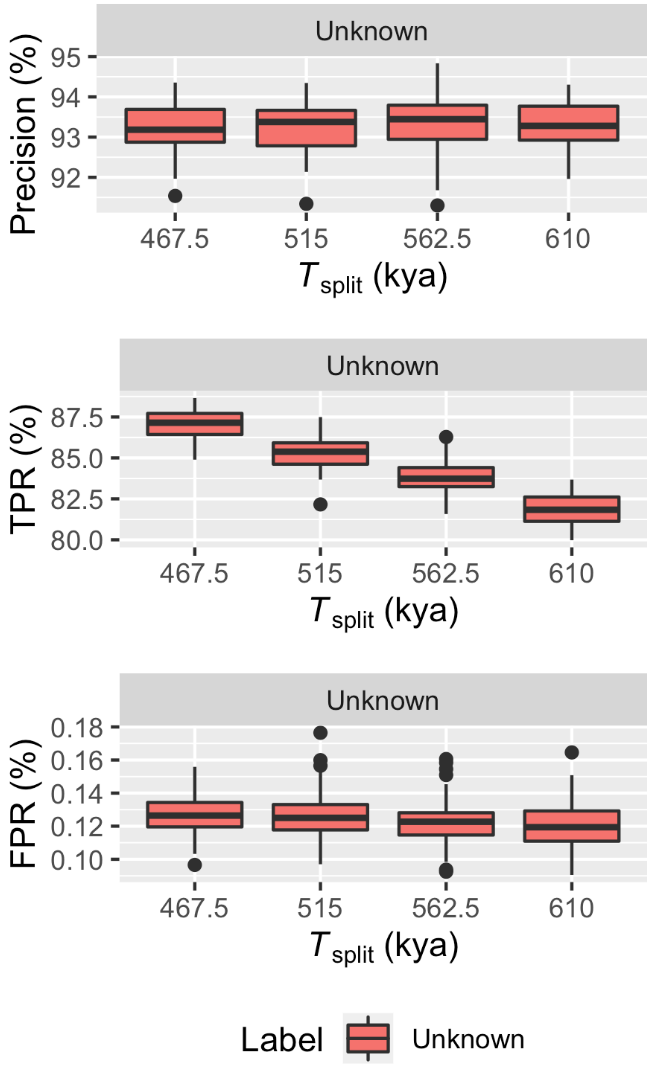

Supplementary Figure 4.7 Length based comparison of "Unknown Archaic Scenarios". Comparison between the inferred introgressed segments and the ground truth segments under the scenarios of deep divergent unknown archaic lineage from the ancestor of Denisovan and Neanderthal. The x-axis represents the divergence time from the archaic lineage and values is in kya (thousand years ago). The y-axis represents the summary statistics of precision, TPR and FPR. 100

replicates were performed independently and 100 test individuals were analyzed within each replication. Bounds of box represent the interquartile range (IQR; 25th to 75th percentile), with a center line indicating the median. Whiskers are represented in the form of Tukey style.

Supplementary Table 4.6 Length based comparison of "Unknown Archaic Scenarios"

Archaic Lineage	T_{split} (kya)	Precision	TPR	FPR
Unknown	467.5	93.2%(92.0%~94.2%)*	87.2%(85.6%~88.5%)	0.126%(0.104%~0.149%)
Unknown	515	93.4%(92.2%~94.2%)	85.4%(83.7%~86.9%)	0.125%(0.103%~0.157%)
Unknown	562.5	93.5%(92.0%~94.4%)	83.7%(82.2%~85.8%)	0.123%(0.098%~0.155%)
Unknown	610	93.3%(92.3%~94.3%)	81.9%(80.0%~83.5%)	0.119%(0.097%~0.150%)

* This interval is the 95% CI for 100 repeat simulations.

4.1.2 SNP-Based Evaluation

To evaluate the performance of our method at SNPs level, we compared the number of SNPs in the inferred introgressed segments with that in the ground-truth introgressed segments. We modified the three simple statistics (17-19). We defined

$$\text{Precision} = \frac{N_{overlapping}}{N_{inferred}}, \tag{20}$$

$$\text{TPR} = \frac{N_{overlapping}}{N_{intro}}, \tag{21}$$

$$\text{FPR} = \frac{N_{mis-inferred}}{N_{non-intro}}, \tag{22}$$

where $N_{overlapping}$ is the total number of SNPs in the inferred introgressed segments overlapping with that in the ground-truth introgressed segments; $N_{inferred}$ is the total number of SNPs in the inferred introgressed segments; N_{intro} is the total number of SNPs in the ground-truth introgressed segments; $N_{non-intro}$ is the total number of SNPs in the ground-truth AMH (non-introgressed) segments.

The performance of the SNPs based statistics are similar with the length based statistics for all of these 14400 simulations. The median value of precision is 93.1%

(89.5%~96.0%, 95% CI); the median value of TPR is 90.6% (84.3%~94.2%, 95% CI); the median value of FPR is 0.14% (0.08%~0.22%, 95% CI) (Supplementary Figure 4.8 ~ 4.13)

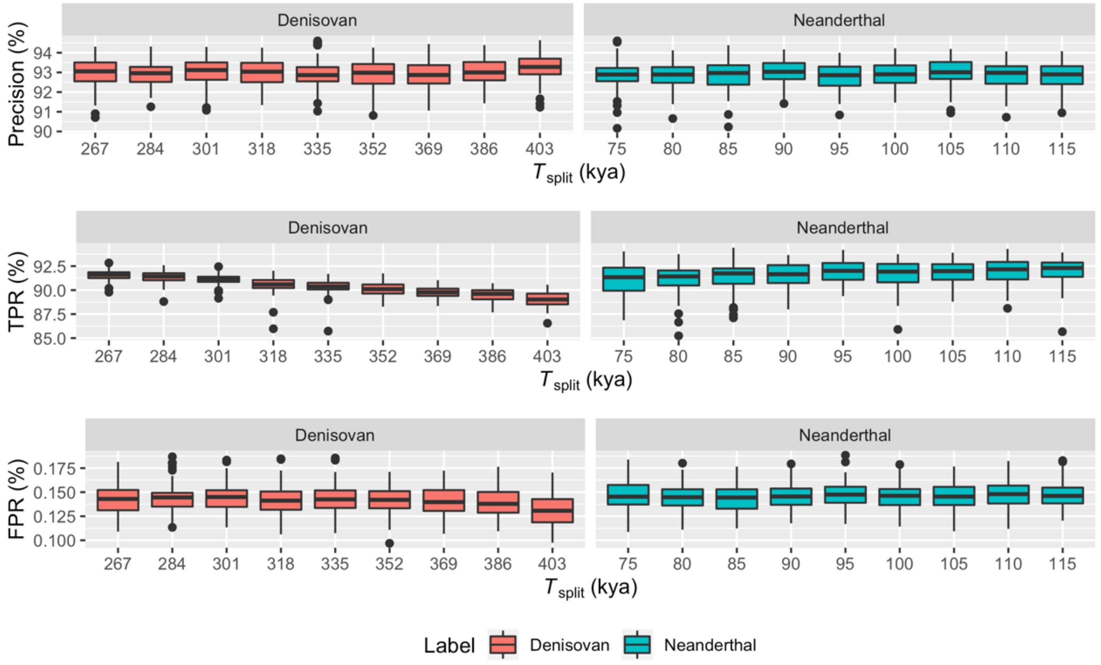

Supplementary Figure 4.8 SNPs based comparison of "Diverse Divergence Time Scenarios". Comparison between the SNPs in the inferred introgressed segments and that in the ground truth segments under the scenarios of different divergence time from the Denisovan and Neanderthal. The x-axis represents the divergence time from the archaic lineage (Denisovan or Neanderthal) and values is in kya (thousand years ago). The y-axis represents the summary statistics of precision, TPR and FPR. 100 replicates were performed independently and 100 test individuals were analyzed within each replication. Bounds of box represent the interquartile range (IQR; 25th to 75th percentile), with a center line indicating the median. Whiskers are represented in the form of Tukey style.

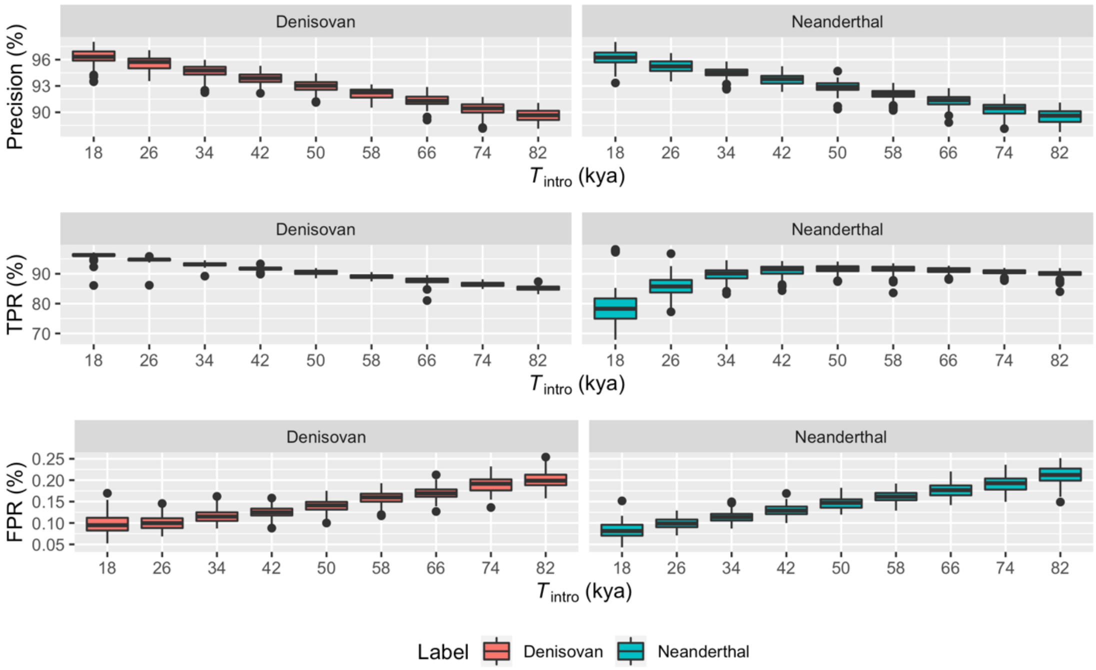

Supplementary Figure 4.9 SNPs based comparison of "Diverse Introgression Time Scenarios". Comparison between SNPs in the inferred introgressed segments and that in the ground truth segments under the scenarios of different introgression time. The x-axis represents the introgression time from the archaic lineage (Denisovan or Neanderthal) and values is in kya (thousand years ago). The y-axis represents the summary statistics of precision, TPR and FPR. 100 replicates were performed independently and 100 test individuals were analyzed within each replication. Bounds of box represent the interquartile range (IQR; 25th to 75th percentile), with a center line indicating the median. Whiskers are represented in the form of Tukey style.

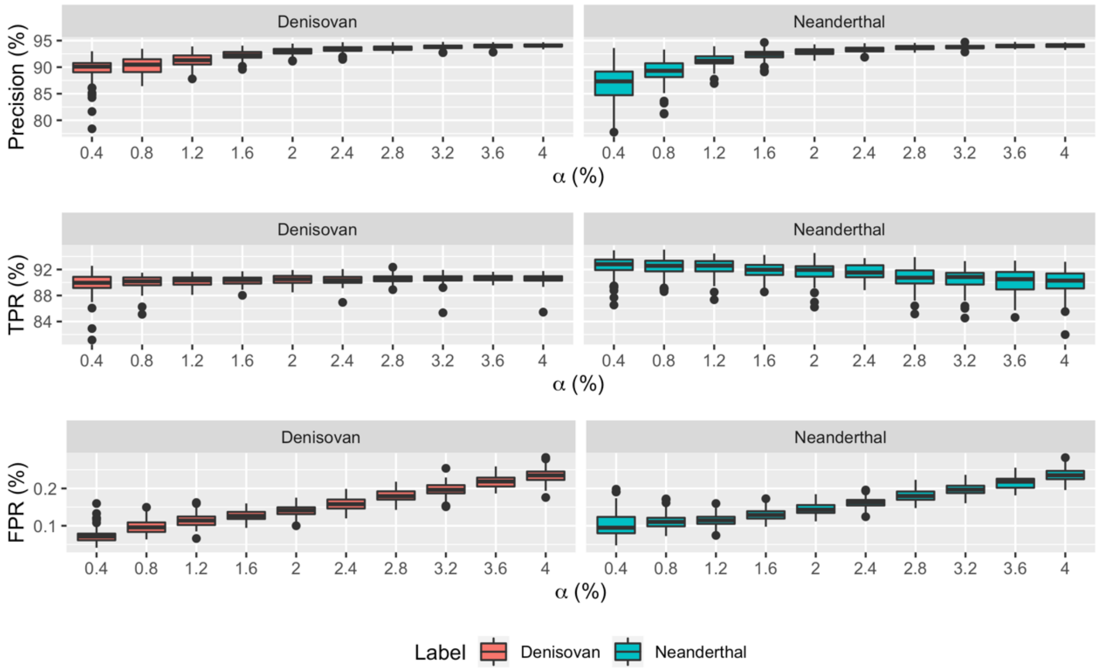

Supplementary Figure 4.10 SNPs based comparison of "Diverse Introgression Proportion Scenarios". Comparison between SNPs in the inferred introgressed segments and that in the ground truth segments under the scenarios of different introgression proportion. The x-axis represents the introgression proportion from the archaic lineage (Denisovan or Neanderthal). The y-axis represents the summary statistics of precision, TPR and FPR. 100 replicates were performed independently and 100 test individuals were analyzed within each replication. Bounds of box represent the interquartile range (IQR; 25th to 75th percentile), with a center line indicating the median. Whiskers are represented in the form of Tukey style.

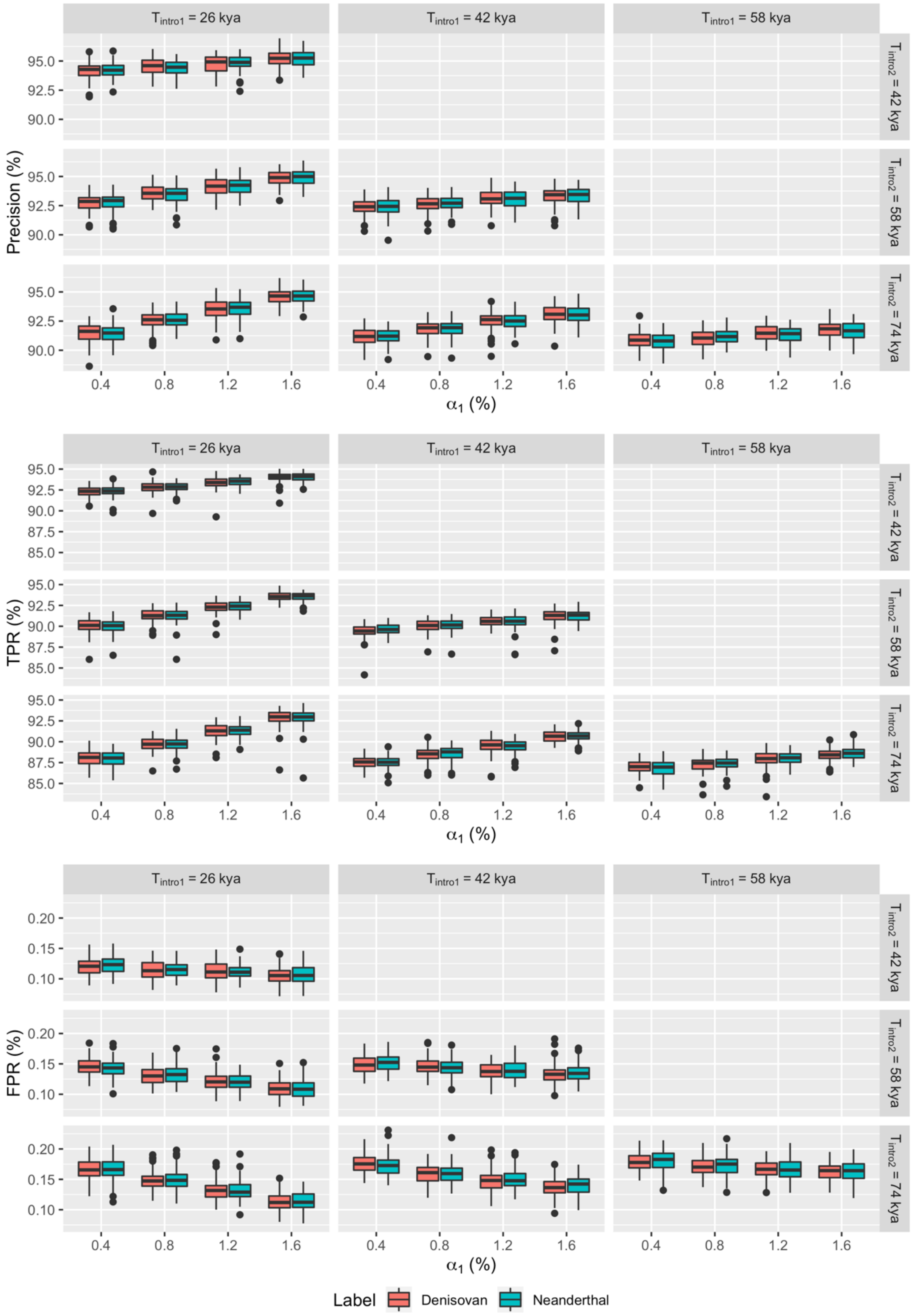

Supplementary Figure 4.11 SNPs based comparison of "Two Waves Introgression from Single Archaic Population Scenarios". Comparison between SNPs in the inferred introgressed segments and that in the ground truth segments under the scenarios of two waves of introgression from one archaic lineage. The introgression time and introgression proportion of the two waves are not

constant. Introgression time of the two waves are different. The summation of the introgression proportion of the two waves is 2%. The x-axis represents the introgression proportion from the first wave. The y-axis represents the summary statistics of precision, TPR and FPR. 100 replicates were performed independently and 100 test individuals were analyzed within each replication. Bounds of box represent the interquartile range (IQR; 25th to 75th percentile), with a center line indicating the median. Whiskers are represented in the form of Tukey style.

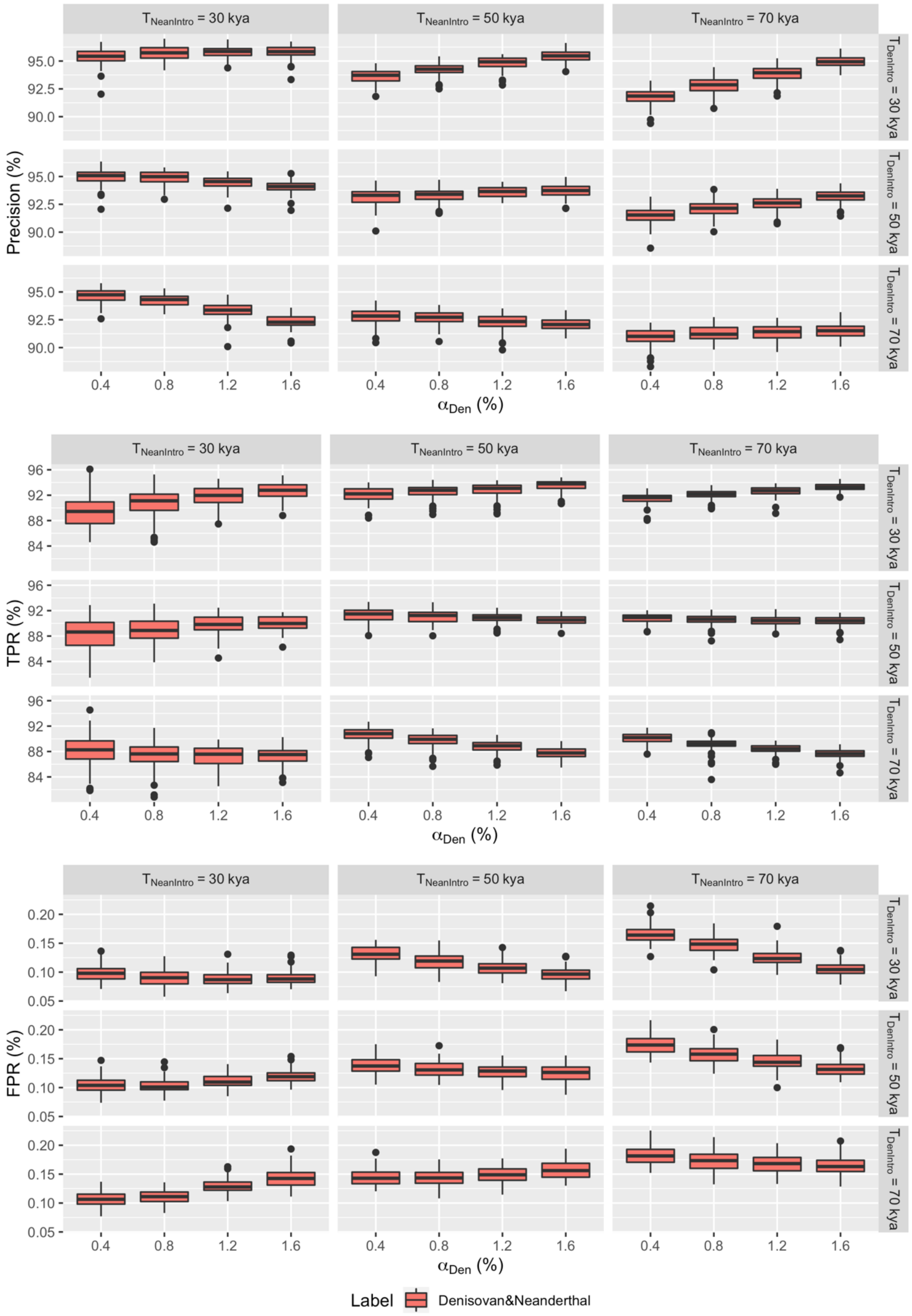

Supplementary Figure 4.12 SNPs based comparison of "Double Archaic Population Scenarios". Comparison between SNPs in the inferred introgressed segments and that in the ground truth segments under the scenarios of two waves of introgression from different archaic lineage. The introgression time and introgression proportion of the two waves are not constant. The summation

of the introgression proportion of the two waves is 2%. The x-axis represents the introgression proportion from the Denisovan lineage. The y-axis represents the summary statistics of precision, TPR and FPR. 100 replicates were performed independently and 100 test individuals were analyzed within each replication. Bounds of box represent the interquartile range (IQR; 25th to 75th percentile), with a center line indicating the median. Whiskers are represented in the form of Tukey style.

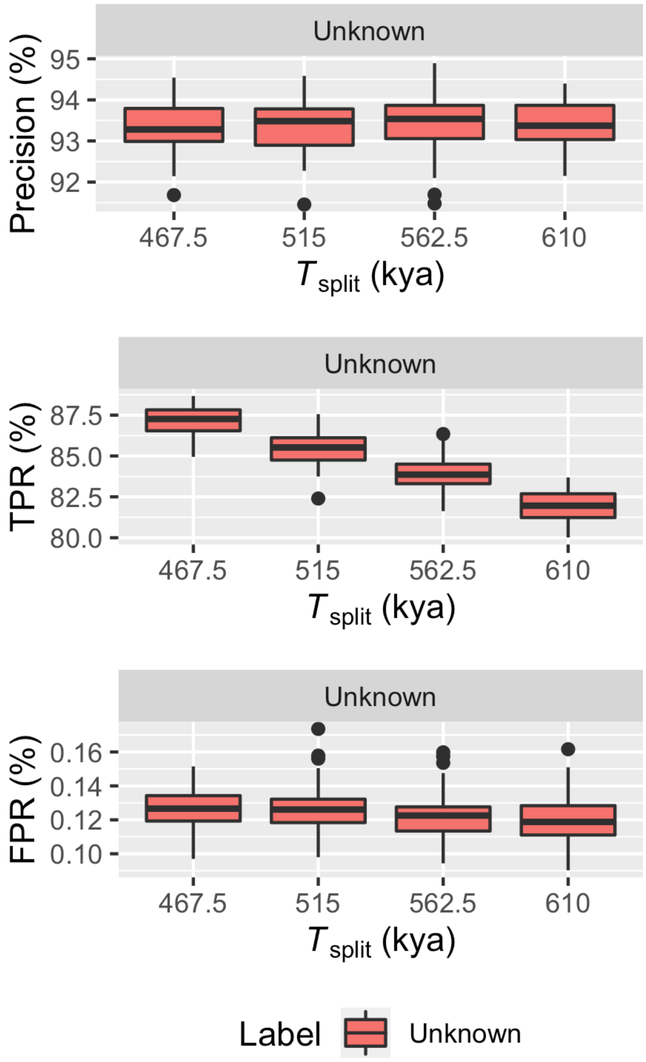

Supplementary Figure 4.13 SNPs based comparison of "Unknown Archaic Scenarios". Comparison between SNPs in the inferred introgressed segments and that in the ground truth segments under the scenarios of deep divergent unknown archaic lineage from the ancestor of Denisovan and Neanderthal. The x-axis represents the divergence time from the archaic lineage and values is in kya (thousand years ago). The y-axis represents the summary statistics of precision, TPR and FPR. 100 replicates were performed independently and 100 test individuals were analyzed

within each replication. Bounds of box represent the interquartile range (IQR; 25th to 75th percentile), with a center line indicating the median. Whiskers are represented in the form of Tukey style.

The most informative markers in our method are the non-AMH ancestry informative markers (AIMs, monomorphic in Africans), which means SNPs not present in Africans, while exist in non-Africans. We selected SNPs with minor allele frequency (MAF) in African equals to zero and MAF in non-African is nonzero, and repeated the SNPs based evaluation.

If we only consider the non-AMH AIMs, the results show that precision of our method reach to around 99%. The median value of the precision is 99.3% (98.9%~99.6%, 95% CI); the median value the TPR is 93.7% (87.1%~96.5%, 95% CI); the median value of the FPR is 0.14% (0.07%~0.24%, 95% CI) (Supplementary Figure 4.14 ~ 4.19, Supplementary Table 4.7 ~4.12).

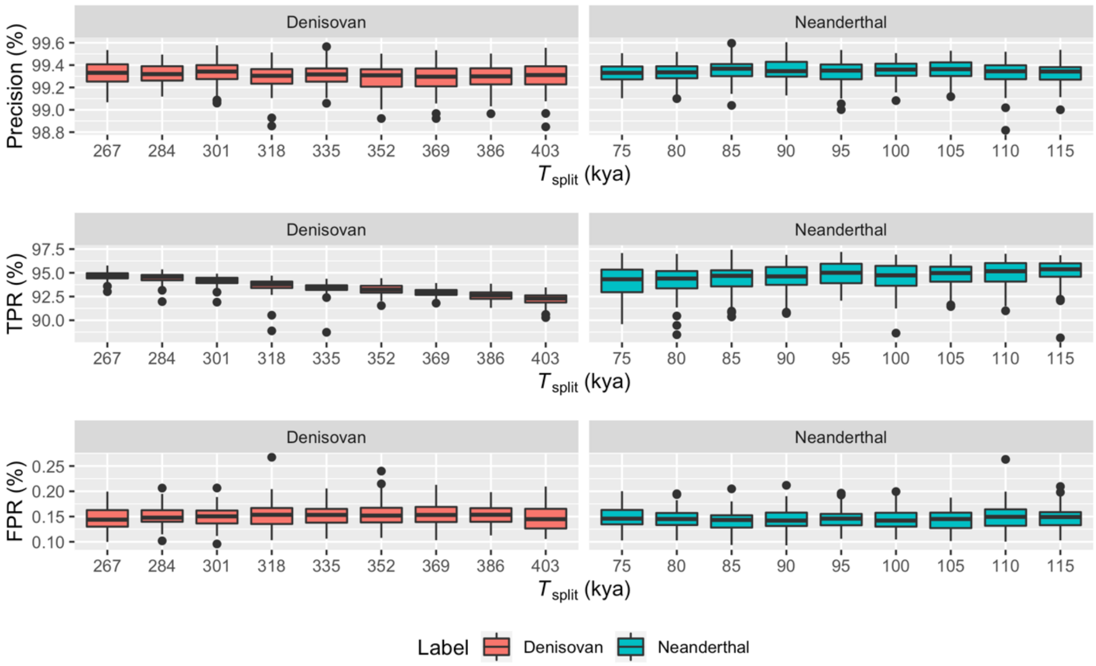

Supplementary Figure 4.14 Comparison of "Diverse Divergence Time Scenarios" for non-AMH markers. Comparison between the non-AMH markers in the inferred introgressed segments and that in the ground truth segments under the scenarios of different divergence time from the Denisovan and. The x-axis represents the divergence time from the archaic lineage (Denisovan or

Neanderthal) and values is in kya (thousand years ago). The y-axis represents the summary statistics of precision, TPR and FPR. 100 replicates were performed independently and 100 test individuals were analyzed within each replication. Bounds of box represent the interquartile range (IQR; 25th to 75th percentile), with a center line indicating the median. Whiskers are represented in the form of Tukey style.

Supplementary Table 4.7 Comparison of "Diverse Divergence Time Scenarios" for non-AMH markers

Archaic Lineage	T_{split} (kya)	Precision	TPR	FPR
Denisovan	267	99.3%(99.1%~99.5%)*	94.7%(93.7%~95.5%)	0.144%(0.108%~0.194%)
Denisovan	284	99.3%(99.2%~99.4%)	94.6%(93.4%~95.2%)	0.148%(0.117%~0.190%)
Denisovan	301	99.3%(99.1%~99.5%)	94.2%(93.1%~94.9%)	0.151%(0.114%~0.188%)
Denisovan	318	99.3%(99.1%~99.5%)	93.8%(92.7%~94.6%)	0.154%(0.114%~0.196%)
Denisovan	335	99.3%(99.1%~99.5%)	93.4%(92.5%~94.2%)	0.153%(0.112%~0.188%)
Denisovan	352	99.3%(99.0%~99.5%)	93.2%(92.1%~94.2%)	0.152%(0.121%~0.209%)
Denisovan	369	99.3%(99.1%~99.5%)	92.9%(92.0%~93.6%)	0.153%(0.115%~0.197%)
Denisovan	386	99.3%(99.1%~99.4%)	92.6%(91.3%~93.5%)	0.154%(0.113%~0.195%)
Denisovan	403	99.3%(99.1%~99.5%)	92.3%(90.8%~93.3%)	0.145%(0.110%~0.200%)
Neanderthal	75	99.3%(99.1%~99.5%)	94.3%(90.1%~96.6%)	0.146%(0.113%~0.194%)
Neanderthal	80	99.3%(99.1%~99.5%)	94.4%(90.5%~96.6%)	0.146%(0.113%~0.182%)
Neanderthal	85	99.4%(99.1%~99.5%)	94.7%(90.8%~96.5%)	0.143%(0.101%~0.176%)
Neanderthal	90	99.3%(99.2%~99.5%)	94.6%(90.9%~96.8%)	0.142%(0.101%~0.190%)
Neanderthal	95	99.4%(99.1%~99.5%)	95.0%(92.4%~96.9%)	0.146%(0.112%~0.186%)
Neanderthal	100	99.4%(99.2%~99.5%)	94.7%(91.3%~96.6%)	0.142%(0.108%~0.190%)
Neanderthal	105	99.4%(99.1%~99.5%)	95.0%(92.0%~96.9%)	0.145%(0.105%~0.179%)
Neanderthal	110	99.4%(99.1%~99.5%)	95.2%(91.9%~96.7%)	0.149%(0.111%~0.195%)
Neanderthal	115	99.3%(99.1%~99.5%)	95.4%(92.2%~96.7%)	0.149%(0.111%~0.195%)

* This interval is the 95% CI for 100 repeat simulations

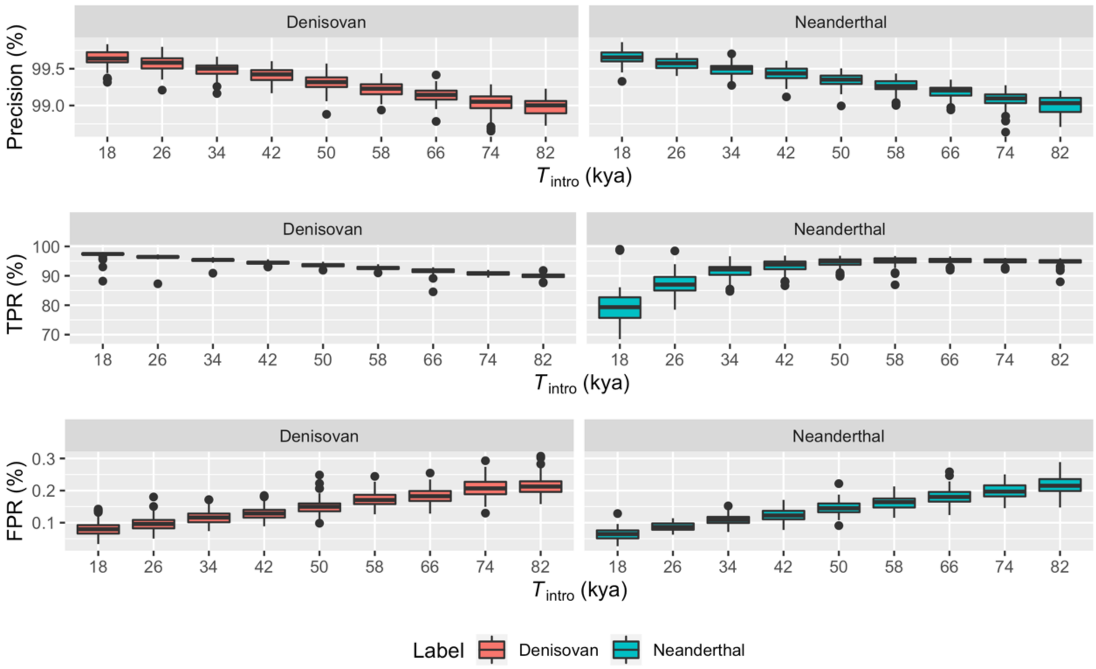

Supplementary Figure 4.15 Comparison of "Diverse Introgression Time Scenarios" for non-AMH markers. Comparison between the non-AMH markers in the inferred introgressed segments and that in the ground truth segments under the scenarios of different introgression time. The x-axis represents the introgression time from the archaic lineage (Denisovan or Neanderthal) and values is in kya (thousand years ago). The y-axis represents the summary statistics of precision, TPR and FPR.

Supplementary Table 4.8 Comparison of "Diverse Introgression Time Scenarios" for non-AMH markers

Archaic Lineage	T_{intro} (kya)	Precision	TPR	FPR
Denisovan	18	99.6%(99.4%~99.8%)	97.4%(95.5%~98.1%)	0.080%(0.042%~0.137%)
Denisovan	26	99.6%(99.4%~99.7%)	96.4%(95.7%~97.1%)	0.096%(0.063%~0.147%)
Denisovan	34	99.5%(99.3%~99.6%)	95.4%(94.5%~96.2%)	0.117%(0.084%~0.160%)
Denisovan	42	99.4%(99.2%~99.6%)	94.5%(93.2%~95.2%)	0.129%(0.093%~0.180%)
Denisovan	50	99.3%(99.1%~99.5%)	93.6%(92.6%~94.5%)	0.150%(0.109%~0.207%)
Denisovan	58	99.2%(99.0%~99.4%)	92.7%(91.5%~93.6%)	0.171%(0.128%~0.216%)
Denisovan	66	99.1%(99.0%~99.3%)	91.8%(90.1%~92.8%)	0.183%(0.142%~0.225%)
Denisovan	74	99.1%(98.7%~99.3%)	90.8%(89.6%~91.9%)	0.207%(0.153%~0.270%)
Denisovan	82	99.0%(98.8%~99.2%)	90.1%(88.3%~91.1%)	0.213%(0.162%~0.283%)

Neanderthal	18	99.7%(99.5%~99.8%)	79.4%(69.9%~98.8%)	0.065%(0.029%~0.093%)
Neanderthal	26	99.6%(99.4%~99.7%)	87.1%(79.2%~93.3%)	0.087%(0.063%~0.113%)
Neanderthal	34	99.5%(99.3%~99.6%)	92.2%(86.6%~95.7%)	0.109%(0.080%~0.144%)
Neanderthal	42	99.4%(99.2%~99.6%)	93.8%(88.0%~96.5%)	0.123%(0.092%~0.164%)
Neanderthal	50	99.4%(99.2%~99.5%)	95.0%(90.4%~96.6%)	0.145%(0.108%~0.187%)
Neanderthal	58	99.3%(99.0%~99.4%)	95.2%(91.0%~96.7%)	0.164%(0.125%~0.198%)
Neanderthal	66	99.2%(99.0%~99.3%)	95.4%(92.7%~96.4%)	0.180%(0.131%~0.229%)
Neanderthal	74	99.1%(98.8%~99.3%)	95.3%(93.1%~96.0%)	0.197%(0.156%~0.247%)
Neanderthal	82	99.0%(98.8%~99.2%)	95.0%(92.0%~95.7%)	0.215%(0.170%~0.272%)

* This interval is the 95% CI for 100 repeat simulations.

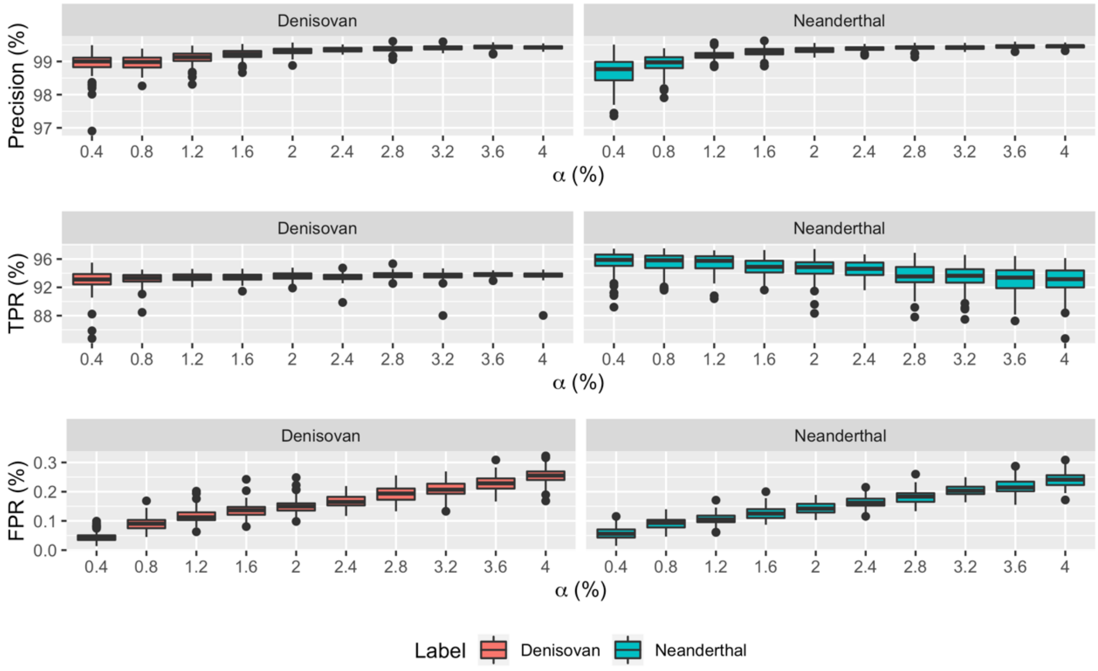

Supplementary Figure 4.16 Comparison of "Diverse Introgression Proportion Scenarios" for non-AMH markers. Comparison between the non-AMH markers in the inferred introgressed segments and that in the ground truth segments under the scenarios of different introgression proportion. The x-axis represents the introgression proportion from the archaic lineage (Denisovan or Neanderthal). The y-axis represents the summary statistics of precision, TPR and FPR. 100 replicates were performed independently and 100 test individuals were analyzed within each replication. Bounds of box represent the interquartile range (IQR; 25th to 75th percentile), with a center line indicating the median. Whiskers are represented in the form of Tukey style.

Supplementary Table 4.9 Comparison of "Diverse Introgression Proportion Scenarios" for non-AMH markers

Archaic Lineage	α	Precision	TPR	FPR
Denisovan	0.4%	99.0%(98.2%~99.4%)*	93.1%(88.2%~95.1%)	0.042%(0.019%~0.083%)
Denisovan	0.8%	99.0%(98.5%~99.4%)	93.3%(91.7%~94.3%)	0.091%(0.056%~0.139%)
Denisovan	1.2%	99.1%(98.6%~99.4%)	93.4%(92.1%~94.5%)	0.112%(0.079%~0.176%)
Denisovan	1.6%	99.2%(98.9%~99.5%)	93.4%(92.4%~94.3%)	0.137%(0.092%~0.180%)
Denisovan	2.0%	99.3%(99.1%~99.5%)	93.6%(92.6%~94.5%)	0.150%(0.109%~0.207%)
Denisovan	2.4%	99.4%(99.2%~99.5%)	93.5%(92.6%~94.5%)	0.165%(0.134%~0.212%)
Denisovan	2.8%	99.4%(99.2%~99.5%)	93.7%(92.8%~94.5%)	0.194%(0.139%~0.251%)
Denisovan	3.2%	99.4%(99.3%~99.5%)	93.7%(92.7%~94.5%)	0.207%(0.145%~0.259%)
Denisovan	3.6%	99.4%(99.3%~99.5%)	93.8%(93.1%~94.3%)	0.230%(0.180%~0.280%)
Denisovan	4.0%	99.4%(99.3%~99.5%)	93.7%(93.0%~94.3%)	0.255%(0.203%~0.316%)
Neanderthal	0.4%	98.8%(97.7%~99.4%)	95.9%(91.2%~97.3%)	0.056%(0.019%~0.107%)
Neanderthal	0.8%	99.0%(98.2%~99.3%)	95.9%(91.9%~97.5%)	0.094%(0.059%~0.136%)
Neanderthal	1.2%	99.2%(98.9%~99.4%)	95.8%(92.5%~97.1%)	0.105%(0.066%~0.142%)
Neanderthal	1.6%	99.3%(98.9%~99.5%)	94.9%(91.9%~97.1%)	0.125%(0.090%~0.176%)
Neanderthal	2.0%	99.4%(99.2%~99.5%)	94.9%(91.5%~97.0%)	0.142%(0.105%~0.181%)
Neanderthal	2.4%	99.4%(99.2%~99.5%)	94.6%(91.7%~96.3%)	0.162%(0.129%~0.206%)
Neanderthal	2.8%	99.4%(99.2%~99.5%)	93.6%(90.0%~96.4%)	0.183%(0.148%~0.231%)
Neanderthal	3.2%	99.4%(99.3%~99.5%)	93.7%(89.1%~96.0%)	0.203%(0.169%~0.242%)
Neanderthal	3.6%	99.4%(99.3%~99.6%)	93.4%(88.3%~96.1%)	0.215%(0.172%~0.282%)
Neanderthal	4.0%	99.5%(99.3%~99.6%)	93.2%(88.4%~96.0%)	0.242%(0.195%~0.301%)

* This interval is the 95% CI for 100 repeat simulations.

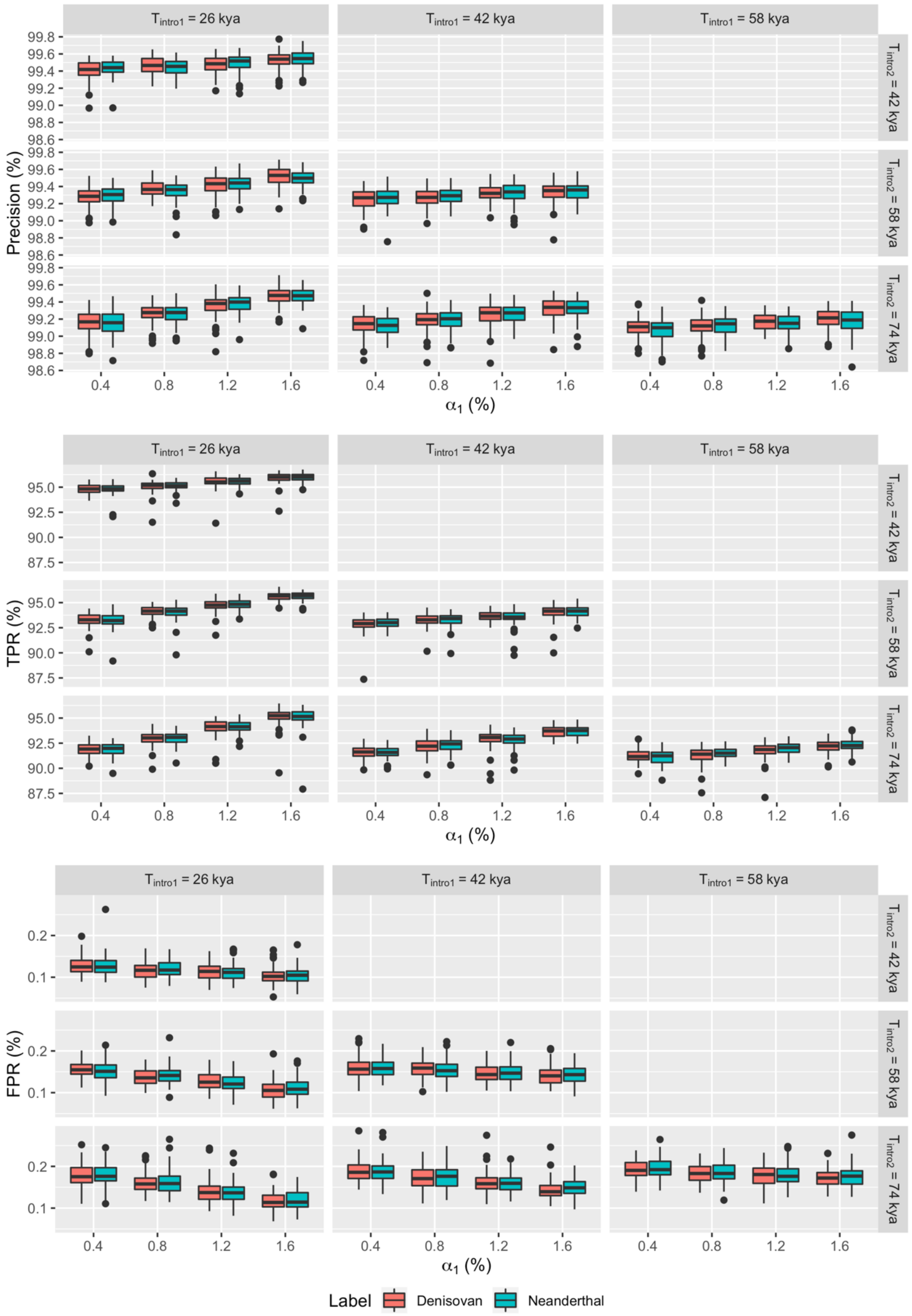

Supplementary Figure 4.17 Comparison of "Two Waves Introgression from Single Archaic Population Scenarios" for non-AMH markers. Comparison between non-AMH markers in the inferred introgressed segments and that in the ground truth segments under the scenarios of two waves of introgression from one archaic lineage. The introgression time and introgression

proportion of the two waves are not constant. Introgression time of the two waves are different. The summation of the introgression proportion of the two waves is 2%. The x-axis represents the introgression proportion from the first wave. The y-axis represents the summary statistics of precision, TPR and FPR. 100 replicates were performed independently and 100 test individuals were analyzed within each replication. Bounds of box represent the interquartile range (IQR; 25th to 75th percentile), with a center line indicating the median. Whiskers are represented in the form of Tukey style.

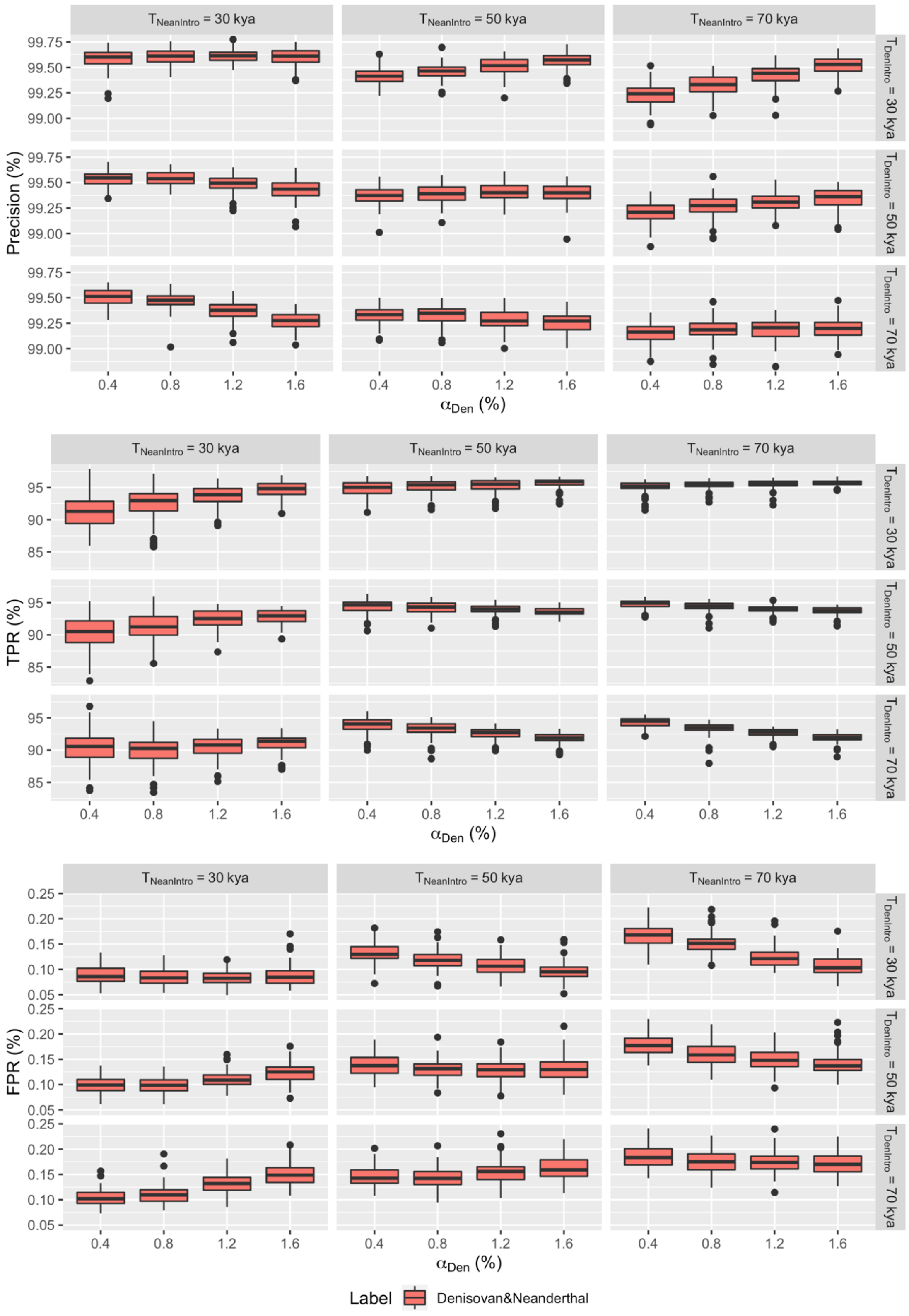

Supplementary Figure 4.18 Comparison of "Double Archaic Population Scenarios" for non-AMH makers. Comparison between non-AMH markers in the inferred introgressed segments and that in the ground truth segments under the scenarios of two waves of introgression from different archaic lineage. The introgression time and introgression proportion of the two waves are not

constant. The summation of the introgression proportion of the two waves is 2%. The x-axis represents the introgression proportion from the Denisovan lineage. The y-axis represents the summary statistics of precision, TPR and FPR. 100 replicates were performed independently and 100 test individuals were analyzed within each replication. Bounds of box represent the interquartile range (IQR; 25th to 75th percentile), with a center line indicating the median. Whiskers are represented in the form of Tukey style.

Supplementary Table 4.10 Comparison of "Two Waves Introgression from Single Archaic Population Scenarios" for non-AMH markers

Archaic Lineage	T_{intro1} (kya)	T_{intro2} (kya)	α_1	α_2	Precision	TPR	FPR
Denisovan	26	42	0.4	1.6	99.4%(99.2%~99.6%)*	94.8%(93.8%~95.7%)	0.125%(0.091%~0.171%)
Denisovan	26	42	0.8	1.2	99.5%(99.3%~99.6%)	95.2%(94.3%~95.7%)	0.117%(0.080%~0.156%)
Denisovan	26	42	1.2	0.8	99.5%(99.2%~99.6%)	95.5%(94.7%~96.4%)	0.114%(0.077%~0.152%)
Denisovan	26	42	1.6	0.4	99.5%(99.3%~99.7%)	96.0%(95.3%~96.6%)	0.102%(0.073%~0.154%)
Denisovan	26	58	0.4	1.6	99.3%(99.0%~99.4%)	93.3%(92.2%~94.3%)	0.155%(0.120%~0.199%)
Denisovan	26	58	0.8	1.2	99.4%(99.2%~99.5%)	94.2%(92.8%~95.1%)	0.137%(0.100%~0.176%)
Denisovan	26	58	1.2	0.8	99.4%(99.1%~99.6%)	94.8%(93.6%~95.8%)	0.125%(0.089%~0.177%)
Denisovan	26	58	1.6	0.4	99.5%(99.3%~99.7%)	95.7%(94.7%~96.5%)	0.106%(0.069%~0.145%)
Denisovan	26	74	0.4	1.6	99.2%(98.8%~99.4%)	91.9%(90.4%~93.1%)	0.175%(0.129%~0.233%)
Denisovan	26	74	0.8	1.2	99.3%(99.0%~99.5%)	93.0%(91.7%~94.0%)	0.158%(0.119%~0.216%)
Denisovan	26	74	1.2	0.8	99.4%(99.1%~99.5%)	94.2%(92.8%~95.1%)	0.137%(0.099%~0.194%)
Denisovan	26	74	1.6	0.4	99.5%(99.3%~99.7%)	95.2%(93.4%~96.1%)	0.114%(0.074%~0.155%)
Denisovan	42	58	0.4	1.6	99.3%(99.0%~99.4%)	92.9%(91.7%~93.8%)	0.157%(0.116%~0.220%)
Denisovan	42	58	0.8	1.2	99.3%(99.1%~99.4%)	93.3%(92.3%~94.4%)	0.159%(0.115%~0.197%)
Denisovan	42	58	1.2	0.8	99.3%(99.1%~99.5%)	93.7%(92.7%~94.6%)	0.144%(0.114%~0.193%)
Denisovan	42	58	1.6	0.4	99.4%(99.1%~99.5%)	94.2%(92.8%~95.1%)	0.141%(0.104%~0.193%)
Denisovan	42	74	0.4	1.6	99.1%(98.8%~99.3%)	91.6%(90.5%~92.9%)	0.186%(0.150%~0.228%)
Denisovan	42	74	0.8	1.2	99.2%(98.9%~99.4%)	92.2%(90.7%~93.8%)	0.171%(0.133%~0.222%)
Denisovan	42	74	1.2	0.8	99.3%(99.0%~99.5%)	93.1%(90.8%~93.9%)	0.159%(0.117%~0.217%)
Denisovan	42	74	1.6	0.4	99.3%(99.1%~99.5%)	93.7%(92.6%~94.6%)	0.140%(0.114%~0.186%)
Denisovan	58	74	0.4	1.6	99.1%(98.9%~99.3%)	91.2%(90.1%~92.3%)	0.191%(0.152%~0.236%)
Denisovan	58	74	0.8	1.2	99.1%(98.8%~99.3%)	91.4%(89.6%~92.4%)	0.184%(0.141%~0.229%)
Denisovan	58	74	1.2	0.8	99.2%(99.0%~99.3%)	91.9%(90.1%~93.0%)	0.181%(0.137%~0.219%)
Denisovan	58	74	1.6	0.4	99.2%(98.9%~99.4%)	92.2%(90.9%~93.4%)	0.172%(0.130%~0.215%)

Archaic Lineage	T_{intro1} (kya)	T_{intro2} (kya)	α_1	α_2	Precision	TPR	FPR
Neanderthal	26	42	0.4	1.6	99.4%(99.3%~99.6%)	94.8%(94.1%~95.7%)	0.124%(0.091%~0.159%)
Neanderthal	26	42	0.8	1.2	99.5%(99.2%~99.6%)	95.2%(94.2%~95.8%)	0.117%(0.091%~0.157%)
Neanderthal	26	42	1.2	0.8	99.5%(99.2%~99.6%)	95.6%(94.9%~96.2%)	0.112%(0.080%~0.166%)
Neanderthal	26	42	1.6	0.4	99.5%(99.3%~99.7%)	96.0%(95.1%~96.7%)	0.105%(0.065%~0.138%)
Neanderthal	26	58	0.4	1.6	99.3%(99.1%~99.5%)	93.2%(92.1%~94.4%)	0.151%(0.116%~0.193%)
Neanderthal	26	58	0.8	1.2	99.4%(99.1%~99.5%)	94.1%(93.0%~94.9%)	0.141%(0.108%~0.184%)
Neanderthal	26	58	1.2	0.8	99.4%(99.2%~99.6%)	94.8%(93.9%~95.7%)	0.121%(0.083%~0.171%)
Neanderthal	26	58	1.6	0.4	99.5%(99.3%~99.6%)	95.7%(94.9%~96.3%)	0.108%(0.074%~0.161%)
Neanderthal	26	74	0.4	1.6	99.2%(98.9%~99.3%)	92.0%(90.6%~92.9%)	0.176%(0.128%~0.232%)
Neanderthal	26	74	0.8	1.2	99.3%(99.0%~99.5%)	93.1%(91.9%~93.8%)	0.159%(0.118%~0.224%)
Neanderthal	26	74	1.2	0.8	99.4%(99.2%~99.6%)	94.1%(93.0%~95.2%)	0.137%(0.095%~0.185%)
Neanderthal	26	74	1.6	0.4	99.5%(99.3%~99.6%)	95.2%(94.0%~96.1%)	0.115%(0.078%~0.158%)
Neanderthal	42	58	0.4	1.6	99.3%(99.1%~99.4%)	93.0%(92.0%~94.0%)	0.158%(0.119%~0.205%)
Neanderthal	42	58	0.8	1.2	99.3%(99.1%~99.5%)	93.4%(92.1%~94.2%)	0.153%(0.112%~0.208%)
Neanderthal	42	58	1.2	0.8	99.3%(99.0%~99.5%)	93.6%(92.1%~94.6%)	0.147%(0.108%~0.199%)
Neanderthal	42	58	1.6	0.4	99.4%(99.1%~99.5%)	94.2%(93.0%~95.1%)	0.143%(0.108%~0.179%)
Neanderthal	42	74	0.4	1.6	99.1%(98.9%~99.3%)	91.6%(90.2%~92.8%)	0.187%(0.141%~0.231%)
Neanderthal	42	74	0.8	1.2	99.2%(98.9%~99.4%)	92.5%(90.8%~93.5%)	0.176%(0.125%~0.223%)
Neanderthal	42	74	1.2	0.8	99.3%(99.0%~99.5%)	92.9%(91.3%~93.9%)	0.160%(0.119%~0.209%)
Neanderthal	42	74	1.6	0.4	99.3%(99.1%~99.5%)	93.8%(92.6%~94.6%)	0.149%(0.103%~0.189%)
Neanderthal	58	74	0.4	1.6	99.1%(98.8%~99.3%)	91.2%(89.7%~92.3%)	0.193%(0.144%~0.238%)
Neanderthal	58	74	0.8	1.2	99.1%(98.9%~99.3%)	91.5%(90.4%~92.7%)	0.183%(0.140%~0.227%)
Neanderthal	58	74	1.2	0.8	99.2%(98.9%~99.3%)	92.1%(90.6%~93.1%)	0.176%(0.130%~0.244%)
Neanderthal	58	74	1.6	0.4	99.2%(98.9%~99.4%)	92.3%(91.0%~93.7%)	0.177%(0.133%~0.229%)

* This interval is the 95% CI for 100 repeat simulations.

Supplementary Table 4.11 Comparison of "Double Archaic Population Scenarios" for non-AMH makers

Archaic Lineage	$T_{DenIntro}$ (kya)	$T_{NeanIntro}$ (kya)	α_{Den}	α_{Nean}	Precision	TPR	FPR
Denisovan&Neanderthal	30	30	0.4	1.6	99.6%(99.4%~99.7%)*	91.3%(86.6%~95.9%)	0.086%(0.058%~0.127%)
Denisovan&Neanderthal	30	30	0.8	1.2	99.6%(99.4%~99.7%)	93.0%(86.4%~96.5%)	0.083%(0.055%~0.120%)
Denisovan&Neanderthal	30	30	1.2	0.8	99.6%(99.5%~99.8%)	93.9%(89.6%~96.1%)	0.083%(0.055%~0.113%)
Denisovan&Neanderthal	30	30	1.6	0.4	99.6%(99.4%~99.7%)	94.8%(92.2%~96.5%)	0.085%(0.063%~0.140%)
Denisovan&Neanderthal	30	50	0.4	1.6	99.4%(99.2%~99.6%)	95.0%(92.3%~96.7%)	0.130%(0.093%~0.166%)
Denisovan&Neanderthal	30	50	0.8	1.2	99.5%(99.3%~99.6%)	95.4%(92.2%~96.6%)	0.118%(0.087%~0.154%)
Denisovan&Neanderthal	30	50	1.2	0.8	99.5%(99.3%~99.6%)	95.5%(92.6%~96.5%)	0.106%(0.074%~0.145%)
Denisovan&Neanderthal	30	50	1.6	0.4	99.6%(99.4%~99.7%)	95.9%(93.0%~96.6%)	0.096%(0.062%~0.153%)
Denisovan&Neanderthal	30	70	0.4	1.6	99.2%(99.0%~99.4%)	95.2%(92.3%~96.2%)	0.168%(0.125%~0.218%)
Denisovan&Neanderthal	30	70	0.8	1.2	99.3%(99.1%~99.5%)	95.5%(93.5%~96.4%)	0.151%(0.115%~0.204%)
Denisovan&Neanderthal	30	70	1.2	0.8	99.4%(99.2%~99.6%)	95.6%(94.2%~96.4%)	0.122%(0.097%~0.167%)
Denisovan&Neanderthal	30	70	1.6	0.4	99.5%(99.4%~99.7%)	95.8%(94.7%~96.6%)	0.103%(0.071%~0.137%)
Denisovan&Neanderthal	50	30	0.4	1.6	99.5%(99.4%~99.7%)	90.5%(84.7%~94.4%)	0.099%(0.073%~0.127%)
Denisovan&Neanderthal	50	30	0.8	1.2	99.5%(99.4%~99.7%)	91.3%(86.6%~95.2%)	0.099%(0.071%~0.129%)
Denisovan&Neanderthal	50	30	1.2	0.8	99.5%(99.3%~99.6%)	92.5%(89.0%~94.7%)	0.109%(0.084%~0.149%)
Denisovan&Neanderthal	50	30	1.6	0.4	99.4%(99.3%~99.6%)	93.0%(90.7%~94.4%)	0.125%(0.092%~0.164%)
Denisovan&Neanderthal	50	50	0.4	1.6	99.4%(99.2%~99.5%)	94.6%(91.7%~96.1%)	0.138%(0.105%~0.181%)
Denisovan&Neanderthal	50	50	0.8	1.2	99.4%(99.2%~99.5%)	94.3%(92.0%~95.6%)	0.132%(0.094%~0.164%)
Denisovan&Neanderthal	50	50	1.2	0.8	99.4%(99.2%~99.6%)	94.0%(92.2%~95.2%)	0.129%(0.089%~0.170%)
Denisovan&Neanderthal	50	50	1.6	0.4	99.4%(99.2%~99.5%)	93.5%(92.6%~94.9%)	0.130%(0.095%~0.179%)
Denisovan&Neanderthal	50	70	0.4	1.6	99.2%(99.0%~99.4%)	94.9%(93.0%~95.8%)	0.177%(0.142%~0.219%)
Denisovan&Neanderthal	50	70	0.8	1.2	99.3%(99.0%~99.4%)	94.5%(92.8%~95.3%)	0.159%(0.118%~0.206%)
Denisovan&Neanderthal	50	70	1.2	0.8	99.3%(99.1%~99.5%)	94.0%(92.6%~94.8%)	0.148%(0.108%~0.193%)
Denisovan&Neanderthal	50	70	1.6	0.4	99.4%(99.1%~99.5%)	93.8%(92.1%~94.6%)	0.137%(0.104%~0.198%)
Denisovan&Neanderthal	70	30	0.4	1.6	99.5%(99.3%~99.6%)	90.6%(84.1%~94.2%)	0.102%(0.075%~0.133%)

Archaic Lineage	$T_{DenIntro}$ (kya)	$T_{NeanIntro}$ (kya)	α_{Den}	α_{Nean}	Precision	TPR	FPR
Denisovan&Neanderthal	70	30	0.8	1.2	99.5%(99.3%~99.6%)	90.3%(84.7%~93.3%)	0.110%(0.082%~0.144%)
Denisovan&Neanderthal	70	30	1.2	0.8	99.4%(99.2%~99.5%)	90.8%(86.0%~93.0%)	0.132%(0.099%~0.173%)
Denisovan&Neanderthal	70	30	1.6	0.4	99.3%(99.1%~99.4%)	91.4%(87.6%~92.6%)	0.149%(0.111%~0.203%)
Denisovan&Neanderthal	70	50	0.4	1.6	99.3%(99.1%~99.5%)	94.1%(90.9%~95.7%)	0.143%(0.115%~0.186%)
Denisovan&Neanderthal	70	50	0.8	1.2	99.3%(99.1%~99.5%)	93.4%(89.9%~94.9%)	0.142%(0.108%~0.183%)
Denisovan&Neanderthal	70	50	1.2	0.8	99.3%(99.1%~99.4%)	92.7%(90.2%~93.8%)	0.156%(0.116%~0.203%)
Denisovan&Neanderthal	70	50	1.6	0.4	99.3%(99.1%~99.4%)	91.9%(89.9%~93.2%)	0.159%(0.119%~0.201%)
Denisovan&Neanderthal	70	70	0.4	1.6	99.2%(98.9%~99.3%)	94.5%(92.4%~95.5%)	0.183%(0.151%~0.227%)
Denisovan&Neanderthal	70	70	0.8	1.2	99.2%(99.0%~99.4%)	93.5%(90.4%~94.6%)	0.175%(0.131%~0.217%)
Denisovan&Neanderthal	70	70	1.2	0.8	99.2%(99.0%~99.3%)	92.8%(90.9%~93.6%)	0.174%(0.136%~0.220%)
Denisovan&Neanderthal	70	70	1.6	0.4	99.2%(99.0%~99.4%)	92.0%(90.2%~93.1%)	0.171%(0.131%~0.216%)

* This interval is the 95% CI for 100 repeat simulations.

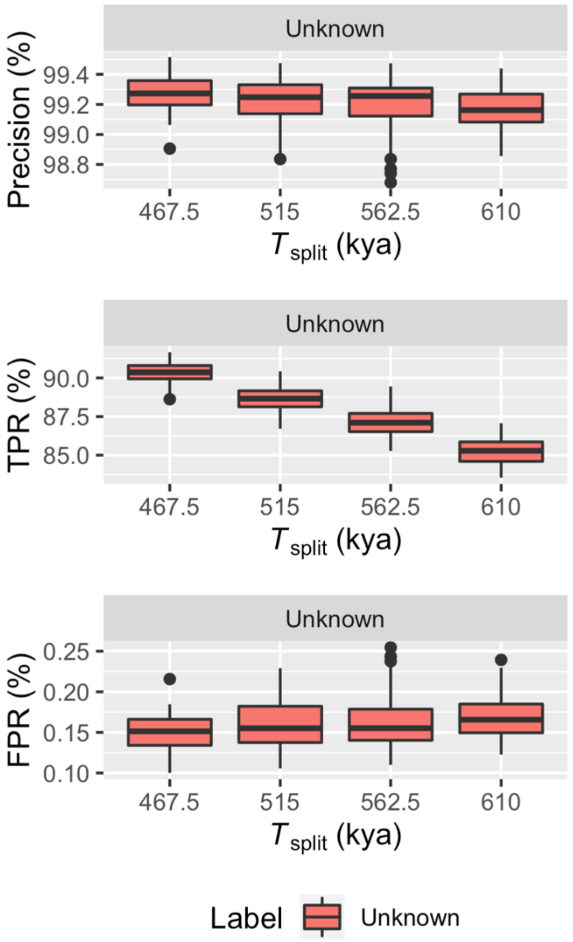

Supplementary Figure 4.19 Comparison of "Unknown Archaic Scenarios" for non-AMH markers. Comparison between non-AMH markers in the inferred introgressed segments and that in the ground truth segments under the scenarios of deep divergent unknown archaic lineage from the ancestor of Denisovan and Neanderthal. The x-axis represents the divergence time from the archaic lineage and values is in kya (thousand years ago). The y-axis represents the summary statistics of precision, TPR and FPR. 100 replicates were performed independently and 100 test individuals were analyzed within each replication. Bounds of box represent the interquartile range (IQR; 25th to 75th percentile), with a center line indicating the median. Whiskers are represented in the form of Tukey style.

Supplementary Table 4.12 Comparison of "Unknown Archaic Scenarios" for non-AMH markers

Archaic Lineage	T_{split} (kya)	Precision	TPR	FPR
Unknown	467.5	99.3%(99.1%~99.5%)*	90.4%(89.1%~91.5%)	0.152%(0.111%~0.183%)
Unknown	515.0	99.3%(99.0%~99.4%)	88.7%(87.2%~89.9%)	0.156%(0.117%~0.226%)
Unknown	562.5	99.3%(98.8%~99.4%)	87.1%(85.8%~88.6%)	0.155%(0.118%~0.238%)
Unknown	610.0	99.2%(98.9%~99.4%)	85.3%(83.6%~86.8%)	0.166%(0.128%~0.225%)

* This interval is the 95% CI for 100 repeat simulations.

4.1.3 Segment-Based Evaluation

To evaluate the proportion of the correctly inferred segments, we performed the segment-based evaluation. Firstly, we defined the correctly inferred segment is an inferred segment overlapped with the ground-truth segments more than 80% of the inferred segment length. The smaller segments trend to be subjected to random errors more, so we set a threshold to remove the shorter segments. We evaluated the proportion of the correctly inferred segments with different minimum length threshold (Supplementary Figure 4.20 ~ 4.25).

For all the simulation data, the proportion of correct inferred segments increasing as the minimum length threshold increased (Supplementary Figure 4.20 ~ 4.25).

Under scenarios of different divergence time to the archaic lineage, the divergence time T_{split} does not affect the results very much. Results of different T_{split} exhibit a similar pattern (Supplementary Figure 4.20).

Under scenarios of different introgression time, analysis with scenarios of more recent introgression time, show a relative higher correctly inferred proportion. When T_{intro} equals to 18 kya, more than 90% inferred segments are correct without any filtration. However, if the T_{intro} is 82 kya, the proportion drops to ~70% without any filtration (Supplementary Figure 4.21).

Under scenarios of different introgression proportions, our method shows a better performance in cases of larger introgression proportion. However, when α is larger than 2.0%, the patterns become similar (Supplementary Figure 4.22).

When we consider two-wave introgression, higher correctly inferred proportion with a longer minimum length threshold still the main tendency. If a scenario contains more genetic materials from the recent wave introgression, the results trend to be better (Supplementary Figure 4.23 ~ 4.24).

Under the unknown archaic scenarios, the pattern is similar with the results of “diverse divergence time scenarios”. The divergence time from the archaic lineage, not affect the results very much (Supplementary Figure 4.25).

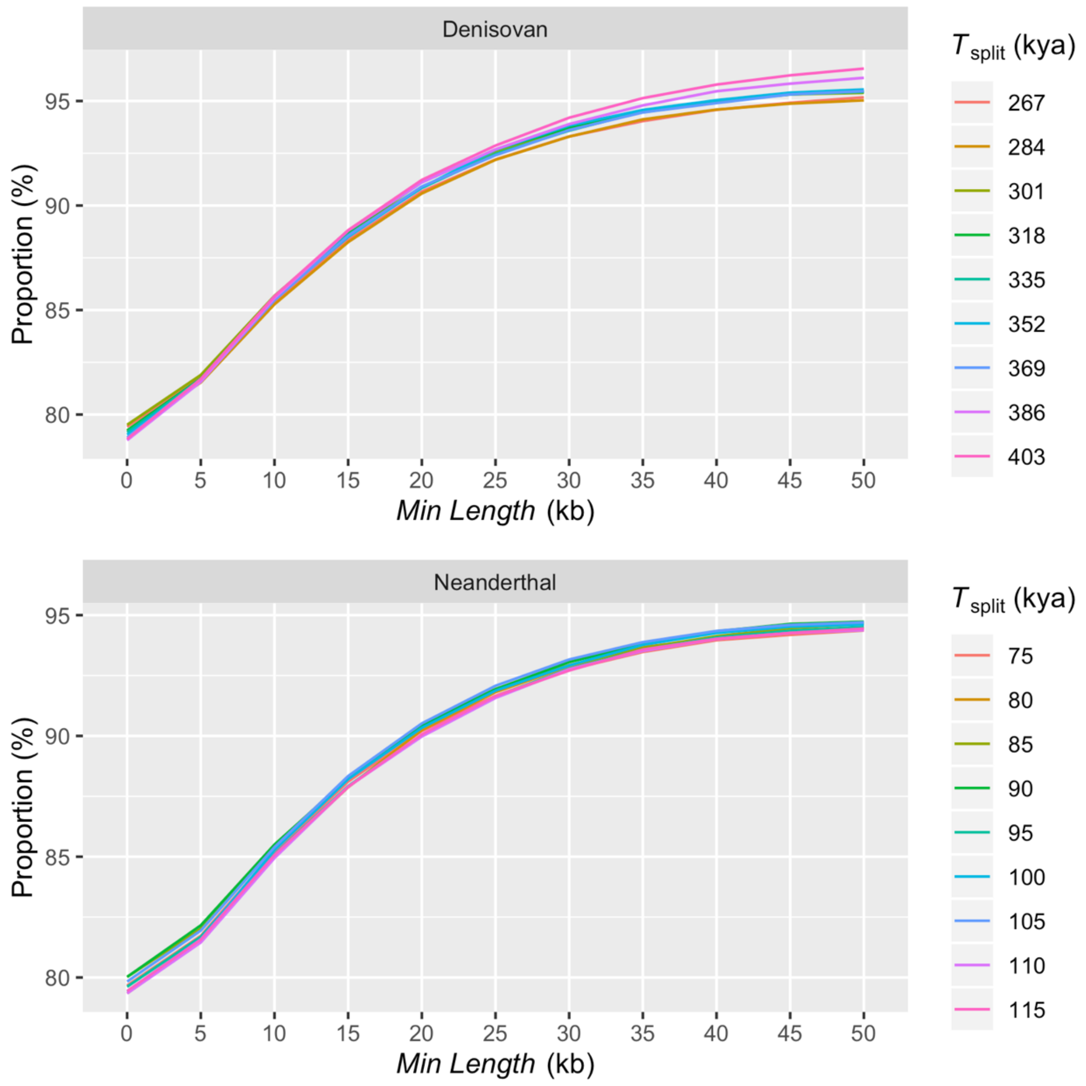

Supplementary Figure 4.20 Segment comparison of "Diverse Divergence Time Scenarios". The correctly inferred segment proportion distribution with different minimum length threshold under "Diverse Divergence Time Scenarios". Different lines stand for different divergence time from the archaic lineage. The x-axis represents the minimum threshold and the values are in kb. The y-axis represents the proportion of the correctly inferred segments in the segments longer than the minimum threshold.

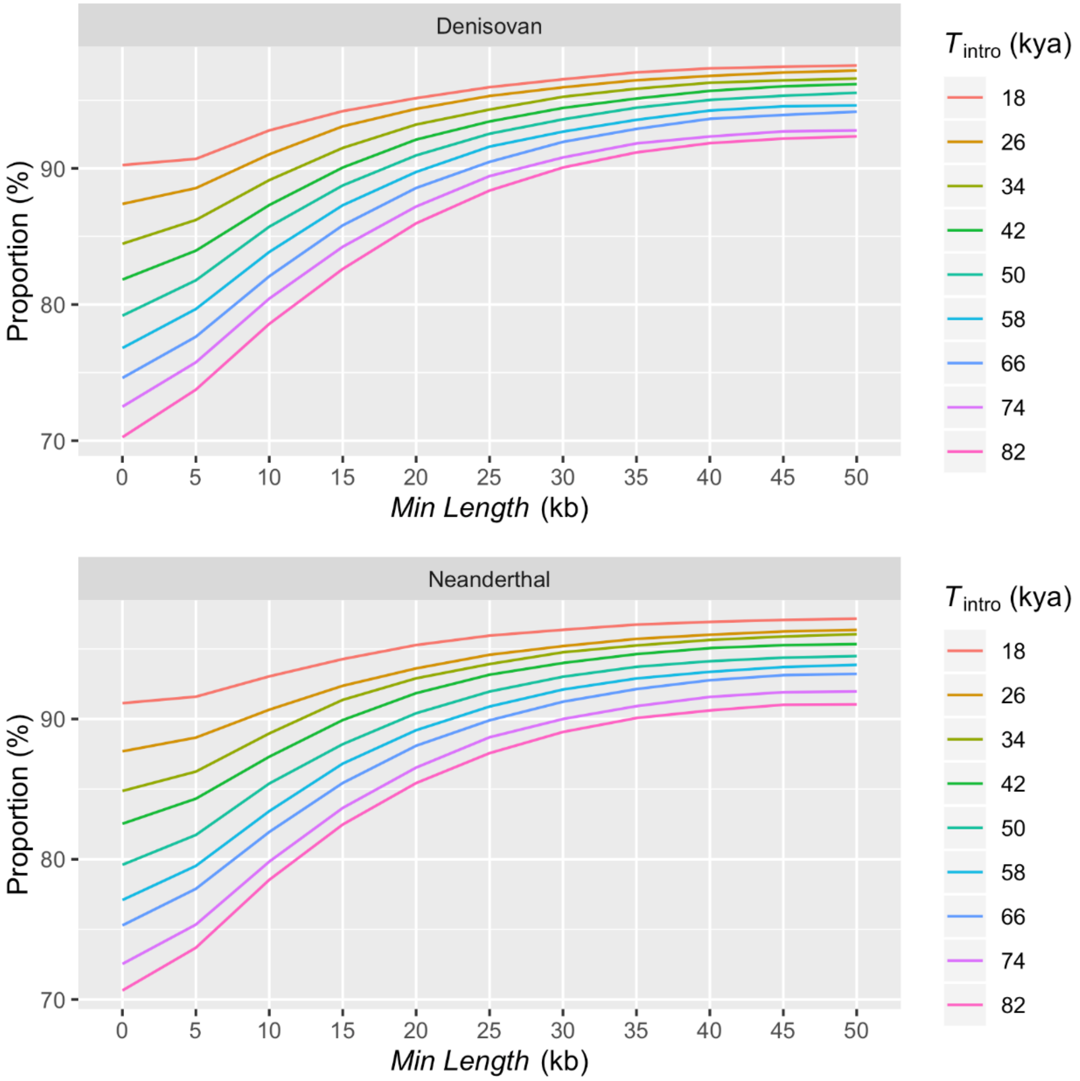

Supplementary Figure 4.21 Segment Comparison of "Diverse Introgression Time Scenarios". The correctly inferred segment proportion distribution with different minimum length threshold under "Diverse Introgression Time Scenarios". Different lines stand for different introgression time. The x-axis represents the minimum threshold and the values are in kb. The y-axis represents the proportion of the correctly inferred segments in the segments longer than the minimum threshold.

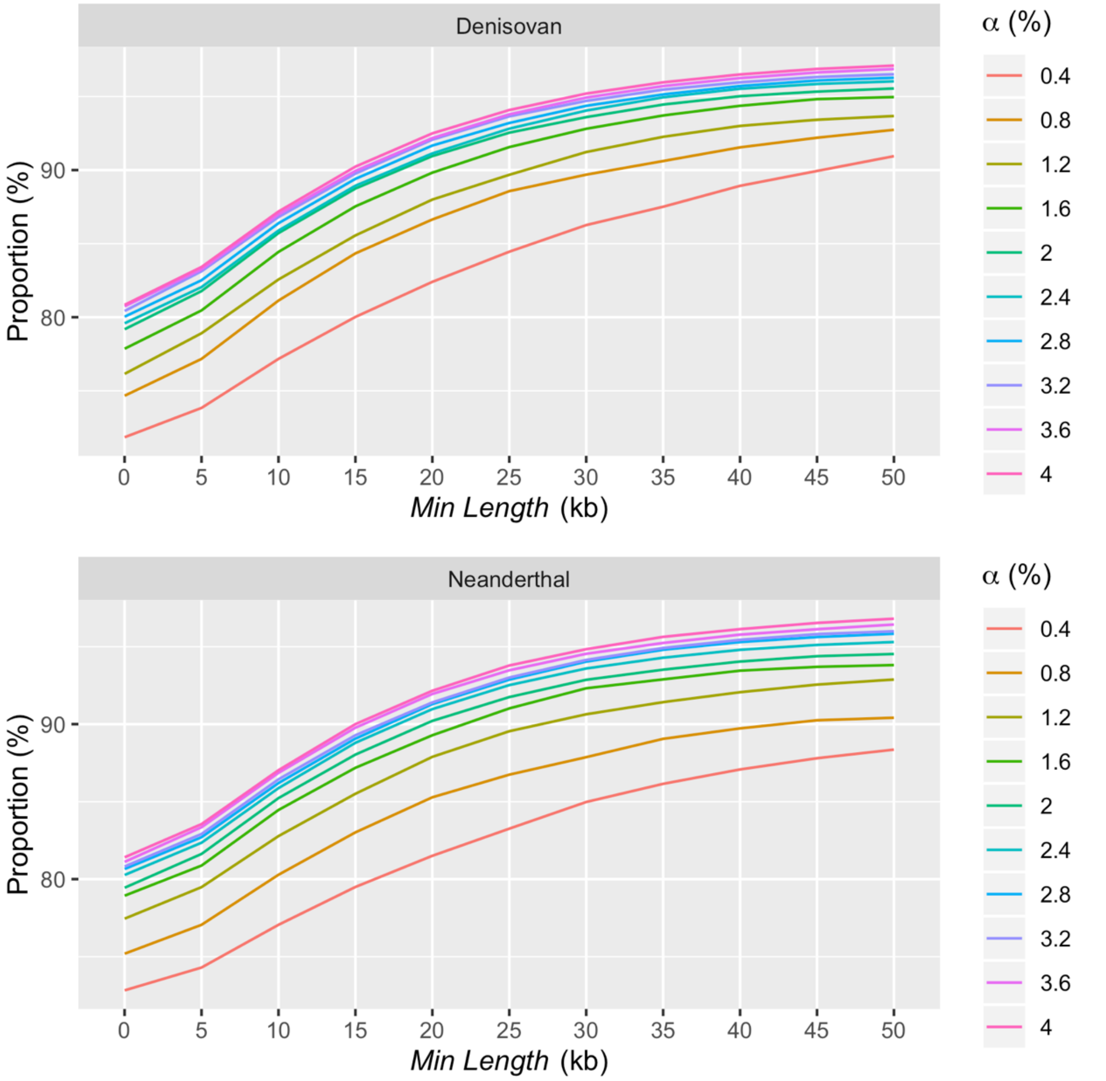

Supplementary Figure 4.22 Segment comparison of "Diverse Introgression Proportion Scenarios". The correctly inferred segment proportion distribution with different minimum length threshold under "Diverse Introgression Proportion Scenarios". Different lines stand for different introgression proportion. The x-axis represents the minimum threshold and the values are in kb. The y-axis represents the proportion of the correctly inferred segments in the segments longer than the minimum threshold.

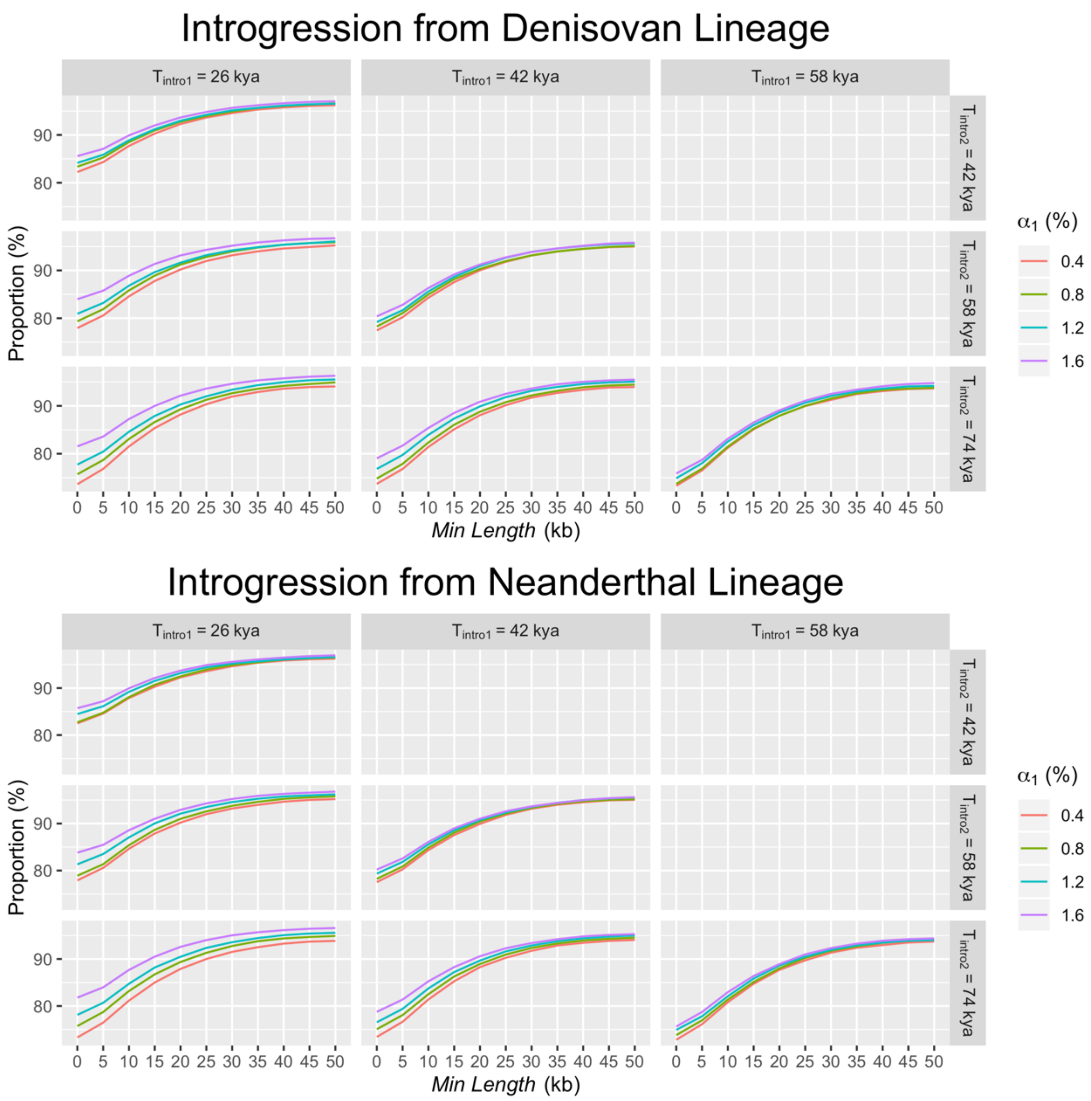

Supplementary Figure 4.23 Segment comparison of "Two Waves Introgression from Single Archaic Population Scenarios". The correctly inferred segment proportion distribution with different minimum length threshold under "Two Waves Introgression from Single Archaic Population Scenarios". Different lines stand for introgression proportion of the first wave introgression. The x-axis represents the minimum threshold and the values are in kb. The y-axis represents the proportion of the correctly inferred segments in the segments longer than the minimum threshold.

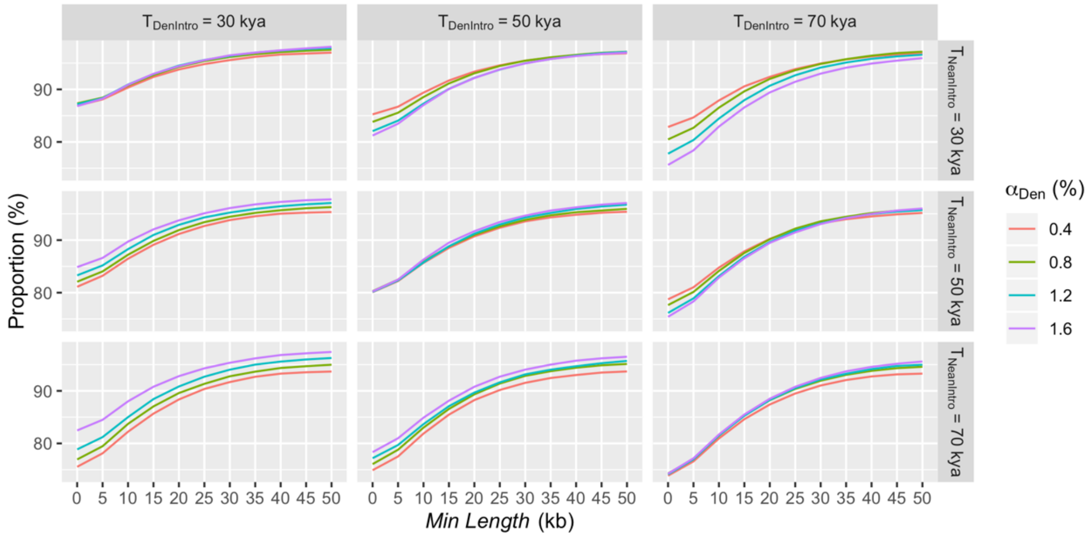

Supplementary Figure 4.24 Segment comparison of "Double Archaic Population Scenarios". The correctly inferred segment proportion distribution with different minimum length threshold under "Double Archaic Population Scenarios". Different lines stand for the introgression proportion from Denisovan lineage. The x-axis represents the minimum threshold and the values are in kb. The y-axis represents the proportion of the correctly inferred segments in the segments longer than the minimum threshold.

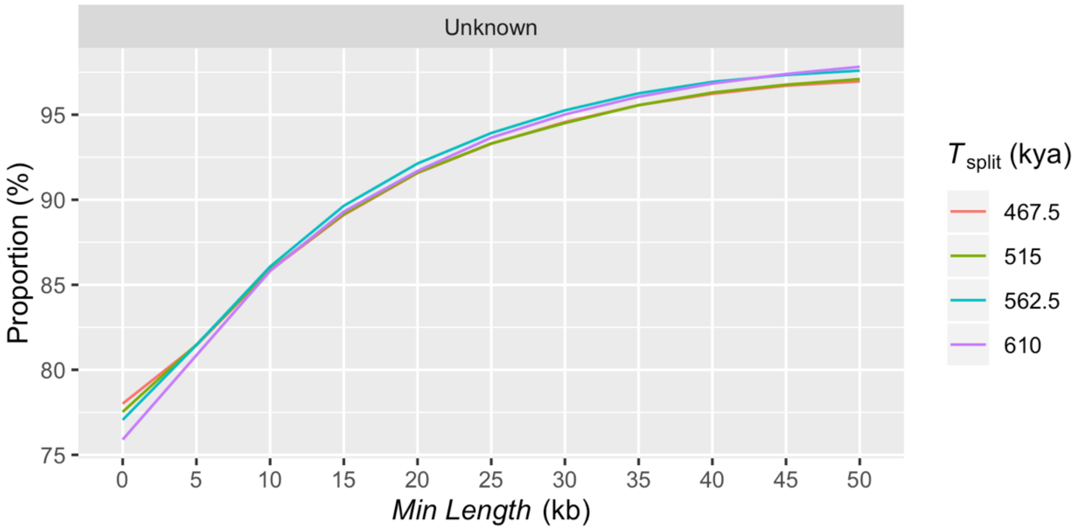

Supplementary Figure 4.25 Segment comparison of "Unknown Archaic Scenarios". The correctly inferred segment proportion distribution with different minimum length threshold under "Unknown Archaic Scenarios". Different lines stand for different divergence time from the archaic lineage. The x-axis represents the minimum threshold and the values are in kb. The y-axis represents

the proportion of the correctly inferred segments in the segments longer than the minimum threshold.

4.1.4 Matching Algorithm Evaluation

We also perform an analysis to evaluate the accuracy of the matching algorithm. By comparing the real ancestry and the inferred ancestry under different simulation scenarios, we evaluate the performance of our method under different demographic parameters.

For all these simulations, we use an identical matching model (0)

"((Africans:100,Non-Africans:100):557.5,(Denisovan:340,Neanderthal:350):237.5);"

In this model, there are four leaf nodes, which correspond to four different populations, and three internal nodes. Here, we use the format "*A_B*" stands for the ancestral node of A and B. For example, *Denisovan_Neanderthal* stands for the ancestor of Denisovan and Neanderthal. In total, seven possible introgressed nodes could be matched, *Denisovan*, *Neanderthal*, *Africans*, *non-Africans*, *Denisovan_Neanderthal* (*Den_Nean*), *Africans_non_Africans* (*Afr_nonAfr*) and *Africans_non-Africans_Denisovan_Neanderthal* (*Afr_nonAfr_Den_Nean*) (0).

We firstly evaluate the influence of different divergence time. Under the scenarios of diverse divergence time (Supplementary Note 3.1.1), the divergence time affects the accuracy of matching algorithm differently for two archaic lineages. For Neanderthal introgressed scenarios, the divergence time not affect the results very much and almost all the inferred segments matched to the correct ancestry (Supplementary Figure 4.26). For Denisovan introgressed scenarios, as the increasing of divergence time, more and more Denisovan derived segments matched to Den_Nean and Neanderthal (Supplementary Figure 4.26). The reason of the differences is the possible divergence time interval of Denisovan is much more ancient than that of Neanderthal. When divergence time equals to 403 kya, it is much closer to the divergence time between Denisovan and Neanderthal, which is 420 kya. The possibility of incomplete lineage sorting (ILS) among the introgressed lineage, Denisovan and Neanderthal increased when the divergence time is very ancient.

Different introgression time also influence the performance of matching algorithm.

The effect is a little bit weak than the divergence time (Supplementary Figure 4.27). For Denisovan introgressed scenarios, around 5%~20% segments might mismatch from Denisovan to Den_Nean. As the increasing of the introgression time, the mismatch proportion increased. Still the Neanderthal introgression scenarios not affected by the different introgression time and almost all inferred segments matched correctly (Supplementary Figure 4.27).

Introgression proportion will also affect the matching accuracy. When the introgression proportion is extreme low, for example, less than 0.8%, the accuracy of matching algorithm may lower, especially for 0.4% introgression proportion. The effect is stronger for Denisovan introgression scenarios (Supplementary Figure 4.28).

When we allowed two waves introgression from one archaic lineage, no matter the introgression time and proportion, there are around 20% segments will mismatch to Den_Nean (Supplementary Figure 4.29 ~ 4.30). If the two waves introgression from different archaic lineages, Neanderthal performed very good results, while ~10% Denisovan segments will mismatch to Neanderthal and ~10% mismatch to Den_Nean (Supplementary Figure 4.31).

The performance is barely satisfactory, when matching algorithm applied to the unknown archaic scenarios. Around half segments will match to the real ancestry Den_Nean and one quarter to Denisovan, one quarter to Neanderthal. As the increase of the divergence time, more segments will match to Afr_noAfr_Den_Nean, the ancestor of all the linages (Supplementary Figure 4.32).

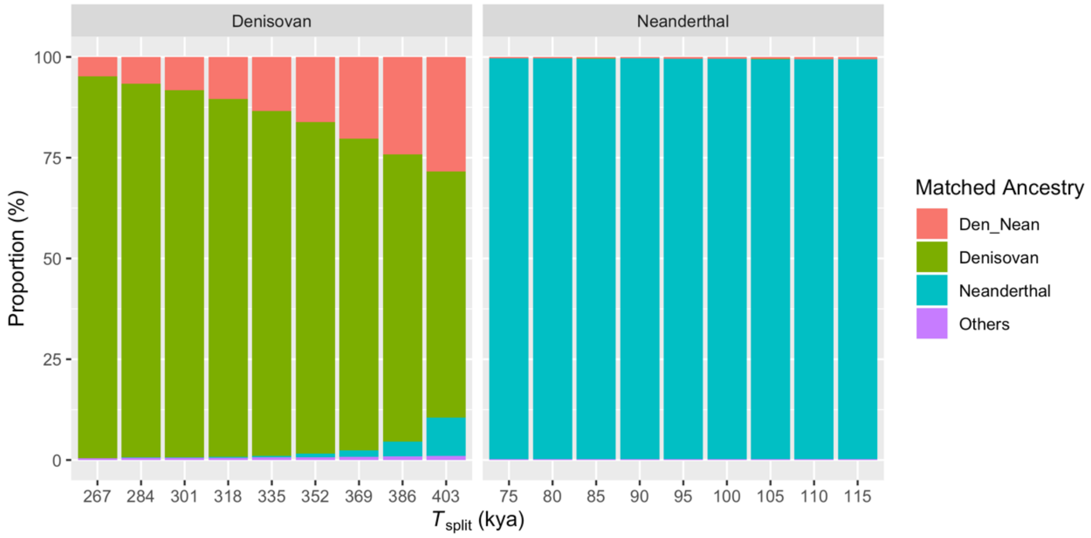

Supplementary Figure 4.26 Matching algorithm evaluation with "Diverse Divergence Time Scenarios". Different colors stand for different matched ancestries. "Den_Nean" stands for the ancestor of Denisovan and Neanderthal; "Others" include all other possible ancestry in the matching model. The label above each figure represents the true introgressed lineage. The x-axis indicates the different divergence from the introgressed lineage.

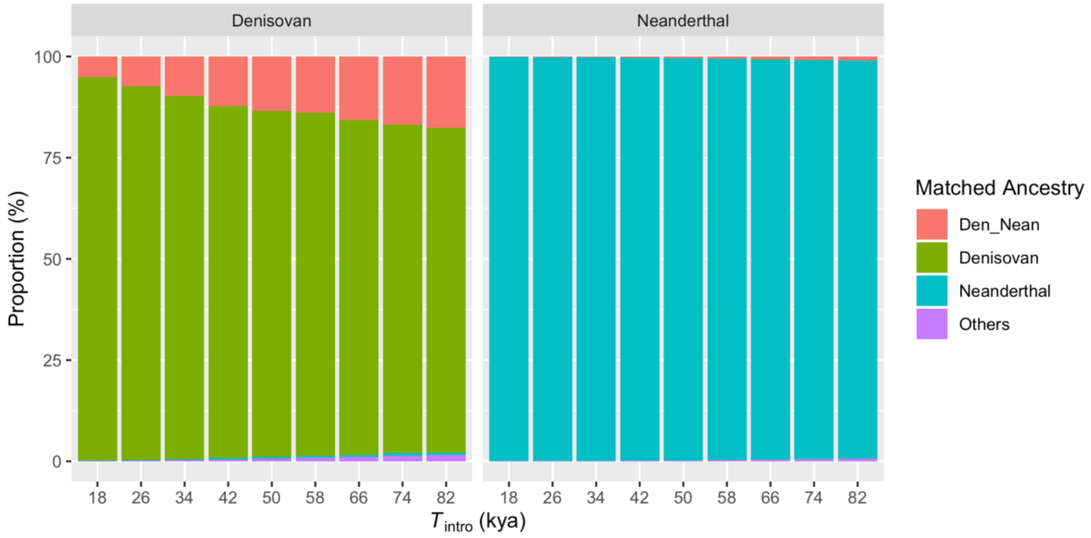

Supplementary Figure 4.27 Matching algorithm evaluation with "Diverse introgression Time Scenarios". Different colors stand for different matched ancestries. "Den_Nean" stands for the ancestor of Denisovan and Neanderthal; "Others" include all other possible ancestry in the matching model. The label above each figure represents the true introgressed lineage. The x-axis indicates the different introgression time.

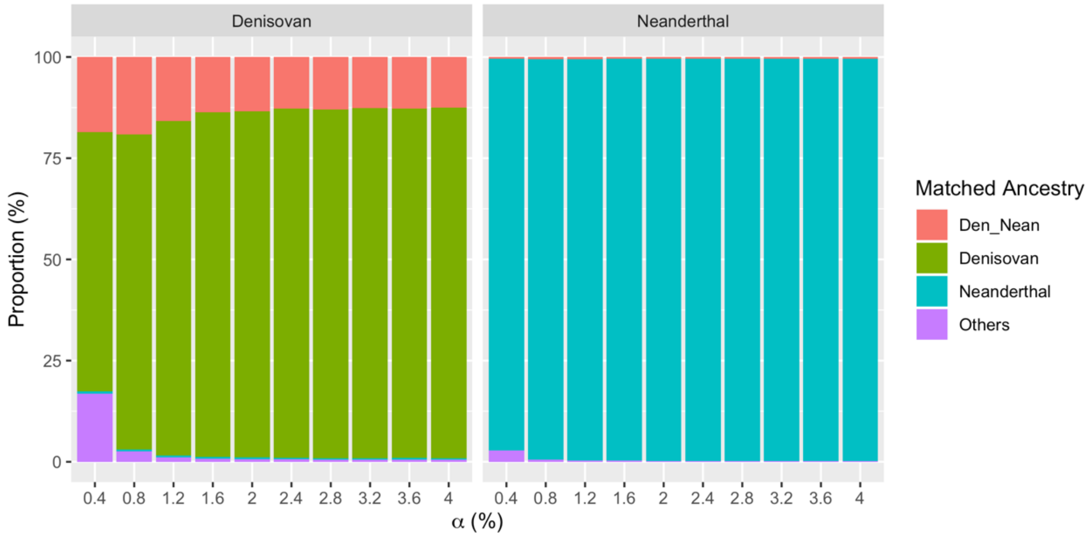

Supplementary Figure 4.28 Matching algorithm evaluation with "Diverse introgression Proportion Scenarios". Different colors stand for different matched ancestries. "Den_Nean" stands for the ancestor of Denisovan and Neanderthal; "Others" include all other possible ancestry in the matching model. The label above each figure represents the true introgressed lineage. The x-axis indicates the different introgression proportion.

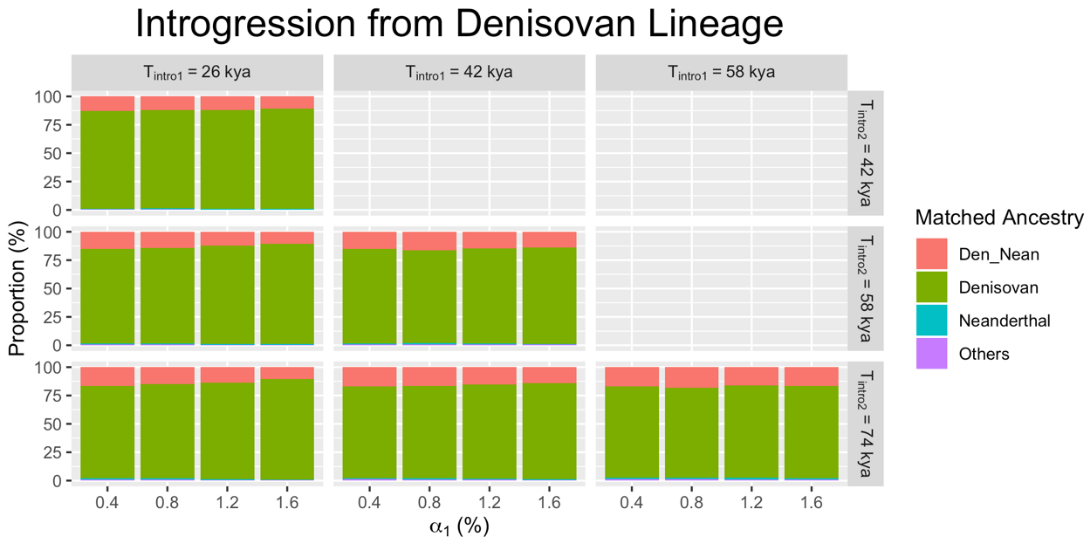

Supplementary Figure 4.29 Matching algorithm evaluation with "Two Waves Introgression from Denisovan Scenarios". Different colors stand for different matched ancestries. "Den_Nean" stands for the ancestor of Denisovan and Neanderthal; "Others" include all other possible ancestry in the matching model. The true introgressed lineage is Denisovan. The combination of top label and right label is the two waves' introgression time. The x-axis indicates the different introgression proportion of the recent wave of introgression.

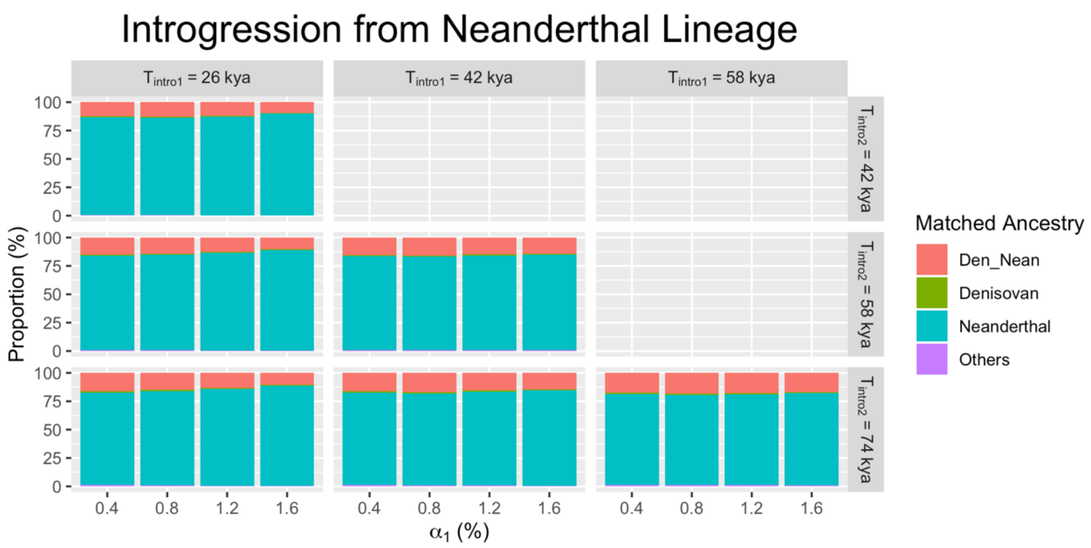

Supplementary Figure 4.30 Matching algorithm evaluation with "Two Waves Introgression from Neanderthal Scenarios". Different colors stand for different matched ancestries. "Den_Nean" stands for the ancestor of Denisovan and Neanderthal; "Others" include all other possible ancestry in the matching model. The true introgressed lineage is Neanderthal. The combination of top label and right label is the two waves' introgression time. The x-axis indicates the different introgression proportion of the recent wave of introgression.

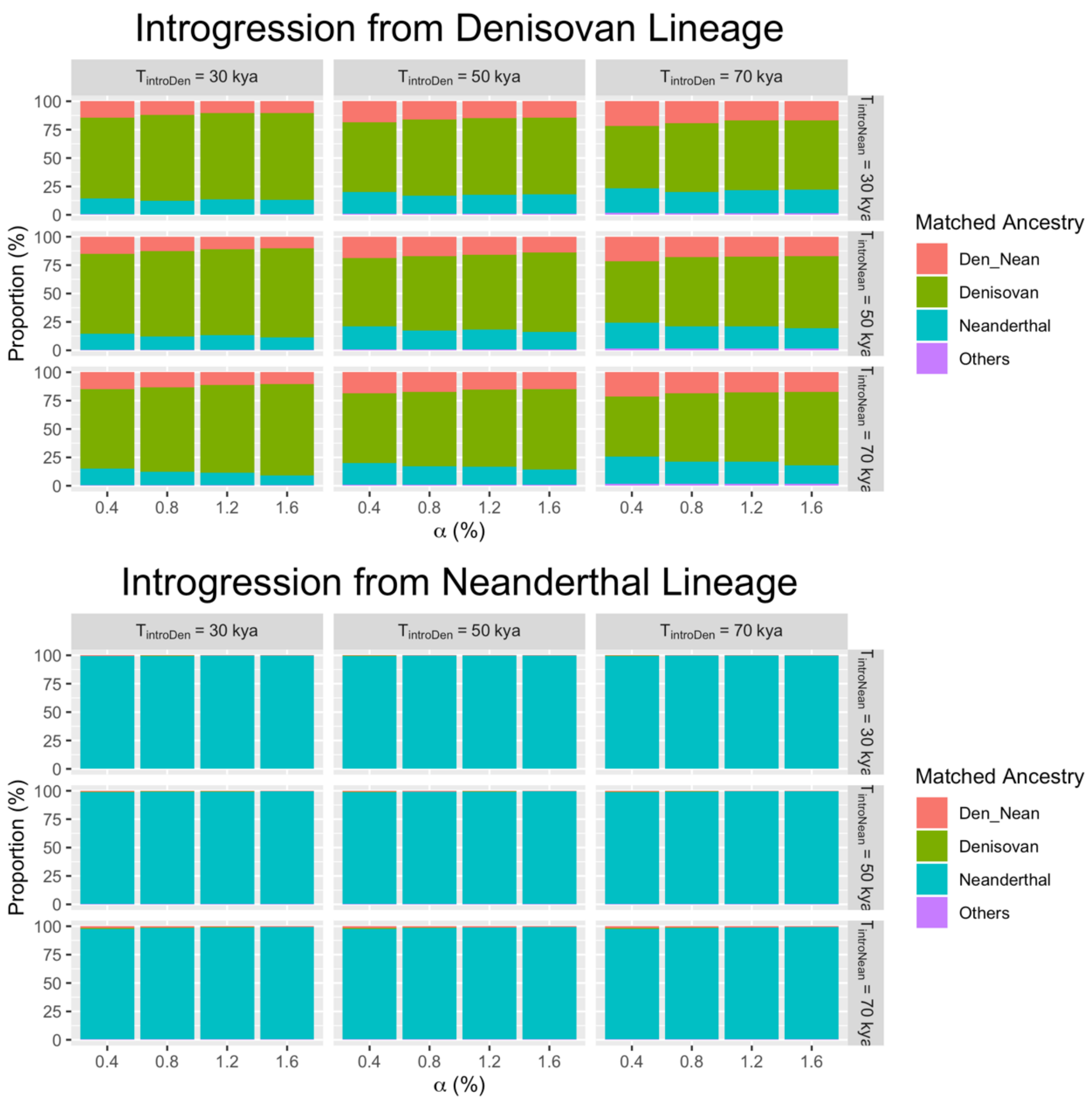

Supplementary Figure 4.31 Matching algorithm evaluation with "Double Archaic Population Scenarios". Different colors stand for different matched ancestries. "Den_Nean" stands for the ancestor of Denisovan and Neanderthal; "Others" include all other possible ancestry in the matching model. The true introgressed lineage of top figure is Denisovan and the bottom is Neanderthal. The combination of top label and right label of each figure is the two waves' introgression time. The x-axis indicates the different introgression proportion of the archaic lineage.

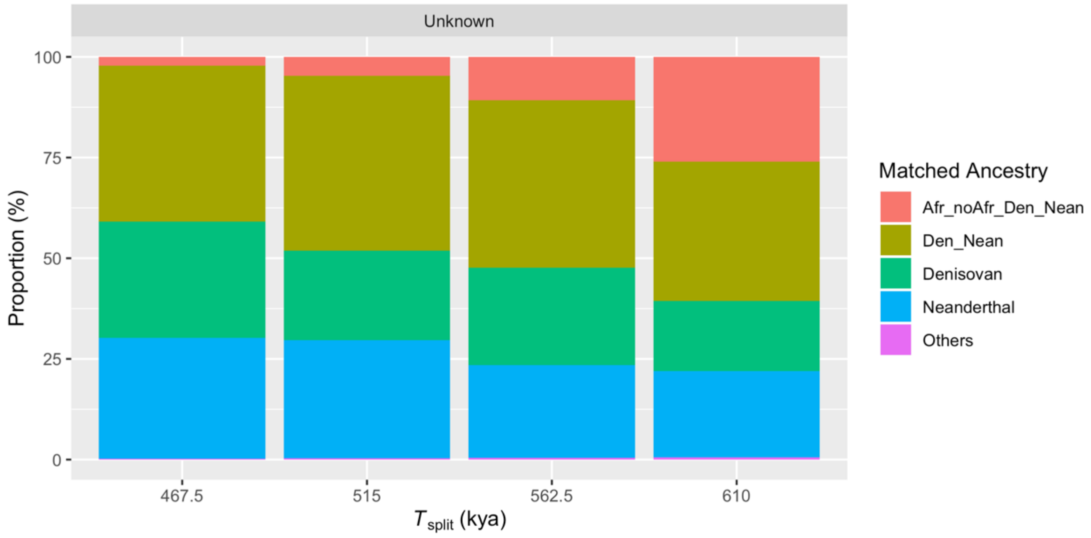

Supplementary Figure 4.32 Matching algorithm evaluation with "Unknown Archaic Scenarios". Different colors stand for different matched ancestries. "Afr_noAfr_Den_Nean" stands for the ancestor of modern human and archaic hominins; "Den_Nean" stands for the ancestor of Denisovan and Neanderthal; "Others" include all other possible ancestry in the matching model. The true ancestry of these scenarios is a deep divergence from the ancestor of Denisovan and Neanderthal. The x-axis indicates the different divergence time from the archaic linage.

4.2 Introgression History Inference

We also tested the performance of introgression history inference with simulation data. Since this method depends on the length distribution of archaic segments and the number of archaic segments in one simulation is not sufficient to get a good approximation of the distribution, we randomly selected 90 repeats results from the 100 repeats for each simulation parameter set and merged the archaic segments of the 90 repeats together. The total length test non-African genome of 90 repeats is $90 \times 200 \times 10 = 1.8 \times 10^5$ Mb. That is about the genome length of 30 human individuals (diploid). To increase the stability of our method, we removed segments shorter than 0.015 cM (15kb) (Supplementary Note 4.1.3). For each run, we did 100 times bootstrapping.

4.2.1 Segment Length Distribution

The introgression history inference relies on the accuracy of the distribution of inferred segment length. Before evaluating the performance of history inference, we compare the distribution of inferred segment length with the distribution of ground-truth segment length.

We removed the segment length shorter than 0.015 cM (15kb) and plotted the two distributions under simulation scenarios (Supplementary Figure 4.33 ~ 4.43). We found that for all the scenarios, the distribution of inferred segments and that of the ground-truth segments was almost identical. The accurate inference of segment length distribution makes the following introgression history inference possible.

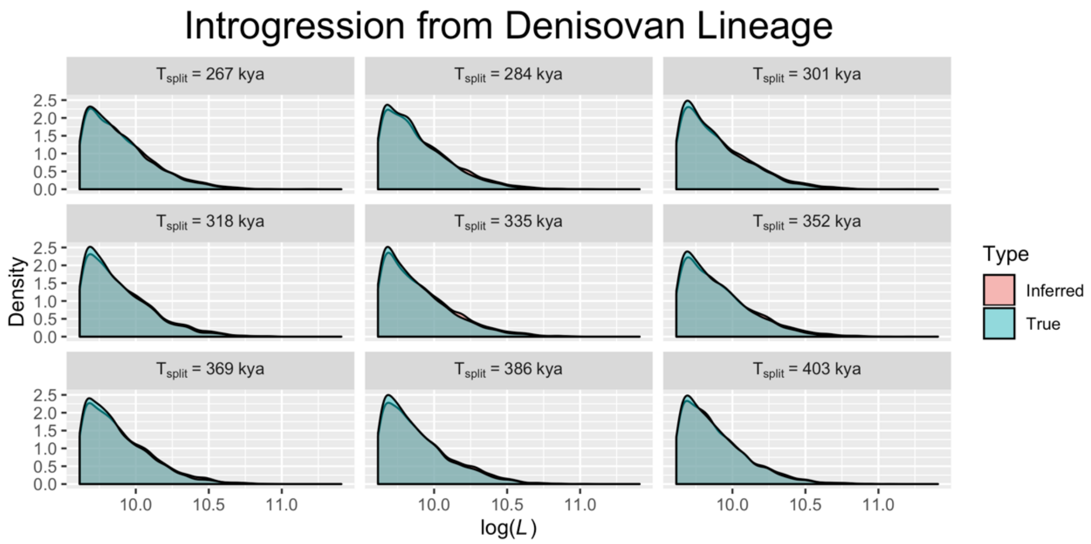

Supplementary Figure 4.33 Introgressed segment length distribution of Denisovan lineage under "Diverse Divergence Time Scenarios". "Inferred" stands for the *ArchaicSeeker 2.0* inferred introgressed segments and "True" stands for the ground truth introgressed segments. The segment length (L) is in unit of bp and we take the natural logarithm of the length. Different sub-figures stand for scenarios of different divergence time.

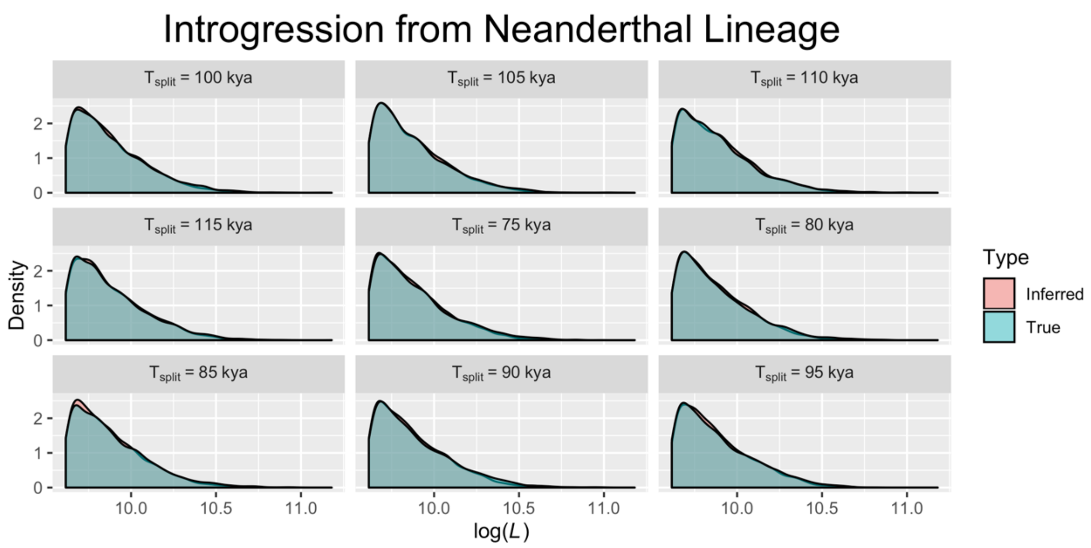

Supplementary Figure 4.34 Introgressed segment length distribution of Neanderthal lineage under "Diverse Divergence Time Scenarios". "Inferred" stands for the *ArchaicSeeker 2.0* inferred introgressed segments and "True" stands for the ground truth introgressed segments. The segment length (L) is in unit of bp and we take the natural logarithm of the length. Different sub-figures stand for scenarios of different divergence time.

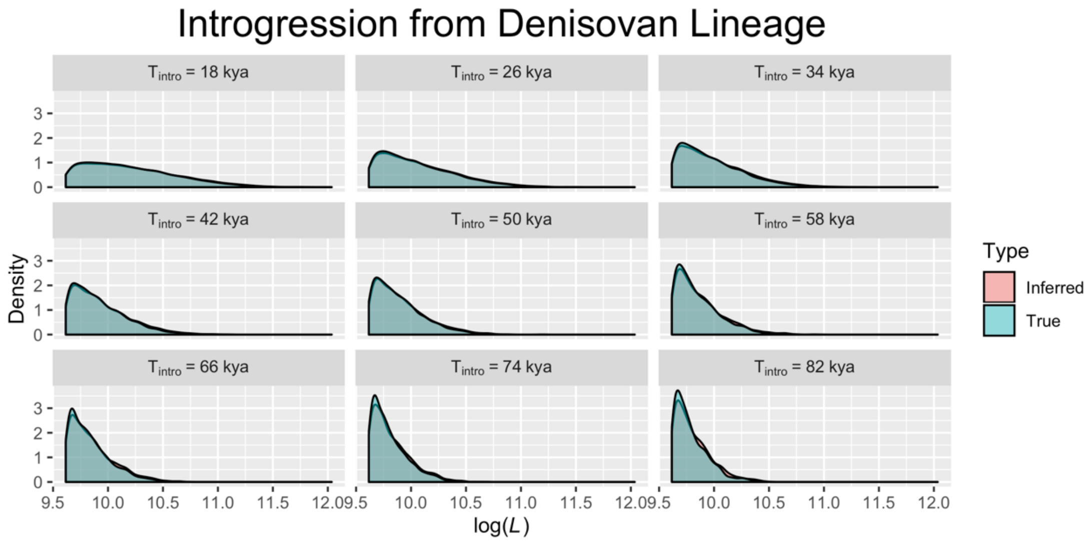

Supplementary Figure 4.35 Introgressed segment length distribution of Denisovan lineage under "Diverse Introgression Time Scenarios". "Inferred" stands for the *ArchaicSeeker 2.0* inferred introgressed segments and "True" stands for the ground truth introgressed segments. The segment length (L) is in unit of bp and we take the natural logarithm of the length. Different sub-figures stand for scenarios of different introgression time.

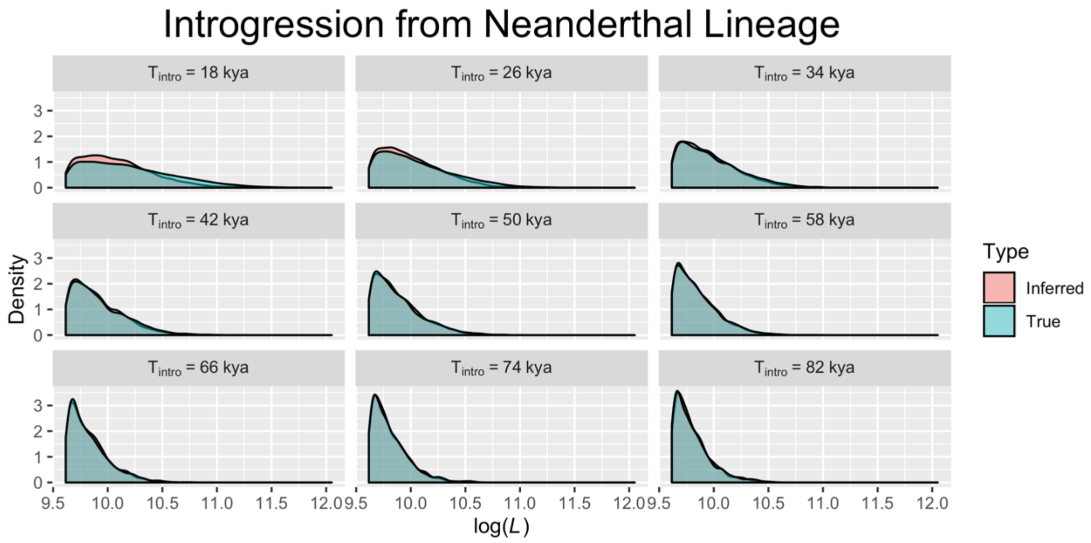

Supplementary Figure 4.36 Introgressed segment length distribution of Neanderthal lineage under "Diverse Introgression Time Scenarios". "Inferred" stands for the *ArchaicSeeker 2.0* inferred introgressed segments and "True" stands for the ground truth introgressed segments. The segment length (L) is in unit of bp and we take the natural logarithm of the length. Different sub-figures stand for scenarios of different introgression time.

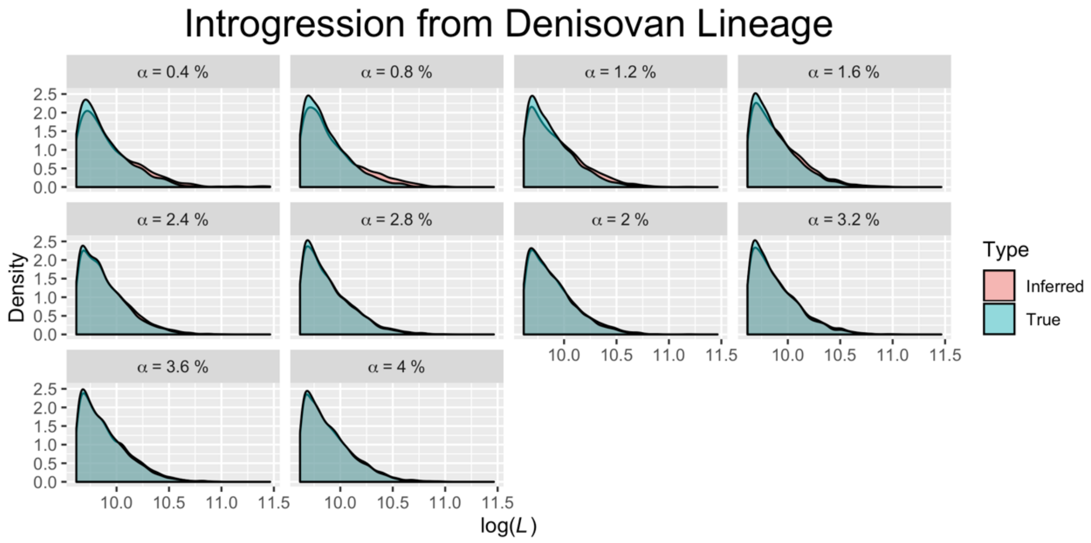

Supplementary Figure 4.37 Introgressed segment length distribution of Denisovan lineage under "Diverse Introgression Proportion Scenarios". "Inferred" stands for the *ArchaicSeeker 2.0* inferred introgressed segments and "True" stands for the ground truth introgressed segments. The segment length (L) is in unit of bp and we take the natural logarithm of the length. Different sub-figures stand for scenarios of different introgression proportion.

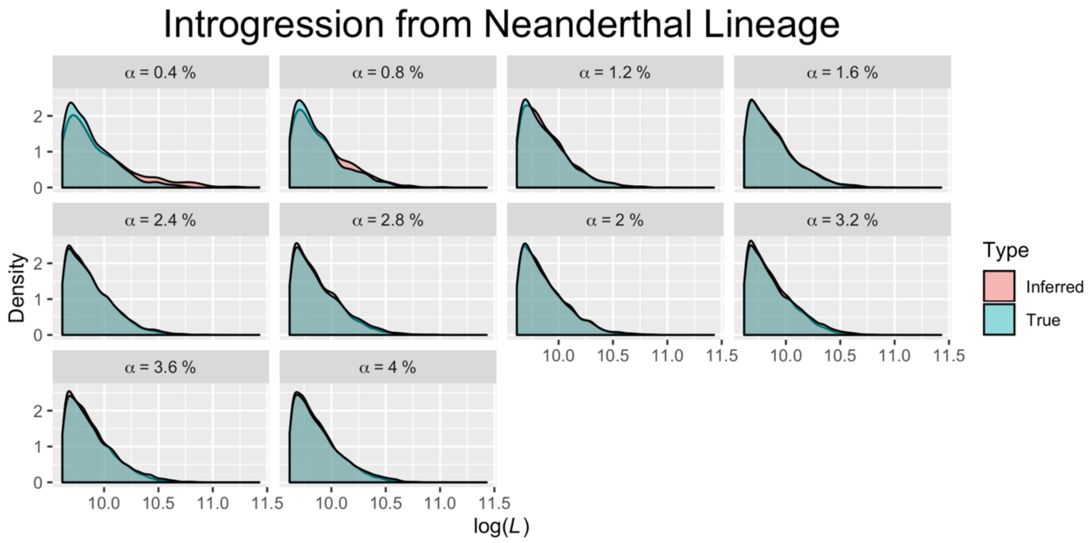

Supplementary Figure 4.38 Introgressed segment length distribution of Neanderthal lineage under "Diverse Introgression Proportion Scenarios". "Inferred" stands for the *ArchaicSeeker 2.0* inferred introgressed segments and "True" stands for the ground truth introgressed segments. The segment length (L) is in unit of bp and we take the natural logarithm of the length. Different sub-figures stand for scenarios of different introgression proportion.

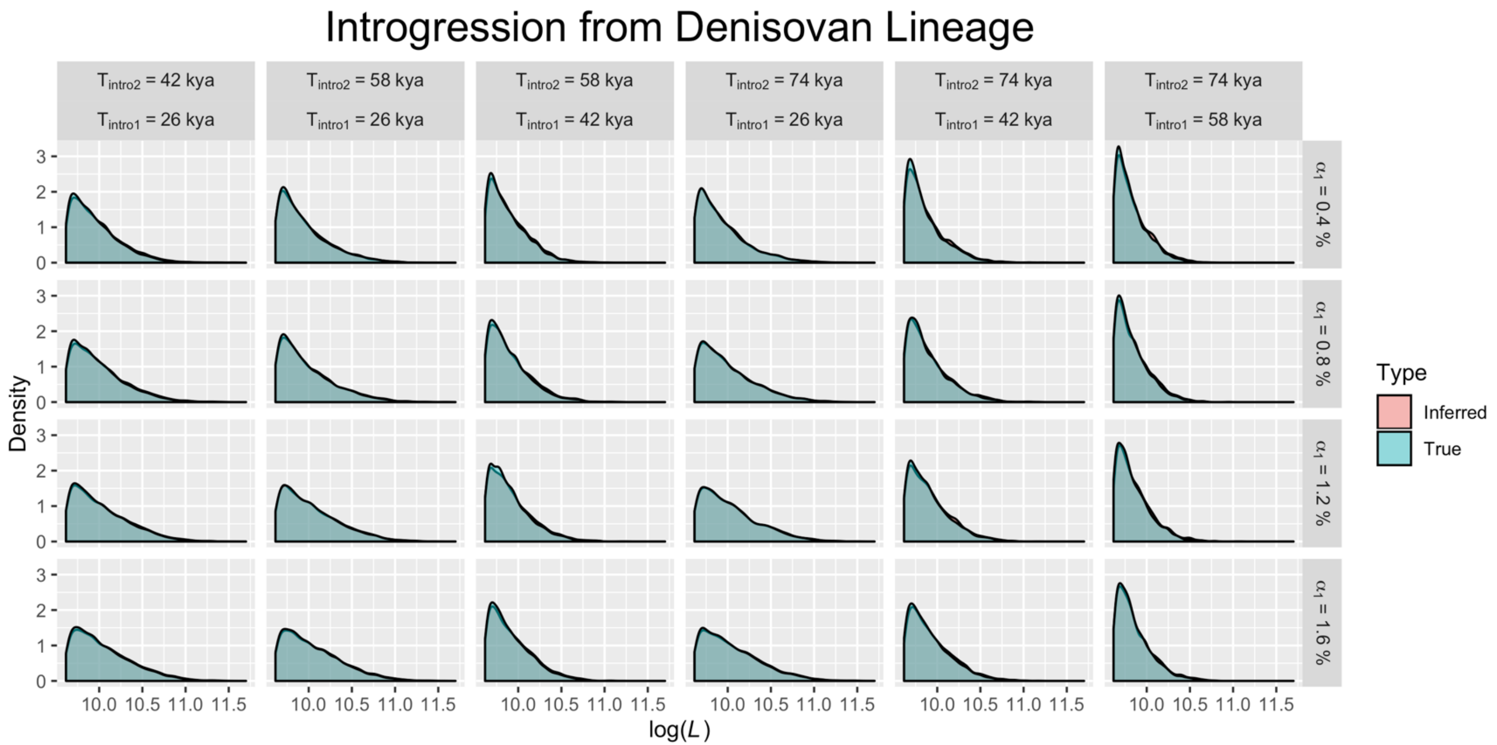

Supplementary Figure 4.39 Introgressed segment length distribution of Denisovan lineage under "Two Waves Introgression from Single Archaic Population Scenarios". "Inferred" stands for the *ArchaicSeeker 2.0* inferred introgressed segments and "True" stands for the ground truth introgressed segments. The segment length (L) is in unit of bp and we take the natural logarithm of the length. Different sub-figures stand for scenarios of different introgression time and introgression proportion combination.

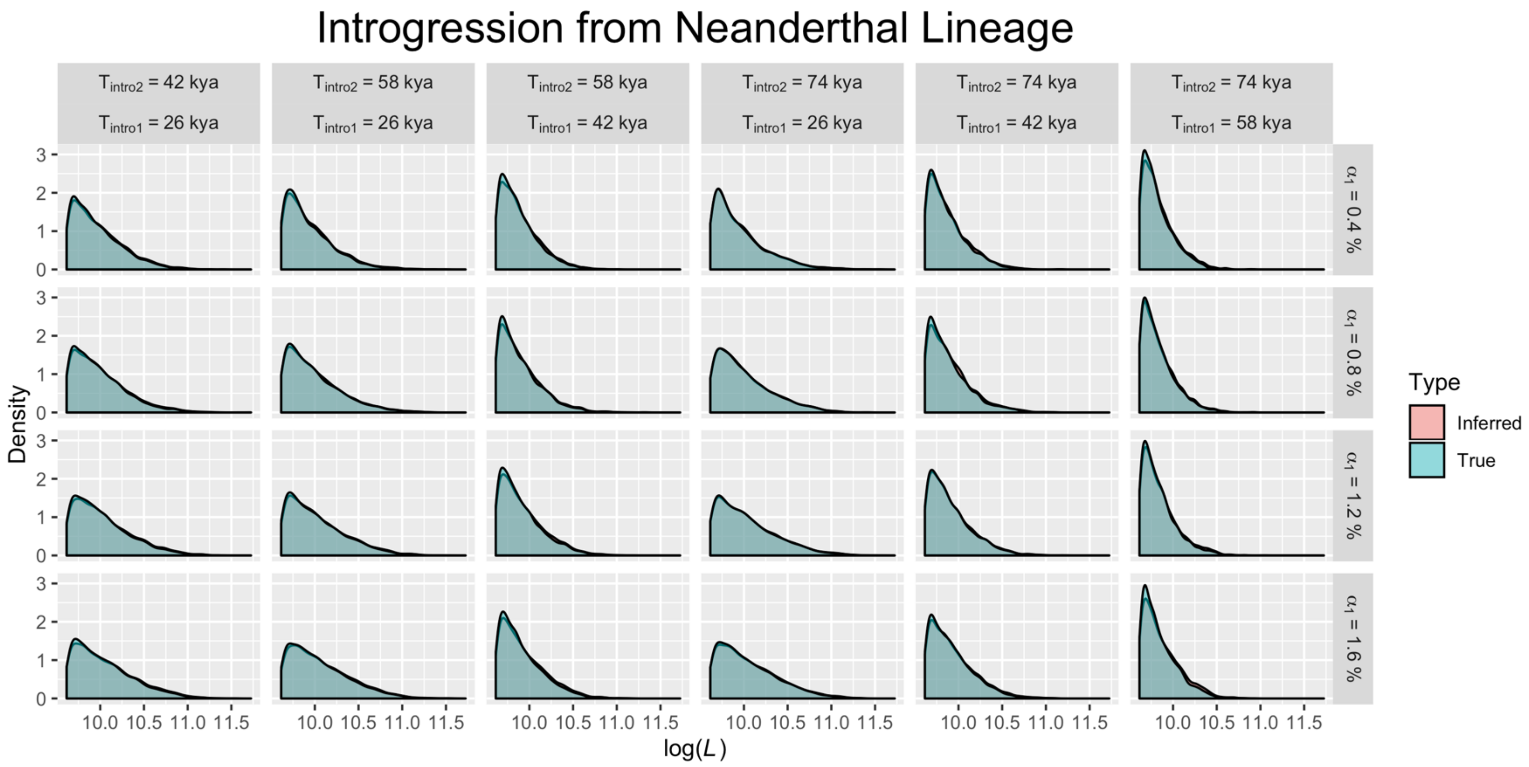

Supplementary Figure 4.40 Introgressed segment length distribution of Neanderthal lineage under "Two Waves Introgression from Single Archaic Population Scenarios". "Inferred" stands for the *ArchaicSeeker 2.0* inferred introgressed segments and "True" stands for the ground truth introgressed segments. The segment length (L) is in unit of bp and we take the natural logarithm of the length. Different sub-figures stand for scenarios of different introgression time and introgression proportion combination.

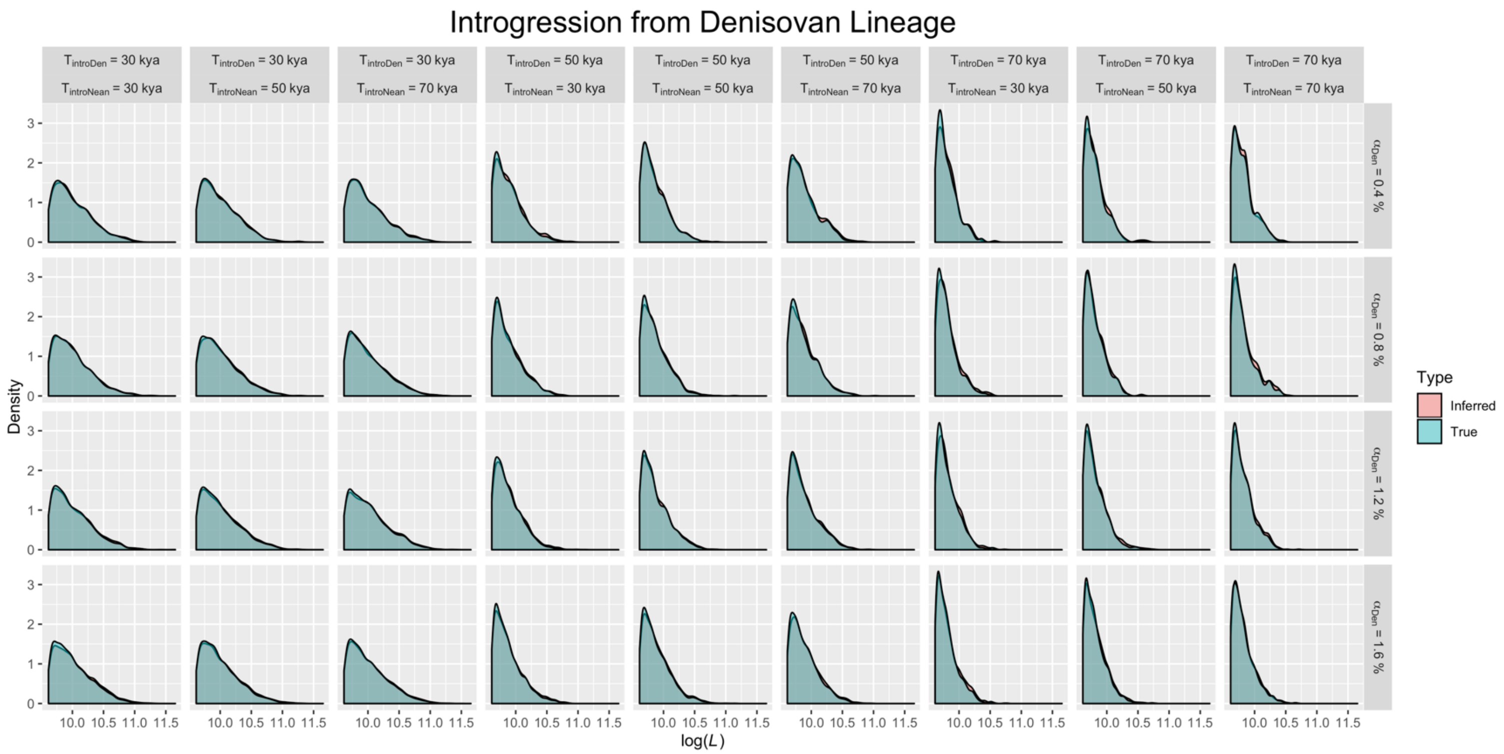

Supplementary Figure 4.41 Introgressed segment length distribution of Denisovan lineage under "Double Archaic Population Scenarios". "Inferred" stands for the *ArchaicSeeker 2.0* inferred introgressed segments and "True" stands for the ground truth introgressed segments. The segment length (L) is in unit of bp and we take the natural logarithm of the length. Different sub-figures stand for scenarios of different introgression time and introgression proportion combination.

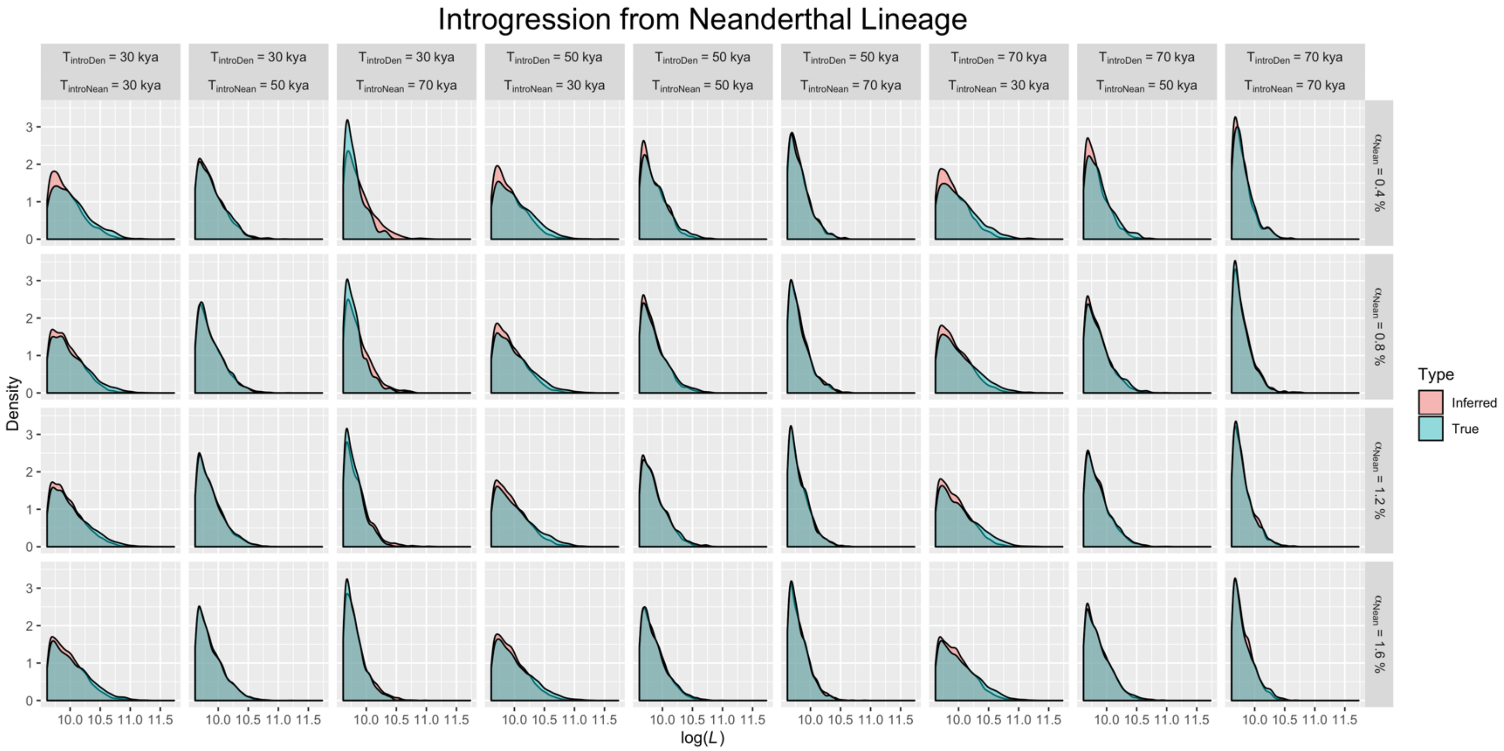

Supplementary Figure 4.42 Introgressed segment length distribution of Neanderthal lineage under "Double Archaic Population Scenarios". "Inferred" stands for the *ArchaicSeeker 2.0* inferred introgressed segments and "True" stands for the ground truth introgressed segments. The segment length (L) is in unit of bp and we take the natural logarithm of the length. Different sub-figures stand for scenarios of different introgression time and introgression proportion combination.

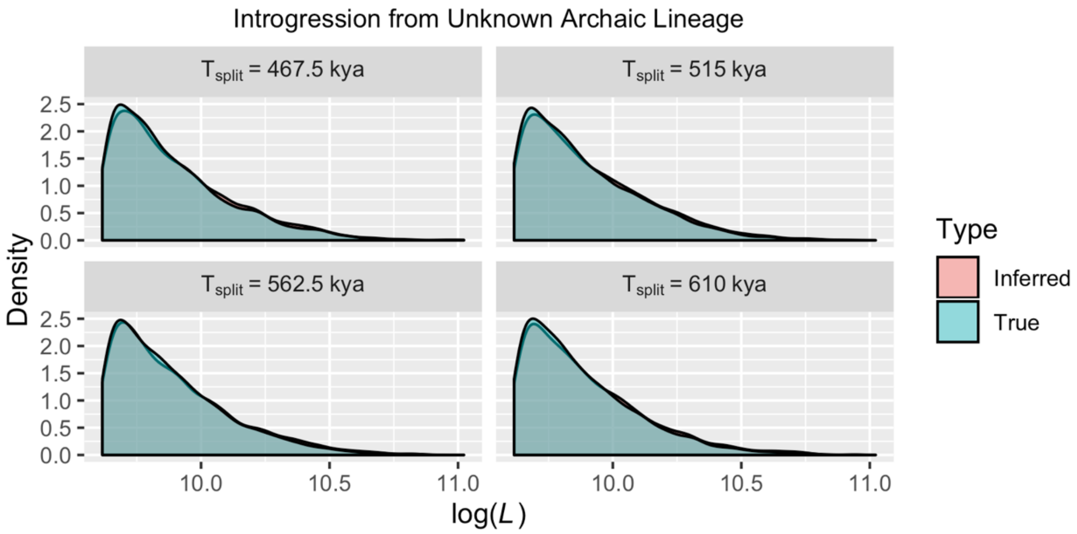

Supplementary Figure 4.43 Introgressed segment length distribution under "Unknown Archaic Scenarios". "Inferred" stands for the *ArchaicSeeker 2.0* inferred introgressed segments and "True" stands for the ground truth introgressed segments. The segment length (L) is in unit of bp and we take the natural logarithm of the length. Different sub-figures stand for scenarios of different divergence time.

4.2.2 Inference with the Ground-truth Introgressed Segments

We analysis the history inference method with the ground truth introgressed segments to confirm the new modification of *MultiWaver* could handle that large time scale history inference, firstly. Segments shorter than 0.015 cM (15kb) will not take in the analysis.

For single wave introgression scenarios or double wave introgression from different archaic lineages, our method could give a good inference of both the introgression time and the number of introgression wave (Supplementary Table 4.13 ~ 4.14). However, when dealing with two waves introgression from a single archaic lineage, our method did not give the accurate inference of a few (7/48) scenarios with biased introgression proportion and closer introgression time of two waves. Instead of giving the two waves results, our method merged the two waves into one wave (Supplementary Table 4.13).

Supplementary Table 4.13 **Introgression history inference results with the ground-truth introgressed segments of single source archaic scenarios**

Section	Archaic Lineage	T_{split} (Gen)	T_{intro} (Gen)	α	Support Ratio	Results Gen(α)
S4.1.1	Denisovan	10680	2000	2.0%	100%	1978.14~2011.65(2.02%)
S4.1.1	Denisovan	11360	2000	2.0%	100%	1990.8~2023.23(2.00%)
S4.1.1	Denisovan	12040	2000	2.0%	99%	1981.76~2013.76(2.04%)
S4.1.1	Denisovan	12720	2000	2.0%	100%	2001.57~2027.22(2.01%)
S4.1.1	Denisovan	13400	2000	2.0%	100%	1983.1~2020.69(2.01%)
S4.1.1	Denisovan	14080	2000	2.0%	92%	1987.68~2015.33(1.98%)
S4.1.1	Denisovan	14760	2000	2.0%	100%	1993.36~2022.18(1.98%)
S4.1.1	Denisovan	15440	2000	2.0%	100%	1989.61~2019.59(2.01%)
S4.1.1	Denisovan	16120	2000	2.0%	100%	2001.37~2033.12(1.96%)
S4.1.1	Neanderthal	3000	2000	2.0%	100%	1984.55~2015.72(2.02%)
S4.1.1	Neanderthal	3200	2000	2.0%	100%	1986.56~2017.82(1.98%)
S4.1.1	Neanderthal	3400	2000	2.0%	100%	1997.1~2025.26(1.99%)
S4.1.1	Neanderthal	3600	2000	2.0%	94%	1987.76~2019.01(2.05%)
S4.1.1	Neanderthal	3800	2000	2.0%	100%	2001.24~2037.07(1.98%)
S4.1.1	Neanderthal	4000	2000	2.0%	100%	1992.67~2027.53(2.00%)
S4.1.1	Neanderthal	4200	2000	2.0%	100%	1995.71~2029.57(2.01%)
S4.1.1	Neanderthal	4400	2000	2.0%	100%	1981.48~2016.63(2.00%)
S4.1.1	Neanderthal	4600	2000	2.0%	100%	1994.9~2029.57(2.00%)
S4.1.2	Denisovan	13400	720	2.0%	100%	721.091~739.371(2.00%)
S4.1.2	Denisovan	13400	1040	2.0%	100%	1036.67~1060.29(1.99%)

Section	Archaic Lineage	T_{split} (Gen)	T_{intro} (Gen)	α	Support Ratio	Results Gen(α)
S4.1.2	Denisovan	13400	1360	2.0%	100%	1351.67~1376.52(2.06%)
S4.1.2	Denisovan	13400	1680	2.0%	100%	1690.21~1714.99(2.00%)
S4.1.2	Denisovan	13400	2000	2.0%	100%	1982.46~2012.53(2.02%)
S4.1.2	Denisovan	13400	2320	2.0%	100%	2297.48~2340.73(2.03%)
S4.1.2	Denisovan	13400	2640	2.0%	99%	2611.09~2647.39(2.01%)
S4.1.2	Denisovan	13400	2960	2.0%	100%	2929.65~2972.37(2.05%)
S4.1.2	Denisovan	13400	3280	2.0%	100%	3239.22~3286.57(2.00%)
S4.1.2	Neanderthal	3800	720	2.0%	98%	714.096~732.83(2.02%)
S4.1.2	Neanderthal	3800	1040	2.0%	100%	1033.8~1053.82(2.02%)
S4.1.2	Neanderthal	3800	1360	2.0%	100%	1350.65~1376.1(2.01%)
S4.1.2	Neanderthal	3800	1680	2.0%	100%	1663.13~1693.74(2.02%)
S4.1.2	Neanderthal	3800	2000	2.0%	100%	1986.44~2011.35(2.02%)
S4.1.2	Neanderthal	3800	2320	2.0%	100%	2303.17~2338.28(2.00%)
S4.1.2	Neanderthal	3800	2640	2.0%	100%	2622.44~2652.68(2.00%)
S4.1.2	Neanderthal	3800	2960	2.0%	97%	2949.4~2989.62(1.94%)
S4.1.2	Neanderthal	3800	3280	2.0%	81%	3247.27~3301.91(1.96%)
S4.1.3	Denisovan	13400	2000	0.4%	100%	1928.3~2002.12(0.38%)
S4.1.3	Denisovan	13400	2000	0.8%	100%	1959.67~2007.93(0.82%)
S4.1.3	Denisovan	13400	2000	1.2%	100%	1974.86~2019.39(1.20%)
S4.1.3	Denisovan	13400	2000	1.6%	100%	1989.61~2028.24(1.62%)
S4.1.3	Denisovan	13400	2000	2.0%	100%	1981.14~2011.96(2.02%)
S4.1.3	Denisovan	13400	2000	2.4%	100%	1999.44~2026.58(2.40%)
S4.1.3	Denisovan	13400	2000	2.8%	98%	1996.13~2023.61(2.81%)
S4.1.3	Denisovan	13400	2000	3.2%	100%	1990.57~2018.36(3.20%)

Section	Archaic Lineage	T_{split} (Gen)	T_{intro} (Gen)	α	Support Ratio	Results Gen(α)
S4.1.3	Denisovan	13400	2000	3.6%	100%	1993.78~2019.01(3.62%)
S4.1.3	Denisovan	13400	2000	4.0%	96%	1994.83~2019.79(3.95%)
S4.1.3	Neanderthal	3800	2000	0.4%	100%	1978.4~2039.58(0.40%)
S4.1.3	Neanderthal	3800	2000	0.8%	99%	1994.16~2045.17(0.79%)
S4.1.3	Neanderthal	3800	2000	1.2%	100%	1978.96~2024.88(1.18%)
S4.1.3	Neanderthal	3800	2000	1.6%	100%	1974.92~2011.44(1.60%)
S4.1.3	Neanderthal	3800	2000	2.0%	100%	1982.18~2016.8(1.98%)
S4.1.3	Neanderthal	3800	2000	2.4%	100%	1999.21~2027.73(2.40%)
S4.1.3	Neanderthal	3800	2000	2.8%	91%	1988.9~2016.86(2.86%)
S4.1.3	Neanderthal	3800	2000	3.2%	100%	2003.49~2023.75(3.17%)
S4.1.3	Neanderthal	3800	2000	3.6%	100%	1995.69~2017.1(3.59%)
S4.1.3	Neanderthal	3800	2000	4.0%	100%	2000.31~2022.82(4.00%)
S4.1.4	Denisovan	13400	1040,1680	0.4%,1.6%	100%	536.609~1304.48(0.04%~1.06%),1566.39~1811.03(0.91%~1.92%)
S4.1.4	Denisovan	13400	1040,1680	0.8%,1.2%	100%	897.606~1220.17(0.40%~1.41%),1567.16~2022.96(0.57%~1.58%)
S4.1.4	Denisovan	13400	1040,1680	1.2%,0.8%	100%	925.636~1120.83(0.74%~1.51%),1532.88~1984.67(0.47%~1.24%)
S4.1.4	Denisovan	13400	1040,1680	1.6%,0.4%	100%	991.159~1108.3(1.07%~1.88%),1383.82~2604.4(0.10%~0.92%)
S4.1.4	Denisovan	13400	1040,2320	0.4%,1.6%	100%	953.39~1110.07(0.35%~0.47%),2296.32~2383.06(1.54%~1.66%)
S4.1.4	Denisovan	13400	1040,2320	0.8%,1.2%	99%	946.378~1085.97(0.61%~0.86%),2178.28~2348.97(1.16%~1.40%)
S4.1.4	Denisovan	13400	1040,2320	1.2%,0.8%	100%	983.265~1085.99(1.07%~1.32%),2190.16~2450.11(0.69%~0.94%)
S4.1.4	Denisovan	13400	1040,2320	1.6%,0.4%	100%	1007.23~1091.41(1.47%~1.68%),2102~2590.97(0.31%~0.52%)
S4.1.4	Denisovan	13400	1040,2960	0.4%,1.6%	100%	1059.32~1234.38(0.38%~0.49%),2914.83~3018.52(1.51%~1.62%)
S4.1.4	Denisovan	13400	1040,2960	0.8%,1.2%	100%	1046.61~1134.05(0.81%~0.91%),2899.48~3060.7(1.11%~1.21%)
S4.1.4	Denisovan	13400	1040,2960	1.2%,0.8%	98%	1032.89~1104.84(1.15%~1.28%),2836.34~3069.77(0.72%~0.85%)
S4.1.4	Denisovan	13400	1040,2960	1.6%,0.4%	100%	1017.14~1076.17(1.50%~1.61%),2726.59~3130.29(0.36%~0.47%)

Section	Archaic Lineage	T_{split} (Gen)	T_{intro} (Gen)	α	Support Ratio	Results Gen(α)
S4.1.4	Denisovan	13400	1680,2320	0.4%,1.6%	83%	1205.55~1880.46(0.10%~0.85%),2240.49~2434.25(1.15%~1.89%)
S4.1.4	Denisovan	13400	1680,2320	0.8%,1.2%	100%	1113.97~1662.2(0.13%~0.76%),2125.79~2356.36(1.24%~1.87%)
S4.1.4	Denisovan	13400	1680,2320	1.2%,0.8%	99%	1258.67~1730.84(0.25%~1.44%),2043.85~2591.83(0.54%~1.73%)
S4.1.4	Denisovan	13400	1680,2320	1.6%,0.4%	97%	1791.08~1826.49(1.94%)
S4.1.4	Denisovan	13400	1680,2960	0.4%,1.6%	93%	1328.87~1874.1(0.20%~0.64%),2853.09~3083.5(1.42%~1.85%)
S4.1.4	Denisovan	13400	1680,2960	0.8%,1.2%	99%	1493.56~1809.59(0.60%~1.06%),2825.74~3215.02(0.92%~1.38%)
S4.1.4	Denisovan	13400	1680,2960	1.2%,0.8%	100%	1642.08~1842.7(1.01%~1.51%),2781.87~3371.62(0.47%~0.98%)
S4.1.4	Denisovan	13400	1680,2960	1.6%,0.4%	100%	1528.35~1747.47(1.08%~1.71%),2449.7~3396.67(0.24%~0.88%)
S4.1.4	Denisovan	13400	2320,2960	0.4%,1.6%	95%	2796.68~2836.64(2.00%)
S4.1.4	Denisovan	13400	2320,2960	0.8%,1.2%	62%	1748.95~2381.8(0.14%~1.03%),2794.27~3145.55(0.95%~1.83%)
S4.1.4	Denisovan	13400	2320,2960	1.2%,0.8%	86%	1810.91~2310.87(0.25%~1.37%),2680.99~3227.07(0.62%~1.74%)
S4.1.4	Denisovan	13400	2320,2960	1.6%,0.4%	93%	2433.43~2466.6(2.00%)
S4.1.4	Neanderthal	3800	1040,1680	0.4%,1.6%	100%	1071.2~1337.64(0.45%~1.38%),1664.93~2042.29(0.66%~1.59%)
S4.1.4	Neanderthal	3800	1040,1680	0.8%,1.2%	99%	883.761~1259.81(0.33%~1.54%),1555.05~2195.65(0.41%~1.62%)
S4.1.4	Neanderthal	3800	1040,1680	1.2%,0.8%	100%	974.03~1143.37(0.89%~1.60%),1550.63~2020.51(0.40%~1.12%)
S4.1.4	Neanderthal	3800	1040,1680	1.6%,0.4%	100%	930.771~1082.23(0.82%~1.82%),1338.57~1989.34(0.19%~1.20%)
S4.1.4	Neanderthal	3800	1040,2320	0.4%,1.6%	100%	948.338~1169.55(0.31%~0.50%),2277.83~2402.36(1.48%~1.66%)
S4.1.4	Neanderthal	3800	1040,2320	0.8%,1.2%	100%	980.458~1103.19(0.69%~0.88%),2217.4~2361.19(1.13%~1.32%)
S4.1.4	Neanderthal	3800	1040,2320	1.2%,0.8%	100%	981.424~1088.59(1.03%~1.26%),2125.51~2378.12(0.72%~0.95%)
S4.1.4	Neanderthal	3800	1040,2320	1.6%,0.4%	100%	961.53~1056.67(1.32%~1.60%),1952.75~2373.48(0.39%~0.68%)
S4.1.4	Neanderthal	3800	1040,2960	0.4%,1.6%	95%	1015.39~1173.56(0.39%~0.49%),2949.51~3045.2(1.50%~1.60%)
S4.1.4	Neanderthal	3800	1040,2960	0.8%,1.2%	100%	1037.18~1137.87(0.79%~0.89%),2911.45~3042.35(1.14%~1.25%)
S4.1.4	Neanderthal	3800	1040,2960	1.2%,0.8%	93%	985.503~1075.76(1.11%~1.25%),2803.66~3016.18(0.77%~0.92%)
S4.1.4	Neanderthal	3800	1040,2960	1.6%,0.4%	100%	1019.46~1081.13(1.55%~1.68%),2740.31~3215.73(0.34%~0.48%)

Section	Archaic Lineage	T_{split} (Gen)	T_{intro} (Gen)	α	Support Ratio	Results Gen(α)
S4.1.4	Neanderthal	3800	1680,2320	0.4%,1.6%	67%	1691.63~1973.44(0.48%~1.37%),2354.08~2785.6(0.66%~1.54%)
S4.1.4	Neanderthal	3800	1680,2320	0.8%,1.2%	92%	792.395~1771.15(0.03%~0.87%),2087.15~2321.95(1.11%~1.95%)
S4.1.4	Neanderthal	3800	1680,2320	1.2%,0.8%	83%	1316.11~1671.44(0.23%~1.07%),2008.09~2223.04(0.95%~1.78%)
S4.1.4	Neanderthal	3800	1680,2320	1.6%,0.4%	77%	1792.26~1829.79(2.00%)
S4.1.4	Neanderthal	3800	1680,2960	0.4%,1.6%	100%	1556.26~1916.21(0.32%~0.72%),2883.38~3115.63(1.28%~1.67%)
S4.1.4	Neanderthal	3800	1680,2960	0.8%,1.2%	98%	1562.92~1907.75(0.67%~1.27%),2866.26~3412.43(0.72%~1.32%)
S4.1.4	Neanderthal	3800	1680,2960	1.2%,0.8%	99%	1491.97~1755.86(0.79%~1.37%),2622.35~3115.97(0.66%~1.24%)
S4.1.4	Neanderthal	3800	1680,2960	1.6%,0.4%	100%	1525.75~1731.54(1.12%~1.69%),2460.79~3135.31(0.32%~0.90%)
S4.1.4	Neanderthal	3800	2320,2960	0.4%,1.6%	79%	2801.37~2846.99(2.00%)
S4.1.4	Neanderthal	3800	2320,2960	0.8%,1.2%	94%	2064.49~2450.48(0.43%~1.47%),2862.86~3484.78(0.53%~1.58%)
S4.1.4	Neanderthal	3800	2320,2960	1.2%,0.8%	99%	2557.94~2601.94(1.96%)
S4.1.4	Neanderthal	3800	2320,2960	1.6%,0.4%	100%	2424.71~2461.87(1.97%)
S4.3	Unknown	18700	2000	2.0%	100%	1977.68~2014.01(1.96%)
S4.3	Unknown	20600	2000	2.0%	100%	1972.95~2006.11(2.03%
S4.3	Unknown	22500	2000	2.0%	100%	1977.42~2009.62(2.00%)
S4.3	Unknown	24400	2000	2.0%	100%	1992.12~2020.37(2.02%)

Supplementary Table 4.14 **Introgression history inference results with the ground-truth introgressed segments of double source archaic scenarios**

Section	$T_{introDen}$ (Gen)	α_{Den}	Support Ratio	Denisovan Results Gen(α)	$T_{introNean}$ (Gen)	α_{Nean}	Support Ratio	Neanderthal Results Gen(α)
S4.2	1200	0.4%	100%	1171.05~1223.08(0.40%)	1200	1.6%	100%	1186.33~1208.76(1.63%)

Section	$T_{introDen}$ (Gen)	α_{Den}	Support Ratio	Denisovan Results Gen(α)	$T_{introNean}$ (Gen)	α_{Nean}	Support Ratio	Neanderthal Results Gen(α)
S4.2	1200	0.8%	100%	1195.78~1230.2(0.82%)	1200	1.2%	100%	1186.16~1219.83(1.20%)
S4.2	1200	1.2%	100%	1203.81~1236.16(1.17%)	1200	0.8%	100%	1200.03~1241.45(0.79%)
S4.2	1200	1.6%	100%	1202.79~1230.02(1.58%)	1200	0.4%	99%	1180.38~1231.55(0.40%)
S4.2	1200	0.4%	100%	1188.09~1241.38(0.40%)	2000	1.6%	100%	1984.46~2024.52(1.59%)
S4.2	1200	0.8%	100%	1199.06~1238.27(0.80%)	2000	1.2%	100%	1989.45~2032.7(1.19%)
S4.2	1200	1.2%	100%	1202.26~1232.51(1.20%)	2000	0.8%	100%	1979.98~2032.71(0.77%)
S4.2	1200	1.6%	100%	1195.79~1223.79(1.57%)	2000	0.4%	100%	1951.45~2022.49(0.39%)
S4.2	1200	0.4%	99%	1177.83~1236.4(0.40%)	2800	1.6%	100%	2775.79~2821.67(1.57%)
S4.2	1200	0.8%	100%	1184.8~1230.39(0.81%)	2800	1.2%	100%	2759.15~2807.52(1.22%)
S4.2	1200	1.2%	96%	1185.07~1218.2(1.21%)	2800	0.8%	97%	2787.81~2851.11(0.78%)
S4.2	1200	1.6%	100%	1186.9~1214.52(1.60%)	2800	0.4%	100%	2784.54~2870.45(0.39%)
S4.2	2000	0.4%	100%	1977.22~2040.37(0.40%)	1200	1.6%	100%	1200.27~1225.76(1.66%)
S4.2	2000	0.8%	100%	1964.9~2023.75(0.78%)	1200	1.2%	99%	1181.58~1209.93(1.22%)
S4.2	2000	1.2%	100%	1982.25~2021.45(1.23%)	1200	0.8%	98%	1226.62~1266.22(0.77%)
S4.2	2000	1.6%	100%	1989.55~2025.14(1.60%)	1200	0.4%	100%	1167.24~1225.8(0.42%)
S4.2	2000	0.4%	100%	1991.86~2063.55(0.40%)	2000	1.6%	100%	1987.32~2020.15(1.59%)
S4.2	2000	0.8%	100%	2019.14~2071.5(0.76%)	2000	1.2%	100%	1997.98~2039.69(1.18%)
S4.2	2000	1.2%	100%	1995.15~2033.91(1.17%)	2000	0.8%	100%	1998.48~2042.21(0.82%)
S4.2	2000	1.6%	100%	1992.98~2027.09(1.60%)	2000	0.4%	97%	1968.76~2041.11(0.39%)
S4.2	2000	0.4%	100%	1951.74~2026.36(0.41%)	2800	1.6%	100%	2772.99~2815.81(1.61%)
S4.2	2000	0.8%	100%	1980.16~2036.68(0.80%)	2800	1.2%	100%	2775.97~2825.95(1.17%)
S4.2	2000	1.2%	97%	1996.53~2047.44(1.17%)	2800	0.8%	100%	2790.76~2848.9(0.78%)
S4.2	2000	1.6%	100%	1979.46~2016.18(1.56%)	2800	0.4%	100%	2742.28~2832.25(0.39%)

Section	$T_{introDen}$ (Gen)	α_{Den}	Support Ratio	Denisovan Results Gen(α)	$T_{introNean}$ (Gen)	α_{Nean}	Support Ratio	Neanderthal Results Gen(α)
S4.2	2800	0.4%	100%	2753.55~2839.07(0.40%)	1200	1.6%	100%	1177.7~1207.22(1.61%)
S4.2	2800	0.8%	100%	2751.21~2823.04(0.80%)	1200	1.2%	100%	1175.04~1209.46(1.21%)
S4.2	2800	1.2%	100%	2781.57~2833.39(1.22%)	1200	0.8%	96%	1195.36~1229.83(0.79%)
S4.2	2800	1.6%	100%	2785.25~2830.97(1.55%)	1200	0.4%	100%	1178.25~1233.22(0.39%)
S4.2	2800	0.4%	100%	2742.03~2840.24(0.40%)	2000	1.6%	100%	1980.35~2023.9(1.58%)
S4.2	2800	0.8%	99%	2801.21~2863.68(0.77%)	2000	1.2%	100%	1995.53~2036.07(1.18%)
S4.2	2800	1.2%	99%	2771.45~2822.27(1.19%)	2000	0.8%	100%	1986.49~2044.17(0.78%)
S4.2	2800	1.6%	100%	2760.35~2804.09(1.62%)	2000	0.4%	100%	1986.84~2069.25(0.40%)
S4.2	2800	0.4%	100%	2722.11~2817.17(0.39%)	2800	1.6%	98%	2767.22~2809.52(1.60%)
S4.2	2800	0.8%	100%	2803.72~2870.51(0.79%)	2800	1.2%	100%	2778.35~2836.26(1.18%)
S4.2	2800	1.2%	95%	2790.26~2840.97(1.16%)	2800	0.8%	100%	2781.63~2850.82(0.81%)
S4.2	2800	1.6%	100%	2776.91~2822.68(1.59%)	2800	0.4%	100%	2770.97~2855.5(0.40%)

In summary, the modification of *MultiWaver* method could theoretically deal with the introgression history inference under diverse scenarios. In most cases, our methods could accurate estimate the time of introgression events and their corresponding introgression time and proportion.

4.2.3 Inference with the Inferred Segments

The ground-truth segments helped us prove the validity of the history inference method. Then we evaluated this method with the inferred segments. For all 144 different simulation scenarios, our method correctly inferred 122 times of them (Supplementary Table 4.15 ~ 4.16). Here, the correct inferred stands for accurate inferring the number of introgression events and relative accurate estimating the introgression time and proportion of each event. We found that, when the number of introgression events correctly inferred, the results of introgression time and proportion were often not so bad (Supplementary Table 4.15 ~ 4.16).

Under different demographic scenarios, the performances of our method are slightly different. For scenarios with different introgression time (Supplementary Note 3.1.2) and the scenarios of introgression from unknown archaic (Supplementary Note 3.3), our method perfectly inferred all 24 times analysis (Supplementary Table 4.15). Under the scenarios with different divergence time to the archaic lineages (Supplementary Note 3.1.1), our method only make one time mistake for inferring an extra introgression event in all 18 times analysis (Supplementary Table 4.15).

In the scenarios of different introgression proportion, our method shows bad performance when the introgression is small (⩽1.2% for Denisovan and ⩽0.4% for Neanderthal). That may because the bad segments inference under these extreme lower introgression proportion scenarios (Supplementary Table 4.15).

When two introgression events from one archaic hominin (Supplementary Note 3.1.4), our methods accurate inferred most cases (44/48). The 4 times erroneous inferences are in those scenarios with two relative closer introgression events and one event play a dominant role in the proportion. For those 4 results, our methods combined the 2 waves into one wave (Supplementary Table 4.16).

For those scenarios of two archaic hominins introgression, our method correctly inferred 23 times in the total 36 analysis. In the 13 times erroneous inferences, 2 of them are due to the mistake of Denisovan introgression history and the other 11 times are due to Neanderthal. For the 2 analyses of misestimating of Denisovan history, our method inferred one extra wave of ancient (~6200 generation ago) and lower proportion event. The rest 11 times of Neanderthal misestimate are due to inferring one extra wave with smaller introgression proportion event. The introgression time of the extra event in the Neanderthal analysis are not determined. For the scenarios with recent Neanderthal introgression event (1200 generation), the extra event is often ancient, while the scenarios with ancient introgression event (>2000 generation), the extra event is often recent (Supplementary Table 4.16). In summary, the extra events of all those 13 misestimates often with smaller introgression proportion and the introgression proportion shows unstable in the bootstrapping analysis. The extra events often not affect the inference of the "real" introgressed wave (Supplementary Table 4.16).

Supplementary Table 4.15 **Introgression history inference results with the inferred introgressed segments of single source archaic scenarios**

Section	Archaic Lineage	T_{split} (Gen)	T_{intro} (Gen)	α	Support Ratio	Results Gen(α)
S4.1.1	Denisovan	10680	2000	2.0%	93%	1876.84~1910.12(1.97%)
S4.1.1	Denisovan	11360	2000	2.0%	91%	1872.43~1904.37(1.95%)
S4.1.1	Denisovan	12040	2000	2.0%	94%	1860.4~1891.07(1.97%)
S4.1.1	Denisovan	12720	2000	2.0%	100%	1885.77~1915.35(1.93%)
S4.1.1	Denisovan	13400	2000	2.0%	96%	1870.12~1898.02(1.92%)
S4.1.1	Denisovan	14080	2000	2.0%	81%	1019.47~1638.27(0.07%~1.04%),1913.73~2224.84(0.86%~1.82%)
S4.1.1	Denisovan	14760	2000	2.0%	99%	1863.08~1901.61(1.86%)
S4.1.1	Denisovan	15440	2000	2.0%	99%	1875.49~1906.14(1.84%)
S4.1.1	Denisovan	16120	2000	2.0%	100%	1924.6~1961.7(1.67%)
S4.1.1	Neanderthal	3000	2000	2.0%	100%	1893.19~1923.72(1.97%)
S4.1.1	Neanderthal	3200	2000	2.0%	100%	1907.1~1937.15(1.93%)
S4.1.1	Neanderthal	3400	2000	2.0%	100%	1911.49~1941.95(1.95%)
S4.1.1	Neanderthal	3600	2000	2.0%	100%	1905.22~1935.25(2.01%)
S4.1.1	Neanderthal	3800	2000	2.0%	100%	1911.06~1941.58(1.95%)
S4.1.1	Neanderthal	4000	2000	2.0%	100%	1902.33~1932.06(1.96%)
S4.1.1	Neanderthal	4200	2000	2.0%	100%	1912.45~1947.56(1.98%)
S4.1.1	Neanderthal	4400	2000	2.0%	100%	1870.99~1903.61(1.97%)
S4.1.1	Neanderthal	4600	2000	2.0%	100%	1894.98~1931.99(1.98%)
S4.1.2	Denisovan	13400	720	2.0%	92%	685.257~703.852(1.99%)
S4.1.2	Denisovan	13400	1040	2.0%	100%	984.894~1009.38(1.97%)

Section	Archaic Lineage	T_{split} (Gen)	T_{intro} (Gen)	α	Support Ratio	Results Gen(α)
S4.1.2	Denisovan	13400	1360	2.0%	89%	1279.68~1303.13(2.01%)
S4.1.2	Denisovan	13400	1680	2.0%	100%	1600.39~1627.07(1.93%)
S4.1.2	Denisovan	13400	2000	2.0%	99%	1866.99~1896.99(1.93%)
S4.1.2	Denisovan	13400	2320	2.0%	98%	2142.43~2182.07(1.92%)
S4.1.2	Denisovan	13400	2640	2.0%	89%	2432.48~2479.1(1.89%)
S4.1.2	Denisovan	13400	2960	2.0%	100%	2722.85~2761.95(1.91%)
S4.1.2	Denisovan	13400	3280	2.0%	100%	3005.74~3049.26(1.84%)
S4.1.2	Neanderthal	3800	720	2.0%	100%	795.519~813.109(1.65%)
S4.1.2	Neanderthal	3800	1040	2.0%	100%	1055.57~1075.47(1.81%)
S4.1.2	Neanderthal	3800	1360	2.0%	100%	1323.75~1349.68(1.90%)
S4.1.2	Neanderthal	3800	1680	2.0%	100%	1607.79~1636.81(1.95%)
S4.1.2	Neanderthal	3800	2000	2.0%	100%	1891.74~1922.75(1.98%)
S4.1.2	Neanderthal	3800	2320	2.0%	100%	2190.61~2228.06(1.98%)
S4.1.2	Neanderthal	3800	2640	2.0%	100%	2473.02~2505(1.98%)
S4.1.2	Neanderthal	3800	2960	2.0%	100%	2762.64~2809.48(1.93%)
S4.1.2	Neanderthal	3800	3280	2.0%	96%	3031.02~3079.67(1.95%)
S4.1.3	Denisovan	13400	2000	0.4%	62%	322.297~779.04(0.01%~0.03%),1790.11~1883.29(0.29%~0.31%)
S4.1.3	Denisovan	13400	2000	0.8%	88%	871.534~1207.61(0.06%~0.14%),1869.33~1959.28(0.64%~0.73%)
S4.1.3	Denisovan	13400	2000	1.2%	82%	911.945~1487.55(0.04%~0.43%),1886.12~2080.69(0.74%~1.12%)
S4.1.3	Denisovan	13400	2000	1.6%	74%	1848.48~1897.19(1.56%)
S4.1.3	Denisovan	13400	2000	2.0%	100%	1864.71~1894.78(1.93%)
S4.1.3	Denisovan	13400	2000	2.4%	100%	1887.61~1919.63(2.29%)
S4.1.3	Denisovan	13400	2000	2.8%	99%	1885.29~1910.87(2.69%)
S4.1.3	Denisovan	13400	2000	3.2%	100%	1893.25~1917.55(3.05%)

Section	Archaic Lineage	T_{split} (Gen)	T_{intro} (Gen)	α	Support Ratio	Results Gen(α)
S4.1.3	Denisovan	13400	2000	3.6%	100%	1898.22~1919.13(3.45%)
S4.1.3	Denisovan	13400	2000	4.0%	96%	1894.37~1917.73(3.75%)
S4.1.3	Neanderthal	3800	2000	0.4%	100%	551.003~765.296(0.03%~0.06%),1857.03~1962.06(0.36%~0.39%)
S4.1.3	Neanderthal	3800	2000	0.8%	54%	1831.87~1875.62(0.81%)
S4.1.3	Neanderthal	3800	2000	1.2%	100%	1867.48~1906.74(1.18%)
S4.1.3	Neanderthal	3800	2000	1.6%	100%	1877.75~1912.83(1.59%)
S4.1.3	Neanderthal	3800	2000	2.0%	100%	1896.59~1928.28(1.94%)
S4.1.3	Neanderthal	3800	2000	2.4%	100%	1916.37~1946.94(2.35%)
S4.1.3	Neanderthal	3800	2000	2.8%	100%	1912.52~1936.62(2.76%)
S4.1.3	Neanderthal	3800	2000	3.2%	100%	1929.92~1958.38(3.05%)
S4.1.3	Neanderthal	3800	2000	3.6%	100%	1922.37~1946.28(3.43%)
S4.1.3	Neanderthal	3800	2000	4.0%	100%	1938.51~1963.96(3.81%)
S4.1.4	Denisovan	13400	1040,1680	0.4%,1.6%	99%	476.831~1218.82(0.04%~1.08%),1471.51~1797.7(0.83%~1.86%)
S4.1.4	Denisovan	13400	1040,1680	0.8%,1.2%	100%	903.433~1163.5(0.48%~1.45%),1508.69~2008.99(0.47%~1.44%)
S4.1.4	Denisovan	13400	1040,1680	1.2%,0.8%	100%	933.827~1080.55(0.94%~1.54%),1494.58~1990.79(0.39%~0.99%)
S4.1.4	Denisovan	13400	1040,1680	1.6%,0.4%	100%	886.753~1028.14(0.91%~1.76%),1318.48~2107.42(0.17%~1.04%)
S4.1.4	Denisovan	13400	1040,2320	0.4%,1.6%	100%	1000.67~1163.11(0.42%~0.61%),2203.1~2332.13(1.30%~1.49%)
S4.1.4	Denisovan	13400	1040,2320	0.8%,1.2%	100%	921.473~1055.81(0.61%~0.85%),2049.75~2221.46(1.08%~1.32%)
S4.1.4	Denisovan	13400	1040,2320	1.2%,0.8%	100%	954.976~1041.61(1.08%~1.31%),2097.86~2350.54(0.63%~0.86%)
S4.1.4	Denisovan	13400	1040,2320	1.6%,0.4%	100%	963.472~1037.92(1.48%~1.70%),2090.6~2676.74(0.23%~0.46%)
S4.1.4	Denisovan	13400	1040,2960	0.4%,1.6%	100%	1070.72~1261.55(0.42%~0.57%),2764.25~2925.54(1.30%~1.45%)
S4.1.4	Denisovan	13400	1040,2960	0.8%,1.2%	100%	1002.29~1105.37(0.81%~0.94%),2713.19~2905.17(0.97%~1.10%)
S4.1.4	Denisovan	13400	1040,2960	1.2%,0.8%	100%	994.289~1079.86(1.18%~1.30%),2746.14~3041.76(0.61%~0.73%)
S4.1.4	Denisovan	13400	1040,2960	1.6%,0.4%	99%	967.199~1026.46(1.47%~1.58%),2506.13~2936.83(0.33%~0.44%)

Section	Archaic Lineage	T_{split} (Gen)	T_{intro} (Gen)	α	Support Ratio	Results Gen(α)
S4.1.4	Denisovan	13400	1680,2320	0.4%,1.6%	95%	1440.87~1788.14(0.32%~1.09%),2186.95~2447.81(0.81%~1.57%)
S4.1.4	Denisovan	13400	1680,2320	0.8%,1.2%	100%	1053.58~1535.67(0.17%~0.77%),2017.44~2242.76(1.13%~1.72%)
S4.1.4	Denisovan	13400	1680,2320	1.2%,0.8%	99%	1336.49~1587.26(0.52%~1.23%),1989.15~2285.42(0.67%~1.38%)
S4.1.4	Denisovan	13400	1680,2320	1.6%,0.4%	70%	1673.84~1712.2(1.86%)
S4.1.4	Denisovan	13400	1680,2960	0.4%,1.6%	100%	1276.5~1750.96(0.22%~0.52%),2694.39~2892.38(1.40%~1.70%)
S4.1.4	Denisovan	13400	1680,2960	0.8%,1.2%	100%	1495.25~1714.45(0.66%~1.09%),2679.9~3111.14(0.77%~1.19%)
S4.1.4	Denisovan	13400	1680,2960	1.2%,0.8%	100%	1551.85~1701.25(1.01%~1.42%),2612.88~3250.48(0.45%~0.87%)
S4.1.4	Denisovan	13400	1680,2960	1.6%,0.4%	100%	1447.28~1625.15(1.08%~1.64%),2308.45~3393.98(0.24%~0.80%)
S4.1.4	Denisovan	13400	2320,2960	0.4%,1.6%	56%	2594.76~2640.44(1.86%)
S4.1.4	Denisovan	13400	2320,2960	0.8%,1.2%	70%	1701.66~2185.2(0.19%~0.90%),2617.66~2897.48(0.93%~1.64%)
S4.1.4	Denisovan	13400	2320,2960	1.2%,0.8%	92%	1484.91~2109.78(0.16%~1.10%),2484.72~2937.92(0.76%~1.69%)
S4.1.4	Denisovan	13400	2320,2960	1.6%,0.4%	52%	1585.57~2064.17(0.23%~1.25%),2391.95~2777.49(0.64%~1.66%)
S4.1.4	Neanderthal	3800	1040,1680	0.4%,1.6%	100%	1053.19~1293.3(0.55%~1.51%),1625.97~2050.66(0.47%~1.42%)
S4.1.4	Neanderthal	3800	1040,1680	0.8%,1.2%	100%	837.133~1176.61(0.37%~1.43%),1466.54~1964.97(0.45%~1.52%)
S4.1.4	Neanderthal	3800	1040,1680	1.2%,0.8%	100%	943.166~1076.05(1.01%~1.58%),1530.37~1919.51(0.38%~0.95%)
S4.1.4	Neanderthal	3800	1040,1680	1.6%,0.4%	100%	932.276~1032.47(1.18%~1.76%),1360.86~1875.98(0.21%~0.80%)
S4.1.4	Neanderthal	3800	1040,2320	0.4%,1.6%	100%	985.33~1179.94(0.36%~0.57%),2164.18~2318.21(1.31%~1.51%)
S4.1.4	Neanderthal	3800	1040,2320	0.8%,1.2%	100%	957.691~1101.92(0.73%~0.96%),2098.19~2311.61(0.96%~1.19%)
S4.1.4	Neanderthal	3800	1040,2320	1.2%,0.8%	100%	976.49~1056.67(1.11%~1.33%),2116.18~2405.85(0.58%~0.80%)
S4.1.4	Neanderthal	3800	1040,2320	1.6%,0.4%	100%	918.03~990.958(1.32%~1.54%),1839.89~2177.32(0.41%~0.62%)
S4.1.4	Neanderthal	3800	1040,2960	0.4%,1.6%	96%	1007.57~1191.46(0.41%~0.55%),2769.16~2911.65(1.30%~1.45%)
S4.1.4	Neanderthal	3800	1040,2960	0.8%,1.2%	100%	1042.81~1135.49(0.82%~0.94%),2761.09~2940.85(0.98%~1.10%)
S4.1.4	Neanderthal	3800	1040,2960	1.2%,0.8%	98%	957.32~1040.01(1.11%~1.25%),2627.69~2878.96(0.69%~0.83%)
S4.1.4	Neanderthal	3800	1040,2960	1.6%,0.4%	100%	974.202~1027.28(1.51%~1.64%),2502.94~2949.17(0.32%~0.45%)

Section	Archaic Lineage	T_{split} (Gen)	T_{intro} (Gen)	α	Support Ratio	Results Gen(α)
S4.1.4	Neanderthal	3800	1680,2320	0.4%,1.6%	90%	1621.09~1865.18(0.50%~1.47%),2217.42~2758.33(0.45%~1.42%)
S4.1.4	Neanderthal	3800	1680,2320	0.8%,1.2%	98%	860.702~1637.5(0.05%~0.85%),1962.55~2223.41(1.04%~1.83%)
S4.1.4	Neanderthal	3800	1680,2320	1.2%,0.8%	100%	1021.76~1467.17(0.16%~0.75%),1880.11~2070.4(1.17%~1.76%)
S4.1.4	Neanderthal	3800	1680,2320	1.6%,0.4%	56%	1680.08~1710.07(1.92%)
S4.1.4	Neanderthal	3800	1680,2960	0.4%,1.6%	100%	1555.41~1898.83(0.42%~0.82%),2783.93~3034.3(1.04%~1.44%)
S4.1.4	Neanderthal	3800	1680,2960	0.8%,1.2%	100%	1433.68~1699.75(0.57%~0.99%),2607.46~2956.6(0.88%~1.30%)
S4.1.4	Neanderthal	3800	1680,2960	1.2%,0.8%	100%	1451.37~1720.47(0.88%~1.48%),2485.85~3218.1(0.44%~1.03%)
S4.1.4	Neanderthal	3800	1680,2960	1.6%,0.4%	100%	1415.89~1586.86(1.07%~1.57%),2303.63~2890.38(0.36%~0.86%)
S4.1.4	Neanderthal	3800	2320,2960	0.4%,1.6%	75%	1858.15~2264.01(0.30%~0.88%),2758.66~3011.12(0.98%~1.56%)
S4.1.4	Neanderthal	3800	2320,2960	0.8%,1.2%	69%	1685.7~2230.03(0.21%~1.13%),2622.02~3012.42(0.74%~1.66%)
S4.1.4	Neanderthal	3800	2320,2960	1.2%,0.8%	69%	2382.75~2427.91(1.83%)
S4.1.4	Neanderthal	3800	2320,2960	1.6%,0.4%	70%	1639.11~2002.7(0.28%~1.02%),2388.12~2673.27(0.84%~1.58%)
S4.3	Unknown	18700	2000	2.0%	100%	1865.8~1899.85(1.78%)
S4.3	Unknown	20600	2000	2.0%	100%	1844.29~1878.73(1.75%)
S4.3	Unknown	22500	2000	2.0%	100%	1812.01~1850.55(1.58%)
S4.3	Unknown	24400	2000	2.0%	100%	1817.26~1854.48(1.29%)

Supplementary Table 4.16 **Introgression history inference results with the inferred introgressed segments of double source archaic scenarios**

Section	$T_{introDen}$ (Gen)	α_{Den}	Support Ratio	Denisovan Results Gen(α)	$T_{introNean}$ (Gen)	α_{Nean}	Support Ratio	Neanderthal Results Gen(α)
S4.2	1200	0.4%	98%	1135.87~1199.05(0.34%)	1200	1.6%	100%	1193.21~1215.52(1.63%)
S4.2	1200	0.8%	100%	1123.42~1164.84(0.70%)	1200	1.2%	100%	1250.26~1279.24(1.32%)
S4.2	1200	1.2%	100%	1121.21~1155.86(1.00%)	1200	0.8%	100%	1316.65~1357.73(1.02%)
S4.2	1200	1.6%	100%	1103.04~1128.13(1.36%)	1200	0.4%	100%	1415.5~1468.4(0.75%)
S4.2	1200	0.4%	100%	1218.2~1277.14(0.36%)	2000	1.6%	100%	1870.31~1912.06(1.65%)
S4.2	1200	0.8%	99%	1164.79~1205.59(0.69%)	2000	1.2%	100%	1850.8~1892.83(1.36%)
S4.2	1200	1.2%	100%	1121.41~1153.28(1.04%)	2000	0.8%	100%	1814.8~1862.68(1.04%)
S4.2	1200	1.6%	100%	1094.41~1124.62(1.36%)	2000	0.4%	96%	1749.88~1802.07(0.76%)
S4.2	1200	0.4%	97%	1327.65~1408.09(0.37%)	2800	1.6%	57%	1242.5~2150.8(0.04%~0.64%), 2575.71~2815.18(1.01%~1.60%)
S4.2	1200	0.8%	99%	1183.76~1225.48(0.71%)	2800	1.2%	100%	894.151~2063.44(0.03%~0.64%), 2465.62~2804.5(0.76%~1.37%)
S4.2	1200	1.2%	89%	1136.92~1173.62(1.06%)	2800	0.8%	100%	1442.75~1948.47(0.22%~0.67%), 2611.09~3107.83(0.38%~0.84%)
S4.2	1200	1.6%	100%	1100.07~1126.13(1.39%)	2800	0.4%	100%	1280.29~1773.6(0.17%~0.51%), 2414.61~3061.03(0.26%~0.59%)
S4.2	2000	0.4%	99%	1871.03~1956.7(0.32%)	1200	1.6%	100%	1264.77~1291.54(1.66%)
S4.2	2000	0.8%	100%	1842.88~1898.95(0.61%)	1200	1.2%	100%	1348.75~1379.53(1.38%)
S4.2	2000	1.2%	100%	1809.87~1861.19(0.99%)	1200	0.8%	100%	1419.06~1487.4(0.95%~1.01%),

Section	$T_{introDen}$ (Gen)	α_{Den}	Support Ratio	Denisovan Results Gen(α)	$T_{introNean}$ (Gen)	α_{Nean}	Support Ratio	Neanderthal Results Gen(α)
								3335.93~4388.21(0.07%~0.13%)
S4.2	2000	1.6%	100%	1801.51~1847.35(1.30%)	1200	0.4%	100%	1406.12~1575.87(0.53%~0.69%),<br>2550.62~3172.17(0.18%~0.34%)
S4.2	2000	0.4%	97%	1977.08~2054.99(0.33%)	2000	1.6%	100%	1932.91~1964.78(1.67%)
S4.2	2000	0.8%	100%	1913.29~1968.72(0.62%)	2000	1.2%	100%	1980.98~2024.7(1.38%)
S4.2	2000	1.2%	100%	1842.42~1883.27(0.96%)	2000	0.8%	100%	2068.03~2113.01(1.15%)
S4.2	2000	1.6%	100%	1814.05~1851.72(1.31%)	2000	0.4%	100%	2182.01~2239.85(0.84%)
S4.2	2000	0.4%	67%	1771.03~1905.24(0.32%~0.33%),<br>6230.85~9613.72(0.03%~0.04%)	2800	1.6%	100%	2567.5~2617.96(1.70%)
S4.2	2000	0.8%	99%	1905.3~1964.18(0.67%)	2800	1.2%	100%	2580.45~2630.49(1.38%)
S4.2	2000	1.2%	100%	1881.63~1923.93(0.96%)	2800	0.8%	100%	2570.49~2621.33(1.10%)
S4.2	2000	1.6%	100%	1812.37~1854.24(1.28%)	2800	0.4%	100%	2501.33~2557.1(0.85%)
S4.2	2800	0.4%	100%	2594.43~2690.94(0.30%)	1200	1.6%	100%	1301.42~1326.13(1.63%)
S4.2	2800	0.8%	100%	2535.01~2600.95(0.60%)	1200	1.2%	100%	1342.98~1380.22(1.29%~1.32%),<br>4736.31~5977.73(0.07%~0.09%)
S4.2	2800	1.2%	100%	2525.09~2585.86(0.93%)	1200	0.8%	100%	1359.01~1446.55(0.86%~0.92%),<br>3761.84~4406.11(0.20%~0.26%)
S4.2	2800	1.6%	100%	2521.96~2576.47(1.19%)	1200	0.4%	100%	1429.32~1554.16(0.49%~0.55%),<br>3555.57~3930.48(0.33%~0.40%)
S4.2	2800	0.4%	99%	2681.98~2765.48(0.32%)	2000	1.6%	100%	1976.75~2008.11(1.67%)
S4.2	2800	0.8%	100%	2621.66~2700.53(0.60%)	2000	1.2%	96%	2102.4~2141.24(1.40%)
S4.2	2800	1.2%	100%	2539.7~2599.4(0.92%)	2000	0.8%	98%	1980.55~2176.77(0.72%~1.04%),<br>2890.34~4228.2(0.11%~0.43%)

Section	$T_{introDen}$ (Gen)	α_{Den}	Support Ratio	Denisovan Results Gen(α)	$T_{introNean}$ (Gen)	α_{Nean}	Support Ratio	Neanderthal Results Gen(α)
S4.2	2800	1.6%	100%	2506.81~2562.17(1.26%)	2000	0.4%	100%	1787.37~2300.41(0.26%~0.67%), 3069.57~3981.35(0.25%~0.66%)
S4.2	2800	0.4%	75%	2469.75~2666.61(0.28%~0.31%), 6180.64~10076.1(0.03%~0.05%)	2800	1.6%	100%	2627.42~2673.52(1.69%)
S4.2	2800	0.8%	96%	2674.18~2741.85(0.63%)	2800	1.2%	100%	2719.95~2763.01(1.41%)
S4.2	2800	1.2%	100%	2568.8~2623.81(0.91%)	2800	0.8%	100%	2798.25~2864.51(1.17%)
S4.2	2800	1.6%	100%	2529.06~2583.37(1.24%)	2800	0.4%	100%	2942.74~3007.16(0.90%)

4.3 Further Simulation Validations

We would like to thank three anonymous reviewers for providing us with insightful comments on previous versions of this paper, especially those concerning the benchmarking studies. Partially motivated to address these concerns, we did some additional simulation validations with *msprime* [27] under another demographic model from a more recent study[28], with a focus on the impact of incomplete lineage sorting (ILS) sequences and the differences between *ArchaicSeeker 2.0* and *D statistics*, one popular site-based introgression estimation method. These studies provided further evidence that the performance of *ArchaicSeeker 2.0* is robust for various simulation tools and demographic models. Furthermore, they revealed that compared with *D statistics*, *ArchaicSeeker 2.0* performs relatively better on mitigating the confounding impacts caused by ILS, and hence is less likely to result in overestimation.

4.3.1 Simulations with *msprime*

The simulation approaches we used in S3 are based on the software *ms* and the demographic model used in a study by *Vernot et al*[20]. To assess how *ArchaicSeeker 2.0* might be affected by different simulation approaches and demographic models. For additional simulation approaches, we used *msprime,* which is an updated version of *ms* which allows proper interpretation of the written model when we simulated to generate ancient samples[29] and contains a built-in module to trace the ground truth introgressed sequences. For additional demographic model, we chose the demographic model used by *Skov et al* in a recent study[28], where the estimated demographic parameters and admixture events differ at certain levels from *Vernot's* model[20].

Based on the simulation model by *Skov et al*[28], we used both *ms* and *msprime* to generate simulation datasets under *Skov's* model, with *msprime* code obtained directly from their study (https://github.com/LauritsSkov/ArchaicSimulations). We simulated 10 Mb sequences of 100 Africans (200 haplotypes), 100 Non-Africans (200 haplotypes), 1 Neanderthal (2 haplotypes) and 1 Denisovan (2 haplotypes) with

msprime and *ms*, respectively, and repeated them 100 times.

The inference results by *ArchaicSeeker 2.0* on the datasets generated by both simulation tools are almost identical. For *ms*-based simulations, the mean value of Precision is 94.72% (92.47% ~ 96.06%), TPR is 84.37% (80.45% ~ 88.01%) and FPR is 0.50% (0.32% ~ 0.76%). For *msprime*-based simulations, the mean value of Precision is 94.52% (92.59% ~ 95.81%), TPR is 86.05% (82.13% ~ 89.19%) and FPR is 0.52% (0.30% ~ 0.86%).

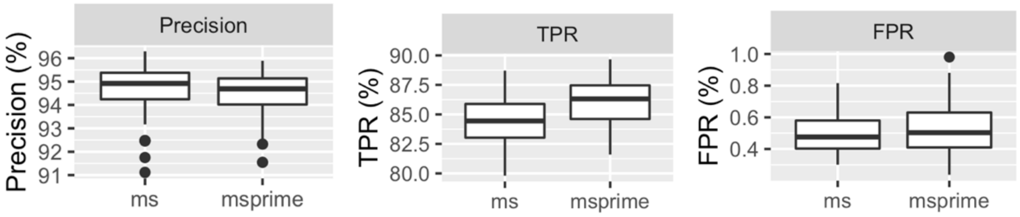

Supplementary Figure 4.44 Length based evaluation with *ms* and *msprime* under *Skov's* simulation scenario. Comparison between the inferred introgressed segments and the ground truth segments under the scenarios described in *Skov et al* 2020[28]. The y-axis represents the summary statistics of precision, TPR and FPR, respectively. The x-axis represents the two simulation methods (*ms* and *msprime*) that are used to generate the data. 100 replicates were performed independently and 100 test individuals were analyzed within each replication. Bounds of box represent the interquartile range (IQR; 25th to 75th percentile), with a center line indicating the median. Whiskers are represented in the form of Tukey style.

Although, there might be some reservation on *ms*, especially when people applied this method to complex history model, we did not observe significant differences between the results of the two simulation methods in our study. Therefore, these additional studies provide further confidence on the robustness and reliability of the simulation framework used in Supplementary Note 3.

4.3.2 Incomplete Lineage Sorting

Incomplete lineage sorting (ILS) is a challenging issue for studying archaic introgression due to its confounding impact. To assess its impact on *ArchaicSeeker*

2.0, we used msprime to generate simulation data in which the real introgressed sequences and the ILS sequences are distinguished, a feature not available for *ms*.

We note that the TPR of msprime simulations (86.05%) are slightly higher than that of the *ms* simulations (84.37%) (Supplementary Figure 4.44), as ILS sequences are excluded in msprime simulations. This indicated our method could remove certain ILS sequences, as part of the design goals of our method. Indeed, in *ArchaicSeeker 2.0* we used the Hidden Markov Model to model the distribution of the archaic introgression sequences on the modern non-African human genomes. Unlike site-based methods, such as D statistics and F statistics, we considered not only the nucleotide / SNP information, but also the length of each introgressed sequence. The ILS should affect the site-based method much greater than our method, which might also be part of the reasons that comparatively higher over estimations observed on site-based methods, as detailed in the next section.

4.3.3 D Statistics

Compared with several previous studies based on site-based introgression estimation methods, such as *D Statistics* and *F Statistics*, our method turned out to be more conservative when it comes to estimate introgression proportions. To investigate this difference, we calculated *D Statistics* with our simulation data, which showed *D Statistics* overestimated the archaic introgression for 5-10 folds based on our simulation analysis.

As a step towards assessing the effectiveness of site-based statistics for estimating the archaic introgression proportion, we used the *D Statistics* to calculate the introgression proportion of Neanderthal and Denisovan based on simulated data. We used *msprime* to simulate five populations approximating Vindija Neanderthal, Denisovan, Chimpanzee, Africans, and non-Africans mainly following the *Skov's* model[28]. The sample size of each population is 1, 1, 1, 1, 100, and 100, respectively. In addition to the parameters in *Skov's* model, we here also included detailed demographic parameters simulating population Chimpanzee: the population size of the common ancestor of Chimpanzee and human was 52,000 and the population size

of Chimpanzee was 15,000 [30]. The split time of Chimpanzee and human was set as 6,000,000 years [31]. The simulated chromosome length was 30Mb and we assumed the recombination rate as 1.2 cM/Mb and mutation rate as 1.2e-8 per generation per site, along with a 29-year generation time. The introgression proportions were set as 0.5% and 1% 45,000 years ago. *AdmixTools* v 7.0.2 [32] was used to calculate the D Statistics and the equation was

$$\frac{D(African, Test, Introgressor, Chimpanzee)}{D(African, Introgressor_1, Introgressor_2, Chimpanzee)}, \quad (23)$$

where $Introgressor_1$ and $Introgressor_2$ were randomly chosen subsets of half of the Introgressor (Neanderthal or Denisovan)[33]. It turned out that the *D statistics* overestimated for 5-10 folds based on the estimation results (Supplementary Figure 4.45 and Supplementary Table 4.17, 4.18).

Finally, we would like to point out that our results are in line with some other studies which show that *D Statistics* are likely to overestimate the archaic introgression proportion. For example, the authors in a recent study[33] compared the introgression proportion estimated by *D Statistics* and their method SARGE, and they did not observe that high proportion of Denisovan ancestry in Oceania with their method while D Statistics suggested a higher introgression proportion (Figure 3, Nathan K. Schaefer et al. Science Advances, 10.1126/sciadv.abc0776).

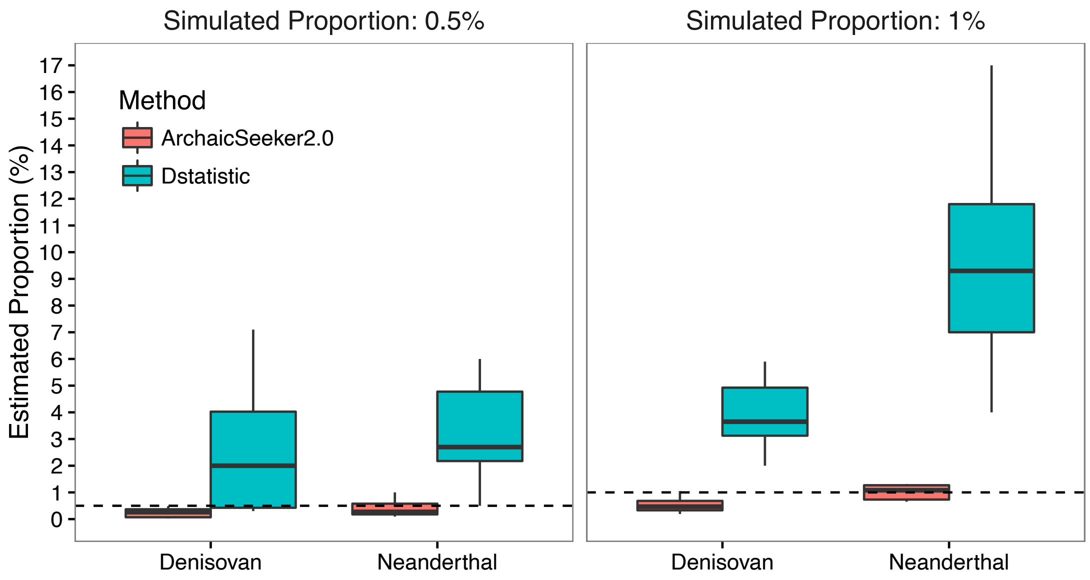

Supplementary Figure 4.45 Archaic introgression estimated by *ArchaicSeeker2.0* and *D Statistics*. Introgression proportions were estimated through the simulation data. The black dash line indicated the ground truth of introgression proportion. 100 test individuals were analyzed. Bounds of box represent the interquartile range (IQR; 25th to 75th percentile), with a center line indicating the median. Whiskers are represented in the form of Tukey style.

Supplementary Table 4.17 The mean of estimated introgression proportion under the Denisovan introgression scenario

Introgression Proportion	*ArchaicSeeker2.0*		*D statistics*
	Denisovan	Others	
0.5%	0.245%	0.171%	2.55%
1%	0.516%	0.385%	3.7%

Supplementary Table 4.18 The mean of the estimated introgression proportion under the Neanderthal introgression scenario

Introgression Proportion	*ArchaicSeeker2.0*		*D statistics*
	Neanderthal	Others	
0.5%	0.384%	0.044%	3.27%
1%	1.107%	0.066%	11.4%

We also estimated the introgression proportion with *ArchaicSeeker2.0*. The Denisovan introgression proportion is underestimated. The reason was that about half of the Denisovan introgression sequences were matched the corrected ancestry and ~ 40% were matched to other ancestries, most of which are the ancestor of Denisovan and Neanderthal. The Neanderthal introgression proportion is slightly underestimated for the "0.5% introgression scenario" and is slightly overestimated for the "1% introgression scenario". As we discussed in the supplementary (Supplementary Note 4.1, Supplementary Figure 4.26-4.32), ancestry mismatch happened more frequently in Denisovan introgressed sequences and would cause the underestimation of the Denisovan introgression proportion. Compared with the extreme overestimation of *D Statistics*, the estimation based on *ArchaicSeeker2.0* is

more reliable and accurate.

The results showed that our previous simulation studies (Supplementary Note 3, Supplementary Note 4.1-4.2) have covered the key parameters which could have substantial influences on our proposed methods in a reasonable range, and the simulation outcomes indicate that our method is less influenced by these changes, and hence the results are acceptable with different demographic parameters.

5 Supplementary Note 5: Empirical Data Analysis

We applied *ArchaicSeeker 2.0* to the worldwide population genomic data. We used our method to detect Denisovan-like and Neanderthal-like introgressed sequences and reconstruct the introgression history with these sequences. We also used an ancestry sharing statistics (see Methods) to measure the introgression history relationship among different populations. With our methods, we also detected 84 introgression desert regions, which lack of introgressed sequences.

5.1 Archaic Sequences Detection

We detected introgressed sequences of populations in 1000 Genome Phase III [4], SGDP [5] and EGDP [6]. We used Altai Neanderthal [2] and Altai Denisovan [3] as the archaic references and used YRI from 1000 Genome Project as African reference. The initial value of introgression time is 2000 generation and introgression proportion is 0.02. The matching model we used to infer modern human data is that

((YRI:100,Test:100):557.5,(Denisovan:340,Altai:300):237.5)

We also applied our method to an ancient Siberian Ust'-Ishim [1]. We used a slight different matching model to deal with that ancient sample.

((YRI:100,Ust:55):557.5,(Denisovan:340,Altai:300):237.5)

The initial value introgression time is 500 generation, since Ust'-Ishim has been dead around 1500 generations ago [1].

Denisovan-like introgression proportions ranged from 0.05 ~ 0.61% and Neanderthal-like introgressions ranged from 1.09 ~ 1.39 % among non-Africans (Table 1, Supplementary Figure 5.1 ~ 5.12, Supplementary Table 5.1 ~ 5.3).

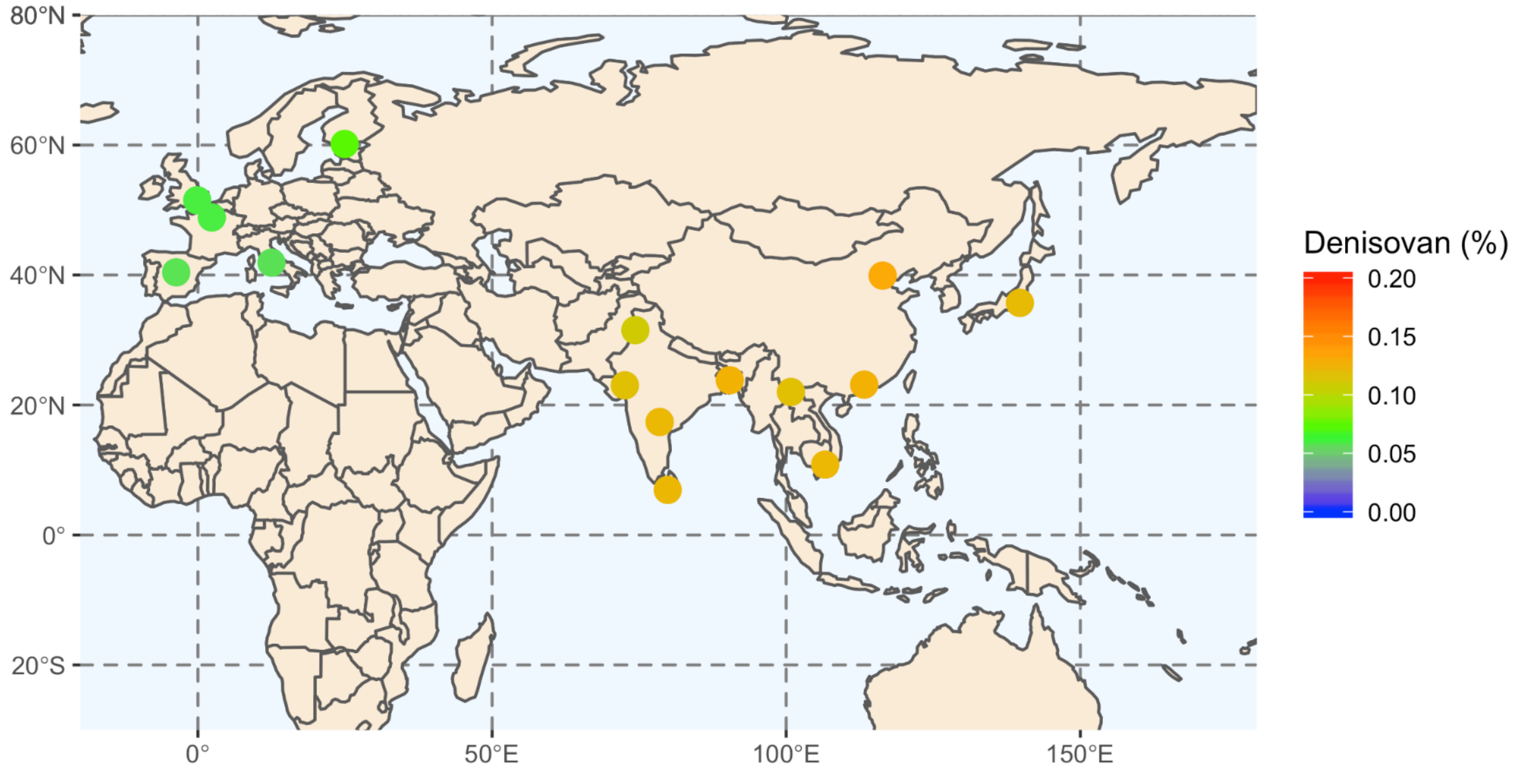

Supplementary Figure 5.1 **Geographical distribution of Denisovan introgression proportion of the 1000 Genome Project populations.** Different colors stand for different introgression proportion from Denisovan of all the non-African populations in the 1000 Genome Project. The warm color stands for higher introgression proportion and cold color stands for lower proportion.

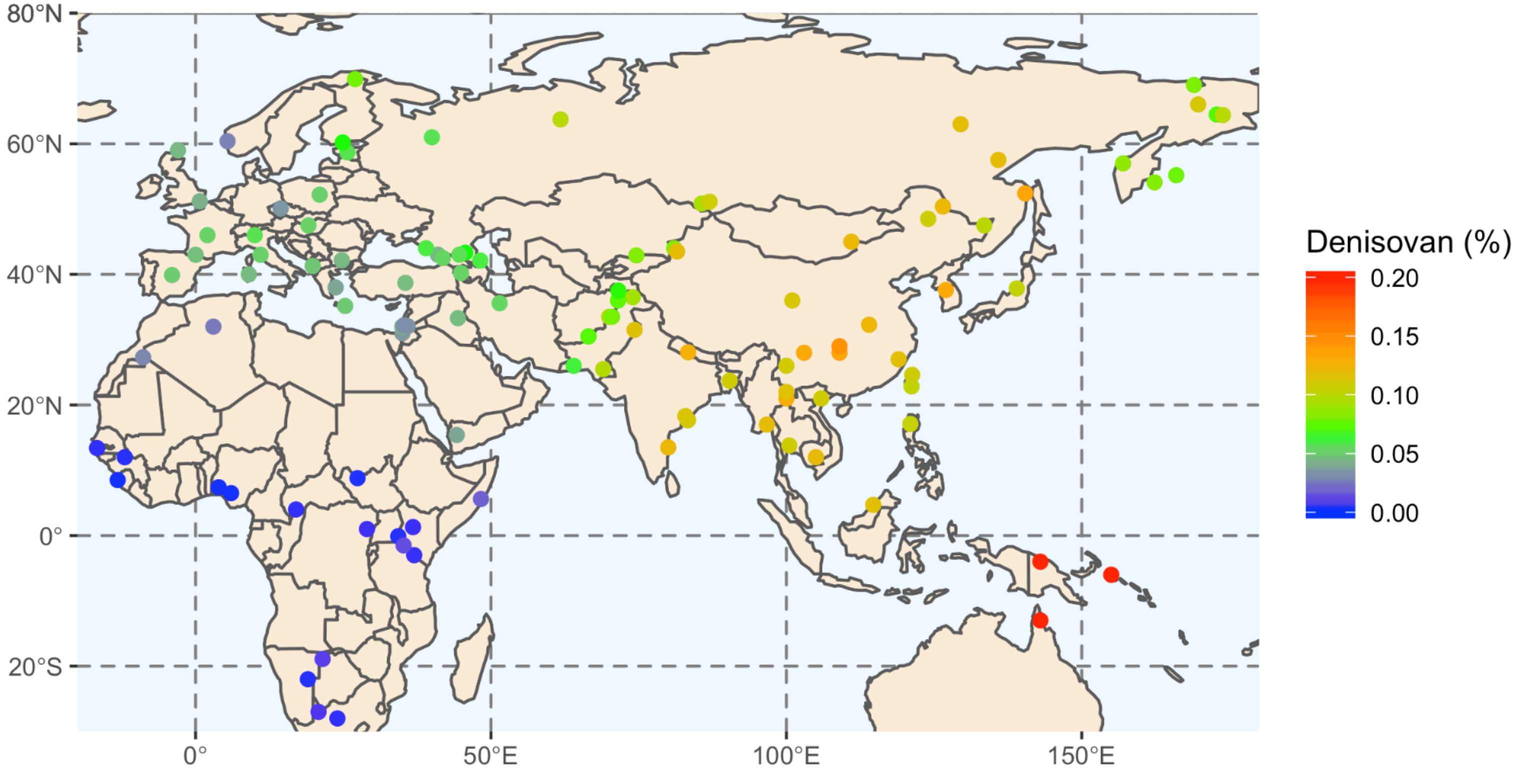

Supplementary Figure 5.2 Geographical distribution of Denisovan introgression proportion of the SGDP populations. Different colors stand for different introgression proportion from Denisovan of all the populations in the SGDP. The warm color stands for higher introgression proportion and cold color stands for lower proportion. The maximum value of the introgression was set as 0.2%. Red color stands for greater or equal to 0.2%.

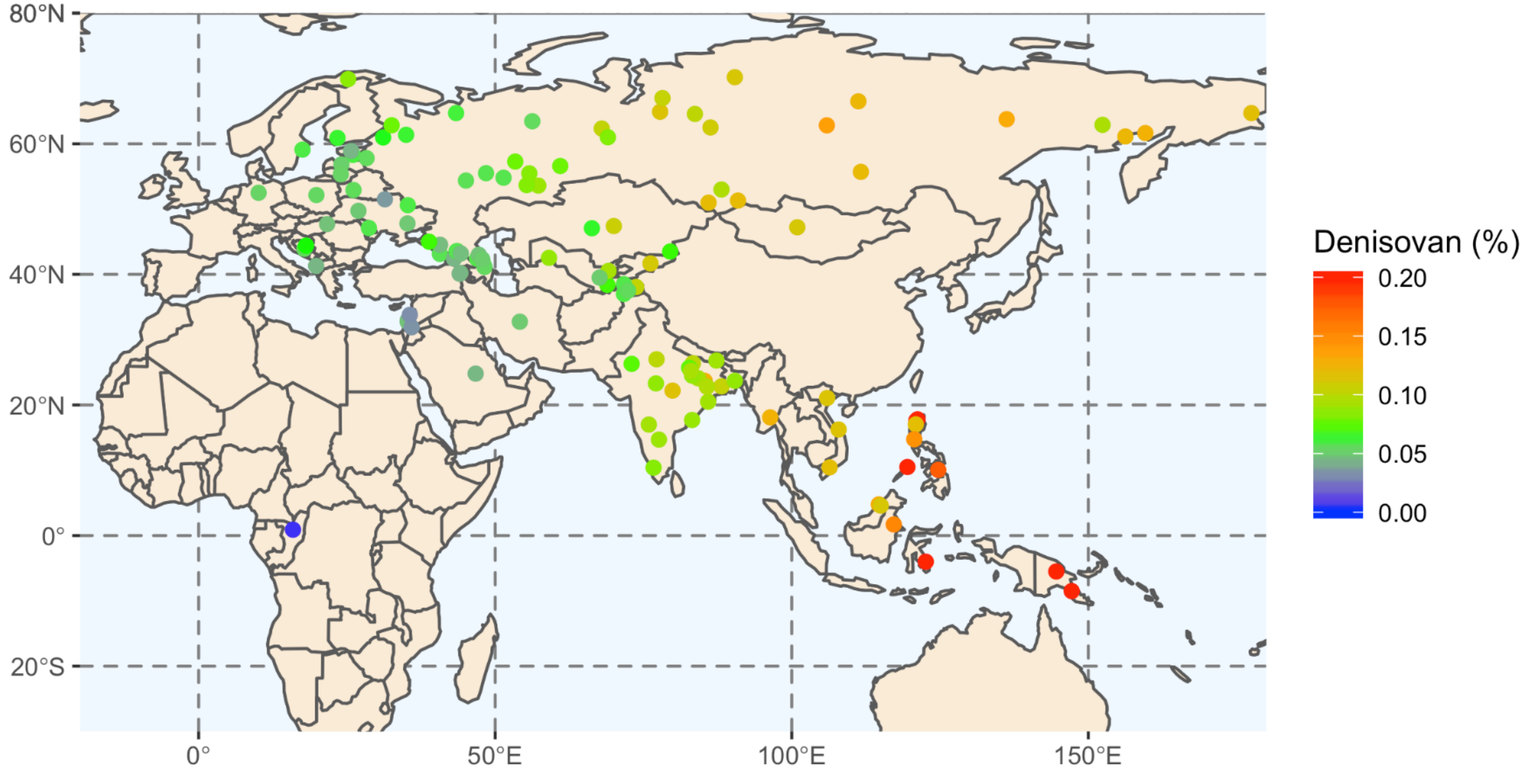

Supplementary Figure 5.3 Geographical distribution of Denisovan introgression proportion of the EGDP populations. Different colors stand for different introgression proportion from Denisovan of all the populations in the EGDP. The warm color stands for higher introgression proportion and cold color stands for lower proportion. The maximum value of the introgression was set as 0.2%. Red color stands for greater or equal to 0.2%.

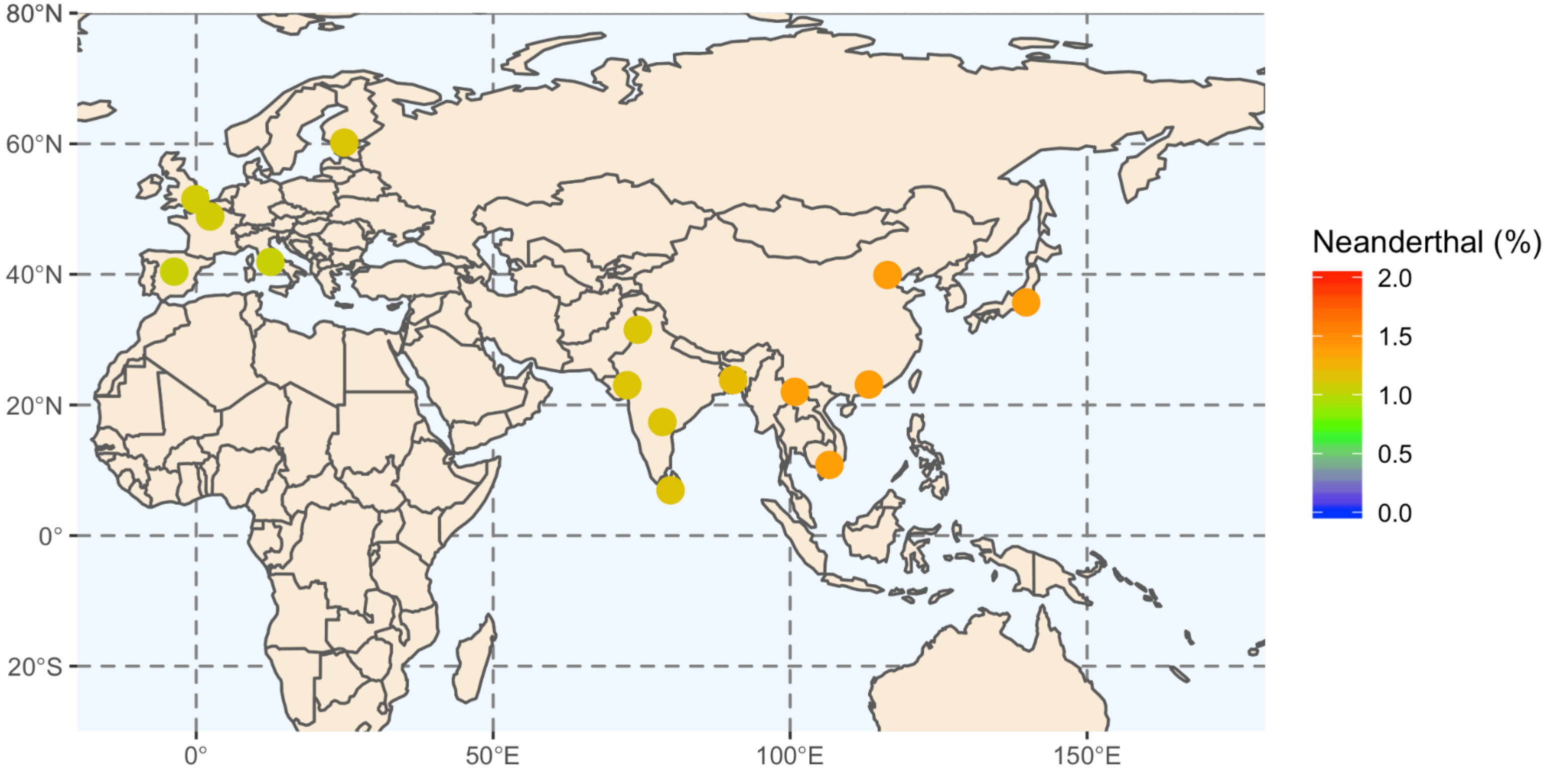

Supplementary Figure 5.4 Geographical distribution of Neanderthal introgression proportion of the 1000 Genome Project populations. Different colors stand for different introgression proportion from Neanderthal of all the non-African populations in the 1000 Genome Project. The warm color stands for higher introgression proportion and cold color stands for lower proportion.

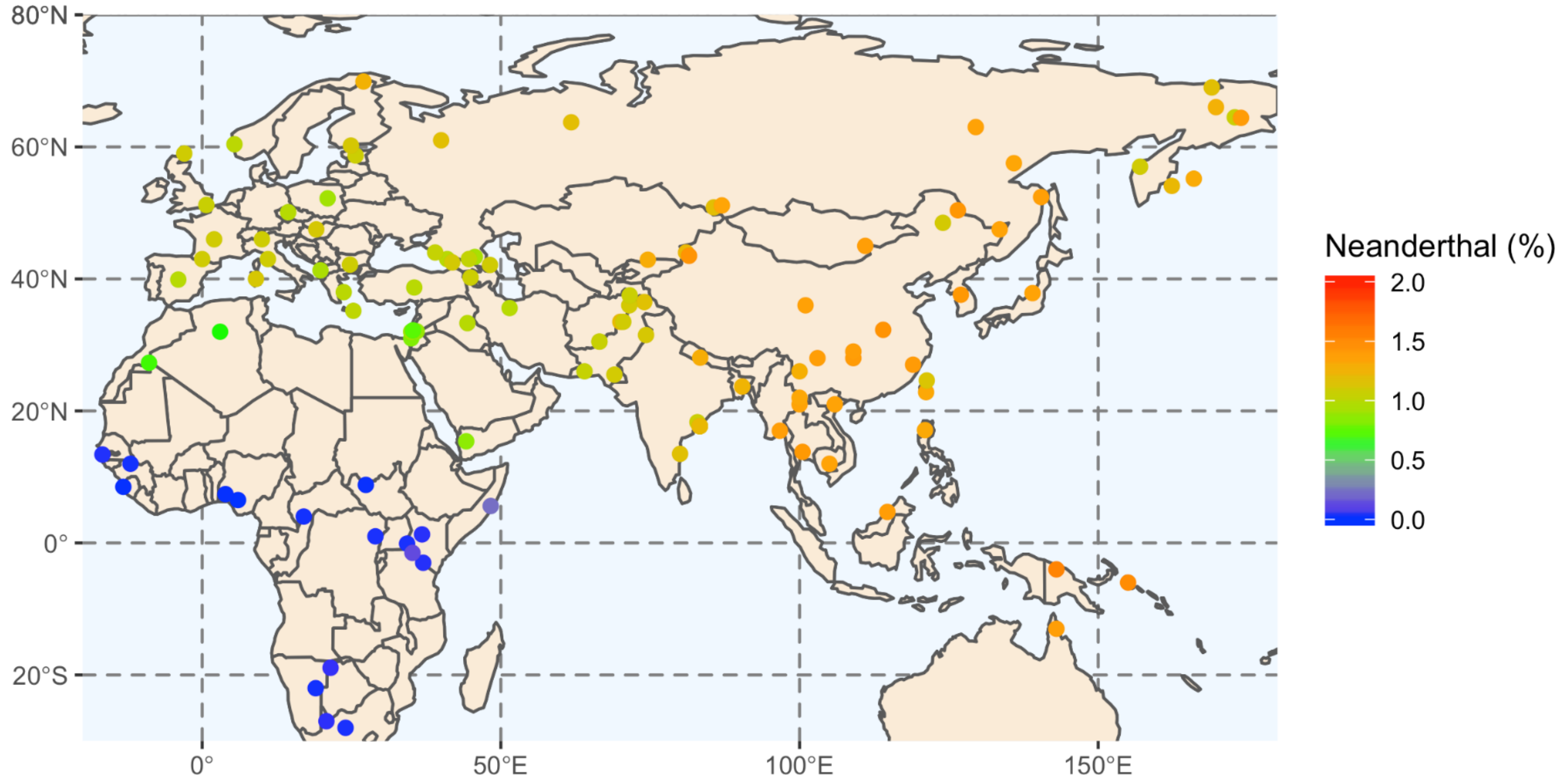

Supplementary Figure 5.5 **Geographical distribution of Neanderthal introgression proportion of the SGDP populations.** Different colors stand for different introgression proportion from Neanderthal of all the populations in the SGDP. The warm color stands for higher introgression proportion and cold color stands for lower proportion.

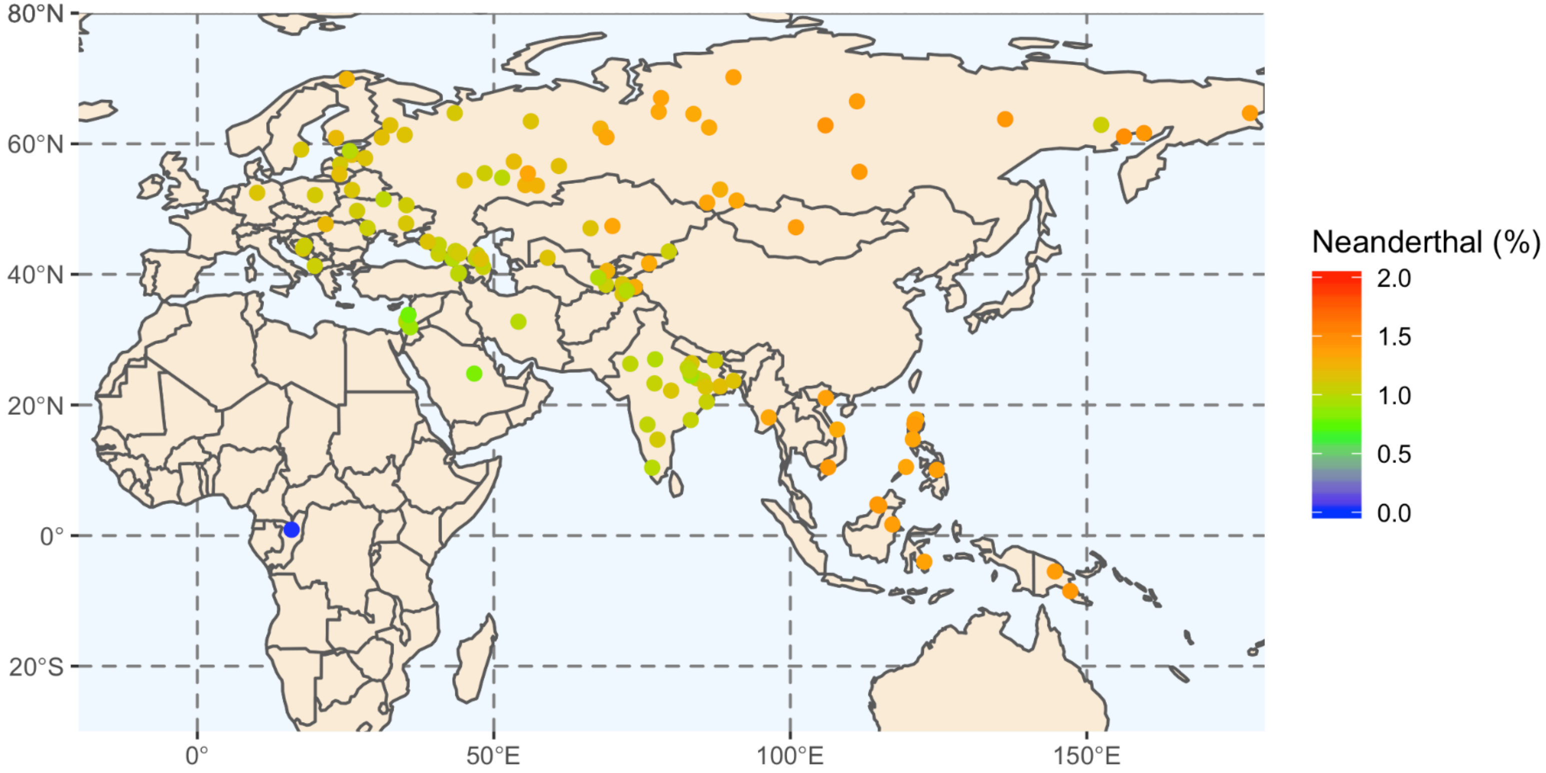

Supplementary Figure 5.6 Geographical distribution of Neanderthal introgression proportion of the EGDP populations. Different colors stand for different introgression proportion from Neanderthal of all the populations in the EGDP. The warm color stands for higher introgression proportion and cold color stands for lower proportion.

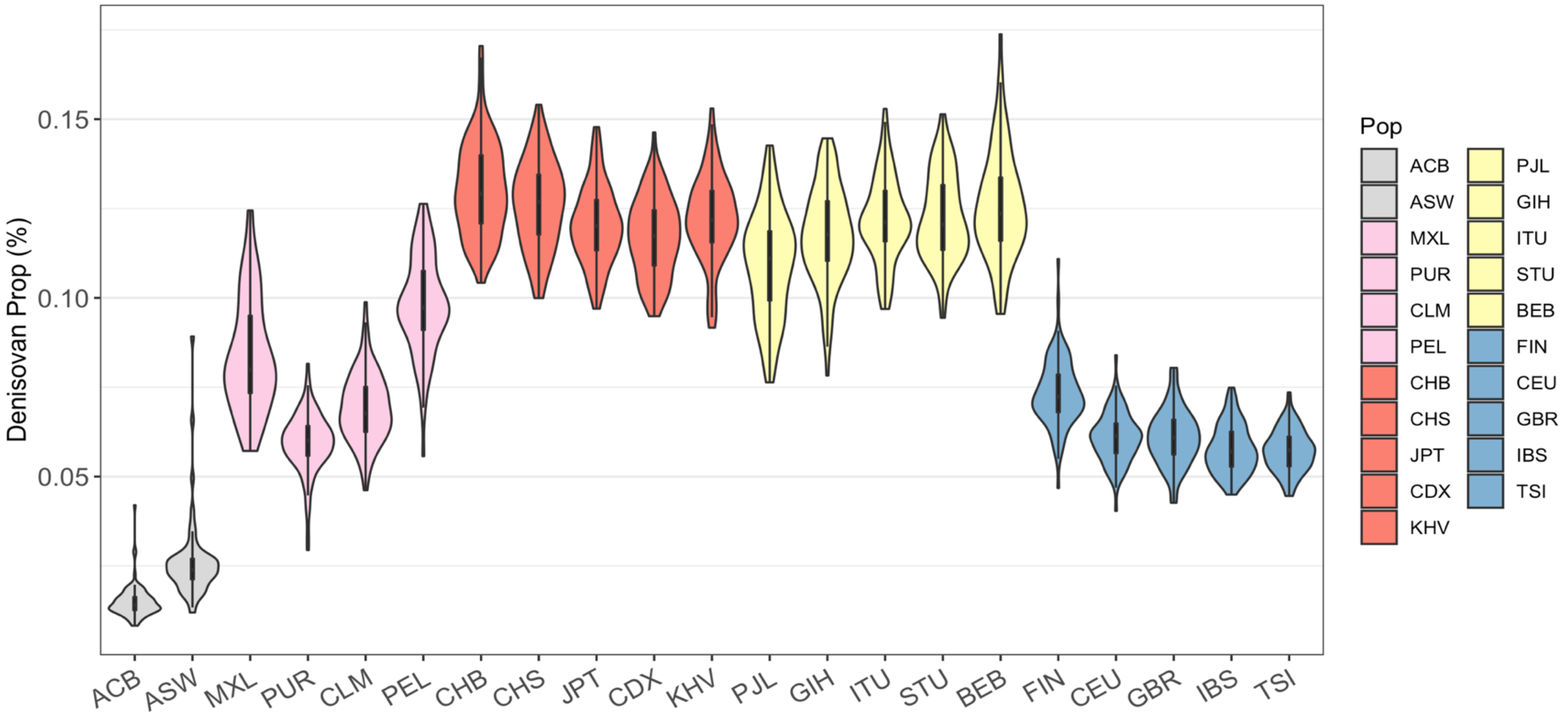

Supplementary Figure 5.7 Denisovan introgression proportion of the 1000 Genome Project populations. Different colors stand for populations from different continents or regions. The grey colored populations sampled from America, but most of the ancestries originated from Africa; the pink colored populations sampled from America; the red colored populations sampled from the East Asia; the yellow colored populations sampled from the South Asia and the blue colored populations sampled from Europe. The sample size of each population are listed as follows: ACB (African Caribbean in Barbados, 96), ASW (African Ancestry in Southwest US,

61), MXL (Mexican Ancestry in Los Angeles, California, 64), PUR (Puerto Rican in Puerto Rico, 104), CLM (Colombian in Medellin, Colombia, 94), PEL (Peruvian in Lima, Peru, 85), CHB (Han Chinese from Beijing, China, 103), CHS (Han Chinese from South China, 105), JPT (Japanese from Tokyo, Japan, 104), CDX (Chinese Dai from Xishuangbanna, China, 93), KHV (Kinh from Ho Chi Minh City, Vietnam, 99), PJL (Punjabi from Lahore, Pakistan, 96), GIH (Gujarati Indians from Houston, Texas, United States, 103), ITU (Indian Telugu from the UK, 102), STU (Sri Lankan Tamil from the UK, 102), BEB (Bengali in Bangladesh, 86), FIN (Finnish in Finland, 99), CEU (Utah residents with Northern and Western European ancestry from the CEPH collection, 99), GBR (British from England and Scotland, 91), IBS (Iberian populations in Spain, 107), TSI (Tuscans in Italy, 107). Bounds of box in the violin plot represent the interquartile range (IQR; 25th to 75th percentile), with a center line indicating the median. Whiskers are represented in the form of Tukey style.

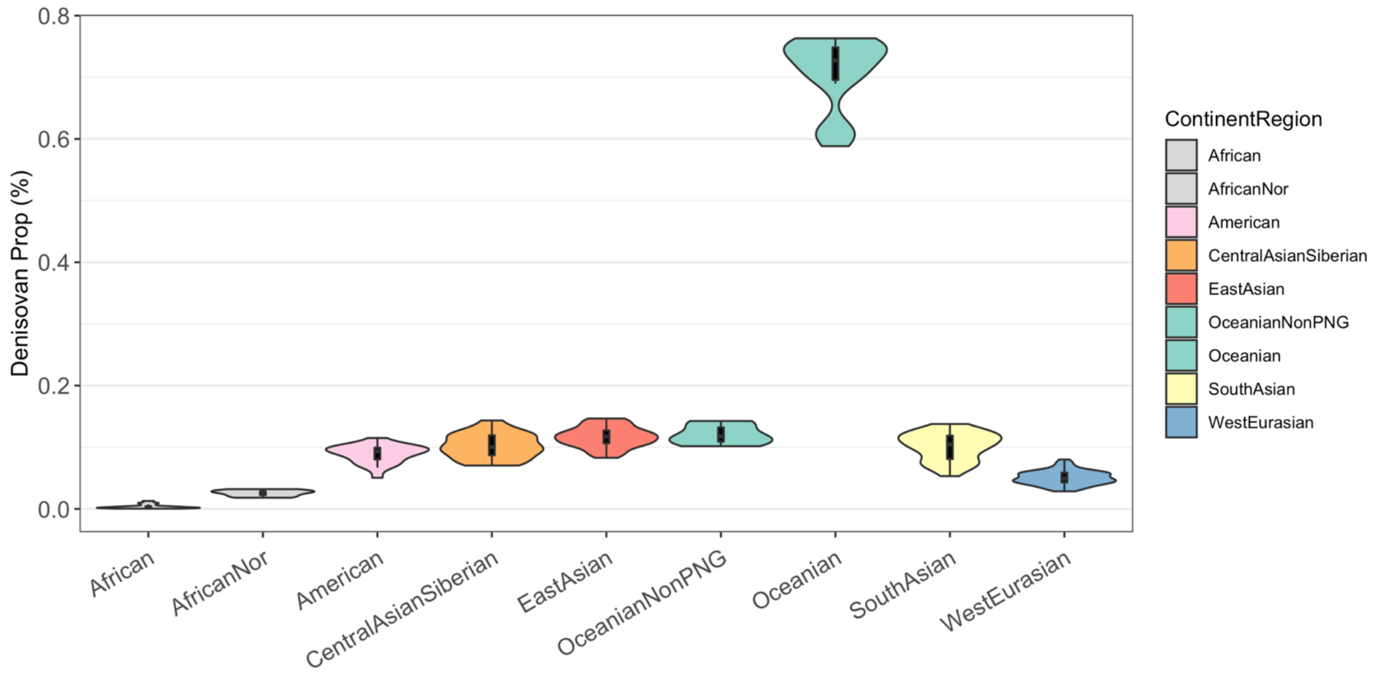

Supplementary Figure 5.8 **Denisovan introgression proportion of the SGDP populations.** Different colors stand for samples from different continents or regions. The grey colored populations sampled from Africa, which *AfricanNor* labeled population sampled from the North African. The pink colored population sampled from America. The orange colored population sampled from the Central Asia and Siberia. The red colored population sampled from the East Asia. The light blue colored populations sampled from the Oceania, which *Oceanian* labeled population sampled from the Papua New Guinea Island and Australia. The yellow colored population sampled from the South Asia and the blue colored population sampled from Europe. The sample size of each population is listed as follows: African (n=39), AfricanNor (n =5), American (n=22), CentralAsianSiberian (n=27), EastAsian (n=47), OceanianNonPNG (n=10), Oceanian (n=25), SouthAsian (n=39), WestEurasian (n=75).

Bounds of box in the violin plot represent the interquartile range (IQR; 25th to 75th percentile), with a center line indicating the median. Whiskers are represented in the form of Tukey style.

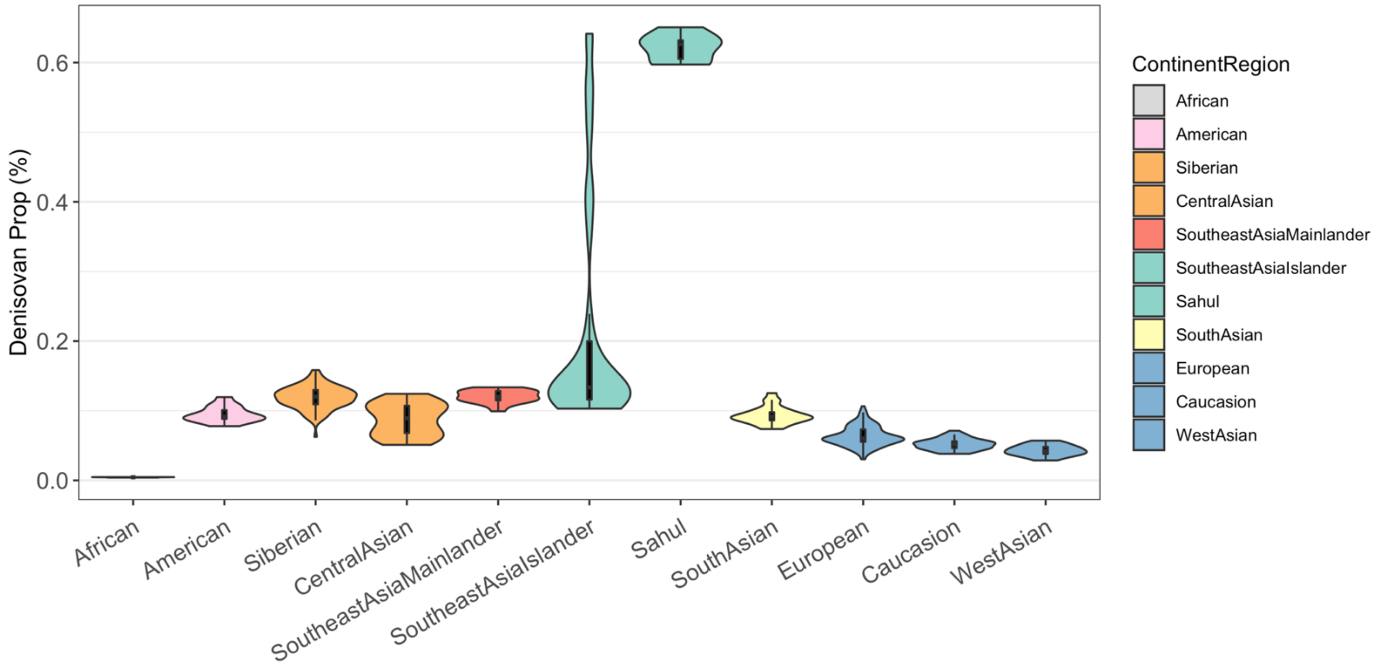

Supplementary Figure 5.9 **Denisovan introgression proportion of the EGDP populations**. Different colors stand for samples from different continents or regions. The grey colored populations sampled from Africa. The pink colored population sampled from America. The orange colored populations sampled from the Central Asia and Siberia. The red colored population sampled from the Southeast Asia Mainland. The dark blue colored populations sampled from the Sahul region (Papua New Guinea Island and Australia) and Southeast Asia Island. The yellow colored population sampled from the South Asia and the blue colored populations sampled from Europe Caucus and West Asia. The sample size of each population are listed as follows: African (n=3), American (n=13), Siberian (n=108), Central Asian (n=24), Southeast Asia Mainlander (n=18), Southeast Asian Islander (n=45), Sahul (n=6), South Asian (n=25), European (n=101), Caucasian (n=39), West Asian (n=20).

Bounds of box in the violin plot represent the interquartile range (IQR; 25th to 75th percentile), with a center line indicating the median. Whiskers are represented in the form of Tukey style.

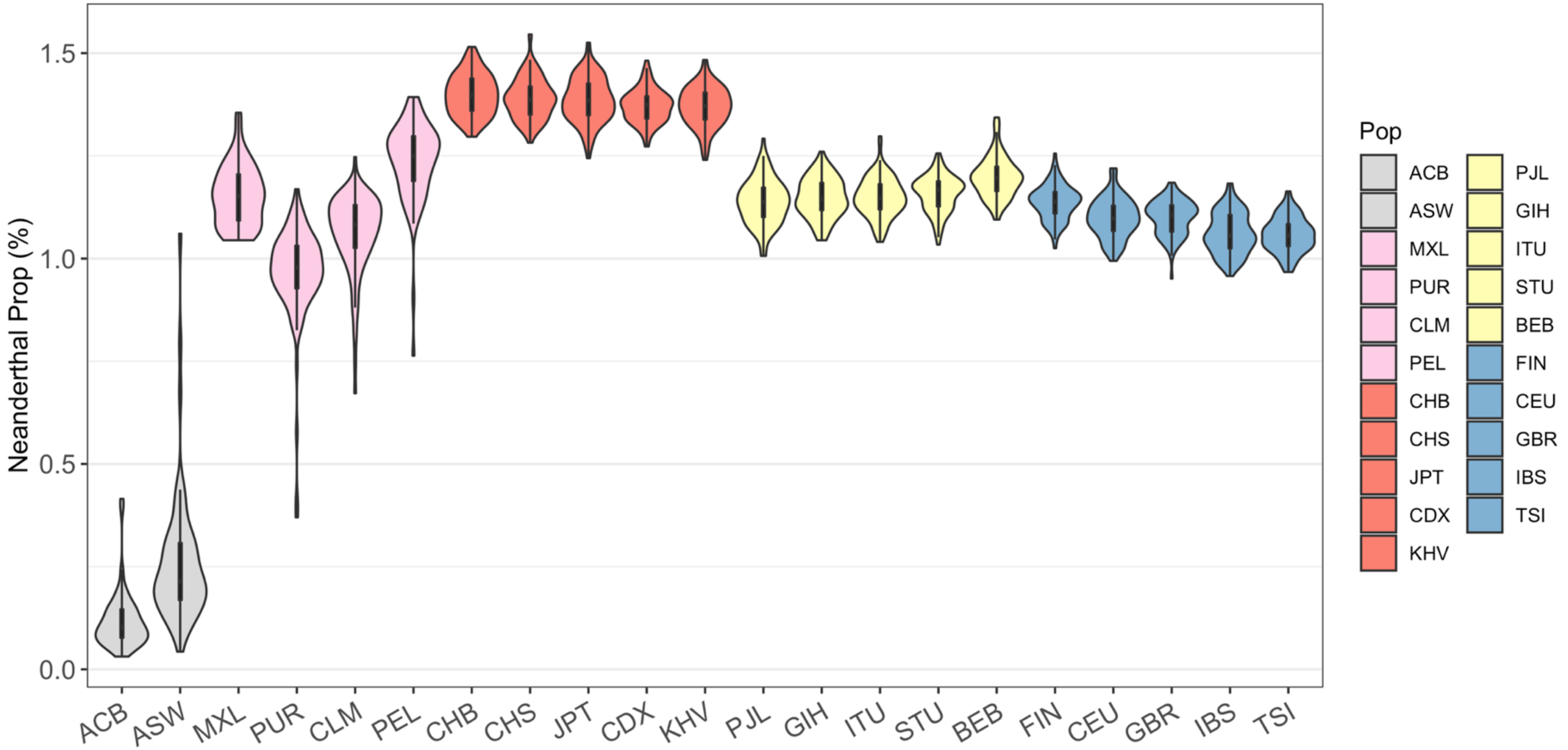

Supplementary Figure 5.10 Neanderthal introgression proportion of the 1000 Genome Project populations. Different colors stand for populations from different continents or regions. The grey colored populations sampled from America, but most of the ancestries originated from Africa; the pink colored populations sampled from America; the red colored populations sampled from the East Asia; the yellow colored populations sampled from the South Asia and the blue colored populations sampled from Europe. The sample size of each population are listed as follows: ACB (African Caribbean in Barbados, 96), ASW (African Ancestry in Southwest US,

61), MXL (Mexican Ancestry in Los Angeles, California, 64), PUR (Puerto Rican in Puerto Rico, 104), CLM (Colombian in Medellin, Colombia, 94), PEL (Peruvian in Lima, Peru, 85), CHB (Han Chinese from Beijing, China, 103), CHS (Han Chinese from South China, 105), JPT (Japanese from Tokyo, Japan, 104), CDX (Chinese Dai from Xishuangbanna, China, 93), KHV (Kinh from Ho Chi Minh City, Vietnam, 99), PJL (Punjabi from Lahore, Pakistan, 96), GIH (Gujarati Indians from Houston, Texas, United States, 103), ITU (Indian Telugu from the UK, 102), STU (Sri Lankan Tamil from the UK, 102), BEB (Bengali in Bangladesh, 86), FIN (Finnish in Finland, 99), CEU (Utah residents with Northern and Western European ancestry from the CEPH collection, 99), GBR (British from England and Scotland, 91), IBS (Iberian populations in Spain, 107), TSI (Tuscans in Italy, 107). Bounds of box in the violin plot represent the interquartile range (IQR; 25th to 75th percentile), with a center line indicating the median. Whiskers are represented in the form of Tukey style.

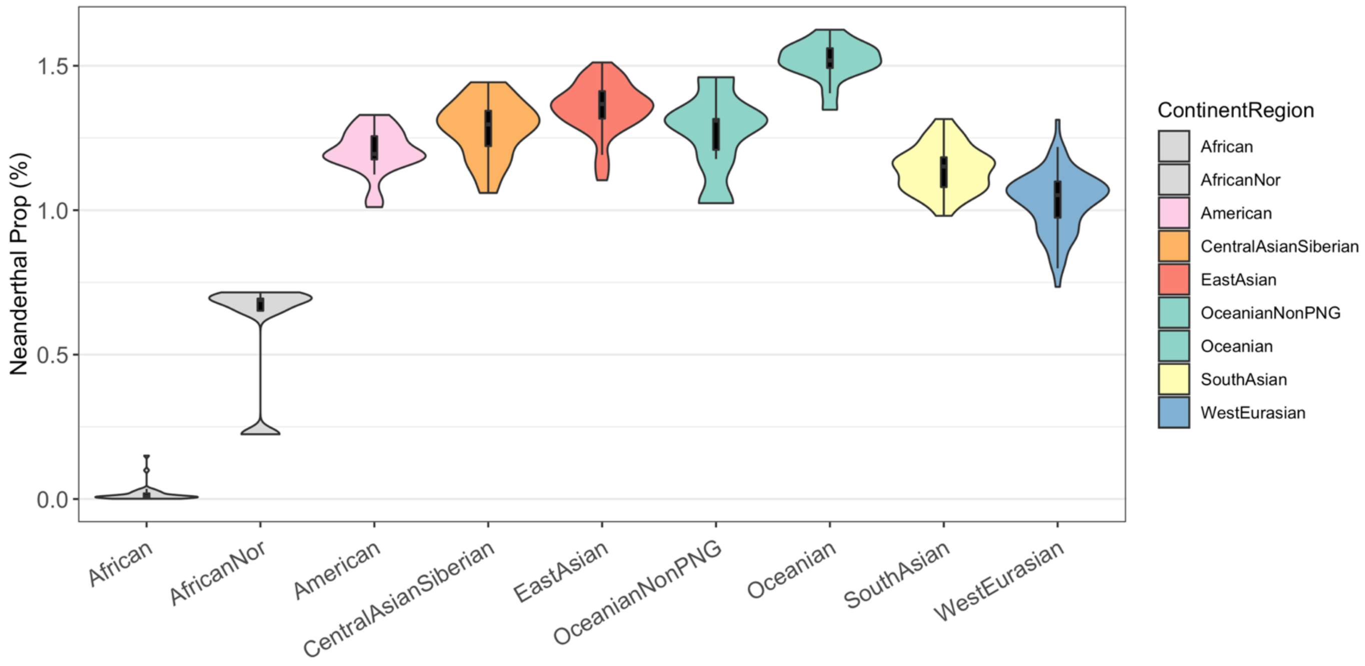

Supplementary Figure 5.11 Neanderthal introgression proportion of the SGDP populations. Different colors stand for samples from different continents or regions. The grey colored populations sampled from Africa, which *AfricanNor* labeled population sampled from the North African. The pink colored population sampled from America. The orange colored population sampled from the Central Asia and Siberia. The red colored population sampled from the East Asia. The light blue colored populations sampled from the Oceania, which *Oceanian* labeled population sampled from the Papua New Guinea Island and Australia. The yellow colored population sampled from the South Asia and the blue colored population sampled from Europe. The sample size of each population is listed as follows: African (n=39), AfricanNor (n =5), American (n=22), CentralAsianSiberian (n=27), EastAsian (n=47), OceanianNonPNG (n=10), Oceanian (n=25), SouthAsian (n=39), WestEurasian (n=75).

Bounds of box in the violin plot represent the interquartile range (IQR; 25th to 75th percentile), with a center line indicating the median. Whiskers are represented in the form of Tukey style.

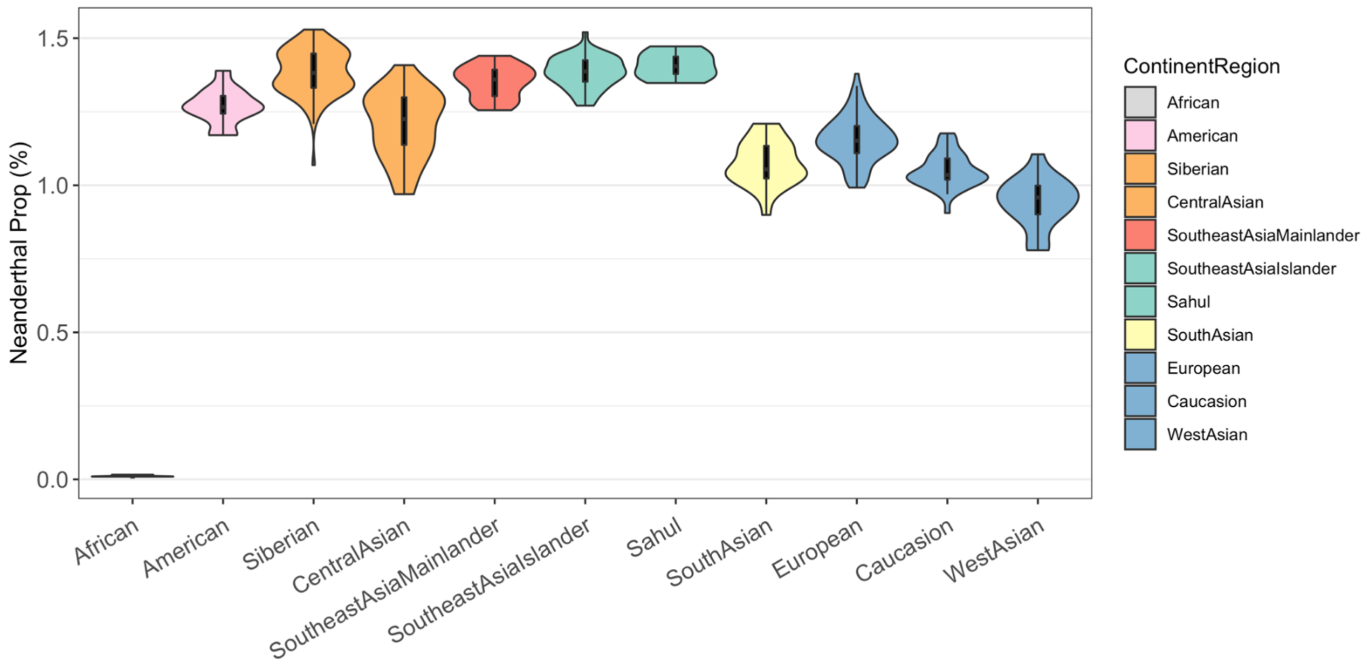

Supplementary Figure 5.12 Neanderthal introgression proportion of the EGDP populations.

Different colors stand for samples from different continents or regions. The grey colored populations sampled from Africa. The pink colored population sampled from America. The orange colored populations sampled from the Central Asia and Siberia. The red colored population sampled from the Southeast Asia Mainland. The dark blue colored populations sampled from the Sahul region (Papua New Guinea Island and Australia) and Southeast Asia Island. The yellow colored population sampled from the South Asia and the blue colored populations sampled from Europe Caucus and West Asia. The sample size of each population is listed as follows: African (n=3), American (n=13), Siberian (n=108), Central Asian (n=24), Southeast Asia Mainlander (n=18), Southeast Asian Islander (n=45), Sahul (n=6), South Asian (n=25),

European (n=101), Caucasian (n=39), West Asian (n=20). Bounds of box in the violin plot represent the interquartile range (IQR; 25th to 75th percentile), with a center line indicating the median. Whiskers are represented in the form of Tukey style.

Supplementary Table 5.1 **Introgression proportion of populations in SGDP**

Population Label	Continent/Region	Denisovan	Neanderthal
BantuHerero	Africa	0.00%	0.01%
BantuKenya	Africa	0.00%	0.02%
BantuTswana	Africa	0.00%	0.01%
Biaka	Africa	0.00%	0.01%
Dinka	Africa	0.00%	0.00%
Esan	Africa	0.00%	0.00%
Gambia	Africa	0.00%	0.01%
Ju_hoan_North	Africa	0.01%	0.03%
Khomani_San	Africa	0.01%	0.02%
Luhya	Africa	0.00%	0.02%
Luo	Africa	0.00%	0.01%
Mandenka	Africa	0.00%	0.01%
Masai	Africa	0.01%	0.12%
Mbuti	Africa	0.00%	0.01%
Mende	Africa	0.00%	0.00%
Yoruba	Africa	0.00%	0.00%
Mozabite	North Africa	0.03%	0.67%
Saharawi	North Africa	0.03%	0.70%
Somali	North Africa	0.02%	0.22%
Chane	America	0.05%	1.01%
Karitiaa	America	0.09%	1.18%
Mayan	America	0.10%	1.26%
Mixe	America	0.10%	1.24%
Mixtec	America	0.09%	1.25%
Piapoco	America	0.10%	1.15%
Pima	America	0.08%	1.23%
Quechua	America	0.09%	1.25%
Surui	America	0.07%	1.11%
Zapotec	America	0.10%	1.23%
Aleut	Central Asia Siberia	0.08%	1.30%
Altaia	Central Asia Siberia	0.09%	1.19%
Chukchi	Central Asia Siberia	0.08%	1.17%
Eskimo_Chaplin	Central Asia Siberia	0.07%	1.06%
Eskimo_Naukan	Central Asia Siberia	0.11%	1.26%
Eskimo_Sireniki	Central Asia Siberia	0.10%	1.39%
Even	Central Asia Siberia	0.12%	1.33%
Itelman	Central Asia Siberia	0.09%	1.09%
Kyrgyz	Central Asia Siberia	0.08%	1.29%
Mansi	Central Asia Siberia	0.10%	1.18%
Mongola	Central Asia Siberia	0.12%	1.38%
Tlingit	Central Asia Siberia	0.08%	1.22%
Tubalar	Central Asia Siberia	0.11%	1.33%
Ulchi	Central Asia Siberia	0.13%	1.37%
Yakut	Central Asia Siberia	0.12%	1.36%
Ami	East Asia	0.10%	1.32%

Population Label	Continent/Region	Denisovan	Neanderthal
Atayal	East Asia	0.11%	1.13%
Burmese	East Asia	0.12%	1.38%
Cambodia	East Asia	0.12%	1.35%
Dai	East Asia	0.13%	1.35%
Daur	East Asia	0.11%	1.10%
Han	East Asia	0.12%	1.45%
Hezhen	East Asia	0.10%	1.35%
Japanese	East Asia	0.10%	1.35%
Kinh	East Asia	0.11%	1.35%
Korean	East Asia	0.13%	1.42%
Lahu	East Asia	0.12%	1.32%
Miao	East Asia	0.13%	1.40%
Naxi	East Asia	0.11%	1.27%
Oroqen	East Asia	0.12%	1.43%
She	East Asia	0.12%	1.40%
Thai	East Asia	0.11%	1.41%
Tu	East Asia	0.11%	1.36%
Tujia	East Asia	0.14%	1.38%
Uygur	East Asia	0.08%	1.22%
Xibo	East Asia	0.12%	1.45%
Yi	East Asia	0.13%	1.38%
Australia	Oceania	0.60%	1.38%
Bougainville	Oceania	0.61%	1.50%
Papuan	Oceania	0.73%	1.54%
Dusun	Oceania	0.12%	1.39%
Hawaiia	Oceania	0.14%	1.02%
Igorot	Oceania	0.10%	1.31%
Maori	Oceania	0.14%	1.18%
Balochi	South Asia	0.06%	1.08%
Bengali	South Asia	0.11%	1.24%
Brahmin	South Asia	0.11%	1.14%
Brahui	South Asia	0.07%	1.05%
Burusho	South Asia	0.09%	1.16%
Hazara	South Asia	0.10%	1.20%
Irula	South Asia	0.12%	1.16%
Kalash	South Asia	0.07%	1.10%
Kapu	South Asia	0.11%	1.13%
Khonda_Dora	South Asia	0.11%	1.08%
Kusunda	South Asia	0.13%	1.28%
Madiga	South Asia	0.12%	1.17%
Makrani	South Asia	0.06%	1.05%
Mala	South Asia	0.12%	1.16%
Pathan	South Asia	0.08%	1.09%
Punjabi	South Asia	0.12%	1.12%
Relli	South Asia	0.13%	1.16%
Sindhi	South Asia	0.10%	1.10%

Population Label	Continent/Region	Denisovan	Neanderthal
Yadava	South Asia	0.11%	1.22%
Abkhasia	West Eurasia	0.04%	0.99%
Adygei	West Eurasia	0.06%	1.05%
Albania	West Eurasia	0.05%	0.94%
Armenia	West Eurasia	0.05%	1.08%
Basque	West Eurasia	0.05%	1.09%
BedouinB	West Eurasia	0.04%	0.81%
Bergamo	West Eurasia	0.06%	1.08%
Bulgaria	West Eurasia	0.04%	1.08%
Chechen	West Eurasia	0.07%	0.93%
Crete	West Eurasia	0.05%	1.01%
Czech	West Eurasia	0.03%	0.98%
Druze	West Eurasia	0.05%	0.94%
English	West Eurasia	0.04%	1.06%
Estonia	West Eurasia	0.06%	1.08%
Finnish	West Eurasia	0.07%	1.12%
French	West Eurasia	0.05%	1.09%
Georgia	West Eurasia	0.05%	1.10%
Greek	West Eurasia	0.04%	1.00%
Hungaria	West Eurasia	0.05%	1.12%
Icelandic	West Eurasia	0.06%	1.14%
Irania	West Eurasia	0.05%	0.99%
Iraqi_Jew	West Eurasia	0.05%	0.99%
Jordania	West Eurasia	0.03%	0.87%
Lezgin	West Eurasia	0.06%	1.09%
North_Ossetia	West Eurasia	0.06%	1.03%
Norwegia	West Eurasia	0.03%	1.00%
Orcadia	West Eurasia	0.04%	1.09%
Palestinia	West Eurasia	0.04%	0.91%
Polish	West Eurasia	0.05%	0.91%
Russia	West Eurasia	0.06%	1.17%
Saami	West Eurasia	0.08%	1.26%
Samaritan	West Eurasia	0.03%	0.73%
Sardinia	West Eurasia	0.05%	1.12%
Spanish	West Eurasia	0.05%	0.99%
Tajik	West Eurasia	0.07%	1.06%
Turkish	West Eurasia	0.05%	1.00%
Tusca	West Eurasia	0.05%	1.09%
Yemenite_Jew	West Eurasia	0.04%	0.85%

Supplementary Table 5.2 Introgression proportion of populations in EGDP

Population Label	Continent/Region	Denisovan	Neanderthal
Congo-pygmies	Africa	0.00%	0.01%
Cachi	America	0.10%	1.31%
Colla	America	0.10%	1.27%

Population Label	Continent/Region	Denisovan	Neanderthal
Wichi	America	0.09%	1.21%
Abkhazias	Caucasus	0.06%	1.06%
Armenias	Caucasus	0.05%	1.00%
Avars	Caucasus	0.06%	1.03%
Azerbaijanis	Caucasus	0.06%	1.06%
Balkars	Caucasus	0.05%	1.04%
Circassias	Caucasus	0.05%	1.04%
Georgias	Caucasus	0.04%	0.98%
Kabardins	Caucasus	0.06%	1.07%
Kumyks	Caucasus	0.05%	1.09%
Lezgins	Caucasus	0.05%	1.01%
North-Ossetias	Caucasus	0.05%	1.10%
Tabasarans	Caucasus	0.05%	1.16%
Ishkasim	Central Asia	0.06%	1.14%
Kazakhs	Central Asia	0.11%	1.33%
Kyrgyz	Central Asia	0.11%	1.30%
KyrgyzTdj	Central Asia	0.10%	1.29%
Rushan-Vanch	Central Asia	0.06%	1.13%
Shugnan	Central Asia	0.06%	0.99%
Tajiks	Central Asia	0.07%	1.06%
Turkmens	Central Asia	0.09%	1.15%
Uygurs	Central Asia	0.07%	1.07%
Uzbek	Central Asia	0.09%	1.25%
Yaghnobi	Central Asia	0.05%	0.97%
Albanias	Europe	0.04%	1.07%
Bashkirs	Europe	0.09%	1.26%
Belarusias	Europe	0.06%	1.13%
Chuvashes	Europe	0.08%	1.26%
Cossacks	Europe	0.05%	1.13%
CossacksKuban	Europe	0.07%	1.15%
Croats	Europe	0.06%	1.12%
Estonias	Europe	0.06%	1.17%
Finnish	Europe	0.06%	1.22%
Germans	Europe	0.05%	1.14%
Hungarias	Europe	0.05%	1.22%
Ingrias	Europe	0.07%	1.18%
Karelias	Europe	0.08%	1.16%
Komis	Europe	0.06%	1.15%
Kryashen-Tatars	Europe	0.08%	1.18%
Latvias	Europe	0.05%	1.10%
Lithuanias	Europe	0.05%	1.18%
Maris	Europe	0.08%	1.33%
Mishar-Tatars	Europe	0.06%	1.00%
Moldavias	Europe	0.06%	1.07%
Mordvins	Europe	0.06%	1.17%
Poles	Europe	0.05%	1.11%

Population Label	Continent/Region	Denisovan	Neanderthal
Roma	Europe	0.07%	1.07%
Russias	Europe	0.04%	0.99%
Russias-Central	Europe	0.06%	1.08%
Russias-North	Europe	0.06%	1.12%
Russias-West	Europe	0.06%	1.15%
Saami	Europe	0.08%	1.24%
Swedes	Europe	0.06%	1.13%
Tatars	Europe	0.06%	1.15%
Udmurds	Europe	0.08%	1.20%
Ukrainiaseast	Europe	0.06%	1.09%
Ukrainiasnorth	Europe	0.04%	1.02%
Ukrainiaswest	Europe	0.05%	1.09%
Vepsas	Europe	0.06%	1.15%
Koinanbe	Sahul	0.61%	1.39%
Kosipe	Sahul	0.63%	1.42%
Altaias	Siberia	0.12%	1.34%
Buryats	Siberia	0.12%	1.41%
Chukchis	Siberia	0.12%	1.39%
Eskimo	Siberia	0.12%	1.32%
Evenks	Siberia	0.14%	1.45%
EvensMagadan	Siberia	0.12%	1.48%
EvensSakha	Siberia	0.13%	1.43%
Forest-Nenets	Siberia	0.11%	1.32%
Kets	Siberia	0.11%	1.31%
Khantys	Siberia	0.10%	1.27%
Koryaks	Siberia	0.13%	1.43%
Mansis	Siberia	0.08%	1.32%
Mongolias	Siberia	0.11%	1.39%
Nganasans	Siberia	0.11%	1.36%
Sakha	Siberia	0.12%	1.39%
Selkups	Siberia	0.10%	1.31%
Shor	Siberia	0.10%	1.28%
Tundra-Nenets	Siberia	0.10%	1.35%
Tuvinias	Siberia	0.12%	1.32%
Yakuts	Siberia	0.09%	1.07%
Asur	South Asia	0.13%	1.04%
Balija	South Asia	0.09%	1.10%
Bengali	South Asia	0.10%	1.18%
Brahmin	South Asia	0.10%	1.13%
Dhaka-mixed-popul	South Asia	0.09%	1.15%
Gond	South Asia	0.12%	1.13%
Gupta	South Asia	0.08%	1.02%
Ho	South Asia	0.09%	1.05%
Kapu	South Asia	0.09%	1.04%
Kol	South Asia	0.09%	0.90%
Kshatriya	South Asia	0.08%	1.04%

Population Label	Continent/Region	Denisovan	Neanderthal
Kurmi	South Asia	0.09%	1.02%
Madhya-Pradesh	South Asia	0.09%	1.04%
Malayan	South Asia	0.08%	0.99%
Marwadi	South Asia	0.07%	1.02%
Orissa	South Asia	0.09%	1.05%
Punjab	South Asia	0.09%	1.02%
Santhal	South Asia	0.10%	1.13%
Tamang	South Asia	0.09%	1.08%
Thakur	South Asia	0.10%	0.98%
Aeta	Southeast Asia Island	0.56%	1.37%
Agta	Southeast Asia Island	0.59%	1.32%
Bajo	Southeast Asia Island	0.24%	1.35%
Batak	Southeast Asia Island	0.41%	1.34%
Dusun	Southeast Asia Island	0.13%	1.43%
Igorot	Southeast Asia Island	0.12%	1.39%
Lebbo	Southeast Asia Island	0.15%	1.38%
Luzon	Southeast Asia Island	0.15%	1.34%
Murut	Southeast Asia Island	0.11%	1.41%
Vizayan	Southeast Asia Island	0.18%	1.37%
Burmese	Southeast Asia Mainland	0.13%	1.32%
Vietnamesecentral	Southeast Asia Mainland	0.11%	1.31%
Vietnamesenorth	Southeast Asia Mainland	0.11%	1.36%
Vietnamesesouth	Southeast Asia Mainland	0.12%	1.41%
Arabs-Israel-1	West Asia	0.04%	0.95%
Arabs-Israel-2	West Asia	0.04%	0.95%
Assyrias	West Asia	0.04%	1.01%
Druze	West Asia	0.05%	0.98%
Iranias	West Asia	0.05%	0.99%
Jordanias	West Asia	0.03%	0.90%
Lebanese	West Asia	0.03%	0.78%
Saudi-Arabias	West Asia	0.04%	0.81%

Supplementary Table 5.3 Introgression proportion of populations in KGP

Population Label	Continent/Region	Denisovan	Neanderthal
ACB	America	0.01%	0.12%
ASW	America	0.03%	0.26%
CLM	America	0.07%	1.06%
MXL	America	0.08%	1.15%
PEL	America	0.10%	1.24%
PUR	America	0.06%	0.97%
CDX	East Asia	0.12%	1.37%
CHB	East Asia	0.13%	1.40%
CHS	East Asia	0.13%	1.39%
JPT	East Asia	0.12%	1.39%

KHV	East Asia	0.12%	1.37%
CEU	Europe	0.06%	1.10%
FIN	Europe	0.07%	1.14%
GBR	Europe	0.06%	1.10%
IBS	Europe	0.06%	1.06%
TSI	Europe	0.06%	1.06%
BEB	South Asia	0.13%	1.20%
GIH	South Asia	0.12%	1.15%
ITU	South Asia	0.12%	1.15%
PJL	South Asia	0.11%	1.14%
STU	South Asia	0.12%	1.16%

We set 2% as the minimum population introgression threshold to calculate the worldwide introgression coverage. We detected 451.3 Mb genomes covered by Neanderthal-like introgression sequences from East Asian; 439.5 Mb from European; 542.7 Mb from South Asian and 355.3 Mb from Papuan. We also detected 45.2 Mb genomes covered by Denisovan-like introgression sequences from East Asian; 22.4 Mb from European; 51.5 Mb from South Asian and 212.9 Mb from Papuan (Figure 2).

5.2 Archaic Introgression Model Inference

With our method, we inferred the introgression model of Eurasian populations from 1000 Genome Project and Papuan from SGDP. We also tried to reconstruct the admixture history of Ust'-Ishim. Denisovan-like and Neanderthal-like introgressed sequences were analyzed separately. Segments smaller than 0.015 cM (15kb) were removed before the analyses. We performed 100 times bootstrapping for each analysis and selected the best-supported model as the final output (Supplementary Table 5.4 ~ 5.5).

For Denisovan-like introgression, we detected two introgression events in Eurasians except IBS (Supplementary Table 5.4). The Ancient wave of Eurasians happened around 118.8 ~ 94.0 kya (Table 2, Supplementary Table 5.4). The recent event happened 48.1 ~ 37.5 kya in East Asians; 56.7 ~ 47.8 kya in South Asian. For Europeans, we detected a weak introgression event (~ 0.01%) in four of the five European 1000 Genome Project (Supplementary Table 5.4). We didn't detect the recent wave in the most west population, IBS. Together with the following ancestry sharing analyses, the recent introgression wave in Europeans should come from the recent Asian gene flow from the east (Supplementary Note 5.3). We detected one introgression event in Papuan, which took place 64.0 ~ 61.9 kya (Table 2, Supplementary Table 5.4).

For Neanderthal-like introgression, two introgression events were found in all the populations. The ancient happened around 61.7 ~ 49.1 kya and the recent happened around 37.5 ~ 28.9 kya (Table 2, Supplementary Table 5.5).

Supplementary Table 5.4 **Denisovan-like introgression models**

Populations	Number of Introgression	Support Ratio	Time* (Proportion)		Time (Proportion) 95% CI	
CDX	2	100%	1250.42 (0.04%)	3133.46 (0.08%)	1137.93 ~ 1375.12 (0.032 ~ 0.045 %)	2977.44 ~ 3275.85 (0.073 ~ 0.087 %)
CHB	2	100%	1344.19 (0.06%)	3167.26 (0.08%)	1227.11 ~ 1468.59 (0.048 ~ 0.066 %)	3007.05 ~ 3353.21 (0.066 ~ 0.084 %)
CHS	2	100%	1352.48 (0.06%)	3397.88 (0.07%)	1255.67 ~ 1475.72 (0.054 ~ 0.070 %)	3244.39 ~ 3629.04 (0.058 ~ 0.074 %)
JPT	2	100%	1435.93 (0.05%)	3272.11 (0.07%)	1295.49 ~ 1577.53 (0.044 ~ 0.066 %)	3059.43 ~ 3540.31 (0.056 ~ 0.078 %)
KHV	2	100%	1602.3 (0.07%)	3776.28 (0.05%)	1469.73 ~ 1743.36 (0.060 ~ 0.081 %)	3489.91 ~ 4212.44 (0.043 ~0.064 %)
CEU	2	72%	1263.46 (0.01%)	3764.64 (0.05%)	1140.8 ~ 1517.84 (0.007 ~ 0.011 %)	3675.19 ~ 3911.2 (0.051 ~ 0.055 %)
FIN	2	100%	755.778 (0.01%)	3385.91 (0.06%)	633.221 ~ 922.091 (0.009 ~ 0.014 %)	3306.55 ~ 3495.91 (0.062 ~ 0.067 %)
GBR	2	96%	1235.53 (0.01%)	3883.29 (0.05%)	1115.21 ~ 1409.92 (0.007 ~ 0.012 %)	3776.31 ~ 4059.2 (0.051 ~ 0.056 %)
IBS	1	66%**	3422.31 (0.06%)		3373.92 ~ 3520.4 (~ 0.060 %)	
TSI	2	56%	1559.7 (0.01%)	3960.33 (0.05%)	1435.98 ~ 1785.45 (0.007 ~ 0.011 %)	3860.6 ~ 4103.9 (0.048 ~ 0.052 %)
BEB	2	100%	1686.26 (0.04%)	3398.11 (0.08%)	1437.09 ~ 1885.12 (0.029 ~ 0.058 %)	3188.55 ~ 3623.38 (0.068 ~ 0.097 %)
GIH	2	100%	1734.39 (0.04%)	3581.28 (0.07%)	409.34 ~ 1929.04 (0.026 ~ 0.058 %)	3275.08 ~ 3938.52 (0.061 ~ 0.093 %)
ITU	2	100%	1891.09 (0.05%)	3693.21 (0.07%)	1762.4 ~ 2055.23 (0.043 ~ 0.062 %)	3504.49 ~ 3938.12 (0.061 ~ 0.080 %)
PJL	2	100%	1591.68 (0.03%)	3483.74 (0.08%)	1436.51 ~ 1769.97 (0.025 ~ 0.042 %)	3367.4 ~ 3649.76 (0.069 ~ 0.086 %)
STU	2	100%	1701.5 (0.04%)	3433.43 (0.08%)	1563.67 ~ 1890.37 (0.029 ~ 0.048 %)	3290.61 ~ 3616.68 (0.075 ~ 0.094 %)

Populations	Number of Introgression	Support Ratio	Time* (Proportion)	Time (Proportion) 95% CI
Papuan	1	99%	2108.59 (0.73%)	2065.05 ~ 2134.94 (~ 0.73 %)
Ust'-Ishim	1	100%	3631.08 (0.041%)	3077.85 ~ 4920.08 (~ 0.041 %) ***

* Time is measured in generation.
** 34% bootstrapping support 2 introgression events.
*** The introgression time has added 1500 generations, since Ust died 45,000 years ago.

Supplementary Table 5.5 Neanderthal-like introgression models

Populations	Number of Introgression	Support Ratio	Time* (Proportion)		Time (Proportion) 95% CI	
CDX	2	100%	1138.58 (0.76%)	1636.57 (0.62%)	1087.2 ~ 1202.77 (0.56 ~ 1.04 %)	1544.12 ~ 1881.43 (0.34 ~ 0.82 %)
CHB	2	100%	1157.56 (1.04%)	1897.45 (0.37%)	1127.14 ~ 1205.67 (0.94 ~ 1.16 %)	1788.17 ~ 2062.29 (0.24 ~ 0.46 %)
CHS	2	100%	1148.42 (0.90%)	1728.90 (0.49%)	1099.7 ~ 1195.1 (0.74 ~ 1.08 %)	1621.44 ~ 1909.34 (0.32 ~ 0.66 %)
JPT	2	100%	1100.47 (0.85%)	1731.47 (0.54%)	1040.69 ~ 1143.59 (0.68 ~ 0.99 %)	1635.42 ~ 1852.34 (0.40 ~ 0.71 %)
KHV	2	100%	1130.53 (0.87%)	1783.80 (0.50%)	1052 ~ 1169.73 (0.65 ~ 1.00 %)	1635.79 ~ 1939.56 (0.37 ~ 0.72 %)
CEU	2	100%	1103.46 (0.66%)	1823.32 (0.45%)	1046.98 ~ 1147.87 (0.53 ~ 0.74 %)	1711.18 ~ 1933.63 (0.36 ~ 0.57 %)
FIN	2	100%	1171.98 (0.80%)	1934.06 (0.33%)	1117.72 ~ 1215.33 (0.70 ~ 0.90 %)	1803.26 ~ 2107.13 (0.24 ~ 0.44 %)
GBR	2	100%	1157.98 (0.74%)	1918.44 (0.36%)	1100.72 ~ 1200.98 (0.63 ~ 0.83 %)	1798.22 ~ 2025.33 (0.28 ~0.48 %)
IBS	1	100%	1174.45 (0.75%)	1960.68 (0.31%)	1123.08 ~ 1216.5 (0.66 ~ 0.83 %)	1842.3 ~ 2103.1 (0.24 ~ 0.41 %)

Populations	Number of Introgression	Support Ratio	Time* (Proportion)		Time (Proportion) 95% CI	
TSI	2	100%	1148.55 (0.71%)	1952.02 (0.35%)	1090.84 ~ 1193.57 (0.57 ~ 0.79 %)	1800.35 ~ 2107.85 (0.27 ~ 0.49 %)
BEB	2	100%	1249.11 (0.76%)	1934.83 (0.45%)	1167.22 ~ 1306.09 (0.51 ~ 0.90 %)	1744.13 ~ 2134.3 (0.30 ~ 0.69 %)
GIH	2	100%	1104.4 (0.48%)	1773.21 (0.67%)	1037.99 ~ 1168.68 (0.38 ~ 0.61 %)	1716.52 ~ 1861.91 (0.54 ~ 0.77 %)
ITU	2	100%	1200.69 (0.58%)	1868.89 (0.57%)	1125.16 ~ 1265.48 (0.43 ~ 0.76 %)	1778.91 ~ 2020.14 (0.39 ~ 0.72 %)
PJL	2	100%	1206.66 (0.66%)	1913.62 (0.48%)	1129.75 ~ 1266.09 (0.50 ~ 0.81 %)	1787.64 ~ 2098.22 (0.33 ~ 0.64 %)
STU	2	100%	1213.12 (0.67%)	1938.60 (0.49%)	1155.29 ~ 1273.72 (0.55 ~ 0.82 %)	1843.86 ~ 2114.56 (0.34 ~ 0.61 %)
Papuan	2	100%	1062.62 (0.69%)	1866.55 (0.86%)	966.415 ~ 1174.54 (0.48 ~ 0.93 %)	1766.69 ~ 2057.88 (0.61 ~ 1.07 %)
Ust'-Ishim	2	94%	1991.71(1.54%)	5090.26(0.068%)	1926.45~2046.47 ** (1.41 ~ 1.57 %)	3185.28~6802.41 (0.042 ~ 0.20 %)

* Time is measured in generation.
** The introgression time has added 1500 generations, since Ust died 45,000 years ago.

5.3 Archaic Ancestry Sharing

Archaic ancestry sharing (AAS), a statistic to measure the ratio of introgression sharing over random, enables us to quantify the similarity of introgression history of different populations. We used this statistic to explore the introgression events sharing and reconstruct the introgression history of Non-African populations. Here, we explore properties and influencing factors of the statistic mathematically and attempt to interpret the ancestry sharing patterns based on the properties and factors.

5.3.1 Archaic Ancestry Sharing Definition

To analyze the relationship of the introgression history, we also proposed an archaic ancestry sharing statistics (see Methods). We assume introgression events happened only few times in the history and the introgression proportion of each event is very small. The introgressed segments derived from the same archaic lineages at the same genomic position are high likely inherited from a common archaic ancestor.

We defined the archaic ancestry sharing (AAS) between population i and population j,

$$S_{ij} = \frac{\sum_{k=1}^{n}(p_{ik} * p_{jk} * L_k)}{P_i * P_j * L}, \tag{24}$$

where P_i stands for the genome wide introgression proportion of population i. p_{ik} is the local introgression proportion of a genomic segment k in population i. We assume there are n segments in the genome and the local introgression proportion of the two populations are identical at any position in one segment. Each segment length is L_k. L is the total length of the genome, $L = \sum_{k=1}^{n} L_k$.

Intuitively, the statistics S_{ij} measures the ratio of introgression sharing over the random introgression sharing of any two populations. Since the introgression proportion of different continental populations are different, two populations with higher introgression proportion trends to share more introgressed segments. To eliminate and control the effect of the population introgression proportion, we divide the introgression sharing by the random sharing.

We applied this method to Eurasian populations from the 1000 Genomes Project and the Papuan from SGDP (Figure 4a, 4b, Supplementary Table 5.6 ~ 5.7). The ancestry sharing between populations from different continents or regions are relatively lower and populations from same region tend to higher. That indicates there might be

both shared introgression events and independent events for populations from different regions.

Supplementary Table 5.6 **Denisovan-like introgression ancestry sharing**

	Papuan	CHB	CHS	JPT	CDX	KHV	CEU	GBR	IBS	TSI	FIN	GIH	PJL	BEB	ITU	STU
Papuan	31.55	4.68	4.61	4.81	5.27	5.06	6.33	6.05	6.40	6.43	5.66	5.42	5.73	5.10	5.77	5.44
CHB	4.68	75.72	72.19	69.96	67.36	66.82	35.21	35.20	34.42	35.20	37.82	25.68	26.24	27.46	24.61	23.28
CHS	4.61	72.19	80.65	71.26	72.23	70.84	34.15	34.10	33.31	33.42	36.89	25.42	25.62	28.20	24.22	23.56
JPT	4.81	69.96	71.26	85.48	67.73	66.39	35.79	35.42	35.28	36.22	38.68	26.86	26.34	28.67	25.10	24.22
CDX	5.27	67.36	72.23	67.73	84.06	72.80	35.08	35.07	34.20	34.58	36.10	27.06	26.93	30.06	25.88	25.47
KHV	5.06	66.82	70.84	66.39	72.80	76.95	36.67	36.88	36.02	36.12	37.22	26.65	27.11	29.43	25.45	25.44
CEU	6.33	35.21	34.15	35.79	35.08	36.67	168.84	154.86	154.35	157.08	128.94	55.09	62.63	47.51	49.66	47.27
GBR	6.05	35.20	34.10	35.42	35.07	36.88	154.86	167.25	149.00	148.60	125.76	55.65	60.36	47.65	47.62	47.44
IBS	6.40	34.42	33.31	35.28	34.20	36.02	154.35	149.00	167.07	158.79	122.34	54.81	61.83	47.19	49.56	47.27
TSI	6.43	35.20	33.42	36.22	34.58	36.12	157.08	148.60	158.79	180.16	122.57	56.47	65.40	48.13	52.59	49.03
FIN	5.66	37.82	36.89	38.68	36.10	37.22	128.94	125.76	122.34	122.57	134.02	47.13	53.20	42.27	42.39	40.97
GIH	5.42	25.68	25.42	26.86	27.06	26.65	55.09	55.65	54.81	56.47	47.13	61.10	49.63	46.12	48.43	46.57
PJL	5.73	26.24	25.62	26.34	26.93	27.11	62.63	60.36	61.83	65.40	53.20	49.63	60.26	45.25	49.00	46.12
BEB	5.10	27.46	28.20	28.67	30.06	29.43	47.51	47.65	47.19	48.13	42.27	46.12	45.25	50.80	44.76	44.58
ITU	5.77	24.61	24.22	25.10	25.88	25.45	49.66	47.62	49.56	52.59	42.39	48.43	49.00	44.76	55.10	46.82
STU	5.44	23.28	23.56	24.22	25.47	25.44	47.27	47.44	47.27	49.03	40.97	46.57	46.12	44.58	46.82	52.99

Supplementary Table 5.7 **Neanderthal-like introgression ancestry sharing**

	Papuan	CHB	CHS	JPT	CDX	KHV	CEU	GBR	IBS	TSI	FIN	GIH	PJL	BEB	ITU	STU
Papuan	18.71	4.71	4.62	4.64	4.68	4.71	3.03	3.01	2.98	2.98	3.24	3.98	3.85	4.20	4.05	4.14
CHB	4.71	11.46	11.07	10.67	10.69	10.62	3.91	3.93	3.82	3.81	4.51	4.94	4.94	5.72	5.15	5.14
CHS	4.62	11.07	11.52	10.60	10.99	10.85	3.85	3.90	3.78	3.76	4.43	4.88	4.89	5.69	5.11	5.11
JPT	4.64	10.67	10.60	11.48	10.23	10.14	3.80	3.81	3.74	3.71	4.40	4.86	4.87	5.62	5.10	5.09
CDX	4.68	10.69	10.99	10.23	11.80	11.11	3.83	3.88	3.77	3.75	4.38	4.87	4.90	5.73	5.12	5.13
KHV	4.71	10.62	10.85	10.14	11.11	11.34	3.86	3.92	3.79	3.78	4.41	4.93	4.93	5.74	5.15	5.15
CEU	3.03	3.91	3.85	3.80	3.83	3.86	10.68	10.21	10.06	9.92	9.64	6.01	6.33	5.39	5.50	5.37
GBR	3.01	3.93	3.90	3.81	3.88	3.92	10.21	10.79	10.05	9.92	9.61	6.04	6.34	5.41	5.51	5.40
IBS	2.98	3.82	3.78	3.74	3.77	3.79	10.06	10.05	10.77	10.14	9.31	5.90	6.21	5.28	5.41	5.29
TSI	2.98	3.81	3.76	3.71	3.75	3.78	9.92	9.92	10.14	10.71	9.16	6.03	6.31	5.36	5.54	5.41
FIN	3.24	4.51	4.43	4.40	4.38	4.41	9.64	9.61	9.31	9.16	10.58	5.97	6.23	5.46	5.50	5.38
GIH	3.98	4.94	4.88	4.86	4.87	4.93	6.01	6.04	5.90	6.03	5.97	7.91	6.99	6.76	7.05	6.96
PJL	3.85	4.94	4.89	4.87	4.90	4.93	6.33	6.34	6.21	6.31	6.23	6.99	7.61	6.64	6.94	6.82
BEB	4.20	5.72	5.69	5.62	5.73	5.74	5.39	5.41	5.28	5.36	5.46	6.76	6.64	7.25	6.82	6.76
ITU	4.05	5.15	5.11	5.10	5.12	5.15	5.50	5.51	5.41	5.54	5.50	7.05	6.94	6.82	7.73	7.09
STU	4.14	5.14	5.11	5.09	5.13	5.15	5.37	5.40	5.29	5.41	5.38	6.96	6.82	6.76	7.09	7.57

5.3.2 Archaic Ancestry Sharing Properties

For a genome with length L (bp), we divide it into n segments with length L_k $(k = 1, 2, \dots, n)$ on which all positions(bp) take an identical frequency of archaic state in each population while frequencies can be different between two populations. It is obvious that the above division certainly exists since a strategy to divide genome with $L_k = 1bp$ $(k = 1, 2, \dots, n)$, satisfies the requiring consistency of frequencies.

For arbitrary two populations, we denote global archaic ancestry proportions of the two populations by P_1, P_2 respectively, and denote the archaic ancestry frequency of segment k by p_{1k}, p_{2k}, $(k = 1, 2, \dots, n)$.

The AAS of these two populations is defined on a division of a genome as

$$S_{12} = \frac{\sum_{k=1}^{n} p_{1k} p_{2k} L_k}{P_1 P_2 L}. \tag{25}$$

This statistic has following properties.

Property 1. The value of the AAS is not affected by the genomic division.

By definition, if a segment satisfying the consistency of archaic ancestry frequency in each population can be divided into some even shorter segments, and these shorter segments still satisfy the consistency.

Considering a segment with length $l\,(l > 2bp)$ in a division, we apply a further splitting step that divide this segment into n' segments with length l_k $(k = 1, 2, \dots, n')$ and obtain a new division. Note that these n' segments share identical archaic ancestry frequencies in two populations, which denoted by p_1, p_2 respectively. We have

$$p_1 p_2 l = \sum_{k=1}^{n} p_1 p_2 l_k, \tag{26}$$

which means the value of the terms related to this segment in the numerator of the new division is equal to the corresponding value of original version. Since other parts keeps unchanged, the value of the whole statistic across the genome are unchanged.

Note that splitting steps increase the number of segments. An arbitrary division can be transformed into the finest division in which each segment takes minimum length, which equal to $1bp$. Moreover, the value of the statistic keeps unchanged during the splitting process, which indicates that the values of all possible division are equal to

that of the finest one. To be convenient for further discussion, we use the finest division to calculate the statistics where $n = L$ and $L_k = 1$.

Property 2. Given two populations with archaic introgression proportion P_1, P_2, respectively and two sets of frequencies, $p_{i1}, p_{i2}, \dots, p_{iL}$ $(i = 1, 2)$ across a genome, let $p_{1j_1} \geq p_{1j_2} \geq \dots \geq p_{1j_L}, p_{1k_1} \geq p_{1k_2} \geq \dots \geq p_{1k_L}$ in which $j_1, j_2, \dots, j_L$ and $k_1, k_2, \dots, k_L$ are two permutations of $1,2,\dots,L$, then the AAS value achieve the maximum when $j_m = k_m$ $(m = 1,2,\dots,L)$ and achieve the minimum when $j_m = k_{L+1-m}$ $(m = 1,2,\dots,L)$.

According to the sequence inequality, we have

$$\sum_{m=1}^{L} p_{1j_m} p_{2k_m} \geq \sum_{m=1}^{L} p_{1j'_m} p_{2k'_m} \geq \sum_{m=1}^{L} p_{1j_m} p_{2k_{L+1-m}}, \tag{27}$$

where $j'_1, j'_2, \dots, j'_L$ and $k'_1, k'_2, \dots, k'_L$ are two arbitrary permutations of $1, 2, \dots, L$.

Since P_1 and P_2 are fixed, we find

$$\frac{\sum_{m=1}^{L} p_{1j_m} p_{2k_m}}{P_1 P_2 L} \geq \frac{\sum_{m=1}^{L} p_{1j'_m} p_{2k'_m}}{P_1 P_2 L} \geq \frac{\sum_{m=1}^{L} p_{1j_m} p_{2k_{L+1-m}}}{P_1 P_2 L}. \tag{28}$$

This proportion reflects that a consistency of frequencies' ranks between two populations generates the greatest value of the AAS on condition that two sets of frequencies are fixed, which suggests that two populations sharing corresponding high-frequency regions and low-frequencies regions could make a greater value of the AAS. Conversely, reversed orders of frequencies between two populations generate the smallest value of the AAS.

Property 3. Given two populations with archaic introgression proportion P_1, P_2, respectively and one set of frequencies, $p_{11}, p_{12}, \dots, p_{1L}$. Let $p_{11} \geq p_{12} \geq \dots \geq p_{1L}$ without loss of generality and

$$P_2 L = t_2 + s_2, \tag{29}$$

where t_2 is non-negative integers and $0 \leq s_2 < 1$. The AAS value achieve the maximum if

$$p_{2m} = \begin{cases} 1 & m = 1,2,\dots,t_2 \\ s_2 & m = t_2 + 1 \\ 0 & m = t_2 + 2, t_2 + 3, \dots, L \end{cases}. \tag{30}$$

We have

$$\max[S_{12}] = \begin{cases} \dfrac{1}{P_1} & t_2 < c_1 \\ \dfrac{1}{P_2} - \dfrac{\sum_{m=t_2+1}^{c_1+c_2} p_{1m} + p_{1t_2+1}(1-s_2)}{P_1 P_2 L} & c_1 \le t_2 < c_1 + c_2 \, , \\ \dfrac{1}{P_2} & t_2 \ge c_1 + c_2 \end{cases} \quad (31)$$

where

$$\begin{cases} c_1 = |\{m | p_{2m} = 1, m = 1,2,\dots,L\}| \\ c_2 = |\{m | 0 < p_{2m} < 1, m = 1,2,\dots,L\}| \, , \\ c_3 = |\{m | p_{2m} = 0, m = 1,2,\dots,L\}| \end{cases} \quad (32)$$

and t_2, s_2 satisfies that $P_2 L = t_2 + s_2$, t_2 is an integer and $0 \le s_2 < 1$.

Note that this is a linear programming problem and the standard form is

$$\begin{cases} \max z = \sum_{m=1}^{L} p_{1m} p_{2m} \\ s.t. \sum_{m=1}^{L} p_{2m} = P_2 L \\ p_{2m} + q_m = 1 & m = 1,2,\dots,L \\ p_{2m} \ge 0, q_m \ge 0 & m = 1,2,\dots,L \end{cases} \, , \quad (33)$$

where q_m are slack variables. We can easily find a feasible solution of the problem such as $p_{2m} = P_2, m = 1, 2, \dots, L$, hence, there is at least one basic feasible solution. Moreover, exist a basic feasible solution which is an optimal solution since the optimal value of the problem is finite. In this problem, the constrained condition is

$$\begin{pmatrix} 1 & 1 & \dots & 1 & 0 & 0 & \dots & 0 \\ 1 & 0 & \dots & 0 & 1 & 0 & \dots & 0 \\ 0 & 1 & \dots & 0 & 0 & 1 & \dots & 0 \\ . & . & & . & . & . & & . \\ . & . & & . & . & . & & . \\ . & . & & . & . & . & & . \\ 0 & 0 & \dots & 1 & 0 & 0 & \dots & 1 \end{pmatrix} \begin{pmatrix} p_{21} \\ p_{22} \\ . \\ . \\ . \\ p_{2L} \\ q_1 \\ q_2 \\ . \\ . \\ . \\ q_L \end{pmatrix} = \begin{pmatrix} P_2 L \\ 1 \\ 1 \\ . \\ . \\ . \\ 1 \end{pmatrix}, \quad (34)$$

in which the constrained matrix is a $(L+1) \times 2L$ matrix with rank $L+1$. For a vector $\mathbf{x} = (x_1, x_2, \dots, x_n)$, denote

$$f(\mathbf{x}, k) = |\{x_i | x_i = k, i = 1,2,\dots,n\}|. \quad (35)$$

Let $\mathbf{p}^* = (p_{21}^*, p_{22}^*, \ldots, p_{2L}^*, q_1^*, q_2^*, \ldots, q_L^*)$ be the optimal basic feasible solution so that $p_{21}^* \geq p_{22}^*, \geq \ldots, \geq p_{2L}^*$, otherwise a permutation of $p_{21}^*, p_{22}^*, \ldots, p_{2L}^*$ with descending order can make a greater value than the optimal value according to Property 2. On the one hand,

$$f(\mathbf{p}^*, 0) \geq L - 1, \quad (36)$$

since $\mathbf{p}^*$ is basic feasible solution. On the other hand,

$$\begin{cases} f(\mathbf{p}^*, 0) = f((p_{21}^*, p_{22}^*, \ldots, p_{2L}^*), 0) + f((p_{21}^*, p_{22}^*, \ldots, p_{2L}^*), 1) \\ f((p_{2m}^*, q_m^*), 0) \leq= 1 \qquad \mathrm{m} = 1, 2, \ldots, L \end{cases} . \quad (37)$$

since $p_{2m}^* + q_m^* = 1$, for each pair p_{2m}, q_m. Note that if exist $p_{2m_1}^*$ in $\mathbf{p}^*$ satisfying $0 < p_{2m_1}^* < 1, p_{2m_1}^* \neq s_2$, there must be m_2 satisfying $0 < p'_{2m_2} < 1$, otherwise $\sum_{m=1}^{L} p_{2m}^* \neq t_2 + s_2$. On this scenario,

$$f(\mathbf{p}^*, 0) \leq L - 2. \quad (38)$$

It is a contradiction. Hence, there is only one type of the optimal basic feasible solution

$$p_{2m}^* = \begin{cases} 1 & m = 1, 2, \ldots, t_2 \\ s_2 & m = t_2 + 1 \\ 0 & m = t_2 + 2, t_2 + 3, \ldots, L \end{cases} , \quad (39)$$

so we have

$$\max[S_{12}] = \begin{cases} \frac{1}{P_1} & t_2 < c_1 \\ \frac{1}{P_2} - \frac{\sum_{m=t_2+1}^{c_1+c_2} p_{1m} + p_{1t_2+1}(1 - s_2)}{P_1 P_2 L} & c_1 \leq t_2 < c_1 + c_2 . \\ \frac{1}{P_2} & t_2 \geq c_1 + c_2 \end{cases} \quad (40)$$

Actually, there are a massive of feasible solutions, not basic solutions, which are also optimal solutions. These solutions could be generated by adjusting values of variables in $\{p_{2m} | m \in A\}$ where $A = \{m | p_{1m} = k\}$ while keeping the summation of it unchanged for all possible k. This property demonstrates that the extreme enrichment of the archaic introgression of population 2 on regions with top frequencies in population 1 makes the AAS achieve the maximum on condition that global proportions of the archaic introgression in two populations and frequencies in population 1 are given.

Property 4. Given two populations with archaic introgression proportion P_1, P_2, satisfying

$$P_1 L = t_1 + s_1, \tag{41}$$

$$P_2 L = t_2 + s_2, \tag{42}$$

where t_1, t_2 are non-negative integers and $0 \le s_1 < 1$, $0 \le s_2 < 1$, we have

$$\max[S_{12}] = \begin{cases} \dfrac{1}{\max[P_1, P_2]} & t_1 \neq t_2 \\ \dfrac{1}{\max[P_1, P_2]} - \dfrac{s_{argmin_i[P_i]} - s_1 s_2}{P_1 P_2 L} & t_1 = t_2 \end{cases}. \tag{43}$$

Note that this is a non-linear programming problem

$$\begin{cases} \max z = \sum_{m=1}^{L} p_{1m} p_{2m} & \\ s.t. \sum_{m=1}^{L} p_{im} - P_i L = 0 & i = 1,2 \\ p_{im} - 1 \le 0, -p_{im} \le 0 & m = 1,2,\dots,L \end{cases}, \tag{44}$$

where P_1, P_2, L are fixed. The problem satisfies the linearity constraint qualification (LCQ) and Karush–Kuhn–Tucker (KKT) conditions can be used to explore the maximum. Denote

$$\begin{cases} \mathbf{x} = (p_{11}, p_{12}, \dots, p_{1L}, p_{21}, p_{22}, \dots, p_{2L}) \\ f(\mathbf{x}) = \sum_{m=1}^{L} p_{1m} p_{2m} \\ s_i(\mathbf{x}) = \sum_{m=1}^{L} p_{im} - P_i L & i = 1,2 \\ g_{im}(\mathbf{x}) = p_{im} - 1, h_{im}(\mathbf{x}) = -p_{im} & i = 1,2, \quad m = 1,2,\dots,L \end{cases}. \tag{45}$$

According to KKT conditions, the Lagrangian function is

$$L(\mathbf{x}, \boldsymbol{\mu}, \boldsymbol{\lambda}) = f(\mathbf{x}) - \boldsymbol{\mu}\mathbf{g}(\mathbf{x})^T - \boldsymbol{\lambda}\mathbf{h}(\mathbf{x})^T - \boldsymbol{\gamma}\mathbf{s}(\mathbf{x})^T \tag{46}$$

where

$$\begin{cases} \boldsymbol{s}_i(\mathbf{x}) = (s_1(\mathbf{x}), s_2(\mathbf{x})) \\ \mathbf{g} = (g_{11}(\mathbf{x}), \dots, g_{iL}(\mathbf{x}), g_{21}(\mathbf{x}), \dots, g_{2L}(\mathbf{x})) \\ \mathbf{h} = (h_{11}(\mathbf{x}), \dots, h_{iL}(\mathbf{x}), h_{21}(\mathbf{x}), \dots, h_{2L}(\mathbf{x})) \\ \qquad \boldsymbol{\mu} = (\mu_{11}, \dots, \mu_{1L}, \mu_{21}, \dots, \mu_{2L}) \\ \qquad \boldsymbol{\lambda} = (\lambda_{11}, \dots, \lambda_{1L}, \lambda_{21}, \dots, \lambda_{2L}) \\ \qquad \boldsymbol{\gamma} = (\gamma_1, \gamma_2) \end{cases}, \tag{47}$$

and the optimal solution $\mathbf{x}^*$ satisfies

$$\begin{cases} \dfrac{\partial L}{\partial \mathbf{x}^*} = \mathbf{0} \\ s_i(\mathbf{x}^*) = 0 & i = 1,2 \\ g_{im}(\mathbf{x}^*) \leq 0, h_{im}(\mathbf{x}^*) \leq 0 \\ \mu_{im} \geq 0, \lambda_{im} \geq 0 & i = 1,2 m = 1,2, \dots, L \\ \mu_{im} g_{im}(\mathbf{x}^*) = 0, \lambda_{im} h_{im}(\mathbf{x}^*) = 0 \end{cases}. \tag{48}$$

So we have

$$\begin{cases} \sum_{m=1}^{L} p_{im}^* - P_i L = 0 & i = 1,2 & (1) \\ p_{2m}^* - \mu_{1m} + \lambda_{1m} - \gamma_1 = 0 & & (2) \\ p_{1m}^* - \mu_{2m} + \lambda_{2m} - \gamma_2 = 0 & & (3) \\ \mu_{im} \geq 0, \lambda_{im} \geq 0 & i = 1,2, m = 1,2, \dots, L & (4) \\ p_{im}^* - 1 \leq 0, -p_{im}^* \leq 0 & & (5) \\ \mu_{im}(p_{im}^* - 1) = 0, -\lambda_{im} p_{im}^* = 0 & & (6) \end{cases}. \tag{49}$$

Considering the condition (2), (3), (5), (6), We obtain some expressions based on 3×3 different possible values of pairs (p_{1m}, p_{2m})

p_{2m}^* \ p_{1m}^*	0	(0,1)	1
0	$\begin{cases} \gamma_1 = \lambda_{1m} \\ \gamma_2 = \lambda_{2m} \end{cases}$	$\begin{cases} \gamma_1 = 0 \\ \gamma_2 = p_{1m}^* + \lambda_{2m} \end{cases}$	$\begin{cases} \gamma_1 = -\mu_{1m} \\ \gamma_2 = \lambda_{2m} + 1 \end{cases}$
(0,1)	$\begin{cases} \gamma_1 = p_{2m}^* + \lambda_{1m} \\ \gamma_2 = 0 \end{cases}$	$\begin{cases} \gamma_1 = 0 \\ \gamma_2 = 0 \end{cases}$	$\begin{cases} \gamma_1 = p_{2m}^* - \mu_{1m} \\ \gamma_2 = 1 \end{cases}$
1	$\begin{cases} \gamma_1 = \lambda_{1m} + 1 \\ \gamma_2 = -\mu_{2m} \end{cases}$	$\begin{cases} \gamma_1 = 1 \\ \gamma_2 = p_{1m}^* - \mu_{2m} \end{cases}$	$\begin{cases} \gamma_1 = 1 - \mu_{1m} \\ \gamma_2 = 1 - \mu_{2m} \end{cases}$

All combinations of these 9 expressions which don't result in contradictions in solutions while the solutions satisfy the condition (1), (4) can achieve the maximum of the sharing statistic. Hence, there are a massive of optimal solutions, which causes difficulties to describe all optimal solution. Here, we show an extreme scenario,

$$p^*_{im} = \begin{cases} 1 & m = 1,2,\dots,t_i \\ s_i & m = t_i + 1 \\ 0 & m = t_i + 2, t_i + 3, .., L \end{cases} , \tag{50}$$

where $i = 1,2$. It is not complex to verify that this scenario satisfies the KKT condition. And we have

$$\max[S_{12}] = \begin{cases} \frac{1}{\max[P_1, P_2]} & t_1 \neq t_2 \\ \frac{1}{\max[P_1, P_2]} - \frac{s_{\mathrm{argmin}_i[P_i]} - s_1 s_2}{P_1 P_2 L} & t_1 = t_2 \end{cases} . \tag{51}$$

Considering that the length of whole genome approximates 3×10^9 and the general proportion of archaic ancestry is in the level of 10^{-2} or 10^{-3}, we have $\frac{s_{\mathrm{argmin}_i[P_i]} - s_1 s_2}{P_1 P_2 L} \ll \frac{1}{\max[P_1, P_2]}$ which means $\frac{1}{\max[P_1, P_2]}$ is generally decide the maximum, therefore, the less the greater global archaic proportion of two is, the larger AAS value is possibly achieved. The proportions of Denisovan-like introgression are generally lower than it of Neanderthal-like introgression, which results in that the AAS value for Denisovan-like introgression is lower than it for Neanderthal-like introgression.

Property 5. Given two populations with archaic introgression proportion P_1, P_2, satisfying

$$P_1 L = t_1 + s_1, \tag{52}$$

$$P_2 L = t_2 + s_2, \tag{53}$$

$$t_1 + t_2 \ll L, \tag{54}$$

where t_1, t_2 are non-negative integers and $0 \leq s_1 < 1$, $0 \leq s_2 < 1$, we have

$$\min(S_{12}) = 0, \tag{55}$$

when and only when

$$\exists \mathbf{M} \subseteq \{1, 2, \dots, L\}, \forall m \in \mathbf{M}, p_{1m} = 0, \forall m' \in \overline{\mathbf{M}}, p_{2m} = 0. \tag{56}$$

Since the low proportion of the archaic introgression in modern human populations, we add a condition, $t_1 + t_2 \ll L$, to Property 3 and explore the minimum. Here, we show a simple deduction instead of the KKT condition for non-linear programming problems.

By the definition of the sharing statistic, since each term in the formula is non-negative, we have

$$S_{12} = \frac{\sum_{k=1}^{n} p_{1k} p_{2k} L_k}{P_1 P_2 L} \geq 0. \tag{57}$$

On the one hand, if $\exists \mathbf{M} \subseteq \{1, 2, \ldots, L\}, \forall m \in \mathbf{M}, p_{1m} = 0, \forall m' \in \overline{\mathbf{M}}, p_{2m} = 0$ which is feasible due to $t_1 + t_2 \ll L$, the AAS value achieves 0. On the other hand, if do not exist a set satisfying the above condition, which means exist one position m_0 taking frequencies $p_{1m_0} > 0$, $p_{2m_0} > 0$, the AAS value is greater than 0. Hence, the proportion is proved.

Based on the deduction above, it can be seen that two aspects of factors impact the AAS value. One is the similarity of two archaic introgression distributions of two populations across the genome, which indicates how much extent that two populations share corresponding high-frequency regions and low-frequency regions. Pearson Correlation Coefficient (PCC) can mostly interpret this factor (Supplementary Figure 5.12 A-B). High PCC values are taken between populations from one region which also take high AAS values, indicating an approximate distribution of the archaic introgression on genomes. Moreover, European populations and South Asian populations take relatively higher PCC values than other pairs of populations from different regions, which suggests common archaic introgression events between populations from two regions.

The other one is the dispersion degree of frequencies relative to the global proportion of one population since frequencies used in the calculation of the AAS is indeed a relative value $\frac{p_{im}}{P_i}$ instead of p_{im}, which indicates that a position taking frequency 1 in a population with a global proportion 0.005 make an increasing impact to the AAS value from it in another population with global proportion 0.05. Coefficient of variation, a measure to describe the degree of dispersion of the frequencies relative to the mean, can interpret this factor (Supplementary Figure 5.12 C-D). For pairs of populations from an identical region taking high PCC values, the AAS mainly reflects dispersion degrees in the region. European populations show a greater coefficient of variations of Denisovan-like introgression whereas South Asian populations show more uniform (Supplementary Figure 5.12 D), which corresponds to that European populations take higher AAS values of Denisovan-like introgression than South Asian

populations. Note that the global proportion of Denisovan-like introgression in the European population (~0.0007) is lower than it in South Asian populations (~0.001), which suggests that the Denisovan-like introgression in European populations enriches on some regions and carry a lower diversity than it in South Asian populations. Considering the high PCC values between European and South Asian populations, we conclude that Denisovan-like introgression in European populations derives from a potential gene flow from South Asian populations.

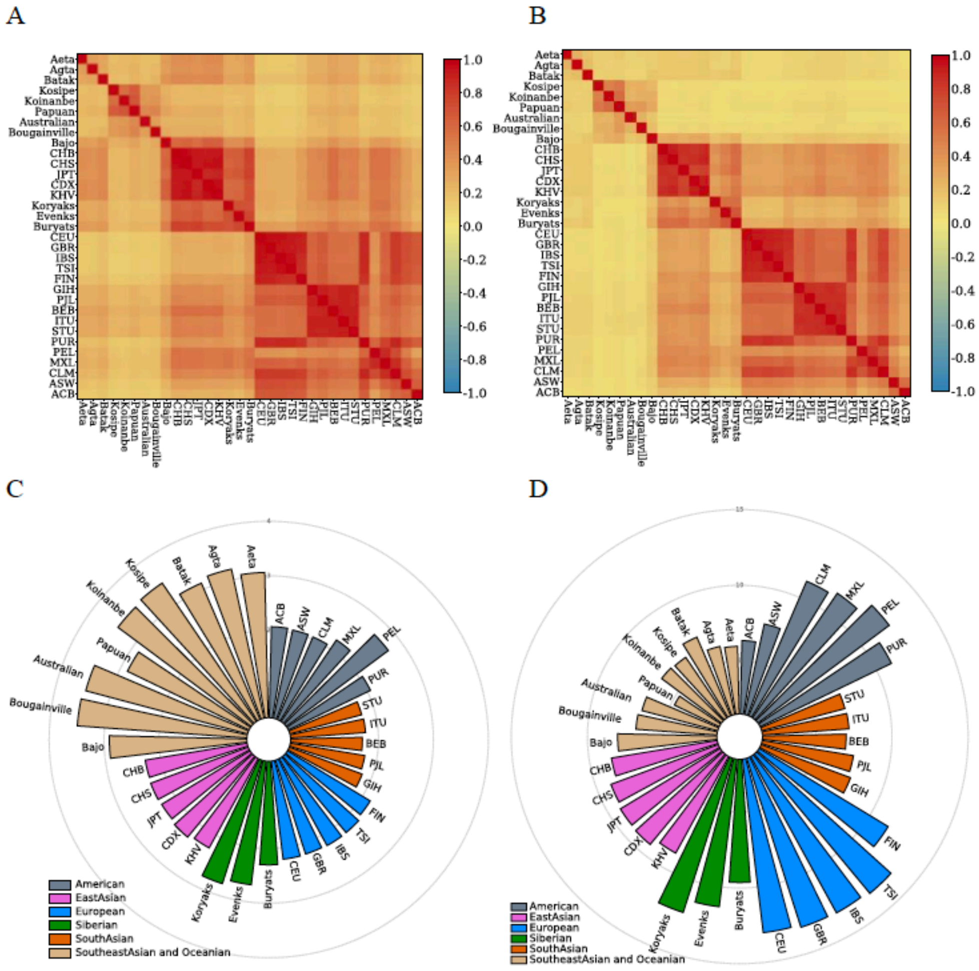

Supplementary Figure 5.13 Two aspects of factors impacting the AAS value. (A) Pearson Correlation Coefficient (PCC) of frequency of Neanderthal-like introgression. (B) Pearson Correlation Coefficient (PCC) of frequency of Denisovan-like introgression. (C) The dispersion degree of frequencies of Denisovan-like introgression relative to the global proportion of one population. (D) The dispersion degree of frequencies of Neanderthal-like introgression relative to the global proportion of one population.

5.4 Introgression Desert

We used Yoruba in Ibadan, Nigeria (YRI, n = 108) from the 1000 Genomes Project (KGP) as the African reference and Altai Neanderthal and Altai Denisovan as the archaic references. For the non-Africans, data of East Asian (including Chinese Dai from Xishuangbanna, China [CDX], n = 93; Han Chinese from Beijing, China [CHB], n = 103; Han Chinese from South China [CHS], n = 105; Japanese from Tokyo, Japan [JPT], n = 104; and Kinh from Ho Chi Minh City, Vietnam [KHV], n = 99), European (including British from England and Scotland [GBR], n = 91; Utah residents with Northern and Western European ancestry [CEU] from the CEPH collection, n = 99; Iberian populations in Spain [IBS], n = 107; and Tuscans in Italy [TSI], n = 107), and South Asian (including Gujarati Indians from Houston, Texas, United States [GIH], n = 103; Indian Telugu from the UK [ITU], n = 102; Punjabi from Lahore, Pakistan [PJL], n = 96; and Sri Lankan Tamil from the UK [STU], n = 102)* populations were obtained from the KGP, and data of the Papuan population (Papua New Guineans, n = 15) data were obtained from the Simons Genome Diversity Project. Here, we did not include Bengali from Bangladesh (BEB) of the KGP dataset in this analysis, since there might have been gene flow from East Asia to BEB. We detected 84 genomic regions, which lack introgression sequences. We firstly divided the genome into thousands of 100kb-bins. Then, we got the empirical distribution of introgression covered length. A two-tail test was performed to identify those genomic regions with extremely rare introgression sequences (Supplementary Table 5.8).

Supplementary Table 5.8 **Introgression "desert"**

Chromosome	Start	End	Desert length (Mb)	Introgressed sequences length (kb)	P^*
1	72,210,505	79,510,505	7.3	59.6	0.004
1	103,910,505	112,610,505	8.7	151.8	0.004
1	153,310,505	158,010,505	4.7	21.1	0.010
1	173,510,505	175,110,505	1.6	0.0	0.047
2	21,310,200	28,310,200	7	85.5	0.007
2	41,110,200	46,210,200	5.1	53.1	0.014
2	72,010,200	75,210,200	3.2	10.1	0.017
2	61,110,200	63,910,200	2.8	12.9	0.028
2	130,010,200	134,110,200	4.1	33.7	0.019
2	201,010,200	209,010,200	8	153.6	0.009
2	185,810,200	188,310,200	2.5	5.8	0.027
2	213,610,200	215,410,200	1.8	0.0	0.036
2	228,610,200	232,310,200	3.7	64.8	0.044
2	192,910,200	194,710,200	1.8	3.1	0.047
2	224,210,200	225,810,200	1.6	1.0	0.048
3	46,960,069	50,160,069	3.2	0.0	0.007
3	76,460,069	90,060,069	13.6	657.6	0.010
3	70,260,069	72,060,069	1.8	0.0	0.036
3	95,360,069	100,560,069	5.2	15.5	0.007
3	100,960,069	103,960,069	3	0.0	0.009
3	135,760,069	139,760,069	4	31.8	0.020
4	47,410,202	49,210,202	1.8	0.0	0.036
4	140,410,202	143,510,202	3.1	20.7	0.029
4	158,610,202	160,410,202	1.8	0.0	0.036
4	132,810,202	135,910,202	3.1	36.8	0.040
4	85,510,202	88,210,202	2.7	27.2	0.049
4	103,110,202	104,710,202	1.6	0.0	0.047
4	151,010,202	152,710,202	1.7	0.0	0.041
5	67,910,043	71,210,043	3.3	0.0	0.006
5	75,810,043	78,110,043	2.3	3.1	0.028
5	61,610,043	63,910,043	2.3	9.6	0.039
5	135,810,043	137,810,043	2	0.0	0.028
5	129,610,043	131,310,043	1.7	0.0	0.041
5	139,010,043	141,010,043	2	0.0	0.028
6	12,463,854	14,263,854	1.8	0.0	0.036
6	48,163,854	49,763,854	1.6	0.0	0.047
6	158,263,854	160,363,854	2.1	9.2	0.047
7	71,914,808	74,614,808	2.7	5.5	0.023
7	86,214,808	94,414,808	8.2	323.4	0.035
7	98,714,808	100,314,808	1.6	0.0	0.047
7	105,214,808	125,414,808	20.2	989.2	0.001
7	143,414,808	145,814,808	2.4	0.0	0.018
8	52,811,740	66,011,740	13.2	644.9	0.011

Chromosome	Start	End	Desert length (Mb)	Introgressed sequences length (kb)	P^*
8	46,911,740	48,511,740	1.6	0.0	0.047
8	110,011,740	117,311,740	7.3	130.3	0.010
8	91,111,740	94,111,740	3	0.0	0.009
8	105,711,740	108,711,740	3	0.0	0.009
8	99,511,740	101,511,740	2	1.8	0.033
9	14,010,327	16,610,327	2.6	4.7	0.023
9	7,910,327	9,610,327	1.7	0.0	0.041
9	121,010,327	123,010,327	2	3.4	0.039
10	21,760,494	23,360,494	1.6	0.0	0.047
10	99,260,494	109,560,494	10.3	225.5	0.010
10	58,560,494	61,060,494	2.5	1.5	0.019
11	13,973,015	17,373,015	3.4	5.2	0.012
11	46,273,015	48,073,015	1.8	2.9	0.047
11	63,173,015	68,473,015	5.3	40.8	0.010
11	76,273,015	78,673,015	2.4	0.0	0.018
11	73,773,015	76,073,015	2.3	14.8	0.049
12	72,660,181	74,660,181	2	0.0	0.028
12	64,360,181	66,160,181	1.8	0.0	0.036
12	110,160,181	113,160,181	3	0.0	0.009
13	55,220,047	60,820,047	5.6	33.7	0.007
14	25,600,017	28,300,017	2.7	10.5	0.028
15	72,000,041	74,400,041	2.4	3.9	0.026
15	75,200,041	77,500,041	2.3	0.0	0.020
15	99,000,041	100,800,041	1.8	0.0	0.036
16	29,660,086	33,760,086	4.1	6.2	0.010
16	9,760,086	19,860,086	10.1	485.3	0.029
16	62,260,086	68,360,086	6.1	156.7	0.028
16	46,460,086	48,460,086	2	0.0	0.028
17	15,400,052	19,100,052	3.7	53.6	0.037
17	26,800,052	29,200,052	2.4	4.6	0.028
17	56,000,052	62,300,052	6.3	63.4	0.008
17	38,200,052	49,900,052	11.7	542.9	0.017
17	65,600,052	69,100,052	3.5	43.4	0.036
18	30,310,644	32,110,644	1.8	0.0	0.036
18	38,010,644	40,910,644	2.9	35.9	0.047
18	62,910,644	64,710,644	1.8	0.9	0.036
19	12,460,842	14,460,842	2	1.9	0.036
19	15,060,842	16,860,842	1.8	0.0	0.036
19	21,660,842	24,460,842	2.8	29.7	0.046
20	31,960,343	34,760,343	2.8	0.0	0.012

* A two-tail test was performed to identify those genomic regions with extremely rare introgression sequences

Despite the introgressed sequences wide-spreading across the genome, we

identified a set of archaic deserts showing depleted archaic ancestry, of which 6 extended up to 10 Mb in length (Supplementary Data 5). Interestingly, the archaic deserts are significantly enriched for genes related to skin development and keratinization (Supplementary Figure 5.14; Supplementary Data 7), most of which (59 in 73) belong to the *KRT* (keratin) or *KRTAP* (keratin-associated protein) gene family. The underlying mechanisms of the archaic deserts are not yet fully understood, but there should be some driving forces leading to the repeated loss of archaic ancestry at these regions across multiple independent admixture events.

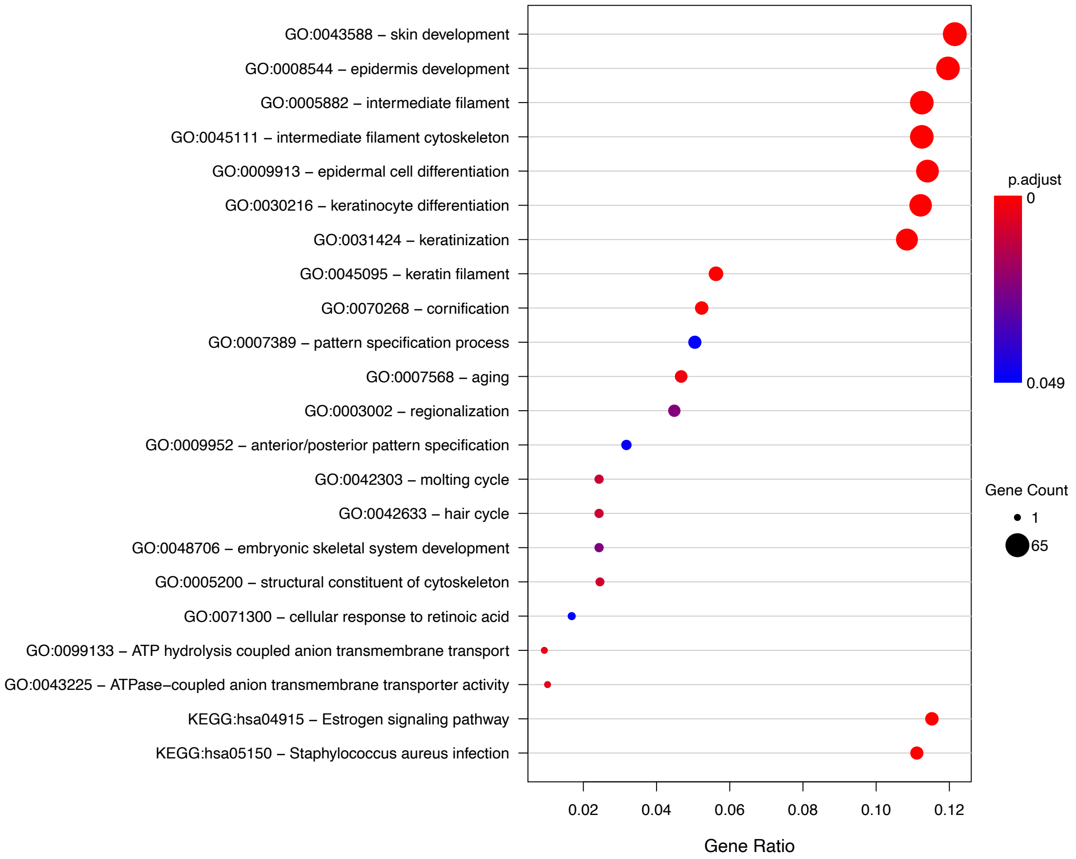

Supplementary Figure 5.14 **Enrichment analysis of introgression "desert".**

6 Reference

1. Fu, Q. *et al.* Genome sequence of a 45,000-year-old modern human from western Siberia. *Nature* **514**, 445, doi:10.1038/nature13810 (2014).
2. Prufer, K. *et al.* The complete genome sequence of a Neanderthal from the Altai Mountains. *Nature* **505**, 43-49, doi:10.1038/nature12886 (2014).
3. Meyer, M. *et al.* A high-coverage genome sequence from an archaic Denisovan individual. *Science* **338**, 222-226, doi:10.1126/science.1224344 (2012).
4. Genomes Project, C. *et al.* An integrated map of genetic variation from 1,092 human genomes. *Nature* **491**, 56-65, doi:10.1038/nature11632 (2012).
5. Mallick, S. *et al.* The Simons Genome Diversity Project: 300 genomes from 142 diverse populations. *Nature* **538**, 201-206 (2016).
6. Pagani, L. *et al.* Genomic analyses inform on migration events during the peopling of Eurasia. *Nature* **538**, 238, doi:10.1038/nature19792 (2016).
7. Prufer, K. *et al.* A high-coverage Neandertal genome from Vindija Cave in Croatia. *Science* **358**, 655-658, doi:10.1126/science.aao1887 (2017).
8. Delaneau, O., Marchini, J. & Zagury, J. F. A linear complexity phasing method for thousands of genomes. *Nat Methods* **9**, 179-181, doi:10.1038/nmeth.1785 (2011).
9. Lu, D. *et al.* Ancestral origins and genetic history of Tibetan highlanders. *Am J Hum Genet* **99**, 580-594, doi:10.1016/j.ajhg.2016.07.002 (2016).
10. Ni, X. *et al.* Corrigendum: Length distribution of ancestral tracks under a general admixture model and its applications in population history inference. *Scientific reports* **6**, 26367, doi:10.1038/srep26367 (2016).
11. Ni, X. *et al.* MultiWaver 2.0: modeling discrete and continuous gene flow to reconstruct complex population admixtures. *Eur J Hum Genet* **27**, 133-139, doi:10.1038/s41431-018-0259-3 (2019).
12. Ni, X. *et al.* Inference of multiple-wave admixtures by length distribution of ancestral tracks. *Heredity* **121**, 52-63, doi:10.1038/s41437-017-0041-2 (2018).
13. Dempster, A. P., Laird, N. M. & Rubin, D. B. Maximum likelihood from incomplete data via the EM algorithm. *Journal of the Royal Statistical Society. Series B (Methodological)* **39**, 1-38 (1977).
14. Chimpanzee, S. & Analysis, C. Initial sequence of the chimpanzee genome and comparison with the human genome. *Nature* **437**, 69-87, doi:10.1038/nature04072 (2005).
15. Svanberg, K. A class of globally convergent optimization methods based on conservative convex separable approximations. *Siam Journal on Optimization* **12**, 555-573 (2009).
16. Johnson, S. G. *The NLopt nonlinear-optimization package*, <http://github.com/stevengj/nlopt>
17. International HapMap, C. *et al.* A second generation human haplotype map of over 3.1 million SNPs. *Nature* **449**, 851-861, doi:10.1038/nature06258 (2007).
18. Viterbi, A. J. Error bounds for convolutional codes and an asymptotically optimum decoding algorithm. *IEEE Trans.informat.theory* **13**, 260-269 (1967).
19. Browning, S. R., Browning, B. L., Zhou, Y., Tucci, S. & Akey, J. M. Analysis of human sequence data reveals two pulses of archaic Denisovan admixture. *Cell* **173**, 53-61 e59, doi:10.1016/j.cell.2018.02.031 (2018).
20. Vernot, B. & Akey, J. M. Resurrecting surviving Neandertal lineages from modern human genomes. *Science* **343**, 1017-1021,

doi:10.1126/science.1245938 (2014).
21 Sankararaman, S. *et al.* The genomic landscape of Neanderthal ancestry in present-day humans. *Nature* **507**, 354-357, doi:10.1038/nature12961 (2014).
22 Huelsenbeck, J. P. & Crandall, K. A. Phylogeny estimation and hypothesis testing using maximum likelihood. *Annual Review of Ecology & Systematics* **28**, 437-466 (1997).
23 Auton, A. & McVean, G. Recombination rate estimation in the presence of hotspots. *Genome Res* **17**, 1219-1227, doi:10.1101/gr.6386707 (2007).
24 McVean, G. A. *et al.* The fine-scale structure of recombination rate variation in the human genome. *Science* **304**, 581-584, doi:10.1126/science.1092500 (2004).
25 Hudson, R. R. Generating samples under a Wright-Fisher neutral model of genetic variation. *Bioinformatics* **18**, 337-338 (2002).
26 Fenner, J. N. Cross-cultural estimation of the human generation interval for use in genetics-based population divergence studies. *Am J Phys Anthropol* **128**, 415-423, doi:10.1002/ajpa.20188 (2005).
27 Kelleher, J., Etheridge, A. M. & McVean, G. Efficient coalescent simulation and genealogical analysis for large sample sizes. *PLoS Comput Biol* **12**, e1004842, doi:10.1371/journal.pcbi.1004842 (2016).
28 Skov, L. *et al.* The nature of Neanderthal introgression revealed by 27,566 Icelandic genomes. *Nature* **582**, 78-83, doi:10.1038/s41586-020-2225-9 (2020).
29 Ragsdale, A. P., Nelson, D., Gravel, S. & Kelleher, J. Lessons learned from bugs in models of human history. *Am J Hum Genet* **107**, 583-588, doi:doi.org/10.1016/j.ajhg.2020.08.017 (2020).
30 Chen, F.-C. & Li, W.-H. Genomic divergences between humans and other hominoids and the effective population size of the common ancestor of humans and chimpanzees. *Am J Hum Genet* **68**, 444-456, doi:doi.org/10.1086/318206 (2001).
31 Won, Y.-J. & Hey, J. Divergence population genetics of chimpanzees. *Molecular biology and evolution* **22**, 297-307, doi:10.1093/molbev/msi017 (2005).
32 Harney, É., Patterson, N., Reich, D. & Wakeley, J. Assessing the performance of qpAdm: a statistical tool for studying population admixture. *Genetics* **217**, doi:10.1093/genetics/iyaa045 (2021).
33 Schaefer, N. K., Shapiro, B. & Green, R. E. An ancestral recombination graph of human, Neanderthal, and Denisovan genomes. *Sci Adv* **7**, eabc0776, doi:10.1126/sciadv.abc0776 (2021).
