# Supplementary material for: Refining models of archaic admixture in Eurasia with ArchaicSeeker 2.0
Source: Nat Commun. 2021 Oct 29;12:6232. doi: 10.1038/s41467-021-26503-5 (PMC8556419; doi:10.1038/s41467-021-26503-5)
Supplement: Supplementary file 3 — Description of Additional Supplementary Files [file 41467_2021_26503_MOESM3_ESM.pdf]

Description of Additional Supplementary Files

File Name: Supplementary Data 1
Description: Functional annotation and enrichment of genes located in the high-frequency Neanderthal-like regions that are shared by Eurasian populations. We conducted a functional enrichment analysis for genes overlapping with the high-frequency (>0.3) Neanderthal-like regions shared between Europeans and East Asians, based on the Disease Ontology, Gene Ontology, and KEGG pathways provided in clusterProfiler v3.10.1. The “qvalues” are estimated for preventing high false discovery rate (FDR) in multiple testing.

File Name: Supplementary Data 2
Description: Functional annotation and enrichment of genes located in the high-frequency Neanderthal-like regions that are specific in diverse Eurasian populations. We conducted a functional enrichment analysis for genes overlapping with the high-frequency (>0.3) Neanderthallike regions specifically identified in Europeans or East Asians, based on the Disease Ontology, Gene Ontology, and KEGG pathways provided in clusterProfiler v3.10.1. The “qvalues” are estimated for preventing high false discovery rate (FDR) in multiple testing.

File Name: Supplementary Data 3
Description: Functional annotation and enrichment of genes located in the high-frequency Denisovan-like regions that are specific in Papuans. We conducted a functional enrichment analysis for genes overlapping with the high-frequency (>0.3) Denisovan-like regions in Papuans, based on the Disease Ontology, Gene Ontology, and KEGG pathways provided in clusterProfiler v3.10.1. The “qvalues” are estimated for preventing high false discovery rate (FDR) in multiple testing.

File Name: Supplementary Data 4
Description: Replication of reported introgressed regions in the Oceanic population (reported in ref.16) using ArchaicSeeker 2.0.

File Name: Supplementary Data 5
Description: Enrichment of the archaic-like alleles associated with gene expression in each tissue. The Neanderthal-like alleles and the Denisovan-like alleles were analyzed independently. For each population group, two subsets of loci were analyzed, including all archaic loci and those with archaic allele frequency ≥ 10%. Empirical P-values are shown in the table, which were obtained by a one-sided empirical test and corrected using the Benjamini–Hochberg procedure accounting for all tissues. The EUR group consists of 503 European samples from CEU, FIN, GBR, IBS, and TSI; The SAS group includes 403 South Asian samples from GIH, ITU, STU, and PJL; The EAS group integrates 405 East Asian samples from CHB, CHS, JPT, and CDX; The OCE group is represented by 30 Papuan samples.

File Name: Supplementary Data 6
Description: Regions with depleted archaic ancestry. A two-tail test was performed to identify those genomic regions with extremely rare introgression sequences.

File Name: Supplementary Data 7
Description: Replication of reported archaic deserts (reported in ref. 16, 17, 26, and 49).

File Name: Supplementary Data 8

Description: Functional enrichment of genes in the archaic deserts. Functional enrichment analysis was carried out based on the Disease Ontology, Gene Ontology, and KEGG pathways provided in clusterProfiler v3.10.1. The “qvalues” are estimated for preventing high false discovery rate (FDR) in multiple testing. Functional categories with qvalue < 0.05 are significantly enriched for the archaic deserts.
