# Supplementary material for: Refining models of archaic admixture in Eurasia with ArchaicSeeker 2.0
Source: Nat Commun. 2021 Oct 29;12:6232. doi: 10.1038/s41467-021-26503-5 (PMC8556419; doi:10.1038/s41467-021-26503-5)
Supplement: Supplementary file 12 — Reporting Summary [file 41467_2021_26503_MOESM12_ESM.pdf]

nature portfolio

Corresponding author(s): Shuhua Xu

Last updated by author(s): Sep 24, 2021

Reporting Summary

Nature Portfolio wishes to improve the reproducibility of the work that we publish. This form provides structure for consistency and transparency in reporting. For further information on Nature Portfolio policies, see our Editorial Policies and the Editorial Policy Checklist.

Statistics

For all statistical analyses, confirm that the following items are present in the figure legend, table legend, main text, or Methods section.

n/a	Confirmed	
☐	☒	The exact sample size (*n*) for each experimental group/condition, given as a discrete number and unit of measurement
☐	☒	A statement on whether measurements were taken from distinct samples or whether the same sample was measured repeatedly
☐	☒	The statistical test(s) used AND whether they are one- or two-sided<br>*Only common tests should be described solely by name; describe more complex techniques in the Methods section.*
☒	☐	A description of all covariates tested
☒	☐	A description of any assumptions or corrections, such as tests of normality and adjustment for multiple comparisons
☐	☒	A full description of the statistical parameters including central tendency (e.g. means) or other basic estimates (e.g. regression coefficient) AND variation (e.g. standard deviation) or associated estimates of uncertainty (e.g. confidence intervals)
☒	☐	For null hypothesis testing, the test statistic (e.g. *F*, *t*, *r*) with confidence intervals, effect sizes, degrees of freedom and *P* value noted<br>*Give P values as exact values whenever suitable.*
☒	☐	For Bayesian analysis, information on the choice of priors and Markov chain Monte Carlo settings
☒	☐	For hierarchical and complex designs, identification of the appropriate level for tests and full reporting of outcomes
☒	☐	Estimates of effect sizes (e.g. Cohen's *d*, Pearson's *r*), indicating how they were calculated

Our web collection on statistics for biologists contains articles on many of the points above.

Software and code

Policy information about availability of computer code

Data collection	We do not generate new datasets in this study. All datasets are downloaded from public resources without using any softwares.
Data analysis	The software mentioned in the manuscript are listed as follows: AdmixTools 7.0.2, ArchaicSeeker 2.0, MultiWaver 2.1, PLINK 1.9, Python 3.7, R 3.5.1, R 3.6.1, SHAPEIT 2, SimAncestry 1.0, clusterProfiler 3.10.1, ms, msprime 1.0.2.<br>The github web-links of the customs codes developed in this study : https://github.com/Shuhua-Group/.

For manuscripts utilizing custom algorithms or software that are central to the research but not yet described in published literature, software must be made available to editors and reviewers. We strongly encourage code deposition in a community repository (e.g. GitHub). See the Nature Portfolio guidelines for submitting code & software for further information.

Data

Policy information about availability of data

All manuscripts must include a data availability statement. This statement should provide the following information, where applicable:

- Accession codes, unique identifiers, or web links for publicly available datasets
- A description of any restrictions on data availability
- For clinical datasets or third party data, please ensure that the statement adheres to our policy

The 1000 Genomes Project dataset used in this study are available at [ftp://ftp.1000genomes.ebi.ac.uk/vol1/ftp/release/20130502/]. The EGDP dataset used in this study are available under accession code PRJEB12437 [https://evolbio.ut.ee/CGgenomes_VCF/]. The SGDP dataset used in this study are available under accession code PRJEB9586 [https://www.ncbi.nlm.nih.gov/bioproject/PRJEB9586/]. The Altai Denisovan data used in this study are available at [http://cdna.eva.mpg.de/neandertal/altai/]. The Altai Neanderthal data used in this study are available under accession code ERP002097 [http://cdna.eva.mpg.de/neandertal/altai/]. The Siberian Ust'-Ishim data used in this study are available at [http://cdna.eva.mpg.de/ust-ishim/]

Field-specific reporting

Please select the one below that is the best fit for your research. If you are not sure, read the appropriate sections before making your selection.

☒ Life sciences ☐ Behavioural & social sciences ☐ Ecological, evolutionary & environmental sciences

For a reference copy of the document with all sections, see nature.com/documents/nr-reporting-summary-flat.pdf

Life sciences study design

All studies must disclose on these points even when the disclosure is negative.

Sample size	The sample size used in this study contains 2000 samples from the 1000 Genomes Project dataset, 279 samples from the SGDP datasets, 402 samples from the EGDP datasets, one Denisovan, one Altai Neanderthal, and one Ust'-Ishim. Archaic introgression inference was performed for each population from the 1000 Genomes Project (n = ~100 for each) and for the combined continental group from the SGDP and EGDP datasets (n > 30 for major groups), with sufficient sample size to get adequate statistical power.
Data exclusions	No sample was excluded.
Replication	Not relevant. The current study is a non-experimental research. The custom codes developed in this study do not involve random number when estimating the introgressed segments.
Randomization	Not relevant. No experimental group allocation.
Blinding	No relevant blinding. No group allocation.

Reporting for specific materials, systems and methods

We require information from authors about some types of materials, experimental systems and methods used in many studies. Here, indicate whether each material, system or method listed is relevant to your study. If you are not sure if a list item applies to your research, read the appropriate section before selecting a response.

Materials & experimental systems

n/a	Involved in the study
☒	☐ Antibodies
☒	☐ Eukaryotic cell lines
☒	☐ Palaeontology and archaeology
☒	☐ Animals and other organisms
☒	☐ Human research participants
☒	☐ Clinical data
☒	☐ Dual use research of concern

Methods

n/a	Involved in the study
☒	☐ ChIP-seq
☒	☐ Flow cytometry
☒	☐ MRI-based neuroimaging
